# Comprehensive Modeling of Multimode Fiber Sensors for Refractive Index Measurement and Experimental Validation

**DOI:** 10.1038/s41598-018-24153-0

**Published:** 2018-04-12

**Authors:** Haris Apriyanto, Gautier Ravet, Olivier D. Bernal, Michel Cattoen, Han Cheng Seat, Valérie Chavagnac, Frederic Surre, James H. Sharp

**Affiliations:** 1 LAAS-CNRS, Université de Toulouse, CNRS, INP, Toulouse, France; 2 Politeknik Negeri Indramayu, Indramayu, Indonesia; 3 GET - UMR5563, OMP, Université de Toulouse, CNRS, IRD, Toulouse, France; 4 School of Mathematics, Computer Science and Engineering, University of London, London, UK; 5 Systems, Power and Energy Research Division, School of Engineering, University of Glasgow, Glasgow, G12 8QQ UK

**Keywords:** Engineering, Applied optics

## Abstract

We propose and develop a comprehensive model for estimating the refractive index (RI) response over three potential sensing zones in a multimode fiber. The model has been developed based on a combined ray optics, Gaussian beam, and wave optics analysis coupled to the consideration of the injected interrogating lightwave characteristics and validated experimentally through the realization of three sensors with different lengths of stripped cladding sections as the sensing region. The experimental results highly corroborate and validate the simulation output from the model for the three RI sensing zones. The sensors can be employed over a very wide dynamic RI range from 1.316 to over 1.608 at a wavelength of 1550 nm, with the best resolution of 2.2406 × 10^−5^ RI unit (RIU) obtained in Zone II for a 1-cm sensor length.

## Introduction

The intensive effort invested in laser and optical fiber research since the 1960’s has led to an exponential growth in the development of optical fiber sensor (OFS) technologies. The aftermath of a successful trial experiment on low-loss optical fiber debuting in the 1970’s has since motivated researchers to exploit fiber optics for numerous sensing and measurement techniques. This motivation can also, in large part, be attributed to certain advantages of the optical fiber over more conventional electro-mechanical sensor technologies, such as its immunity to electromagnetic interference, small size and light weight, electrical passivity at the sensing probe or head, multiplexing potential, and remote sensing capability^1–3^.

OFSs have been successfully exploited, either as extrinsic devices, i.e. the optical fiber simply acts as a waveguide to transmit a useful optical signal, or as an intrinsic sensor with the fiber acting directly as the sensing element subject to a modulation of the interrogating lightwave’s optical properties (optical intensity, phase, propagation direction and velocity, etc.) as a function of the physical quantity being measured^1,4^. OFSs have thus been developed for many applications such as for sensing various physical, biological, and chemical parameters. As a chemical sensor, the OFS is commonly employed for sensing chemical processes, monitoring environmental conditions, and pollution parameters^4^ due to its robustness to relatively harsh ambient conditions and its chemical inertness while offering explosion-free security since the sensor requires no electrical power at the sensing point. Moreover, the OFS offers rapid continuous real-time *in-situ* measurement potential for remote sensing.

In chemical sensing, one sensor type that is frequently employed is the bulk refractive index (RI) sensor or refractometer for quantifying the concentration level of various aqueous solutions, such as sucrose, salt, glycerol, dimethyl sulfoxide (DMSO), methanol, aceto-nitrile, etc.^5^, in addition to measuring other parameters like temperature since the RI of a medium is generally temperature-dependent^6–10^. Further, the advent of the sol-gel technique has facilitated the synthesis of bespoke sensitized coatings that can be deposited on the surface of an optical fiber as a thin film in replacement of the cladding^11–13^. This approach can allow the fiber-based refractometer to selectively measure specific organo-chemical species. For example, using a thin functionalized film of Polydimethylsiloxane (PDMS) incorporating cryptophane-A or cryptophane-E supramolecules as the sensitive region^14^, the refractometer can be used to detect methane (CH_4_) concentration. The cryptophane-based molecular traps will absorb or entrap the CH_4_ molecules, and reversibly produce a bond in the bulk polymeric material that will induce variation in the RI of the sensitized region as a function of CH_4_ concentration.

Various operating principles have been exploited for fiber RI sensing such as employing Fresnel reflection at the end face of a single-mode fiber (SMF)^8^, surface plasmon resonance (SPR)^15–19^, tapered optical fibers^20–23^, multimode interference (MMI)^20,24–26^, fiber Brag gratings (FBGs)^27,28^, long-period gratings (LPGs)^29^, etc. A further technique that is simple, fast to realize, and cost effective is by employing a totally or partially stripped-cladding core as the sensing region in a multimode fiber (MMF)^30–34^. Previous work has reported typical dynamic ranges from 1.33–1.55 RIU with resolutions between 6.48 × 10^−3^ RIU and 9.68 × 10^−4^ RIU for different fiber types^30,31^. The de-cladded section is generally substituted by a sensing medium which interacts with lightwaves propagated in the fiber. Through preliminary experimental results^34^, we have demonstrated and reported the classification of three operating zones in the RI response in a stripped-cladding step index MMF subject to variation in the external medium’s RI in contact with the sensing element, according to three different sensing mechanisms. This three-zone phenomenon is theoretically reviewed, explained and experimentally validated in this paper, the outcome of which can potentially be employed for detecting RI variation of any external medium subject to or undergoing perturbation. More advantageously, it can enable RI measurement over a very wide dynamic range, virtually unlimited by the RI of the MMF core since detection is always or still possible even when the external medium’s RI is greater than that of the core.

In previous work^34^, Zone I has been defined for a range of variation of the external medium’s RI up to the cladding index of the MMF. The variation of the power guided in the fiber, induced by the change in RI is due to evanescent wave absorption (EWA). In Zone II, where the induced RI variation is situated between the cladding RI (*n*_*cl*_) and the core RI (*n*_*co*_), i.e. greater than *n*_*cl*_ but less than *n*_*co*_, we have demonstrated that there are two concurrent optical (or in this case sensing) phenomena occurring caused by EWA and the reduction of the number of propagation modes due to the modification of the critical angle as a consequence of the RI change in the sensing or external medium. The final RI response is Zone III where the RI of the external medium is higher than *n*_*co*_. The power guided for this condition is due to the reflection of the “lost” lightwaves, which have been transmitted to the external medium interface, back into the fiber core. This is the most logical hypothesis to explain the existence of the RI response for indices higher than the core index (equally demonstrated experimentally by Mukherjee *et al*.^35^), since under this operating condition, there is no (further) EWA as no further lightwave propagation exists by total internal reflection (TIR). The explanation of the RI sensing behavior is backed up by experimental evidence, via the transmission characteristics of the propagating lightwaves through the fiber.

In this paper, a comprehensive study is carried out on step-index MMFs for RI sensing where the light source is a single-mode DFB laser diode pigtailed to a single-mode fiber (SMF). A microscope objective (MO) is employed to inject the required lightwave modes into the MMF under investigation. From this launch condition, we propose an effective model for this system and define the three sensing zones by stripping the MMF cladding. The model is adapted from the combination of the analytical equations for wave optics, beam optics (Gaussian beam), and ray optics. The motivation in proposing this model is to overcome the complexity in the simulation of propagating lightwaves in MMFs by wave optics, especially in the case of simulation by the finite element method (FEM) in 3D since the mesh size must be smaller than the wavelength. Furthermore, the dimension of MMFs will contribute to a very high number of unknowns, implying a very significant computational load which is difficult to resolve by generic computing. Hence, the simplest way to explain the propagation principle and the power loss phenomenon in MMFs as RI sensors is by ray optics. Nevertheless, EWA and the beam distribution injected into MMFs, which cannot be determined using ray optic analysis, can be compensated by concurrently applying the analytical equations of the evanescent wave, and employing the Gaussian beam equations to define the injected beam power distribution in an MMF. Using the model developed, we demonstrate accurate simulation results which are corroborated by experimental data.

## Results

### Propagation in multimode optical fibers

The laser beam is propagated in an optical fiber by TIR which occurs as the beam injected into the fiber core is incident at the core-cladding interface at an angle higher than the critical angle. This critical angle, *θ*_*c*_, can be explained by Snell’s law as a function of the RI contrast (*n*_*c*o_ and *n*_*cl*_, respectively) as follows:

1 $${\theta }_{c}={\sin }^{-1}(\frac{{n}_{cl}}{{n}_{co}})$$

For an SMF, only one mode propagates in the fiber. However, in an MMF numerous modes can propagate. The propagating modes are modes that are incident at the core-cladding interface with an angle between *θ*_*c*_ and 90°. Theoretically, in wave optics the number of modes (*M*) for a very large number of modes in a step-index MMF can be estimated by^36^

2 $$M\cong \frac{{V}^{2}}{2}$$

where *V* is an all-important parameter for determining whether a fiber is single or multi mode. For *V* < 2.405, the fiber will be single-mode while an MMF has a *V*-parameter greater than 2.405. The value of *V* relates to *n*_*co*_, *n*_*cl*_, the fiber core radius (*a*), and the wavelength (*λ*) of the injected beam as

3 $$V=\frac{2\pi a}{\lambda }\sqrt{{{n}_{{co}}}^{2}-{{n}_{{cl}}}^{2}}$$

where $\sqrt{{{n}_{{co}}}^{2}-{{n}_{{cl}}}^{2}}$ is also known as the numerical aperture (*NA*), which defines the acceptance angle within which the injected beam via the MO can be propagated by the fiber or radiated by the fiber. Hence, to obtain all the modes over the entire possible acceptance angle, the injected beam must be adapted to the *NA* of the step-index MMF.

### Experimental set-up

**Figure 1:**
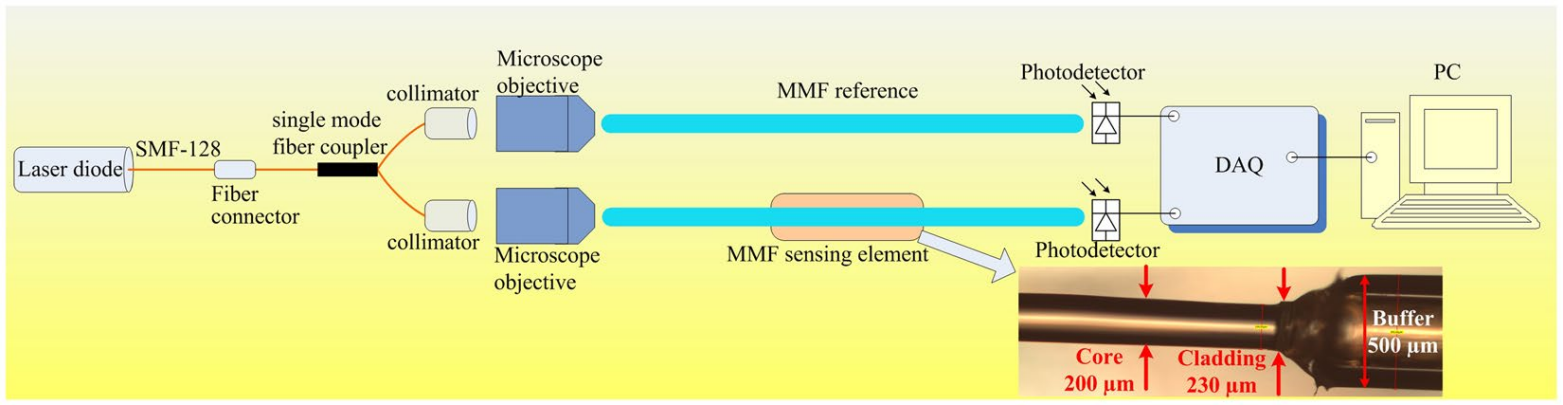
Experimental schematic set-up of MMF-based sensor for measuring RI under differential configuration to compensate common-mode noise. The inset shows an enlarged image of the stripped-cladding core of the MMF used in this work, with the junctions between the core and cladding, and between the cladding and buffer/coating clearly illustrated. There is, in particular, no spurious transitions between the core and cladding (i.e. no obvious filaments left over from the cladding removal process).

During the measurements, a differential probe configuration is employed, as shown in Fig. 1, with one MMF serving as the reference fiber and the other MMF the sensing fiber to compensate common-mode noise produced by both MMFs. The sensor, based on the ratiometric intensities from the two MMFs, thus measures a transmitted power for the reference MMF and the power in the sensing MMF arm using two identical *Ge*-type Thorlabs PDA50B photodetectors (PDs) with a peak spectral response within 800–1800 nm. A single-mode fiber-pigtailed DFB laser diode from Modulight, Inc. emitting at 1550 nm and driven by precision current and temperature controllers is employed to interrogate the fibers. The laser output beam is divided by a single-mode fiber coupler to obtain two equal or symmetrical beams which are first collimated and then injected into the reference and sensing MMF arms of the sensor via two identical MOs with an *NA* of 0.65 in order to transmit the launched beam over all acceptance angles in the fibers. The MMFs are plastic-clad silica (PCS) fibers exhibiting an *NA* of 0.48 ± 0.02, with 200 µm core diameter and 230 µm cladding diameter. The output power detected from both MMF arms are then transmitted to a dedicated computer via a 14-bit 2-MHz Agilent data acquisition system (DAQ) for further processing.

The MMF sensing region is realized by thermally removing a certain length of the fiber cladding (as shown in the inset of Fig. 1), followed by a simple procedure of cleaning the fiber core using acetone and isopropanol solutions. Although the buffer still contains some filaments of the coating material, both the core and cladding sections are smooth (i.e. continous) and undamaged, and hence will not influence or affect the propagation of the launched lightwaves within the sensing region. Three different sensing lengths of 1 cm, 2.5 cm, and 4 cm of stripped fiber cladding have been prepared. This sensing area is thus sensitive to variations in the RI value through variations of the power transmitted as a function of the medium’s RI. As previously mentioned, 3 different conditions or sensing phenomena can intervene in the MMF RI sensor. These detection mechanisms are further elaborated below.

### Sensing mechanism in Zone I

**Figure 2:**
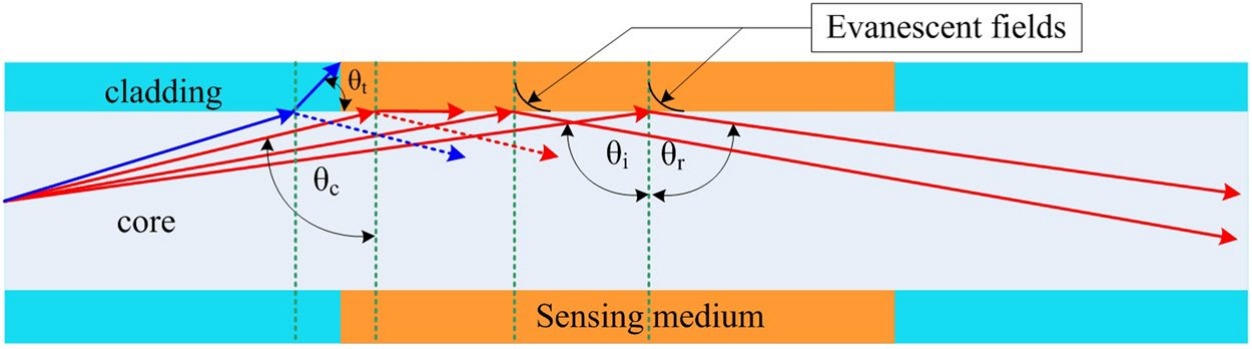
Propagation by TIR in an MMF for incident lightwave angles larger than critical angle, *θ*_*c*_.

Consider the case of the sensing medium having an RI value (*n*_*sm*_) less than the cladding RI. Under this condition, all the modes propagate in the fiber core up to the sensing zone by the phenomenon of TIR, at which point evanescent waves will be generated along the core-cladding interface, as shown in Fig. 2.

When the incident angle (*θ*) is larger than *θ*_*c*_, almost all power is confined within the core while the previously transmitted part of the beam propagates as evanescent waves penetrating into the cladding. Hence, the resulting power transmitted to the fiber end while propagating over a stripped cladding of length, *L*, can be predicted by^37^

4 $${P}_{L}={P}_{0}\,.\,\exp (-\gamma L)$$

where *γ* is the evanescent wave’s attenuation coefficient and *P*_0_ the initial power propagated by TIR in the MMF. For a weakly guiding fiber, i.e. for a fiber with only a small RI difference between the cladding and core, *γ* is a function of the bulk absorption coefficient (*α*) and the fractional power in the cladding and core (*r*), i.e. the ratio of the power in the cladding to the total power, given by *γ* = *r.α*. Now, *r* can be estimated by a simple equation related to the number of modes (*M*) using *r* = *4/(3* $\sqrt{M})$^37^. Nevertheless, since the MMF is generally a non-weakly guiding fiber, *γ* thus has to be re-defined to account for the number of modes with a transmission coefficient (*T*) penetrating into the cladding over a number of reflections per unit length (*N*) as a function of the critical angle of the sensing medium (*θ*_*csm*_ = *sin*^*−1*^(*n*_*sm*_*/n*_*co*_)) in replacement of the cladding and the incident angle (*θ*), for *θ* > *θ*_*c*_^37^ such that

5 $$\gamma =NT$$

where

6 $$N=\frac{\cot \,\theta }{2a}$$

7 $$T=\frac{\alpha \lambda {{\rm{n}}}_{{sm}}\,\cos \,\theta }{\pi {n}_{co}^{2}{\cos }^{2}{\theta }_{csm}\sqrt{{\cos }^{2}{\theta }_{csm}-{\cos }^{2}\theta }}$$

Further, by adapting Equations (5), (6) and (7) to solve Equation (4) for non-weakly guiding fibers, the power over a sensing length, *L*, can be calculated as a function of the angle over all the acceptance angles (*θ, π/*2) as

8 $${P}_{L}=\sum _{{\theta }_{i}=\theta }^{\pi /2}{P}_{0}({\theta }_{i})\,.\,\exp (-N({\theta }_{i})T({\theta }_{i})L)$$

**Figure 3:**
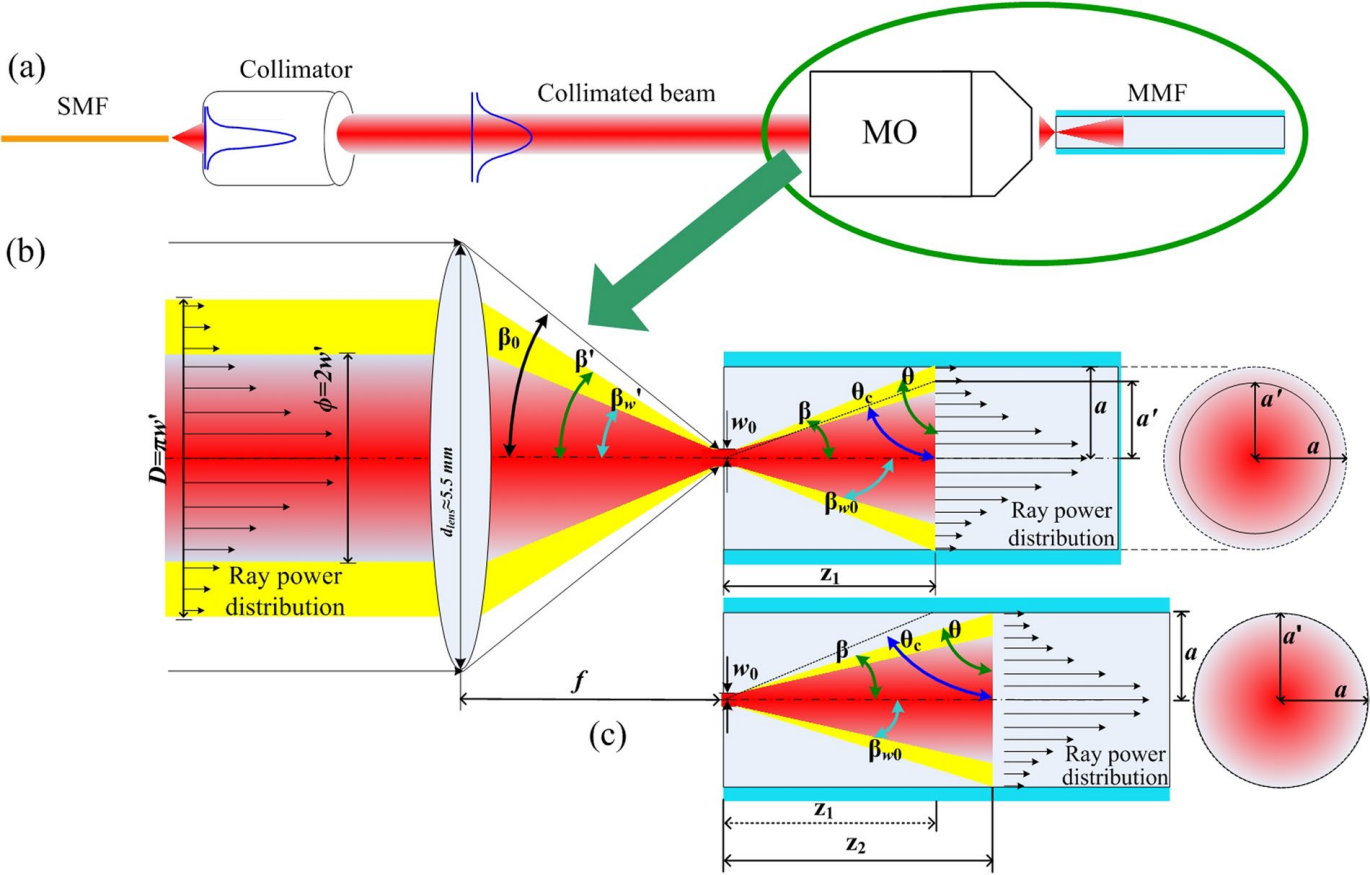
Schematic of beam launching conditions into MMF where the red beam represents the portion of the beam which transmits ~86% of the total power (or 1/e^2^ beam intensity) and which is used to determine the spot size radius (*w*_0_), while the yellow beam represents over 99% of the total beam power. (**a**) Collimated beam from lead-in SMF with Gaussian power distribution is focused by MO into the MMF for more specific launch parameters when (**b**) *θ* < *θ*_*c*_ of the fiber and when (**c**) *θ* > *θ*_*c*_ of the fiber.

In this case, due to the comparatively low power transmission loss in the MMF over a length of ~80 cm, the absorption effect by the fiber cladding is assumed to be negligible (attenuation ~0.028 dB/m from manufacturer’s datasheet). Hence, the initial power can be taken as the combined power of all the rays launched into the fiber under TIR as shown in Fig. 3.

The single-mode Gaussian beam from the SMF is collimated by an F220FC-1550 collimator to a beam diameter (*ϕ* = *2w*′)^38^ of 2.1 mm (red beam in Fig. 3(b) to the left of the lens) while the diameter of the total beam power can be calculated by *D* = *πw*′. This collimated beam (consisting of both the red and yellow beams) is next focused by an MO with an *NA* of 0.65 to match all possible acceptance angles for propagation in the MMF. The beam waist (*w*_0_) can be estimated by Equation (9) which relates *λ*, the focal length of the MO (*f*), and the spot radius of the collimated beam (*w*′) in the form^36^

9 $${w}_{0}=\frac{\lambda f}{\pi w^{\prime} }$$

As illustrated in Fig. 3(b), when the total lens area of the MO is completely illuminated by the input collimated beam, the injection angle *β*′ = *β*_0_, can be obtained from the relationship, *NA* = *n*_*air*_ *sin β*_0_. However, since the input beam does not illuminate the entire lens area due to the lens diameter (*d*_*lens*_ ≈ 5.5 mm) being larger than the total beam diameter (*D* = *πw*′ = 3.2987 mm), *β*′ cannot, consequently, be calculated directly using the *NA* equation which gives a *β*_0_ value of ~40.54°. By simplifying the MO to a one-lens system, *f* can first be obtained using Equation (10) relating *β*_0_ to the lens radius (*r*_*lens*_ = 0.5*d*_*lens*_), after which *β*′ can then be obtained for a given *D* value via Equation (11)

10 $$f={r}_{lens}\,\cot \,{\beta }_{0}$$

11 $$\beta ^{\prime} ={\tan }^{-1}(\frac{0.5D}{f})$$

In ray optics, mode propagation in the MMF is represented by individual rays at specific angles injected into and, subsequently, propagating in the fiber, and can thus be simply analyzed by considering the beam with an incident *θ* at the core-cladding interface. Rays which have incident angles less than *θ*_*c*_ of the fiber will not be propagated in the core, when compared to rays with angles greater than *θ*_*c*_. These latter rays will be guided in the core by TIR. To simplify the propagation model, the incident beam characteristics illustrated in Fig. 3(b) and (c) are analyzed. The beam with a propagating angle *β* in the MMF will arrive at the core-cladding interface at a distance *z*_*i*_, calculated using

12 $${z}_{i}=a\,\cot \,\beta $$

where *β* (=90° − *θ*) is related to *β*′ by Snell’s law via *n*_*air*_ *sin β*′ = *n*_*co*_ *sin β* and *i* = 1 or 2. The incident Gaussian beam can be represented as a set of rays with different individual optical power values exhibiting a Gaussian distribution, where the outer rays have a lower power than the rays towards the center fiber axis. However, since the onset of TIR starts at *z*_*i*_, the diffraction effects of the Gaussian beam are annulled at *z*_*i*_^39^, thus the power distribution for each angle of the individual ray propagated along the MMF follows on from the ray power distribution at point *z*_*i*_, as will be explained below. Consequently, the sensing region must be placed after the point *z*_*i*_. Then, by using Equation (12) and considering that the beam is injected into the center of the fiber core with *a* = 100 µm, for a 1 cm sensing length, the beam will be incident at the interface of the sensing area at least once up to *θ* = 89.427°. Hence, almost all of the rays injected will reach the sensing region. Further, for a Gaussian beam injected from air and expanded in the fiber core (silica with *n*_*co*_ = 1.444), the beam spot radius at *z*_*i*_ (*w*_*zi*_) can be calculated by^40^

13 $${w}_{zi}={w}_{0}\sqrt{1+{(\frac{\lambda {z}_{i}}{{n}_{co}\pi {{w}_{0}}^{2}})}^{2}}$$

The optical intensity at *z*_*i*_ (*I*_*zi*_) and the optical power injected into the fiber (*P*_*inj*_) can respectively then be given by^36,38^

14 $${I}_{{z}_{i}}={I}_{0}\,\exp (\,-\,2{a}^{2}/{w}_{{z}_{i}}^{2})$$

15 $${P}_{inj}=1-\exp (\,-\,2{a}^{2}/{w}_{{z}_{i}}^{2})$$

Subsequently, as illustrated in Fig. 3(b), when *θ* is lower than *θ*_c_ of the fiber, the total power arrives at the core-cladding interface at the point *z*_1_. Only the optical power between *θ* and *θ*_*c*_ will exit the core while the remaining power will still be guided in the fiber core by TIR represented as the beam with a radius *a′* which can be calculated by simple trigonometry as

16 $$a^{\prime} ={z}_{i}\,\cot \,{\theta }_{c}$$

Substituting the new radius for *a*′ in Equation (15) then allows *P*_0_, the power guided by TIR for *θ* < *θ*_*c*_ of the fiber, to be obtained as

17 $${P}_{0}=1-\exp (-2a{^{\prime} }^{2}/{w}_{{z}_{i}}^{2})$$

However, if *θ* > *θ*_*c*_, all the incident beams arriving in the sensing region are reflected by TIR. Thus, *a*′ = *a* and *P*_*inj*_ = *P*_0_. Equation (17) must then employ *z*_*i*_ = *z*_2_ (see Fig. 3(c)), and *w*_*zi*_ takes on *w*_*z*2_. Since the surface of the MO lens is not entirely illuminated by the collimated beam (*d*_*len*s_ ≈ 5.5 mm, and *D* ≈ 3.2987 mm), *θ* can be obtained by applying Equations (10, 11) and Snell’s law. Here, *θ* = 71.57° is higher than *θ*_*c*_ (70.60°), thus all injected beams are guided by TIR. Consequently, the previous definition of Zone I^34^ has to be re-evaluated, i.e. the limit of Zone I is modified to the RI value (*n*_*eq*_) which is equivalent to the angle of the incident beam (via *θ* = *sin*^*−1*^(*n*_*eq*_*/n*_*co*_)). Further, *w*_*z*2_ = 67.898 µm is obtained via Equation (13) for the point *z*_2_ = 300.14 µm and *w*_0_ = 1.51 µm obtained by Equation (9). Then, to solve Equation (8), *P*_0_ in Equation (17) can be discretized into individual ray powers between *θ* and 90° (i.e. *P*_0_(*θ, π/*2)) at the point *z*_*2*_ by varying *a*’ in discrete steps (from 100 µm to 0) corresponding to the *θ* value for each ray. Substituting these step variations of *a*’ into Equation (17) then allows the decremental power difference, *P*_0_(*θ, π/*2), to be calculated for each individual ray with a corresponding set of *T*(*θ, π/*2) and *N*(*θ, π/*2) parameters. Finally, these powers are summed over *θ* − 90° in Equation (8), to obtain the total transmitted power (*P*_*L*_) subject to EWA for a given sensing length *L*.

### Sensing mechanisms in Zone II

**Figure 4:**
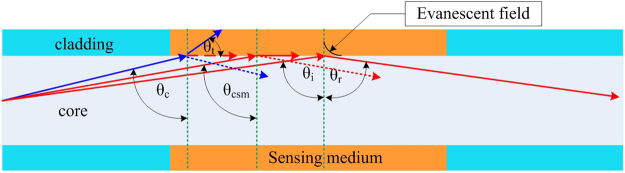
Illustration of sensing mechanisms in Zone II due to the combination of EWA and mode power loss by the modification of the critical angle (*θ*_*csm*_). Under initial conditions, the blue line represents the mode propagated in the MMF by TIR at critical angle; however, when *n*_*sm*_ increases, *θ*_*c*_ is modified to *θ*_*csm*_ and this mode will be transmitted through the medium and lost to the exterior. The remaining rays incident at an angle greater than the new *θ*_*csm*_ will be guided in the MMF by TIR and concurrently subject to EWA. The reflection at the sensing medium-air interface is negligible since the medium thickness is more than 20 times the core diameter in addition to its strong absorption at 1550 nm. The dotted blue and red lines represent very weak reflections obtained by Fresnel equations at the core-medium boundary which are neglected for simplifying the model for Zone II.

For the second operating condition or Zone II response, where the RI of the sensing medium (*n*_*sm*_) falls between the cladding RI and the core RI (i.e., *n*_*cl*_ < *n*_*sm*_ < *n*_*co*_), two optical phenomena concurrently influence the optical power loss^34^ (see Fig. 4): (1) reduction of the number of propagation modes due to the modification of the critical angle (from *θ*_*c*_ to *θ*_*csm*_) upon variation of *n*_*sm*_ and (2) EWA since TIR is always present in this operating zone, identical to the explanation of losses described for Zone I.

**Figure 5:**
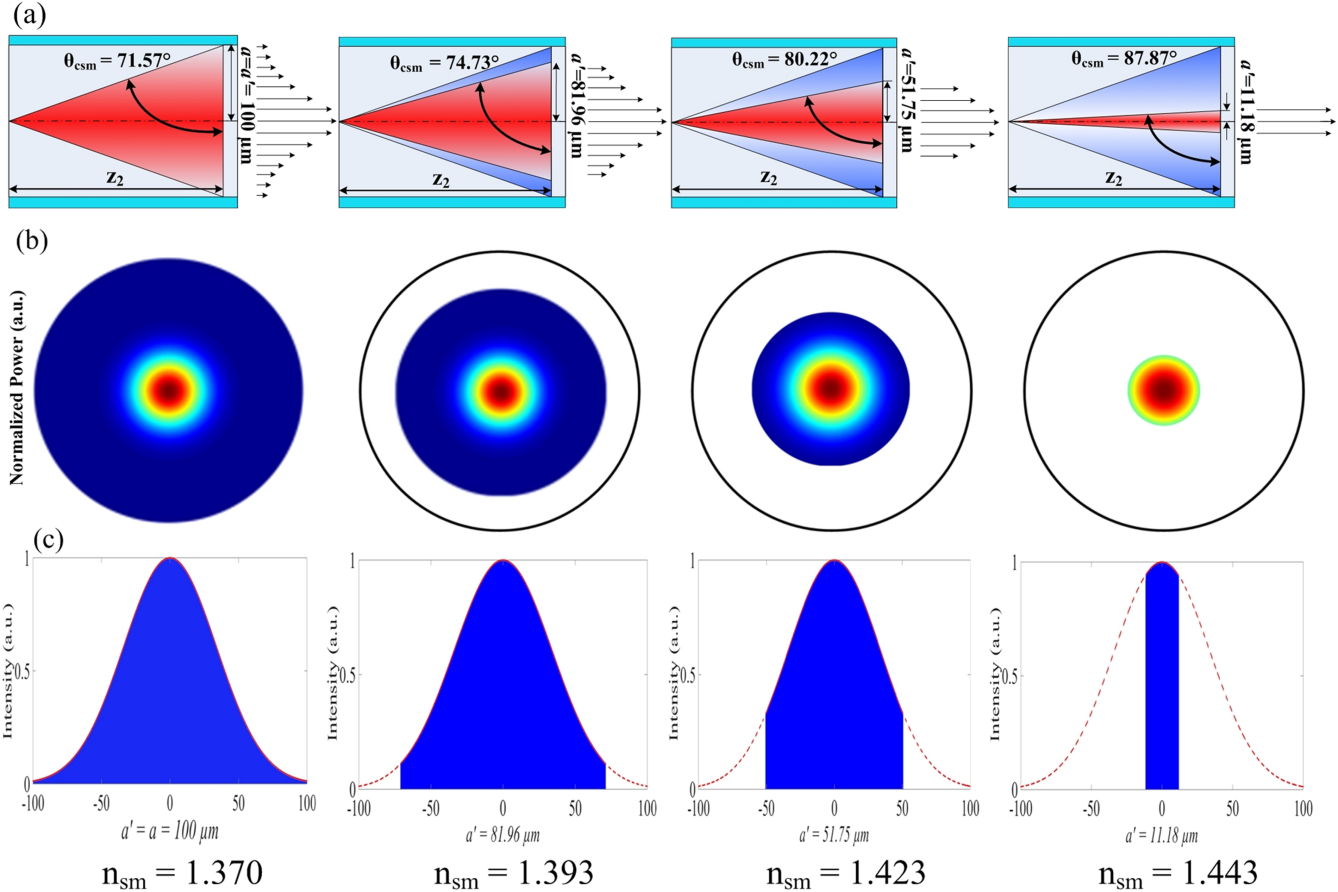
Evolution of optical power and intensity in the MMF RI sensor for various values of *n*_*sm*_ by modification of the critical angle, *θ*_*csm*_, for *n*_*cl*_ < *n*_*sm*_ < *n*_*co*_. (**a**) increasing *n*_*sm*_ will increase *θ*_*csm*_ which reduces the acceptance angle of the propagating beam, (**b**) power in transversal plane decreases for increasing *n*_*sm*_, and (**c**) illustrates decreasing optical intensity over different *a’* obtained by Equation (14).

The reduction in the number of propagation modes occurs for increasing *n*_*sm*_ since the critical angle also increases as *θ*_*csm*_ = *sin*^*−1*^(*n*_*sm*_*/n*_*co*_). This leads to a decrease in the guided power by TIR (*P*_0_) as the beam radius *a’* at point *z*_*2*_ decreases (see Equation (17)), subsequently reducing the sectional area at this point as illustrated by Fig. 5. The beam radius, *a*′, is calculated by the following formula:

18 $$a^{\prime} ={z}_{2}\,\cot ({\sin }^{-1}({n}_{sm}/{n}_{co}))={z}_{2}\,\cot \,{\theta }_{csm}$$

The model for Zone II is subsequently obtained for different values of *P*_0_(*θ*_*csm*_, *π/*2) as a result of the reduction of the number of modes by modification of the critical angle via Equation (17) while the remaining power for rays from *θ*_*csm*_ to 90° which are subject to EWA can be estimated using Equation (8) for a given *α* and sensing length *L* as in Zone I.

### Sensing mechanism in Zone III

**Figure 6:**
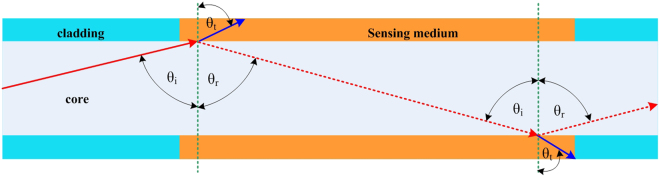
Optical power guided by external reflection in Zone III when *n*_*sm*_ > *n*_*co*_. The majority of the power is transmitted to the exterior while a small portion is reflected back into the core. This mechanism again arises when the ray intersects the interface between the sensing region and the external medium. This phenomenon is more prominent for rays which have incident angles close to 90°.

The last condition is Zone III where *n*_*sm*_ > *n*_*co*_. Here, propagation by TIR is no longer supported although a very small portion of optical power can still be guided due to the phenomenon of external reflection. This can be explained by Fresnel equations for a beam incident at an interface between two media of different RI values as illustrated in Fig. 6.

Since *n*_*sm*_ > *n*_*co*_, most of the optical power of the ray will be transmitted to the exterior. Furthermore, since the sensing medium is much thicker than the core diameter, the transmitted ray will not be back-reflected into the core. However, a small proportion of power is still reflected back into the core by the external reflection mechanism at the core-sensing medium interface. This proportion of back-reflected power can be obtained by calculating the reflectivity (*R*) for the light containing both *P*-polarization (*r*_*p*_) and *S*-polarization (*r*_*s*_) components using the Fresnel equations below^36^

19 $${r}_{p}=\frac{{n}_{sm}\,\cos \,{\theta }_{i}-{n}_{co}\,\cos \,{\theta }_{t}}{{n}_{sm}\,\cos \,{\theta }_{i}+{n}_{co}\,\cos \,{\theta }_{t}}\,\,{r}_{s}=\frac{{n}_{co}\,\cos \,{\theta }_{i}-{n}_{sm}\,\cos \,{\theta }_{t}}{{n}_{co}\,\cos \,{\theta }_{i}+{n}_{sm}\,\cos \,{\theta }_{t}}$$

20 $$R=\frac{({{r}_{p}}^{2}+{{r}_{s}}^{2})}{2}$$

with *θ*_*i*_, *θ*_*t*_ and *θ*_*r*_ the incident, transmitted and reflected angles, respectively. Thus, for a given *R*, the power of the individual ray guided in the core for Zone III as a function of the total number of reflections (*NL*) at the core-sensing medium interface can be estimated by

21 $${P}_{L}=\sum _{{\theta }_{i}=\theta }^{\pi /2}{P}_{0}({\theta }_{i}){R}^{N({\theta }_{i})L}$$

### Simulation and experimental results

**Figure 7:**
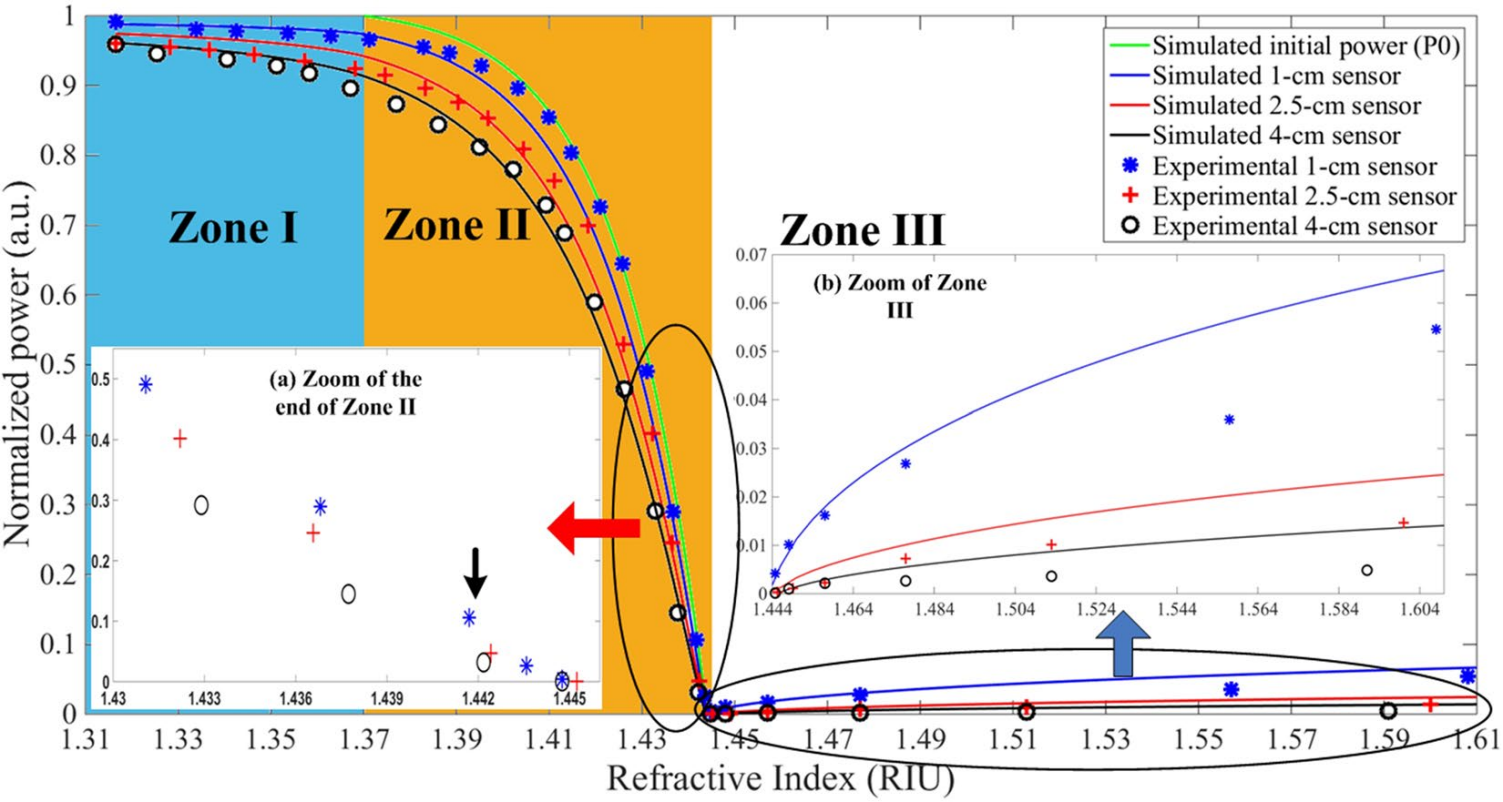
Response of three sensors in three different Zones. Continuous lines are simulation results. Green line represents variation of total initial power (*P*_0_(*θ*, *π/*2)) in Zone II due uniquely to modification of critical angle without EWA. Blue, red, and black lines are simulation results for the 1-cm, 2.5-cm, and 4-cm sensors, respectively. Dotted lines with ‘’, ‘’, and ‘o’ represent experimental results for 1-cm, 2.5-cm and 4-cm sensors, respectively. The black arrow in inset (a) indicates the power response slowly dropping beyond this measured RI point.

A suite of simulations is then performed based on the above models and validated against experimental data for the three different sensing Zones. Figure 7 illustrates the high level of agreement between simulations and the experimentally-measured data. The sensing medium is composed of a combination of glycerol-water mixture which is adjusted to obtain different values of RI from 1.3164 to 1.4571 at a wavelength of 1550 nm^5^. For RI values beyond 1.4571, calibrated RI liquids were employed. Furthermore, different glycerol-water concentration levels exhibit varying absorption coefficients (*α*) with that of water being experimentally determined in a controlled environment to be 11.49 cm^−1^, and that of glycerol being 11.10 cm^−1^. Assuming a linear relationship between the imaginary part or extinction coefficient of the mixture’s RI (*k*_*tot*_) and its volume fraction, which is a function of the glycerol (*f*_*g*_) and water (*f*_*w*_) fractions (with *f*_*g*_ + *f*_*w*_ = *1*), the extinction coefficients of glycerol (*k*_*g*_) and water (*k*_*w*_), we can estimate^41^ *k*_*tot*_ = *f*_*g*_ *k*_*g*_ + *f*_*w*_ *k*_*w*_. The composite absorption coefficient of the solution can then be obtained via^41^

22 $${\alpha }_{tot}=\frac{4{k}_{tot}\pi }{\lambda }$$

**Figure 8:**
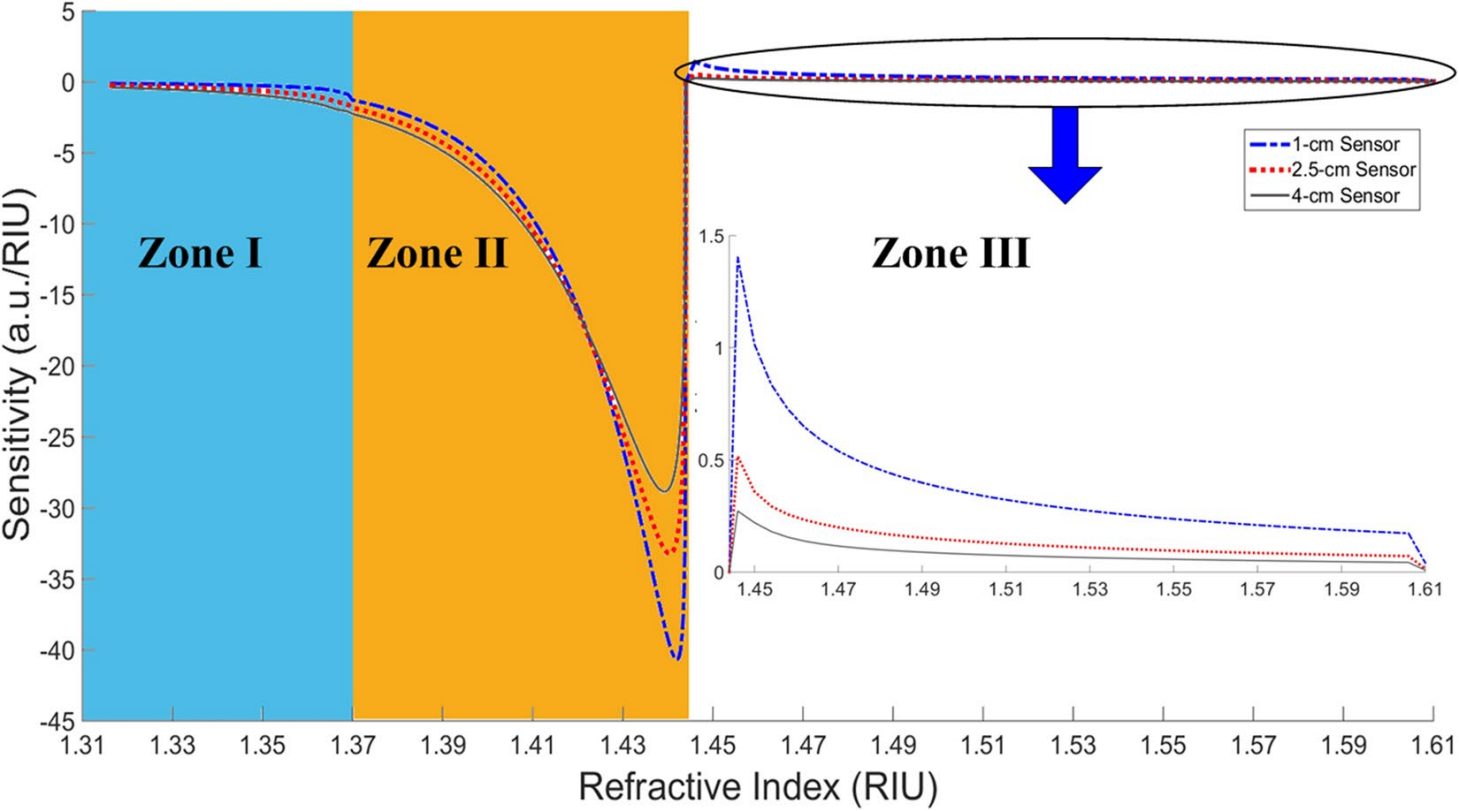
Sensitivity curves of RI sensors in three different Zones by derivation of the experiment curves (dP/dRI). ‘’, ‘’, and ‘—’, represent the sensitivity response for the 1-cm, 2.5-cm, and 4-cm sensors, respectively.

The sensitivity curves of the RI sensors are next obtained for each length of sensing region and plotted in Fig. 8. These curves are obtained by differentiating the fitted curves through the experimental data in Fig. 7 based on our models for each zone.

Here, the sensitivity for each sensor has been obtained by first fitting specific response curves to the 3 respective sensing Zones in Fig. 8 followed by their differentiation with respect to RI.

## Discussion

Three complete models for the three different sensing zones of an MMF refractometer for RI measurement have, for the first time, been developed as a function of the injected lightwave characteristics into the fiber. The mathematical models for Zone I and Zone II are similar as a consequence of direct influences from EWA. Although the principal model for EWA in Equation (8) has been employed for both Zone I and Zone II, they are subject to different initial powers *P*_0_ (in Zone I, *P*_0_ = *P*_0_(*θ, π*/2) for *θ* > *θ*_*c*_ or *P*_0_ = *P*_0_(*θ*_*c*_*, π*/2) if *θ* < *θ*_c_, while in Zone II, *P*_0_ = *P*_0_(*θ*_*csm*_*, π*/2)).

For Zone I response, *P*_0_ is conserved for each of the rays over the entire RI response of the sensing medium as all the rays within the sensing region are guided by TIR. Hence, if *θ* is less than *θ*_*c*_ of the MMF, the injected rays will propagate in the sensing region, with a critical angle of ~70.60° (equivalent to a cladding RI of 1.362), as predicted by Equation (1). However, in this work, since *θ* = 71.57°, corresponding to an equivalent RI value of ~1.370, the Zone I response is extended from our preliminary results^34^ to this new RI value. Subsequently, all incident rays from MO will be guided in the MMF core by TIR. The simulated results plotted in Fig. 7 demonstrate good agreement with the experimental data, with the blue, red, and black continuous lines corresponding to simulation for a 1 cm, 2.5 cm, and 4 cm stripped fiber cladding, respectively, while the symbols ‘’, ‘’, and ‘o’ represent the respective experimental measurements. Longer sensing lengths will incur higher losses, hence the power measured decreases when the RI increases due to the increasing transmission (*T*) penetrating into the cladding, i.e. more power is absorbed at higher RI, as described by Equations (7) and (8).

For Zone II, where *n*_*cl*_ < *n*_*sm*_ < *n*_*c*o_ (*n*_*co*_ = 1.444), the variation of the optical power guided for different values of *n*_*sm*_ is due to two optical phenomena as illustrated in Fig. 4. Here, the rays arrive in the sensing region guided by TIR since the incident beam angle is 71.54° and thus, the beginning of Zone II is equivalent to an RI value of ~1.370. The green line in Fig. 7 represents the simulated initial power variation *P*_0_(*θ*_*csm*_, *π*/2) as a function of RI due uniquely to the modification of the critical angle and does not reflect the influence of EWA. However, the contribution from EWA in the sensing region will lead to a smaller additional decrease in the total guided power along the fiber. This is clearly demonstrated through simulations in the form of continuous blue, red, and black lines for *L* = 1 cm, 2.5 cm, and 4 cm, respectively, as predicted by Equation (8). Now, identical to the EWA phenomenon in Zone I, the longer sensing region in Zone II is subject to higher absorption (Fig. 7). The high agreement between the simulated results and experimental measurements clearly demonstrates that the higher power losses occurring in Zone II are due to the contribution of both modification of the critical angle and EWA. The contribution of losses due to modification of the critical angle is also found to be more dominant than EWA in Zone II. Further, the power variation is observed to increase slowly at the beginning of Zone II toward the end of the Zone where it then decreases sharply. This sharp decrease is due to the power distribution of the Gaussian beam which increases sharply from the sides or wings of the distribution (i.e. top hat radius) towards the peak (i.e. circular aperture) of the Gaussian curve, but which decreases at the peak area (i.e. circular aperture area)^38^. Hence, the optical power response in Fig. 7 will exhibit a flat response or inflexion point at exactly the boundary between Zone II and Zone III. The last operating regime of the MMF refractometer is Zone III which can be employed for probing a medium with *n*_*sm*_ > *n*_*co*_. Under this condition, there is no propagation by TIR. Nevertheless, the incorporation of the Fresnel relations in Equations (19) and (20) into Equation (21) postulates the existence of guided power in the core by the phenomenon of external reflection, in particular, from the rays which have incident angles *θ* in the sensing medium close to 90°. Although the guided power is small, this will increase for further increases in the value of RI beyond that of *n*_*co*_. This is validated experimentally in Fig. 7 through the measurement of increasing power at the fiber output end as *n*_*sm*_ increases beyond that of the core. Complementary to this, Equation (21) further correctly predicts the higher power guided over shorter lengths of the sensing region in the MMF since there are fewer reflections (*NL*) which, in turn, reduce the transmitted power or rays to the exterior through the sensing medium. Nonetheless, the respective discrepancies between the simulations and the experimental results for the different sensing lengths in Zone III could be due to non-ideal conditions during the experimental study, such as the existence of very small bends in the MMF which can alter the optical power distribution and/or modify the beam quality factor (M^2^) by inducing changes to the MMF index profile in the bending area^42^.

The sensitivity curves plotted in Fig. 8 illustrate the best sensitivity being achieved in Zone II for the shortest sensing length (i.e. 1-cm stripped fiber cladding) as the shortest sensor is subject to the least EWA. Consequently it suffers higher losses through modification of the critical angle (mode loss mechanism) as predicted by the continuous green line in Fig. 7. Toward the end of the Zone II response, there is more power variation for a small RI variation. The three sensitivity curves first increase sharply from the middle of Zone II, and then decline less sharply toward almost the end of this Zone before decreasing back toward zero at the core index (1.4444) which is the minimum point of the optical power response (see Fig. 7). The respective inflexion points of the sensitivity curves in Fig. 8 occur before the end of Zone II and correspond to the beginning of the decreasing gradient of the optical power response as described above with respect to the circular aperture area of the Gaussian beam. A zero sensitivity value could potentially be obtained when the power response in Fig. 7 occurred over very small RI variations (i.e. tending toward 0 or ΔRI → 0) of the sensing medium. However, since the practical RI variations induced in this work cannot be infinitesimal, the sensitivity of the three sensing lengths obtained at the end of Zone II cannot reach zero value. Nevertheless, the sensitivity curves as plotted in Fig. 8 decrease toward zero when the measured RIs approach the end of Zone II (i.e. close to the core index).

Conversely, for Zone I, the longest sensor has better sensitivity since the principally EWA contributions to the sensing mechanism are cumulative over the entire sensing length. There is thus more absorption by the longest sensor resulting in the largest power variation as a function of RI. In Zone III, on the contrary, the shortest sensor is again more sensitive since less power is lost to the exterior, and consequently more power is guided in the fiber core. Hence, according to Equations (19), (20) and (21), the guided power increases with increasing RI, with the increasing power being sharper at the beginning of Zone III, and subsequently declines less sharply with increasing RI. Thus, the sensitivity decreases with increasing RI in the sensing medium since the rate of power variation with RI decreases. However, although this sensor theoretically has virtually unlimited dynamic range for operation over Zone III, its performance could be limited to only a certain RI range when the sensitivity approaches the noise level.

Based on the sensitivity curves in Fig. 8, the sensor resolution has been determined with respect to the measurement noise level using the 6*σ*-definition (6 times RMS noise corresponding to ~99.7% confidence level)^43^ with only ~0.3% of the samples lying outside of this distribution. The best resolution achieved is 2.2447 × 10^−5^ RIU by the 1-cm sensor in Zone II. It is also in this Zone that the 2.5-cm and 4-cm sensors have the best relative resolutions of 2.9847 × 10^−5^ RIU and 3.2517 × 10^−5^ RIU, respectively, compared to the other two Zones. For Zone I, the best resolution is achieved by the 4-cm long sensor with a minimum detection level of 1.6116 × 10^−3^ RIU while the 1-cm and 2.5-cm sensors are capable of resolutions of 5.5905 × 10^−3^ RIU and 1.7528 × 10^−3^ RIU, respectively. The achievable sensor resolution in Zone I is not very high due to the induced multiplicative noise from multiple reflections in the sensing region as well as the relatively low sensitivity in this zone. For the Zone III response, the noise level is relatively low since most of the injected power, including the noise from multiple reflections, are transmitted to the exterior. In this Zone, the normalized noise level ranges from approximately 1.2 × 10^−4^ (a.u.) − 1.6 × 10^−4^ (a.u.) and is typically dominated by the measured photodetector noise normalized to 8.66 × 10^−5^ (a.u.). The minimum RI resolution that can be detected in Zone III are 1.0031 × 10^−3^ RIU, 1.8070 × 10^−3^ RIU, and 3.1920 × 10^−3^ RIU for the 1-cm, 2.5-cm, and 4-cm sensing lengths, respectively. The low resolution obtained in Zone III can simply be understood by the low sensitivity in this zone as a consequence of higher losses arising from external reflection, as explained previously.

## Conclusions

We have proposed three models based on a combination of analytical wave optics to obtain the EWA equation, Gaussian beam optics to describe the injected power distribution, and ray optics to explain the principle of optical mode losses in an MMF configured for refractive index measurements. These models have been adapted to consider the three different sensing mechanisms as a function of the relative cladding and core RIs. Nonetheless, the models for Zone I and Zone II are fundamentally similar, whereby both Zones are subject to EWA as the fundamental loss mechanism. However, Zone II involves the additional phenomenon of critical angle modification, which modifies the model employed through the use of different values of *P*_0_(*θ*_*csm*_, *π*/2) as a function of RI variation. Further, since the incident beam angle in the fiber is higher than *θ*_*c*_, the boundary between Zone I and Zone II is no longer the cladding RI value (1.362), but the RI which corresponds to the incident beam angle (RI~1.370). Finally, the model for Zone III exploits Fresnel relations, where the rays propagating in the sensing region exhibit different power variations as a function of the ray angle with respect to their initial *P*_0_(*θ, π/*2) for an acceptance angle carried over from Zone II.

The experimental measurements performed are found to validate the simulation results derived from our models to describe the three different optical sensing mechanisms in the MMF refractometer. The results confirm that in Zone I, the sensing mechanism is uniquely via EWA which induces the largest losses in the sensor with the longest sensing region. For Zone II, the best sensor resolution of 2.2406 × 10^−5^ RIU is achieved for the 1-cm sensor. The sharp power decrease occurring in Zone II is a consequence of the losses induced by modification of the critical angle for a Gaussian beam, where most power is concentrated at the center axis (top hat area), corresponding to an incident angle close to 90° (i.e. close to the core RI). However, at the beginning of Zone II, the losses are relatively small due to the weaker power distribution at the edges of the Gaussian beam which corresponds to an RI approaching that of the cladding, such that the losses are dominated by EWA. Last, but not least, in Zone III, when the external reflection mechanism intervenes, only a relatively small initial guided power exists, which subsequently increases as the external medium’s RI increases due to the increasing reflectivity of the rays back into the fiber core. As predicted by our model, the longest sensor will guide less optical power since more reflections are induced by a longer sensing region, resulting in more rays being transmitted toward the exterior.

Future work will undertake modeling and analysis of the MMF refractometer described above for practical *in-situ* applications, for example, for detecting dissolved methane in aqueous environments. This would involve employing a thin PDMS film incorporating Cryptophane-A molecular traps as the sensing region whose bulk RI (~1.40 at 1550 nm) varies with varying methane concentration. We anticipate a potentially achievable measurement resolution of 1.5592 × 10^−4^ RIU at 1550 nm by the 1-cm MMF sensor. This translates to ~28.35 nM of dissolved methane for a specified sensitivity of 5.5 × 10^−6^ RIU/nM^44^. This performance would also be improved several-fold by exploiting lock-in detection techniques.

## Methods

### Simulation method

**Figure 9:**
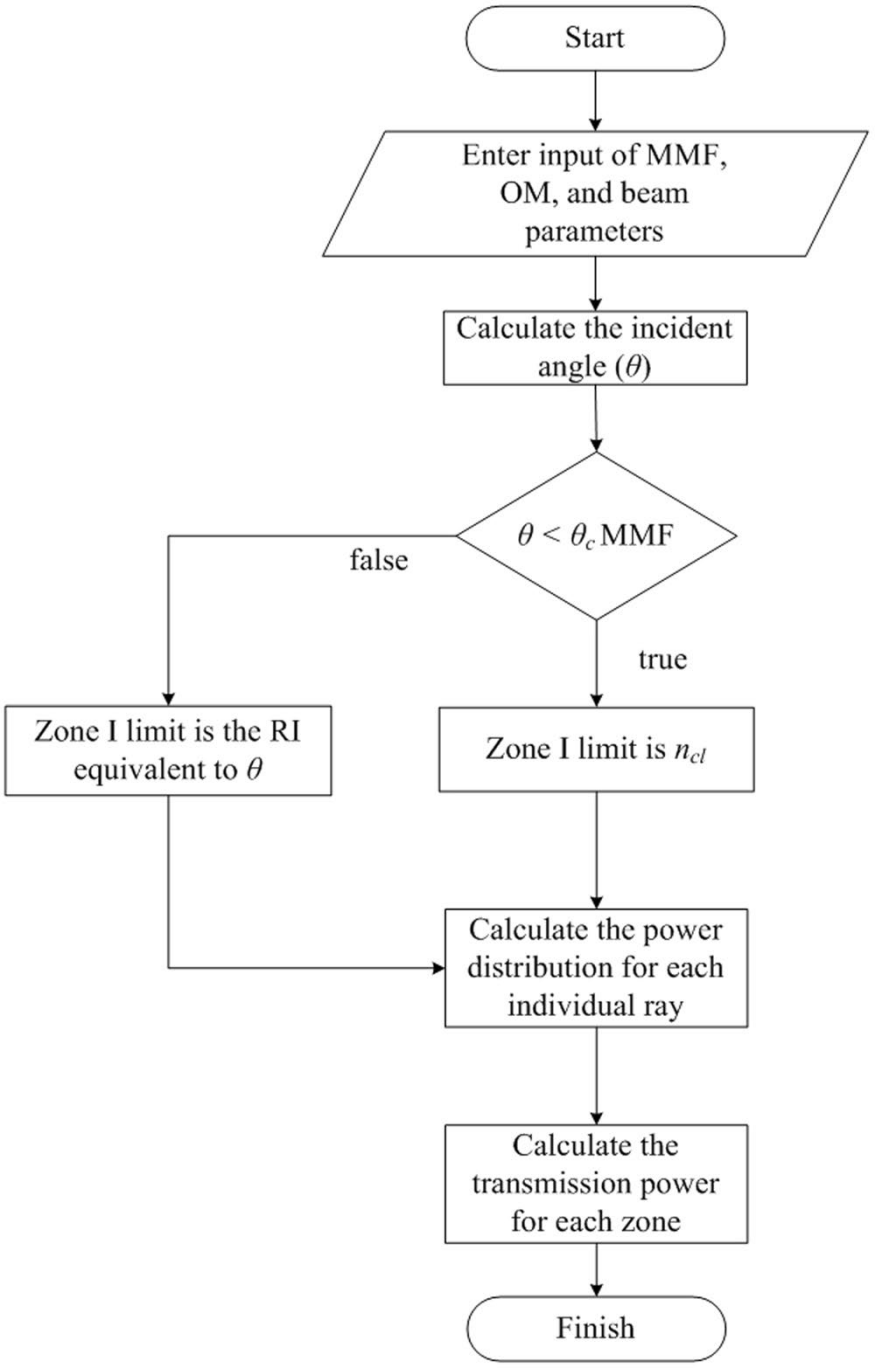
Flow chart to calculate RI-dependent guided power.

Simulations are realized for 3 sensing lengths of the stripped-cladding MMF through the use of the equations in the Results Section which are adapted to efficiently and accurately describe the sensing mechanisms induced by the three optical phenomena in the fiber. The simulation results illustrate the sensor response in terms of the normalized optical power as a function of RI. The simulation procedure has been carried out according to the following flow chart shown in Fig. 9.

// Start program

// Enter input parameters such as *a, n*_*air*_*, n*_*co*_*, n*_*cl*_*, λ, NA, ϕ, D, r*_*lens*_*, n*_*w*_*, n*_*gly*_*, and β*_0_

**//** Incident angle (*θ* = 90° − *β*) obtained by determining *f* in Equation (10), β′ in Equation (11) and from Snell’s law

// If *θ* < *θ*_*c*_, Zone I limit is cladding RI value, but if *θ* > *θ*_*c*_, Zone I limit is RI equivalent of *θ*

**//** To obtain power distribution, calculate spot radius with Equation (16) then determine power with Equation (17), and discretize this power for individual rays.

// For Zone I, obtain transmitted power by integrating *P*_0_(*θ*, *π*/2) using Equation (8). If incident angle < *θ*_*c*_, integrate *P*_*0*_ for all angles from *θ*_*c*_ to 90°. For incident angle > *θ*_*c*_, integrate *P*_0_(*θ*, *π*/2) for all incident angles from *θ* to 90°.

// For Zone II, transmitted power obtained by integrating *P*_0_(*θ*_*csm*_, *π/*2) from an angle equivalent to desired RI value (for critical angle modification) up to 90° and employing Equations (6) and (7) for EWA.

// For Zone III, transmitted power estimated by integrating power distribution *P*_0_(*θ*, *π*/2) and using Equations (19), (20) and (21).

### Experimental system

The interrogating laser beam from a Modulight, Inc. laser diode is injected into the MMFs as shown in Fig. 3. Three-axis MDE122 Martock translation stages (50 nm resolution) from Elliot Scientific are used for the launching of the laser beam into the MMF. The beam injection condition is optimized using adjustable-gain transimpedance photodetectors from Thorlabs Inc., when the highest transmitted power is detected at the fiber output end. The experimental set-up was mounted and stripped over at least 5 times to demonstrate the consistency and repeatability of the results.

### Measurement procedure

The stripped-cladding area is used to measure the variation of RI in liquids. A combination of pure distilled water and pure glycerol from Sigma-Aldrich is used to obtain a range of RIs from 1.3164 (RI of water) to 1.4571 at a wavelength of 1550 nm. This information is obtained from the supplementary material provided by J. E. Saunders *et al*.^5^ which can be accessed from http://faculty.chem.queensu.ca/people/faculty/loock/publications.htm. According to the data, a plot of RI as a function of the concentration of glycerol in water at 25 °C is traced, from which we then obtained the relationship between RI and the glycerol concentration levels. A homogeneous sensing medium (water + glycerol) is then obtained with the aid of a magnetic stirrer operating for ten minutes for each mixing process. This sensing medium is next calibrated against a reference Hanna Instruments optical refractometer incorporating automatic temperature compensation from 10 °C to 40 °C at an operating wavelength of 589 nm. The data from the reference refractometer are subsequently adjusted through the relationship obtained in Hoyt^45^ to determine the mixture’s RI at 1550 nm wavelength. For RI values beyond 1.4571, calibrated oils from Cargille Laboratories have been employed in our experimental measurements.

### Data availability

Both simulation and experimental data are publicly available in the “supplementary information” file.

## Electronic supplementary material

Supplementary Information
