# Author Correction: Comprehensive Modeling of Multimode Fiber Sensors for Refractive Index Measurement and Experimental Validation

**DOI:** 10.1038/s41598-021-99941-2

**Published:** 2021-11-09

**Authors:** Haris Apriyanto, Gautier Ravet, Olivier D. Bernal, Michel Cattoen, Han Cheng Seat, Valérie Chavagnac, Frederic Surre, James H. Sharp

**Affiliations:** 1 LAAS-CNRS, Université de Toulouse, CNRS, INP, Toulouse, France; 2 Politeknik Negeri Indramayu, Indramayu, Indonesia; 3 GET - UMR5563, OMP, Université de Toulouse, CNRS, IRD, Toulouse, France; 4 School of Mathematics, Computer Science and Engineering, University of London, London, UK; 5 Systems, Power and Energy Research Division, School of Engineering, University of Glasgow, Glasgow, G12 8QQ UK

Correction to: *Scientific Reports* 10.1038/s41598-018-24153-0, published online 12 April 2018

The original version of this Article contained errors in Equation 7, which was incorrectly given as:

$$T=\frac{\alpha \lambda {\mathrm{n}}_{\mathrm{co}}\mathrm{cos}\theta }{\pi {{n}_{sm}}^{2}{\mathrm{cos}}^{2}{\theta }_{csm}\sqrt{{\mathrm{cos}}^{2}{\theta }_{csm}-{\mathrm{cos}}^{2}\theta }}$$

The correct Equation 7 appears below.

$$T=\frac{\alpha \lambda {\mathrm{n}}_{sm}\mathrm{cos}\theta }{\pi {n}_{co}^{2}{\mathrm{cos}}^{2}{\theta }_{csm}\sqrt{{\mathrm{cos}}^{2}{\theta }_{csm}-{\mathrm{cos}}^{2}\theta }}$$

As a result of the changes to Equation 7, the Abstract,

“The sensors can be employed over a very wide dynamic RI range from 1.316 to over 1.608 at a wavelength of 1550 nm, with the best resolution of 2.2447 × 10^−5^ RI unit (RIU) obtained in Zone II for a 1-cm sensor length.”

now reads:

“The sensors can be employed over a very wide dynamic RI range from 1.316 to over 1.608 at a wavelength of 1550 nm, with the best resolution of 2.2406 × 10^−5^ RI unit (RIU) obtained in Zone II for a 1-cm sensor length.”

Additionally, in Figure 5a, the unit of fiber diameter “μm” was incorrectly given as “mm”.

The original Figure 5 and accompanying legend appear below.

**Figure 5:**
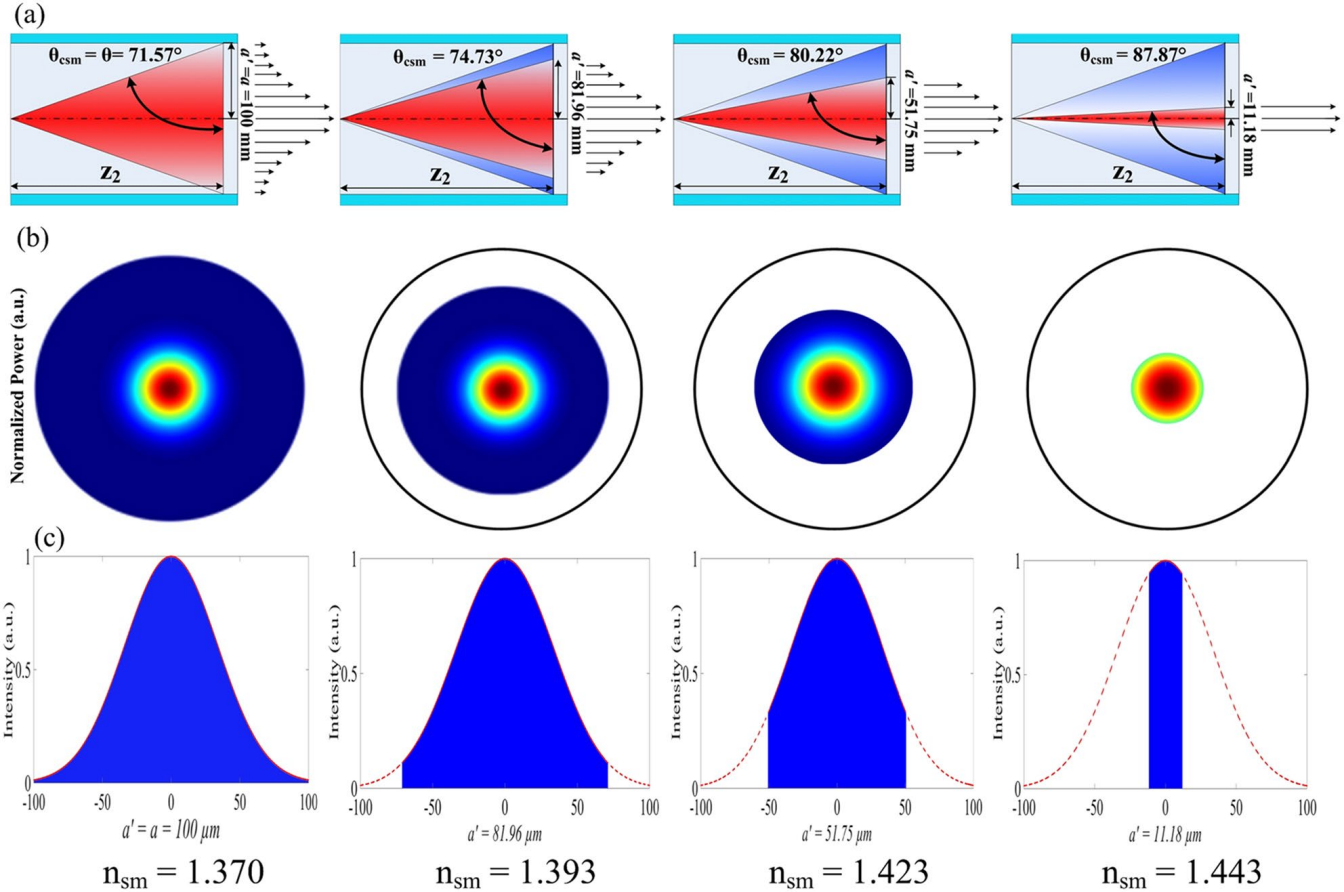
Evolution of optical power and intensity in the MMF RI sensor for various values of *n*_ *sm*_ by modification of the critical angle, *θ*_*csm*_ , for *n*_*cl*_ < *n*_*sm*_ < *n*_*co*_ . (**a**) increasing *n*_*sm*_ will increase *θ*_*csm*_ which reduces the acceptance angle of the propagating beam, (**b**) power in transversal plane decreases for increasing *n*_*sm*_ , and (**c**) illustrates decreasing optical intensity over different *a’* obtained by Equation (14).

In the final paragraph of the Discussion,

“It is also in this Zone that the 2.5-cm and 4-cm sensors have the best relative resolutions of 2.9919 × 10^−5^ RIU and 3.2634 × 10^−5^ RIU, respectively, compared to the other two Zones. For Zone I, the best resolution is achieved by the 4-cm long sensor with a minimum detection level of 1.5438 × 10^−3^ RIU while the 1-cm and 2.5-cm sensors are capable of resolutions of 5.1952 × 10^−3^ RIU and 1.7462 × 10^−3^ RIU, respectively.”

now reads:

“It is also in this Zone that the 2.5-cm and 4-cm sensors have the best relative resolutions of 2.9847 × 10^−5^ RIU and 3.2517 × 10^−5^ RIU, respectively, compared to the other two Zones. For Zone I, the best resolution is achieved by the 4-cm long sensor with a minimum detection level of 1.6116 × 10^−3^ RIU while the 1-cm and 2.5-cm sensors are capable of resolutions of 5.5905 × 10^−3^ RIU and 1.7528 × 10^−3^ RIU, respectively.

In the second paragraph of the Conclusions,

“For Zone II, the best sensor resolution of 2.2447 × 10^−5^ RIU is achieved for the 1-cm sensor.”

now reads:

“For Zone II, the best sensor resolution of 2.2406 × 10^−5^ RIU is achieved for the 1-cm sensor.”

Lastly, as a result of the changes to Equation 7, the data in Table S1, S2, S3, S5, S6 and S7 in the Supplementary Information was incorrect.

The original Supplementary Information file is available below.

The original Article and accompanying Supplementary Information file has now been corrected.

## Supplementary Information

Supplementary Information.

