# Supplementary material for: Comprehensive Modeling of Multimode Fiber Sensors for Refractive Index Measurement and Experimental Validation
Source: Sci Rep. 2018 Apr 12;8:5912. doi: 10.1038/s41598-018-24153-0 (PMC5897385; doi:10.1038/s41598-018-24153-0)
Supplement: Supplementary file 1 — Supplementary Information [file 41598_2018_24153_MOESM1_ESM.docx]

**Comprehensive Modeling of Multimode Fiber Sensors for Refractive Index Measurement and Experimental Validation**

Haris Apriyanto^1,2^, Gautier Ravet^1^, Olivier D. Bernal^1^, Michel Cattoen^1^, Han Cheng Seat^1*^, Valérie Chavagnac^3^, Frédéric Surre^4^, James H. Sharp^5^

^1^LAAS-CNRS, Université de Toulouse, CNRS, INP, Toulouse, France.

^2^Politeknik Negeri Indramayu, Indramayu, Indonesia.

^3^GET - UMR5563, OMP, Université de Toulouse, CNRS, IRD, Toulouse, France.

^4^Photonics & Instrumentation Research Centre, School of Mathematics, Computer Science and Engineering, City, University of London, UK.

^5^Systems, Power and Energy Research Division, School of Engineering, University of Glasgow, Glasgow G12 8QQ, UK.

^*^seat@enseeiht.fr

**Table 1.** Experimental results for 1-cm sensing length over the three sensing zones. All measurements are repeated over 20 times with 100,000 sampling points.

| RI @589 nm | Glycerol concentration | RI @1550 nm | Normalized Power (a.u.) | Sensitivity (a.u./RIU) | Normalized Vrms noise (a.u.) | Resolution (RIU) |
| --- | --- | --- | --- | --- | --- | --- |
| 1.33299 | 0 | 1.316482 | 0.99119794 | -0.095195594 | 0.00030767 | -0.019391864 |
| 1.35062 | 0.14609008 | 1.333793 | 0.98033255 | -0.14846094 | 0.00022878 | -0.009246068 |
| 1.3594 | 0.21514446 | 1.342592 | 0.97757856 | -0.192548805 | 0.00018095 | -0.00563857 |
| 1.37026 | 0.29789025 | 1.353584 | 0.97468488 | -0.286961599 | 0.00039283 | -0.008213573 |
| 1.37936 | 0.36517441 | 1.362838 | 0.97159856 | -0.452827423 | 0.00042192 | -0.005590474 |
| 1.38753 | 0.42415596 | 1.371152 | 0.9665174 | -1.345599201 | 0.00041823 | -0.001864879 |
| 1.39908 | 0.50551309 | 1.382885 | 0.95576725 | -2.43188219 | 0.00030238 | -0.000746039 |
| 1.40457 | 0.54345164 | 1.388444 | 0.94757097 | -3.229827904 | 0.00039544 | -0.000734603 |
| 1.4114 | 0.59007608 | 1.395341 | 0.92870093 | -4.597170357 | 0.00030788 | -0.00040183 |
| 1.41925 | 0.64297519 | 1.403237 | 0.89583139 | -6.881519055 | 0.00017827 | -0.000155434 |
| 1.42582 | 0.68676777 | 1.409819 | 0.85443335 | -9.610505499 | 0.00041745 | -0.000260621 |
| 1.4308 | 0.71971722 | 1.414792 | 0.80337262 | -12.34065447 | 0.0002199 | -0.000106915 |
| 1.43698 | 0.76036419 | 1.420944 | 0.72592745 | -16.74560495 | 0.00042991 | -0.000154038 |
| 1.44193 | 0.79276526 | 1.425857 | 0.64425953 | -21.26692592 | 3.1700E-04 | -8.94346E-05 |
| 1.44719 | 0.82708058 | 1.431065 | 0.49093881 | -27.19509101 | 2.1300E-04 | -4.69938E-05 |
| 1.45301 | 0.86495485 | 1.436811 | 0.28962989 | -35.00714123 | 2.2600E-04 | -3.8735E-05 |
| 1.45798 | 0.89725547 | 1.441705 | 0.10581093 | -40.703212 | 1.5200E-04 | -2.24061E-05 |
| 1.45989 | 0.90966549 | 1.443584 | 0.0264071 | -35.97745197 | 1.3600E-04 | -2.26809E-05 |
| 1.46109 | 0.91746269 | 1.444763 | 0.00420827 | 0.53640825 | 0.00012968 | 0.001450492 |
| 1.46448 | 0.93949546 | 1.448091 | 0.01019219 | 1.20172233 | 0.0002907 | 0.001451402 |
| 1.4736 | 0.99887693 | 1.457025 | 0.01619856 | 0.75160683 | 0.00012567 | 0.001003179 |
|  |  | 1.477 | 0.02685969 | 0.47862122 | 0.0001316 | 0.001649726 |
|  |  | 1.557 | 0.03605211 | 0.22714696 | 0.00012443 | 0.003286718 |
|  |  | 1.608 | 0.05457648 | 0.10736517 | 0.00012593 | 0.007037422 |

**Table 2.** Experimental results for 2.5-cm sensing length over the three sensing zones. All measurements are repeated over 20 times with 100,000 sampling points.

| RI @589 nm | Glycerol concentration | RI @1550 nm | Normalized Power (a.u.) | Sensitivity (a.u./RIU) | Normalized Vrms noise (a.u.) | Resolution (RIU) |
| --- | --- | --- | --- | --- | --- | --- |
| 1.33299 | 0 | 1.316482 | 0.96068347 | -0.23361434 | 0.00045499 | -0.01168567 |
| 1.34507 | 0.10136031 | 1.328294 | 0.95498554 | -0.310939393 | 0.00064901 | -0.012523534 |
| 1.35345 | 0.16857047 | 1.336617 | 0.95089827 | -0.388700217 | 0.00060594 | -0.009353326 |
| 1.36318 | 0.24426603 | 1.346408 | 0.94426718 | -0.524187574 | 0.00121668 | -0.013926465 |
| 1.37389 | 0.32494212 | 1.357273 | 0.93487927 | -0.791670878 | 0.00032077 | -0.002431086 |
| 1.38459 | 0.40307741 | 1.368161 | 0.92451354 | -1.53202244 | 0.00044756 | -0.00175282 |
| 1.39088 | 0.447984 | 1.374559 | 0.91478435 | -2.186009969 | 0.00034708 | -0.00095264 |
| 1.39947 | 0.50822275 | 1.38328 | 0.89624265 | -3.193024359 | 0.00031519 | -0.000592272 |
| 1.40636 | 0.55572971 | 1.390254 | 0.8762907 | -4.351881042 | 0.00063704 | -0.000878296 |
| 1.41283 | 0.59976411 | 1.396782 | 0.85324622 | -5.833600711 | 0.00047364 | -0.00048715 |
| 1.42051 | 0.65140516 | 1.404501 | 0.80933941 | -8.260615634 | 0.00043739 | -0.000317693 |
| 1.42711 | 0.69532156 | 1.411108 | 0.76363633 | -11.11034836 | 0.00018484 | -9.98205E-05 |
| 1.43432 | 0.74289841 | 1.418299 | 0.6990874 | -15.26457351 | 0.00083712 | -0.000329044 |
| 1.44216 | 0.79426796 | 1.426085 | 0.52923788 | -21.25969475 | 0.00088798 | -0.000250609 |
| 1.44834 | 0.83457074 | 1.432201 | 0.40223545 | -27.02090317 | 0.00079974 | -0.000177583 |
| 1.45277 | 0.86339437 | 1.436574 | 0.24614318 | -31.21125507 | 0.00054629 | -0.000105018 |
| 1.4587 | 0.9019336 | 1.442414 | 0.04695708 | -31.38397171 | 0.00015612 | -2.98471E-05 |
| 1.46158 | 0.92064675 | 1.445244 | 0.00029497 | 0.32217908 | 0.00013396 | 0.002494687 |
| 1.46546 | 0.94586738 | 1.449053 | 0.00114947 | 0.39704103 | 0.00011958 | 0.001807007 |
| 1.4736 | 0.99887693 | 1.457025 | 0.00230721 | 0.26644789 | 0.00012084 | 0.002721132 |
|  |  | 1.477 | 0.00723175 | 0.18010735 | 0.00014666 | 0.00488582 |
|  |  | 1.513 | 0.01017297 | 0.12418806 | 0.00013151 | 0.006353751 |
|  |  | 1.6 | 0.01469334 | 0.0734589 | 3.2066E-05 | 0.002619089 |

**Table 3.** Experimental results for 4-cm sensing length over the three sensing zones. All measurements are repeated over 20 times with 100,000 sampling points.

| RI @589 nm | Glycerol concentration | RI @1550 nm | Normalized Power (a.u.) | Sensitivity (a.u./RIU) | Normalized Vrms noise (a.u.) | Resolution (RIU) |
| --- | --- | --- | --- | --- | --- | --- |
| 1.33299 | 0 | 1.316482 | 0.95978558 | -0.365179719 | 0.00054459 | -0.008947759 |
| 1.34205 | 0.07665072 | 1.325329 | 0.94635342 | -0.448620194 | 0.00025364 | -0.003392268 |
| 1.35729 | 0.19873311 | 1.340469 | 0.93757111 | -0.669069141 | 0.00066059 | -0.005923962 |
| 1.36788 | 0.27999401 | 1.351168 | 0.92771297 | -0.93679059 | 0.00087589 | -0.005609941 |
| 1.37494 | 0.33271362 | 1.358341 | 0.91780905 | -1.236714014 | 0.00033218 | -0.001611593 |
| 1.38368 | 0.39652043 | 1.367235 | 0.89666189 | -1.90595075 | 0.00101767 | -0.003203661 |
| 1.39333 | 0.46528756 | 1.377049 | 0.87315602 | -2.936632331 | 0.00037632 | -0.000768881 |
| 1.40221 | 0.52719616 | 1.386056 | 0.84457005 | -4.191511817 | 0.00036702 | -0.000525376 |
| 1.41099 | 0.58729388 | 1.394927 | 0.81228951 | -6.000888651 | 0.00061391 | -0.000613819 |
| 1.4182 | 0.63593805 | 1.402182 | 0.77948725 | -8.070940813 | 0.00059494 | -0.000442283 |
| 1.42539 | 0.68391344 | 1.409389 | 0.7283047 | -10.82554208 | 0.00059226 | -0.000328257 |
| 1.42928 | 0.70968053 | 1.413275 | 0.68864847 | -12.66097989 | 0.00038604 | -0.000182943 |
| 1.43587 | 0.75308089 | 1.419841 | 0.59023452 | -16.40063899 | 0.0004976 | -0.000182042 |
| 1.44238 | 0.79570512 | 1.426303 | 0.46469603 | -20.87511001 | 0.00061064 | -0.000175512 |
| 1.44904 | 0.8391282 | 1.432893 | 0.29156052 | -25.89545445 | 0.00042345 | -9.81137E-05 |
| 1.45395 | 0.87106592 | 1.437737 | 0.14468315 | -28.789427 | 0.00035917 | -7.48546E-05 |
| 1.45846 | 0.90037424 | 1.442178 | 0.03202889 | -26.27716713 | 0.00014241 | -3.25172E-05 |
| 1.46109 | 0.91746269 | 1.444763 | 0.00014526 | 0.10414824 | 0.00013303 | 0.007663999 |
| 1.46448 | 0.93949546 | 1.448091 | 0.00096785 | 0.24579291 | 0.00013076 | 0.003192029 |
| 1.4736 | 0.99887693 | 1.457025 | 0.00213901 | 0.16190594 | 0.00015811 | 0.005859328 |
|  |  | 1.477 | 0.00267084 | 0.10414819 | 0.00012702 | 0.007317362 |
|  |  | 1.513 | 0.00358808 | 0.07287114 | 0.0001421 | 0.011699941 |
|  |  | 1.591 | 0.00488893 | 0.04640004 | 0.00042248 | 0.054630986 |

**Table 4.** Simulation results for normalized initial power (P_0_)

| RI @1550 nm | Incident angle, θ (°) | Normalized power (a.u.) | ΔP, power for each ray (a.u.) |
| --- | --- | --- | --- |
| 1.37 | 71.5777682 | 0.99996886 | 4.2098E-05 |
| 1.37005 | 71.5840471 | 0.99992676 | 4.2227E-05 |
| 1.3701 | 71.5903281 | 0.99988453 | 4.2357E-05 |
| 1.37015 | 71.5966112 | 0.99984217 | 4.2486E-05 |
| 1.3702 | 71.6028964 | 0.99979969 | 4.2616E-05 |
| 1.37025 | 71.6091836 | 0.99975707 | 4.2747E-05 |
| 1.3703 | 71.6154729 | 0.99971432 | 4.2878E-05 |
| 1.37035 | 71.6217642 | 0.99967145 | 4.3009E-05 |
| 1.3704 | 71.6280577 | 0.99962844 | 4.3141E-05 |
| 1.37045 | 71.6343532 | 0.9995853 | 4.3273E-05 |
| 1.3705 | 71.6406508 | 0.99954202 | 4.3405E-05 |
| 1.37055 | 71.6469506 | 0.99949862 | 4.3538E-05 |
| 1.3706 | 71.6532523 | 0.99945508 | 4.3671E-05 |
| 1.37065 | 71.6595562 | 0.99941141 | 4.3805E-05 |
| 1.3707 | 71.6658622 | 0.9993676 | 4.3939E-05 |
| 1.37075 | 71.6721703 | 0.99932366 | 4.4074E-05 |
| 1.3708 | 71.6784805 | 0.99927959 | 4.4208E-05 |
| 1.37085 | 71.6847927 | 0.99923538 | 4.4344E-05 |
| 1.3709 | 71.6911071 | 0.99919104 | 4.4479E-05 |
| 1.37095 | 71.6974236 | 0.99914656 | 4.4615E-05 |
| 1.371 | 71.7037422 | 0.99910194 | 4.4752E-05 |
| 1.37105 | 71.7100628 | 0.99905719 | 4.4888E-05 |
| 1.3711 | 71.7163856 | 0.9990123 | 4.5026E-05 |
| 1.37115 | 71.7227106 | 0.99896728 | 4.5163E-05 |
| 1.3712 | 71.7290376 | 0.99892212 | 4.5301E-05 |
| 1.37125 | 71.7353667 | 0.99887681 | 4.544E-05 |
| 1.3713 | 71.741698 | 0.99883137 | 4.5578E-05 |
| 1.37135 | 71.7480314 | 0.9987858 | 4.5718E-05 |
| 1.3714 | 71.7543669 | 0.99874008 | 4.5857E-05 |
| 1.37145 | 71.7607045 | 0.99869422 | 4.5997E-05 |
| 1.3715 | 71.7670443 | 0.99864822 | 4.6138E-05 |
| 1.37155 | 71.7733862 | 0.99860209 | 4.6279E-05 |
| 1.3716 | 71.7797302 | 0.99855581 | 4.642E-05 |
| 1.37165 | 71.7860764 | 0.99850939 | 4.6562E-05 |
| 1.3717 | 71.7924246 | 0.99846283 | 4.6704E-05 |
| 1.37175 | 71.7987751 | 0.99841612 | 4.6847E-05 |
| 1.3718 | 71.8051277 | 0.99836927 | 4.6989E-05 |
| 1.37185 | 71.8114824 | 0.99832229 | 4.7133E-05 |
| 1.3719 | 71.8178392 | 0.99827515 | 4.7277E-05 |
| 1.37195 | 71.8241983 | 0.99822788 | 4.7421E-05 |
| 1.372 | 71.8305594 | 0.99818045 | 4.7566E-05 |
| 1.37205 | 71.8369227 | 0.99813289 | 4.7711E-05 |
| 1.3721 | 71.8432882 | 0.99808518 | 4.7856E-05 |
| 1.37215 | 71.8496558 | 0.99803732 | 4.8002E-05 |
| 1.3722 | 71.8560256 | 0.99798932 | 4.8148E-05 |
| 1.37225 | 71.8623976 | 0.99794117 | 4.8295E-05 |
| 1.3723 | 71.8687717 | 0.99789288 | 4.8443E-05 |
| 1.37235 | 71.875148 | 0.99784443 | 4.859E-05 |
| 1.3724 | 71.8815264 | 0.99779584 | 4.8738E-05 |
| 1.37245 | 71.887907 | 0.99774711 | 4.8887E-05 |
| 1.3725 | 71.8942898 | 0.99769822 | 4.9036E-05 |
| 1.37255 | 71.9006748 | 0.99764918 | 4.9185E-05 |
| 1.3726 | 71.907062 | 0.9976 | 4.9335E-05 |
| 1.37265 | 71.9134513 | 0.99755066 | 4.9485E-05 |
| 1.3727 | 71.9198428 | 0.99750118 | 4.9636E-05 |
| 1.37275 | 71.9262365 | 0.99745154 | 4.9787E-05 |
| 1.3728 | 71.9326324 | 0.99740175 | 4.9939E-05 |
| 1.37285 | 71.9390304 | 0.99735182 | 5.0091E-05 |
| 1.3729 | 71.9454307 | 0.99730172 | 5.0243E-05 |
| 1.37295 | 71.9518331 | 0.99725148 | 5.0396E-05 |
| 1.373 | 71.9582378 | 0.99720109 | 5.055E-05 |
| 1.37305 | 71.9646447 | 0.99715054 | 5.0704E-05 |
| 1.3731 | 71.9710537 | 0.99709983 | 5.0858E-05 |
| 1.37315 | 71.977465 | 0.99704897 | 5.1013E-05 |
| 1.3732 | 71.9838784 | 0.99699796 | 5.1168E-05 |
| 1.37325 | 71.9902941 | 0.99694679 | 5.1323E-05 |
| 1.3733 | 71.996712 | 0.99689547 | 5.148E-05 |
| 1.37335 | 72.0031321 | 0.99684399 | 5.1636E-05 |
| 1.3734 | 72.0095544 | 0.99679235 | 5.1793E-05 |
| 1.37345 | 72.0159789 | 0.99674056 | 5.1951E-05 |
| 1.3735 | 72.0224056 | 0.99668861 | 5.2109E-05 |
| 1.37355 | 72.0288346 | 0.9966365 | 5.2267E-05 |
| 1.3736 | 72.0352658 | 0.99658424 | 5.2426E-05 |
| 1.37365 | 72.0416992 | 0.99653181 | 5.2585E-05 |
| 1.3737 | 72.0481349 | 0.99647922 | 5.2745E-05 |
| 1.37375 | 72.0545727 | 0.99642648 | 5.2905E-05 |
| 1.3738 | 72.0610129 | 0.99637357 | 5.3066E-05 |
| 1.37385 | 72.0674552 | 0.99632051 | 5.3227E-05 |
| 1.3739 | 72.0738998 | 0.99626728 | 5.3389E-05 |
| 1.37395 | 72.0803466 | 0.99621389 | 5.3551E-05 |
| 1.374 | 72.0867957 | 0.99616034 | 5.3714E-05 |
| 1.37405 | 72.093247 | 0.99610663 | 5.3877E-05 |
| 1.3741 | 72.0997006 | 0.99605275 | 5.404E-05 |
| 1.37415 | 72.1061564 | 0.99599871 | 5.4204E-05 |
| 1.3742 | 72.1126145 | 0.99594451 | 5.4369E-05 |
| 1.37425 | 72.1190748 | 0.99589014 | 5.4534E-05 |
| 1.3743 | 72.1255374 | 0.9958356 | 5.4699E-05 |
| 1.37435 | 72.1320023 | 0.9957809 | 5.4865E-05 |
| 1.3744 | 72.1384694 | 0.99572604 | 5.5032E-05 |
| 1.37445 | 72.1449388 | 0.99567101 | 5.5199E-05 |
| 1.3745 | 72.1514104 | 0.99561581 | 5.5366E-05 |
| 1.37455 | 72.1578843 | 0.99556044 | 5.5534E-05 |
| 1.3746 | 72.1643605 | 0.99550491 | 5.5703E-05 |
| 1.37465 | 72.170839 | 0.9954492 | 5.5872E-05 |
| 1.3747 | 72.1773198 | 0.99539333 | 5.6041E-05 |
| 1.37475 | 72.1838028 | 0.99533729 | 5.6211E-05 |
| 1.3748 | 72.1902881 | 0.99528108 | 5.6381E-05 |
| 1.37485 | 72.1967757 | 0.9952247 | 5.6552E-05 |
| 1.3749 | 72.2032656 | 0.99516815 | 5.6724E-05 |
| 1.37495 | 72.2097578 | 0.99511142 | 5.6896E-05 |
| 1.375 | 72.2162522 | 0.99505453 | 5.7068E-05 |
| 1.37505 | 72.222749 | 0.99499746 | 5.7241E-05 |
| 1.3751 | 72.2292481 | 0.99494022 | 5.7414E-05 |
| 1.37515 | 72.2357494 | 0.9948828 | 5.7588E-05 |
| 1.3752 | 72.2422531 | 0.99482522 | 5.7763E-05 |
| 1.37525 | 72.2487591 | 0.99476745 | 5.7938E-05 |
| 1.3753 | 72.2552674 | 0.99470952 | 5.8113E-05 |
| 1.37535 | 72.261778 | 0.9946514 | 5.8289E-05 |
| 1.3754 | 72.2682909 | 0.99459311 | 5.8465E-05 |
| 1.37545 | 72.2748061 | 0.99453465 | 5.8642E-05 |
| 1.3755 | 72.2813236 | 0.99447601 | 5.882E-05 |
| 1.37555 | 72.2878435 | 0.99441719 | 5.8998E-05 |
| 1.3756 | 72.2943657 | 0.99435819 | 5.9176E-05 |
| 1.37565 | 72.3008902 | 0.99429901 | 5.9355E-05 |
| 1.3757 | 72.3074171 | 0.99423966 | 5.9535E-05 |
| 1.37575 | 72.3139462 | 0.99418012 | 5.9715E-05 |
| 1.3758 | 72.3204778 | 0.99412041 | 5.9896E-05 |
| 1.37585 | 72.3270116 | 0.99406051 | 6.0077E-05 |
| 1.3759 | 72.3335478 | 0.99400043 | 6.0259E-05 |
| 1.37595 | 72.3400863 | 0.99394018 | 6.0441E-05 |
| 1.376 | 72.3466272 | 0.99387974 | 6.0623E-05 |
| 1.37605 | 72.3531704 | 0.99381911 | 6.0807E-05 |
| 1.3761 | 72.359716 | 0.99375831 | 6.0991E-05 |
| 1.37615 | 72.3662639 | 0.99369731 | 6.1175E-05 |
| 1.3762 | 72.3728142 | 0.99363614 | 6.136E-05 |
| 1.37625 | 72.3793668 | 0.99357478 | 6.1545E-05 |
| 1.3763 | 72.3859218 | 0.99351323 | 6.1731E-05 |
| 1.37635 | 72.3924792 | 0.9934515 | 6.1918E-05 |
| 1.3764 | 72.3990389 | 0.99338959 | 6.2105E-05 |
| 1.37645 | 72.405601 | 0.99332748 | 6.2292E-05 |
| 1.3765 | 72.4121654 | 0.99326519 | 6.248E-05 |
| 1.37655 | 72.4187323 | 0.99320271 | 6.2669E-05 |
| 1.3766 | 72.4253015 | 0.99314004 | 6.2858E-05 |
| 1.37665 | 72.4318731 | 0.99307718 | 6.3048E-05 |
| 1.3767 | 72.4384471 | 0.99301413 | 6.3238E-05 |
| 1.37675 | 72.4450234 | 0.9929509 | 6.3429E-05 |
| 1.3768 | 72.4516022 | 0.99288747 | 6.3621E-05 |
| 1.37685 | 72.4581833 | 0.99282385 | 6.3813E-05 |
| 1.3769 | 72.4647668 | 0.99276003 | 6.4005E-05 |
| 1.37695 | 72.4713527 | 0.99269603 | 6.4198E-05 |
| 1.377 | 72.4779411 | 0.99263183 | 6.4392E-05 |
| 1.37705 | 72.4845318 | 0.99256744 | 6.4586E-05 |
| 1.3771 | 72.4911249 | 0.99250285 | 6.4781E-05 |
| 1.37715 | 72.4977204 | 0.99243807 | 6.4976E-05 |
| 1.3772 | 72.5043184 | 0.99237309 | 6.5172E-05 |
| 1.37725 | 72.5109187 | 0.99230792 | 6.5369E-05 |
| 1.3773 | 72.5175215 | 0.99224255 | 6.5566E-05 |
| 1.37735 | 72.5241267 | 0.99217699 | 6.5763E-05 |
| 1.3774 | 72.5307343 | 0.99211122 | 6.5962E-05 |
| 1.37745 | 72.5373443 | 0.99204526 | 6.616E-05 |
| 1.3775 | 72.5439567 | 0.9919791 | 6.636E-05 |
| 1.37755 | 72.5505716 | 0.99191274 | 6.656E-05 |
| 1.3776 | 72.5571889 | 0.99184618 | 6.676E-05 |
| 1.37765 | 72.5638086 | 0.99177942 | 6.6961E-05 |
| 1.3777 | 72.5704308 | 0.99171246 | 6.7163E-05 |
| 1.37775 | 72.5770554 | 0.9916453 | 6.7365E-05 |
| 1.3778 | 72.5836825 | 0.99157793 | 6.7568E-05 |
| 1.37785 | 72.590312 | 0.99151037 | 6.7771E-05 |
| 1.3779 | 72.5969439 | 0.99144259 | 6.7976E-05 |
| 1.37795 | 72.6035783 | 0.99137462 | 6.818E-05 |
| 1.378 | 72.6102152 | 0.99130644 | 6.8385E-05 |
| 1.37805 | 72.6168545 | 0.99123805 | 6.8591E-05 |
| 1.3781 | 72.6234962 | 0.99116946 | 6.8798E-05 |
| 1.37815 | 72.6301405 | 0.99110066 | 6.9005E-05 |
| 1.3782 | 72.6367871 | 0.99103166 | 6.9212E-05 |
| 1.37825 | 72.6434363 | 0.99096245 | 6.942E-05 |
| 1.3783 | 72.6500879 | 0.99089303 | 6.9629E-05 |
| 1.37835 | 72.656742 | 0.9908234 | 6.9839E-05 |
| 1.3784 | 72.6633986 | 0.99075356 | 7.0049E-05 |
| 1.37845 | 72.6700576 | 0.99068351 | 7.0259E-05 |
| 1.3785 | 72.6767192 | 0.99061325 | 7.047E-05 |
| 1.37855 | 72.6833832 | 0.99054278 | 7.0682E-05 |
| 1.3786 | 72.6900497 | 0.9904721 | 7.0895E-05 |
| 1.37865 | 72.6967187 | 0.9904012 | 7.1108E-05 |
| 1.3787 | 72.7033902 | 0.9903301 | 7.1322E-05 |
| 1.37875 | 72.7100641 | 0.99025877 | 7.1536E-05 |
| 1.3788 | 72.7167406 | 0.99018724 | 7.1751E-05 |
| 1.37885 | 72.7234196 | 0.99011549 | 7.1966E-05 |
| 1.3789 | 72.7301011 | 0.99004352 | 7.2183E-05 |
| 1.37895 | 72.7367851 | 0.98997134 | 7.2399E-05 |
| 1.379 | 72.7434716 | 0.98989894 | 7.2617E-05 |
| 1.37905 | 72.7501606 | 0.98982632 | 7.2835E-05 |
| 1.3791 | 72.7568521 | 0.98975349 | 7.3053E-05 |
| 1.37915 | 72.7635462 | 0.98968043 | 7.3273E-05 |
| 1.3792 | 72.7702427 | 0.98960716 | 7.3493E-05 |
| 1.37925 | 72.7769418 | 0.98953367 | 7.3713E-05 |
| 1.3793 | 72.7836435 | 0.98945996 | 7.3935E-05 |
| 1.37935 | 72.7903476 | 0.98938602 | 7.4156E-05 |
| 1.3794 | 72.7970543 | 0.98931186 | 7.4379E-05 |
| 1.37945 | 72.8037635 | 0.98923749 | 7.4602E-05 |
| 1.3795 | 72.8104753 | 0.98916288 | 7.4826E-05 |
| 1.37955 | 72.8171896 | 0.98908806 | 7.505E-05 |
| 1.3796 | 72.8239065 | 0.98901301 | 7.5275E-05 |
| 1.37965 | 72.8306259 | 0.98893773 | 7.5501E-05 |
| 1.3797 | 72.8373478 | 0.98886223 | 7.5728E-05 |
| 1.37975 | 72.8440723 | 0.9887865 | 7.5955E-05 |
| 1.3798 | 72.8507994 | 0.98871055 | 7.6182E-05 |
| 1.37985 | 72.857529 | 0.98863437 | 7.6411E-05 |
| 1.3799 | 72.8642612 | 0.98855796 | 7.664E-05 |
| 1.37995 | 72.8709959 | 0.98848132 | 7.6869E-05 |
| 1.38 | 72.8777333 | 0.98840445 | 7.71E-05 |
| 1.38005 | 72.8844732 | 0.98832735 | 7.7331E-05 |
| 1.3801 | 72.8912156 | 0.98825002 | 7.7562E-05 |
| 1.38015 | 72.8979607 | 0.98817245 | 7.7795E-05 |
| 1.3802 | 72.9047083 | 0.98809466 | 7.8028E-05 |
| 1.38025 | 72.9114585 | 0.98801663 | 7.8261E-05 |
| 1.3803 | 72.9182113 | 0.98793837 | 7.8496E-05 |
| 1.38035 | 72.9249667 | 0.98785987 | 7.8731E-05 |
| 1.3804 | 72.9317247 | 0.98778114 | 7.8966E-05 |
| 1.38045 | 72.9384853 | 0.98770218 | 7.9203E-05 |
| 1.3805 | 72.9452485 | 0.98762297 | 7.944E-05 |
| 1.38055 | 72.9520143 | 0.98754353 | 7.9678E-05 |
| 1.3806 | 72.9587827 | 0.98746386 | 7.9916E-05 |
| 1.38065 | 72.9655537 | 0.98738394 | 8.0155E-05 |
| 1.3807 | 72.9723273 | 0.98730379 | 8.0395E-05 |
| 1.38075 | 72.9791036 | 0.98722339 | 8.0635E-05 |
| 1.3808 | 72.9858824 | 0.98714276 | 8.0877E-05 |
| 1.38085 | 72.9926639 | 0.98706188 | 8.1119E-05 |
| 1.3809 | 72.999448 | 0.98698076 | 8.1361E-05 |
| 1.38095 | 73.0062347 | 0.9868994 | 8.1604E-05 |
| 1.381 | 73.0130241 | 0.98681779 | 8.1848E-05 |
| 1.38105 | 73.0198161 | 0.98673595 | 8.2093E-05 |
| 1.3811 | 73.0266107 | 0.98665385 | 8.2338E-05 |
| 1.38115 | 73.033408 | 0.98657151 | 8.2585E-05 |
| 1.3812 | 73.0402079 | 0.98648893 | 8.2831E-05 |
| 1.38125 | 73.0470105 | 0.9864061 | 8.3079E-05 |
| 1.3813 | 73.0538157 | 0.98632302 | 8.3327E-05 |
| 1.38135 | 73.0606235 | 0.98623969 | 8.3576E-05 |
| 1.3814 | 73.0674341 | 0.98615612 | 8.3826E-05 |
| 1.38145 | 73.0742473 | 0.98607229 | 8.4076E-05 |
| 1.3815 | 73.0810631 | 0.98598821 | 8.4327E-05 |
| 1.38155 | 73.0878816 | 0.98590389 | 8.4579E-05 |
| 1.3816 | 73.0947028 | 0.98581931 | 8.4832E-05 |
| 1.38165 | 73.1015267 | 0.98573448 | 8.5085E-05 |
| 1.3817 | 73.1083532 | 0.98564939 | 8.5339E-05 |
| 1.38175 | 73.1151824 | 0.98556405 | 8.5593E-05 |
| 1.3818 | 73.1220143 | 0.98547846 | 8.5849E-05 |
| 1.38185 | 73.1288489 | 0.98539261 | 8.6105E-05 |
| 1.3819 | 73.1356862 | 0.98530651 | 8.6362E-05 |
| 1.38195 | 73.1425262 | 0.98522014 | 8.662E-05 |
| 1.382 | 73.1493688 | 0.98513352 | 8.6878E-05 |
| 1.38205 | 73.1562142 | 0.98504665 | 8.7137E-05 |
| 1.3821 | 73.1630622 | 0.98495951 | 8.7397E-05 |
| 1.38215 | 73.169913 | 0.98487211 | 8.7658E-05 |
| 1.3822 | 73.1767665 | 0.98478445 | 8.7919E-05 |
| 1.38225 | 73.1836227 | 0.98469653 | 8.8181E-05 |
| 1.3823 | 73.1904816 | 0.98460835 | 8.8444E-05 |
| 1.38235 | 73.1973432 | 0.98451991 | 8.8708E-05 |
| 1.3824 | 73.2042076 | 0.9844312 | 8.8972E-05 |
| 1.38245 | 73.2110746 | 0.98434223 | 8.9237E-05 |
| 1.3825 | 73.2179444 | 0.98425299 | 8.9503E-05 |
| 1.38255 | 73.224817 | 0.98416349 | 8.977E-05 |
| 1.3826 | 73.2316922 | 0.98407372 | 9.0037E-05 |
| 1.38265 | 73.2385703 | 0.98398368 | 9.0306E-05 |
| 1.3827 | 73.245451 | 0.98389338 | 9.0575E-05 |
| 1.38275 | 73.2523345 | 0.9838028 | 9.0844E-05 |
| 1.3828 | 73.2592207 | 0.98371196 | 9.1115E-05 |
| 1.38285 | 73.2661097 | 0.98362084 | 9.1386E-05 |
| 1.3829 | 73.2730015 | 0.98352946 | 9.1658E-05 |
| 1.38295 | 73.279896 | 0.9834378 | 9.1931E-05 |
| 1.383 | 73.2867933 | 0.98334587 | 9.2205E-05 |
| 1.38305 | 73.2936933 | 0.98325366 | 9.2479E-05 |
| 1.3831 | 73.3005962 | 0.98316118 | 9.2754E-05 |
| 1.38315 | 73.3075017 | 0.98306843 | 9.303E-05 |
| 1.3832 | 73.3144101 | 0.9829754 | 9.3307E-05 |
| 1.38325 | 73.3213213 | 0.98288209 | 9.3585E-05 |
| 1.3833 | 73.3282352 | 0.98278851 | 9.3863E-05 |
| 1.38335 | 73.3351519 | 0.98269464 | 9.4142E-05 |
| 1.3834 | 73.3420714 | 0.9826005 | 9.4422E-05 |
| 1.38345 | 73.3489937 | 0.98250608 | 9.4703E-05 |
| 1.3835 | 73.3559188 | 0.98241138 | 9.4984E-05 |
| 1.38355 | 73.3628467 | 0.98231639 | 9.5267E-05 |
| 1.3836 | 73.3697774 | 0.98222113 | 9.555E-05 |
| 1.38365 | 73.3767109 | 0.98212558 | 9.5834E-05 |
| 1.3837 | 73.3836472 | 0.98202974 | 9.6119E-05 |
| 1.38375 | 73.3905864 | 0.98193362 | 9.6404E-05 |
| 1.3838 | 73.3975283 | 0.98183722 | 9.6691E-05 |
| 1.38385 | 73.4044731 | 0.98174053 | 9.6978E-05 |
| 1.3839 | 73.4114207 | 0.98164355 | 9.7266E-05 |
| 1.38395 | 73.4183711 | 0.98154628 | 9.7555E-05 |
| 1.384 | 73.4253244 | 0.98144873 | 9.7845E-05 |
| 1.38405 | 73.4322805 | 0.98135088 | 9.8135E-05 |
| 1.3841 | 73.4392394 | 0.98125275 | 9.8427E-05 |
| 1.38415 | 73.4462012 | 0.98115432 | 9.8719E-05 |
| 1.3842 | 73.4531659 | 0.9810556 | 9.9012E-05 |
| 1.38425 | 73.4601333 | 0.98095659 | 9.9306E-05 |
| 1.3843 | 73.4671037 | 0.98085728 | 9.9601E-05 |
| 1.38435 | 73.4740769 | 0.98075768 | 9.9896E-05 |
| 1.3844 | 73.4810529 | 0.98065779 | 0.00010019 |
| 1.38445 | 73.4880319 | 0.98055759 | 0.00010049 |
| 1.3845 | 73.4950136 | 0.9804571 | 0.00010079 |
| 1.38455 | 73.5019983 | 0.98035632 | 0.00010109 |
| 1.3846 | 73.5089858 | 0.98025523 | 0.00010139 |
| 1.38465 | 73.5159763 | 0.98015384 | 0.00010169 |
| 1.3847 | 73.5229696 | 0.98005215 | 0.00010199 |
| 1.38475 | 73.5299658 | 0.97995016 | 0.00010229 |
| 1.3848 | 73.5369648 | 0.97984787 | 0.00010259 |
| 1.38485 | 73.5439668 | 0.97974528 | 0.0001029 |
| 1.3849 | 73.5509717 | 0.97964238 | 0.0001032 |
| 1.38495 | 73.5579794 | 0.97953918 | 0.00010351 |
| 1.385 | 73.5649901 | 0.97943567 | 0.00010382 |
| 1.38505 | 73.5720037 | 0.97933185 | 0.00010412 |
| 1.3851 | 73.5790202 | 0.97922773 | 0.00010443 |
| 1.38515 | 73.5860396 | 0.97912329 | 0.00010474 |
| 1.3852 | 73.593062 | 0.97901855 | 0.00010505 |
| 1.38525 | 73.6000872 | 0.9789135 | 0.00010536 |
| 1.3853 | 73.6071154 | 0.97880814 | 0.00010568 |
| 1.38535 | 73.6141466 | 0.97870246 | 0.00010599 |
| 1.3854 | 73.6211806 | 0.97859647 | 0.0001063 |
| 1.38545 | 73.6282176 | 0.97849017 | 0.00010662 |
| 1.3855 | 73.6352575 | 0.97838355 | 0.00010693 |
| 1.38555 | 73.6423004 | 0.97827662 | 0.00010725 |
| 1.3856 | 73.6493463 | 0.97816937 | 0.00010757 |
| 1.38565 | 73.6563951 | 0.97806181 | 0.00010788 |
| 1.3857 | 73.6634468 | 0.97795392 | 0.0001082 |
| 1.38575 | 73.6705015 | 0.97784572 | 0.00010852 |
| 1.3858 | 73.6775592 | 0.97773719 | 0.00010884 |
| 1.38585 | 73.6846199 | 0.97762835 | 0.00010917 |
| 1.3859 | 73.6916835 | 0.97751918 | 0.00010949 |
| 1.38595 | 73.6987501 | 0.97740969 | 0.00010981 |
| 1.386 | 73.7058197 | 0.97729988 | 0.00011014 |
| 1.38605 | 73.7128922 | 0.97718974 | 0.00011046 |
| 1.3861 | 73.7199678 | 0.97707928 | 0.00011079 |
| 1.38615 | 73.7270463 | 0.97696849 | 0.00011112 |
| 1.3862 | 73.7341279 | 0.97685737 | 0.00011145 |
| 1.38625 | 73.7412124 | 0.97674592 | 0.00011178 |
| 1.3863 | 73.7483 | 0.97663415 | 0.00011211 |
| 1.38635 | 73.7553906 | 0.97652204 | 0.00011244 |
| 1.3864 | 73.7624841 | 0.97640961 | 0.00011277 |
| 1.38645 | 73.7695807 | 0.97629684 | 0.0001131 |
| 1.3865 | 73.7766803 | 0.97618374 | 0.00011344 |
| 1.38655 | 73.783783 | 0.9760703 | 0.00011377 |
| 1.3866 | 73.7908886 | 0.97595653 | 0.00011411 |
| 1.38665 | 73.7979973 | 0.97584242 | 0.00011444 |
| 1.3867 | 73.8051091 | 0.97572798 | 0.00011478 |
| 1.38675 | 73.8122239 | 0.9756132 | 0.00011512 |
| 1.3868 | 73.8193417 | 0.97549807 | 0.00011546 |
| 1.38685 | 73.8264625 | 0.97538261 | 0.0001158 |
| 1.3869 | 73.8335865 | 0.97526681 | 0.00011614 |
| 1.38695 | 73.8407134 | 0.97515067 | 0.00011649 |
| 1.387 | 73.8478435 | 0.97503418 | 0.00011683 |
| 1.38705 | 73.8549766 | 0.97491736 | 0.00011717 |
| 1.3871 | 73.8621128 | 0.97480018 | 0.00011752 |
| 1.38715 | 73.869252 | 0.97468266 | 0.00011787 |
| 1.3872 | 73.8763943 | 0.9745648 | 0.00011821 |
| 1.38725 | 73.8835397 | 0.97444658 | 0.00011856 |
| 1.3873 | 73.8906882 | 0.97432802 | 0.00011891 |
| 1.38735 | 73.8978398 | 0.97420911 | 0.00011926 |
| 1.3874 | 73.9049945 | 0.97408985 | 0.00011961 |
| 1.38745 | 73.9121523 | 0.97397023 | 0.00011997 |
| 1.3875 | 73.9193131 | 0.97385027 | 0.00012032 |
| 1.38755 | 73.9264771 | 0.97372995 | 0.00012067 |
| 1.3876 | 73.9336442 | 0.97360927 | 0.00012103 |
| 1.38765 | 73.9408144 | 0.97348824 | 0.00012139 |
| 1.3877 | 73.9479877 | 0.97336686 | 0.00012174 |
| 1.38775 | 73.9551642 | 0.97324511 | 0.0001221 |
| 1.3878 | 73.9623437 | 0.97312301 | 0.00012246 |
| 1.38785 | 73.9695264 | 0.97300055 | 0.00012282 |
| 1.3879 | 73.9767123 | 0.97287772 | 0.00012318 |
| 1.38795 | 73.9839013 | 0.97275454 | 0.00012355 |
| 1.388 | 73.9910934 | 0.97263099 | 0.00012391 |
| 1.38805 | 73.9982887 | 0.97250708 | 0.00012428 |
| 1.3881 | 74.0054871 | 0.97238281 | 0.00012464 |
| 1.38815 | 74.0126887 | 0.97225817 | 0.00012501 |
| 1.3882 | 74.0198934 | 0.97213316 | 0.00012538 |
| 1.38825 | 74.0271013 | 0.97200778 | 0.00012574 |
| 1.3883 | 74.0343124 | 0.97188204 | 0.00012611 |
| 1.38835 | 74.0415266 | 0.97175592 | 0.00012649 |
| 1.3884 | 74.048744 | 0.97162944 | 0.00012686 |
| 1.38845 | 74.0559646 | 0.97150258 | 0.00012723 |
| 1.3885 | 74.0631884 | 0.97137535 | 0.0001276 |
| 1.38855 | 74.0704154 | 0.97124775 | 0.00012798 |
| 1.3886 | 74.0776456 | 0.97111977 | 0.00012836 |
| 1.38865 | 74.084879 | 0.97099141 | 0.00012873 |
| 1.3887 | 74.0921156 | 0.97086268 | 0.00012911 |
| 1.38875 | 74.0993554 | 0.97073356 | 0.00012949 |
| 1.3888 | 74.1065984 | 0.97060407 | 0.00012987 |
| 1.38885 | 74.1138446 | 0.9704742 | 0.00013025 |
| 1.3889 | 74.121094 | 0.97034395 | 0.00013064 |
| 1.38895 | 74.1283467 | 0.97021331 | 0.00013102 |
| 1.389 | 74.1356026 | 0.97008229 | 0.0001314 |
| 1.38905 | 74.1428617 | 0.96995089 | 0.00013179 |
| 1.3891 | 74.1501241 | 0.9698191 | 0.00013218 |
| 1.38915 | 74.1573897 | 0.96968692 | 0.00013257 |
| 1.3892 | 74.1646586 | 0.96955436 | 0.00013295 |
| 1.38925 | 74.1719307 | 0.9694214 | 0.00013334 |
| 1.3893 | 74.1792061 | 0.96928806 | 0.00013374 |
| 1.38935 | 74.1864847 | 0.96915432 | 0.00013413 |
| 1.3894 | 74.1937666 | 0.96902019 | 0.00013452 |
| 1.38945 | 74.2010518 | 0.96888567 | 0.00013492 |
| 1.3895 | 74.2083402 | 0.96875075 | 0.00013531 |
| 1.38955 | 74.215632 | 0.96861544 | 0.00013571 |
| 1.3896 | 74.222927 | 0.96847973 | 0.00013611 |
| 1.38965 | 74.2302253 | 0.96834362 | 0.00013651 |
| 1.3897 | 74.2375269 | 0.96820712 | 0.00013691 |
| 1.38975 | 74.2448318 | 0.96807021 | 0.00013731 |
| 1.3898 | 74.25214 | 0.9679329 | 0.00013771 |
| 1.38985 | 74.2594515 | 0.96779519 | 0.00013812 |
| 1.3899 | 74.2667663 | 0.96765707 | 0.00013852 |
| 1.38995 | 74.2740844 | 0.96751855 | 0.00013893 |
| 1.39 | 74.2814059 | 0.96737963 | 0.00013933 |
| 1.39005 | 74.2887307 | 0.96724029 | 0.00013974 |
| 1.3901 | 74.2960588 | 0.96710055 | 0.00014015 |
| 1.39015 | 74.3033902 | 0.9669604 | 0.00014056 |
| 1.3902 | 74.310725 | 0.96681984 | 0.00014097 |
| 1.39025 | 74.3180631 | 0.96667887 | 0.00014139 |
| 1.3903 | 74.3254046 | 0.96653748 | 0.0001418 |
| 1.39035 | 74.3327494 | 0.96639568 | 0.00014222 |
| 1.3904 | 74.3400976 | 0.96625347 | 0.00014263 |
| 1.39045 | 74.3474492 | 0.96611083 | 0.00014305 |
| 1.3905 | 74.3548041 | 0.96596778 | 0.00014347 |
| 1.39055 | 74.3621624 | 0.96582432 | 0.00014389 |
| 1.3906 | 74.3695241 | 0.96568043 | 0.00014431 |
| 1.39065 | 74.3768891 | 0.96553612 | 0.00014473 |
| 1.3907 | 74.3842575 | 0.96539139 | 0.00014516 |
| 1.39075 | 74.3916294 | 0.96524623 | 0.00014558 |
| 1.3908 | 74.3990046 | 0.96510065 | 0.00014601 |
| 1.39085 | 74.4063832 | 0.96495465 | 0.00014643 |
| 1.3909 | 74.4137653 | 0.96480821 | 0.00014686 |
| 1.39095 | 74.4211507 | 0.96466135 | 0.00014729 |
| 1.391 | 74.4285396 | 0.96451406 | 0.00014772 |
| 1.39105 | 74.4359319 | 0.96436634 | 0.00014815 |
| 1.3911 | 74.4433276 | 0.96421819 | 0.00014859 |
| 1.39115 | 74.4507267 | 0.9640696 | 0.00014902 |
| 1.3912 | 74.4581293 | 0.96392058 | 0.00014946 |
| 1.39125 | 74.4655353 | 0.96377113 | 0.00014989 |
| 1.3913 | 74.4729448 | 0.96362123 | 0.00015033 |
| 1.39135 | 74.4803577 | 0.9634709 | 0.00015077 |
| 1.3914 | 74.4877741 | 0.96332013 | 0.00015121 |
| 1.39145 | 74.4951939 | 0.96316892 | 0.00015165 |
| 1.3915 | 74.5026172 | 0.96301727 | 0.0001521 |
| 1.39155 | 74.510044 | 0.96286517 | 0.00015254 |
| 1.3916 | 74.5174742 | 0.96271263 | 0.00015298 |
| 1.39165 | 74.5249079 | 0.96255965 | 0.00015343 |
| 1.3917 | 74.5323451 | 0.96240622 | 0.00015388 |
| 1.39175 | 74.5397858 | 0.96225234 | 0.00015433 |
| 1.3918 | 74.54723 | 0.96209801 | 0.00015478 |
| 1.39185 | 74.5546777 | 0.96194323 | 0.00015523 |
| 1.3919 | 74.5621289 | 0.961788 | 0.00015568 |
| 1.39195 | 74.5695836 | 0.96163231 | 0.00015614 |
| 1.392 | 74.5770418 | 0.96147618 | 0.00015659 |
| 1.39205 | 74.5845036 | 0.96131958 | 0.00015705 |
| 1.3921 | 74.5919688 | 0.96116253 | 0.00015751 |
| 1.39215 | 74.5994376 | 0.96100502 | 0.00015797 |
| 1.3922 | 74.60691 | 0.96084705 | 0.00015843 |
| 1.39225 | 74.6143859 | 0.96068863 | 0.00015889 |
| 1.3923 | 74.6218653 | 0.96052973 | 0.00015935 |
| 1.39235 | 74.6293483 | 0.96037038 | 0.00015982 |
| 1.3924 | 74.6368348 | 0.96021056 | 0.00016028 |
| 1.39245 | 74.6443249 | 0.96005028 | 0.00016075 |
| 1.3925 | 74.6518186 | 0.95988952 | 0.00016122 |
| 1.39255 | 74.6593158 | 0.9597283 | 0.00016169 |
| 1.3926 | 74.6668166 | 0.95956661 | 0.00016216 |
| 1.39265 | 74.674321 | 0.95940445 | 0.00016263 |
| 1.3927 | 74.681829 | 0.95924182 | 0.00016311 |
| 1.39275 | 74.6893406 | 0.95907871 | 0.00016358 |
| 1.3928 | 74.6968557 | 0.95891513 | 0.00016406 |
| 1.39285 | 74.7043745 | 0.95875107 | 0.00016454 |
| 1.3929 | 74.7118969 | 0.95858653 | 0.00016502 |
| 1.39295 | 74.7194229 | 0.95842152 | 0.0001655 |
| 1.393 | 74.7269525 | 0.95825602 | 0.00016598 |
| 1.39305 | 74.7344858 | 0.95809004 | 0.00016646 |
| 1.3931 | 74.7420227 | 0.95792358 | 0.00016695 |
| 1.39315 | 74.7495632 | 0.95775663 | 0.00016743 |
| 1.3932 | 74.7571074 | 0.9575892 | 0.00016792 |
| 1.39325 | 74.7646552 | 0.95742128 | 0.00016841 |
| 1.3933 | 74.7722066 | 0.95725288 | 0.0001689 |
| 1.39335 | 74.7797617 | 0.95708398 | 0.00016939 |
| 1.3934 | 74.7873205 | 0.95691459 | 0.00016988 |
| 1.39345 | 74.794883 | 0.95674471 | 0.00017038 |
| 1.3935 | 74.8024491 | 0.95657433 | 0.00017087 |
| 1.39355 | 74.8100189 | 0.95640346 | 0.00017137 |
| 1.3936 | 74.8175924 | 0.95623209 | 0.00017187 |
| 1.39365 | 74.8251696 | 0.95606023 | 0.00017237 |
| 1.3937 | 74.8327505 | 0.95588786 | 0.00017287 |
| 1.39375 | 74.840335 | 0.95571499 | 0.00017337 |
| 1.3938 | 74.8479233 | 0.95554162 | 0.00017387 |
| 1.39385 | 74.8555153 | 0.95536775 | 0.00017438 |
| 1.3939 | 74.863111 | 0.95519337 | 0.00017488 |
| 1.39395 | 74.8707105 | 0.95501849 | 0.00017539 |
| 1.394 | 74.8783136 | 0.95484309 | 0.0001759 |
| 1.39405 | 74.8859206 | 0.95466719 | 0.00017641 |
| 1.3941 | 74.8935312 | 0.95449078 | 0.00017693 |
| 1.39415 | 74.9011456 | 0.95431385 | 0.00017744 |
| 1.3942 | 74.9087637 | 0.95413641 | 0.00017795 |
| 1.39425 | 74.9163857 | 0.95395846 | 0.00017847 |
| 1.3943 | 74.9240113 | 0.95377999 | 0.00017899 |
| 1.39435 | 74.9316408 | 0.953601 | 0.00017951 |
| 1.3944 | 74.939274 | 0.95342149 | 0.00018003 |
| 1.39445 | 74.946911 | 0.95324146 | 0.00018055 |
| 1.3945 | 74.9545518 | 0.95306091 | 0.00018108 |
| 1.39455 | 74.9621963 | 0.95287983 | 0.0001816 |
| 1.3946 | 74.9698447 | 0.95269823 | 0.00018213 |
| 1.39465 | 74.9774969 | 0.9525161 | 0.00018266 |
| 1.3947 | 74.9851529 | 0.95233344 | 0.00018319 |
| 1.39475 | 74.9928127 | 0.95215026 | 0.00018372 |
| 1.3948 | 75.0004763 | 0.95196654 | 0.00018425 |
| 1.39485 | 75.0081438 | 0.95178229 | 0.00018478 |
| 1.3949 | 75.015815 | 0.9515975 | 0.00018532 |
| 1.39495 | 75.0234902 | 0.95141218 | 0.00018586 |
| 1.395 | 75.0311691 | 0.95122633 | 0.0001864 |
| 1.39505 | 75.038852 | 0.95103993 | 0.00018694 |
| 1.3951 | 75.0465386 | 0.95085299 | 0.00018748 |
| 1.39515 | 75.0542292 | 0.95066551 | 0.00018802 |
| 1.3952 | 75.0619236 | 0.95047749 | 0.00018857 |
| 1.39525 | 75.0696219 | 0.95028893 | 0.00018911 |
| 1.3953 | 75.0773241 | 0.95009981 | 0.00018966 |
| 1.39535 | 75.0850301 | 0.94991015 | 0.00019021 |
| 1.3954 | 75.0927401 | 0.94971995 | 0.00019076 |
| 1.39545 | 75.1004539 | 0.94952919 | 0.00019131 |
| 1.3955 | 75.1081717 | 0.94933787 | 0.00019187 |
| 1.39555 | 75.1158933 | 0.94914601 | 0.00019242 |
| 1.3956 | 75.1236189 | 0.94895358 | 0.00019298 |
| 1.39565 | 75.1313484 | 0.94876061 | 0.00019354 |
| 1.3957 | 75.1390818 | 0.94856707 | 0.0001941 |
| 1.39575 | 75.1468192 | 0.94837297 | 0.00019466 |
| 1.3958 | 75.1545605 | 0.94817831 | 0.00019522 |
| 1.39585 | 75.1623058 | 0.94798309 | 0.00019579 |
| 1.3959 | 75.170055 | 0.9477873 | 0.00019635 |
| 1.39595 | 75.1778082 | 0.94759095 | 0.00019692 |
| 1.396 | 75.1855653 | 0.94739403 | 0.00019749 |
| 1.39605 | 75.1933264 | 0.94719654 | 0.00019806 |
| 1.3961 | 75.2010915 | 0.94699847 | 0.00019863 |
| 1.39615 | 75.2088606 | 0.94679984 | 0.00019921 |
| 1.3962 | 75.2166337 | 0.94660063 | 0.00019978 |
| 1.39625 | 75.2244107 | 0.94640085 | 0.00020036 |
| 1.3963 | 75.2321918 | 0.94620048 | 0.00020094 |
| 1.39635 | 75.2399769 | 0.94599954 | 0.00020152 |
| 1.3964 | 75.247766 | 0.94579802 | 0.0002021 |
| 1.39645 | 75.2555591 | 0.94559592 | 0.00020269 |
| 1.3965 | 75.2633563 | 0.94539323 | 0.00020327 |
| 1.39655 | 75.2711575 | 0.94518995 | 0.00020386 |
| 1.3966 | 75.2789627 | 0.94498609 | 0.00020445 |
| 1.39665 | 75.286772 | 0.94478164 | 0.00020504 |
| 1.3967 | 75.2945853 | 0.9445766 | 0.00020563 |
| 1.39675 | 75.3024027 | 0.94437097 | 0.00020623 |
| 1.3968 | 75.3102242 | 0.94416475 | 0.00020682 |
| 1.39685 | 75.3180497 | 0.94395792 | 0.00020742 |
| 1.3969 | 75.3258794 | 0.94375051 | 0.00020802 |
| 1.39695 | 75.3337131 | 0.94354249 | 0.00020862 |
| 1.397 | 75.3415509 | 0.94333387 | 0.00020922 |
| 1.39705 | 75.3493928 | 0.94312465 | 0.00020982 |
| 1.3971 | 75.3572388 | 0.94291483 | 0.00021043 |
| 1.39715 | 75.3650889 | 0.9427044 | 0.00021104 |
| 1.3972 | 75.3729432 | 0.94249337 | 0.00021164 |
| 1.39725 | 75.3808016 | 0.94228172 | 0.00021225 |
| 1.3973 | 75.3886641 | 0.94206947 | 0.00021287 |
| 1.39735 | 75.3965307 | 0.9418566 | 0.00021348 |
| 1.3974 | 75.4044015 | 0.94164312 | 0.0002141 |
| 1.39745 | 75.4122765 | 0.94142903 | 0.00021471 |
| 1.3975 | 75.4201556 | 0.94121431 | 0.00021533 |
| 1.39755 | 75.4280389 | 0.94099898 | 0.00021595 |
| 1.3976 | 75.4359263 | 0.94078303 | 0.00021657 |
| 1.39765 | 75.443818 | 0.94056645 | 0.0002172 |
| 1.3977 | 75.4517138 | 0.94034926 | 0.00021782 |
| 1.39775 | 75.4596138 | 0.94013143 | 0.00021845 |
| 1.3978 | 75.4675181 | 0.93991298 | 0.00021908 |
| 1.39785 | 75.4754265 | 0.9396939 | 0.00021971 |
| 1.3979 | 75.4833391 | 0.93947418 | 0.00022035 |
| 1.39795 | 75.491256 | 0.93925384 | 0.00022098 |
| 1.398 | 75.4991771 | 0.93903286 | 0.00022162 |
| 1.39805 | 75.5071025 | 0.93881124 | 0.00022225 |
| 1.3981 | 75.515032 | 0.93858899 | 0.00022289 |
| 1.39815 | 75.5229659 | 0.9383661 | 0.00022354 |
| 1.3982 | 75.530904 | 0.93814256 | 0.00022418 |
| 1.39825 | 75.5388463 | 0.93791838 | 0.00022482 |
| 1.3983 | 75.5467929 | 0.93769356 | 0.00022547 |
| 1.39835 | 75.5547438 | 0.93746809 | 0.00022612 |
| 1.3984 | 75.562699 | 0.93724197 | 0.00022677 |
| 1.39845 | 75.5706585 | 0.9370152 | 0.00022742 |
| 1.3985 | 75.5786223 | 0.93678778 | 0.00022808 |
| 1.39855 | 75.5865904 | 0.9365597 | 0.00022873 |
| 1.3986 | 75.5945628 | 0.93633097 | 0.00022939 |
| 1.39865 | 75.6025395 | 0.93610158 | 0.00023005 |
| 1.3987 | 75.6105206 | 0.93587153 | 0.00023071 |
| 1.39875 | 75.618506 | 0.93564082 | 0.00023137 |
| 1.3988 | 75.6264957 | 0.93540944 | 0.00023204 |
| 1.39885 | 75.6344898 | 0.9351774 | 0.00023271 |
| 1.3989 | 75.6424882 | 0.9349447 | 0.00023337 |
| 1.39895 | 75.650491 | 0.93471132 | 0.00023405 |
| 1.399 | 75.6584982 | 0.93447728 | 0.00023472 |
| 1.39905 | 75.6665097 | 0.93424256 | 0.00023539 |
| 1.3991 | 75.6745257 | 0.93400717 | 0.00023607 |
| 1.39915 | 75.682546 | 0.9337711 | 0.00023675 |
| 1.3992 | 75.6905707 | 0.93353436 | 0.00023743 |
| 1.39925 | 75.6985999 | 0.93329693 | 0.00023811 |
| 1.3993 | 75.7066334 | 0.93305882 | 0.00023879 |
| 1.39935 | 75.7146714 | 0.93282003 | 0.00023948 |
| 1.3994 | 75.7227138 | 0.93258055 | 0.00024016 |
| 1.39945 | 75.7307607 | 0.93234039 | 0.00024085 |
| 1.3995 | 75.738812 | 0.93209954 | 0.00024154 |
| 1.39955 | 75.7468677 | 0.93185799 | 0.00024224 |
| 1.3996 | 75.754928 | 0.93161576 | 0.00024293 |
| 1.39965 | 75.7629926 | 0.93137282 | 0.00024363 |
| 1.3997 | 75.7710618 | 0.93112919 | 0.00024433 |
| 1.39975 | 75.7791354 | 0.93088486 | 0.00024503 |
| 1.3998 | 75.7872136 | 0.93063984 | 0.00024573 |
| 1.39985 | 75.7952962 | 0.9303941 | 0.00024644 |
| 1.3999 | 75.8033834 | 0.93014767 | 0.00024714 |
| 1.39995 | 75.811475 | 0.92990052 | 0.00024785 |
| 1.4 | 75.8195712 | 0.92965267 | 0.00024856 |
| 1.40005 | 75.8276719 | 0.92940411 | 0.00024927 |
| 1.4001 | 75.8357772 | 0.92915483 | 0.00024999 |
| 1.40015 | 75.8438869 | 0.92890485 | 0.00025071 |
| 1.4002 | 75.8520013 | 0.92865414 | 0.00025142 |
| 1.40025 | 75.8601202 | 0.92840272 | 0.00025214 |
| 1.4003 | 75.8682437 | 0.92815057 | 0.00025287 |
| 1.40035 | 75.8763717 | 0.9278977 | 0.00025359 |
| 1.4004 | 75.8845044 | 0.92764411 | 0.00025432 |
| 1.40045 | 75.8926416 | 0.9273898 | 0.00025505 |
| 1.4005 | 75.9007834 | 0.92713475 | 0.00025578 |
| 1.40055 | 75.9089299 | 0.92687897 | 0.00025651 |
| 1.4006 | 75.9170809 | 0.92662247 | 0.00025724 |
| 1.40065 | 75.9252366 | 0.92636522 | 0.00025798 |
| 1.4007 | 75.9333969 | 0.92610724 | 0.00025872 |
| 1.40075 | 75.9415619 | 0.92584853 | 0.00025946 |
| 1.4008 | 75.9497315 | 0.92558907 | 0.0002602 |
| 1.40085 | 75.9579058 | 0.92532887 | 0.00026094 |
| 1.4009 | 75.9660847 | 0.92506792 | 0.00026169 |
| 1.40095 | 75.9742683 | 0.92480623 | 0.00026244 |
| 1.401 | 75.9824566 | 0.92454379 | 0.00026319 |
| 1.40105 | 75.9906496 | 0.9242806 | 0.00026394 |
| 1.4011 | 75.9988472 | 0.92401666 | 0.0002647 |
| 1.40115 | 76.0070496 | 0.92375196 | 0.00026546 |
| 1.4012 | 76.0152567 | 0.92348651 | 0.00026621 |
| 1.40125 | 76.0234685 | 0.92322029 | 0.00026698 |
| 1.4013 | 76.0316851 | 0.92295332 | 0.00026774 |
| 1.40135 | 76.0399064 | 0.92268558 | 0.0002685 |
| 1.4014 | 76.0481324 | 0.92241708 | 0.00026927 |
| 1.40145 | 76.0563632 | 0.9221478 | 0.00027004 |
| 1.4015 | 76.0645988 | 0.92187776 | 0.00027081 |
| 1.40155 | 76.0728391 | 0.92160695 | 0.00027159 |
| 1.4016 | 76.0810842 | 0.92133537 | 0.00027236 |
| 1.40165 | 76.0893341 | 0.92106301 | 0.00027314 |
| 1.4017 | 76.0975888 | 0.92078987 | 0.00027392 |
| 1.40175 | 76.1058483 | 0.92051595 | 0.0002747 |
| 1.4018 | 76.1141127 | 0.92024125 | 0.00027549 |
| 1.40185 | 76.1223818 | 0.91996576 | 0.00027627 |
| 1.4019 | 76.1306558 | 0.91968949 | 0.00027706 |
| 1.40195 | 76.1389346 | 0.91941243 | 0.00027785 |
| 1.402 | 76.1472183 | 0.91913457 | 0.00027864 |
| 1.40205 | 76.1555068 | 0.91885593 | 0.00027944 |
| 1.4021 | 76.1638002 | 0.91857649 | 0.00028024 |
| 1.40215 | 76.1720985 | 0.91829625 | 0.00028104 |
| 1.4022 | 76.1804017 | 0.91801521 | 0.00028184 |
| 1.40225 | 76.1887098 | 0.91773338 | 0.00028264 |
| 1.4023 | 76.1970227 | 0.91745073 | 0.00028345 |
| 1.40235 | 76.2053406 | 0.91716728 | 0.00028426 |
| 1.4024 | 76.2136634 | 0.91688303 | 0.00028507 |
| 1.40245 | 76.2219911 | 0.91659796 | 0.00028588 |
| 1.4025 | 76.2303238 | 0.91631208 | 0.0002867 |
| 1.40255 | 76.2386614 | 0.91602538 | 0.00028751 |
| 1.4026 | 76.247004 | 0.91573787 | 0.00028833 |
| 1.40265 | 76.2553515 | 0.91544954 | 0.00028915 |
| 1.4027 | 76.2637041 | 0.91516038 | 0.00028998 |
| 1.40275 | 76.2720616 | 0.9148704 | 0.0002908 |
| 1.4028 | 76.2804241 | 0.9145796 | 0.00029163 |
| 1.40285 | 76.2887915 | 0.91428797 | 0.00029246 |
| 1.4029 | 76.2971641 | 0.9139955 | 0.0002933 |
| 1.40295 | 76.3055416 | 0.91370221 | 0.00029413 |
| 1.403 | 76.3139241 | 0.91340807 | 0.00029497 |
| 1.40305 | 76.3223117 | 0.9131131 | 0.00029581 |
| 1.4031 | 76.3307044 | 0.9128173 | 0.00029665 |
| 1.40315 | 76.3391021 | 0.91252064 | 0.0002975 |
| 1.4032 | 76.3475049 | 0.91222315 | 0.00029834 |
| 1.40325 | 76.3559127 | 0.9119248 | 0.00029919 |
| 1.4033 | 76.3643256 | 0.91162561 | 0.00030004 |
| 1.40335 | 76.3727437 | 0.91132557 | 0.0003009 |
| 1.4034 | 76.3811668 | 0.91102467 | 0.00030175 |
| 1.40345 | 76.389595 | 0.91072291 | 0.00030261 |
| 1.4035 | 76.3980284 | 0.9104203 | 0.00030347 |
| 1.40355 | 76.4064669 | 0.91011683 | 0.00030434 |
| 1.4036 | 76.4149106 | 0.90981249 | 0.0003052 |
| 1.40365 | 76.4233594 | 0.90950729 | 0.00030607 |
| 1.4037 | 76.4318133 | 0.90920122 | 0.00030694 |
| 1.40375 | 76.4402725 | 0.90889428 | 0.00030781 |
| 1.4038 | 76.4487368 | 0.90858647 | 0.00030869 |
| 1.40385 | 76.4572063 | 0.90827778 | 0.00030957 |
| 1.4039 | 76.465681 | 0.90796821 | 0.00031045 |
| 1.40395 | 76.4741609 | 0.90765777 | 0.00031133 |
| 1.404 | 76.482646 | 0.90734644 | 0.00031221 |
| 1.40405 | 76.4911364 | 0.90703423 | 0.0003131 |
| 1.4041 | 76.499632 | 0.90672113 | 0.00031399 |
| 1.40415 | 76.5081329 | 0.90640714 | 0.00031488 |
| 1.4042 | 76.516639 | 0.90609226 | 0.00031578 |
| 1.40425 | 76.5251504 | 0.90577649 | 0.00031667 |
| 1.4043 | 76.533667 | 0.90545981 | 0.00031757 |
| 1.40435 | 76.542189 | 0.90514224 | 0.00031847 |
| 1.4044 | 76.5507163 | 0.90482377 | 0.00031938 |
| 1.40445 | 76.5592488 | 0.90450439 | 0.00032028 |
| 1.4045 | 76.5677867 | 0.90418411 | 0.00032119 |
| 1.40455 | 76.5763299 | 0.90386292 | 0.0003221 |
| 1.4046 | 76.5848785 | 0.90354081 | 0.00032302 |
| 1.40465 | 76.5934324 | 0.90321779 | 0.00032394 |
| 1.4047 | 76.6019917 | 0.90289386 | 0.00032485 |
| 1.40475 | 76.6105563 | 0.902569 | 0.00032578 |
| 1.4048 | 76.6191264 | 0.90224323 | 0.0003267 |
| 1.40485 | 76.6277018 | 0.90191653 | 0.00032763 |
| 1.4049 | 76.6362826 | 0.9015889 | 0.00032856 |
| 1.40495 | 76.6448688 | 0.90126035 | 0.00032949 |
| 1.405 | 76.6534605 | 0.90093086 | 0.00033042 |
| 1.40505 | 76.6620576 | 0.90060044 | 0.00033136 |
| 1.4051 | 76.6706601 | 0.90026908 | 0.0003323 |
| 1.40515 | 76.6792681 | 0.89993678 | 0.00033324 |
| 1.4052 | 76.6878815 | 0.89960354 | 0.00033418 |
| 1.40525 | 76.6965005 | 0.89926936 | 0.00033513 |
| 1.4053 | 76.7051249 | 0.89893423 | 0.00033608 |
| 1.40535 | 76.7137548 | 0.89859815 | 0.00033703 |
| 1.4054 | 76.7223902 | 0.89826112 | 0.00033799 |
| 1.40545 | 76.7310312 | 0.89792313 | 0.00033894 |
| 1.4055 | 76.7396776 | 0.89758418 | 0.0003399 |
| 1.40555 | 76.7483297 | 0.89724428 | 0.00034087 |
| 1.4056 | 76.7569872 | 0.89690341 | 0.00034183 |
| 1.40565 | 76.7656503 | 0.89656158 | 0.0003428 |
| 1.4057 | 76.774319 | 0.89621878 | 0.00034377 |
| 1.40575 | 76.7829933 | 0.89587501 | 0.00034474 |
| 1.4058 | 76.7916732 | 0.89553026 | 0.00034572 |
| 1.40585 | 76.8003587 | 0.89518454 | 0.0003467 |
| 1.4059 | 76.8090498 | 0.89483785 | 0.00034768 |
| 1.40595 | 76.8177465 | 0.89449017 | 0.00034866 |
| 1.406 | 76.8264489 | 0.8941415 | 0.00034965 |
| 1.40605 | 76.8351569 | 0.89379186 | 0.00035064 |
| 1.4061 | 76.8438706 | 0.89344122 | 0.00035163 |
| 1.40615 | 76.85259 | 0.89308959 | 0.00035262 |
| 1.4062 | 76.861315 | 0.89273696 | 0.00035362 |
| 1.40625 | 76.8700457 | 0.89238334 | 0.00035462 |
| 1.4063 | 76.8787822 | 0.89202872 | 0.00035562 |
| 1.40635 | 76.8875243 | 0.89167309 | 0.00035663 |
| 1.4064 | 76.8962722 | 0.89131646 | 0.00035764 |
| 1.40645 | 76.9050259 | 0.89095883 | 0.00035865 |
| 1.4065 | 76.9137852 | 0.89060018 | 0.00035966 |
| 1.40655 | 76.9225504 | 0.89024052 | 0.00036068 |
| 1.4066 | 76.9313213 | 0.88987984 | 0.0003617 |
| 1.40665 | 76.940098 | 0.88951814 | 0.00036272 |
| 1.4067 | 76.9488806 | 0.88915542 | 0.00036374 |
| 1.40675 | 76.9576689 | 0.88879168 | 0.00036477 |
| 1.4068 | 76.966463 | 0.88842691 | 0.0003658 |
| 1.40685 | 76.975263 | 0.8880611 | 0.00036683 |
| 1.4069 | 76.9840688 | 0.88769427 | 0.00036787 |
| 1.40695 | 76.9928805 | 0.8873264 | 0.00036891 |
| 1.407 | 77.0016981 | 0.88695749 | 0.00036995 |
| 1.40705 | 77.0105215 | 0.88658754 | 0.00037099 |
| 1.4071 | 77.0193509 | 0.88621655 | 0.00037204 |
| 1.40715 | 77.0281861 | 0.8858445 | 0.00037309 |
| 1.4072 | 77.0370273 | 0.88547141 | 0.00037414 |
| 1.40725 | 77.0458744 | 0.88509727 | 0.0003752 |
| 1.4073 | 77.0547274 | 0.88472207 | 0.00037626 |
| 1.40735 | 77.0635864 | 0.88434581 | 0.00037732 |
| 1.4074 | 77.0724514 | 0.88396849 | 0.00037838 |
| 1.40745 | 77.0813223 | 0.88359011 | 0.00037945 |
| 1.4075 | 77.0901992 | 0.88321065 | 0.00038052 |
| 1.40755 | 77.0990822 | 0.88283013 | 0.00038159 |
| 1.4076 | 77.1079711 | 0.88244854 | 0.00038267 |
| 1.40765 | 77.1168661 | 0.88206587 | 0.00038375 |
| 1.4077 | 77.1257671 | 0.88168212 | 0.00038483 |
| 1.40775 | 77.1346742 | 0.88129729 | 0.00038591 |
| 1.4078 | 77.1435873 | 0.88091138 | 0.000387 |
| 1.40785 | 77.1525065 | 0.88052437 | 0.00038809 |
| 1.4079 | 77.1614319 | 0.88013628 | 0.00038919 |
| 1.40795 | 77.1703633 | 0.87974709 | 0.00039028 |
| 1.408 | 77.1793008 | 0.87935681 | 0.00039138 |
| 1.40805 | 77.1882445 | 0.87896543 | 0.00039248 |
| 1.4081 | 77.1971943 | 0.87857295 | 0.00039359 |
| 1.40815 | 77.2061503 | 0.87817936 | 0.0003947 |
| 1.4082 | 77.2151124 | 0.87778466 | 0.00039581 |
| 1.40825 | 77.2240807 | 0.87738885 | 0.00039692 |
| 1.4083 | 77.2330552 | 0.87699193 | 0.00039804 |
| 1.40835 | 77.2420359 | 0.87659389 | 0.00039916 |
| 1.4084 | 77.2510229 | 0.87619472 | 0.00040028 |
| 1.40845 | 77.2600161 | 0.87579444 | 0.00040141 |
| 1.4085 | 77.2690155 | 0.87539303 | 0.00040254 |
| 1.40855 | 77.2780212 | 0.87499049 | 0.00040367 |
| 1.4086 | 77.2870331 | 0.87458682 | 0.00040481 |
| 1.40865 | 77.2960514 | 0.87418201 | 0.00040595 |
| 1.4087 | 77.3050759 | 0.87377606 | 0.00040709 |
| 1.40875 | 77.3141068 | 0.87336897 | 0.00040823 |
| 1.4088 | 77.323144 | 0.87296074 | 0.00040938 |
| 1.40885 | 77.3321875 | 0.87255136 | 0.00041053 |
| 1.4089 | 77.3412374 | 0.87214083 | 0.00041169 |
| 1.40895 | 77.3502936 | 0.87172914 | 0.00041284 |
| 1.409 | 77.3593563 | 0.87131629 | 0.000414 |
| 1.40905 | 77.3684253 | 0.87090229 | 0.00041517 |
| 1.4091 | 77.3775008 | 0.87048712 | 0.00041633 |
| 1.40915 | 77.3865826 | 0.87007079 | 0.0004175 |
| 1.4092 | 77.3956709 | 0.86965328 | 0.00041868 |
| 1.40925 | 77.4047657 | 0.86923461 | 0.00041985 |
| 1.4093 | 77.4138669 | 0.86881476 | 0.00042103 |
| 1.40935 | 77.4229746 | 0.86839372 | 0.00042221 |
| 1.4094 | 77.4320888 | 0.86797151 | 0.0004234 |
| 1.40945 | 77.4412095 | 0.86754811 | 0.00042459 |
| 1.4095 | 77.4503367 | 0.86712352 | 0.00042578 |
| 1.40955 | 77.4594705 | 0.86669774 | 0.00042698 |
| 1.4096 | 77.4686108 | 0.86627076 | 0.00042818 |
| 1.40965 | 77.4777577 | 0.86584259 | 0.00042938 |
| 1.4097 | 77.4869111 | 0.86541321 | 0.00043058 |
| 1.40975 | 77.4960712 | 0.86498263 | 0.00043179 |
| 1.4098 | 77.5052379 | 0.86455084 | 0.000433 |
| 1.40985 | 77.5144111 | 0.86411783 | 0.00043422 |
| 1.4099 | 77.5235911 | 0.86368362 | 0.00043543 |
| 1.40995 | 77.5327776 | 0.86324818 | 0.00043666 |
| 1.41 | 77.5419709 | 0.86281153 | 0.00043788 |
| 1.41005 | 77.5511708 | 0.86237365 | 0.00043911 |
| 1.4101 | 77.5603774 | 0.86193454 | 0.00044034 |
| 1.41015 | 77.5695907 | 0.8614942 | 0.00044157 |
| 1.4102 | 77.5788108 | 0.86105263 | 0.00044281 |
| 1.41025 | 77.5880376 | 0.86060982 | 0.00044405 |
| 1.4103 | 77.5972711 | 0.86016576 | 0.0004453 |
| 1.41035 | 77.6065115 | 0.85972047 | 0.00044655 |
| 1.4104 | 77.6157586 | 0.85927392 | 0.0004478 |
| 1.41045 | 77.6250125 | 0.85882612 | 0.00044905 |
| 1.4105 | 77.6342732 | 0.85837707 | 0.00045031 |
| 1.41055 | 77.6435408 | 0.85792676 | 0.00045157 |
| 1.4106 | 77.6528152 | 0.85747519 | 0.00045284 |
| 1.41065 | 77.6620964 | 0.85702236 | 0.0004541 |
| 1.4107 | 77.6713846 | 0.85656825 | 0.00045538 |
| 1.41075 | 77.6806796 | 0.85611288 | 0.00045665 |
| 1.4108 | 77.6899816 | 0.85565623 | 0.00045793 |
| 1.41085 | 77.6992905 | 0.8551983 | 0.00045921 |
| 1.4109 | 77.7086063 | 0.85473909 | 0.0004605 |
| 1.41095 | 77.7179291 | 0.85427859 | 0.00046178 |
| 1.411 | 77.7272588 | 0.85381681 | 0.00046308 |
| 1.41105 | 77.7365956 | 0.85335373 | 0.00046437 |
| 1.4111 | 77.7459393 | 0.85288936 | 0.00046567 |
| 1.41115 | 77.7552901 | 0.85242369 | 0.00046697 |
| 1.4112 | 77.7646479 | 0.85195671 | 0.00046828 |
| 1.41125 | 77.7740127 | 0.85148843 | 0.00046959 |
| 1.4113 | 77.7833847 | 0.85101884 | 0.0004709 |
| 1.41135 | 77.7927637 | 0.85054794 | 0.00047222 |
| 1.4114 | 77.8021498 | 0.85007572 | 0.00047354 |
| 1.41145 | 77.811543 | 0.84960218 | 0.00047486 |
| 1.4115 | 77.8209434 | 0.84912732 | 0.00047619 |
| 1.41155 | 77.8303509 | 0.84865112 | 0.00047752 |
| 1.4116 | 77.8397656 | 0.8481736 | 0.00047886 |
| 1.41165 | 77.8491875 | 0.84769474 | 0.0004802 |
| 1.4117 | 77.8586165 | 0.84721455 | 0.00048154 |
| 1.41175 | 77.8680528 | 0.84673301 | 0.00048288 |
| 1.4118 | 77.8774964 | 0.84625013 | 0.00048423 |
| 1.41185 | 77.8869471 | 0.8457659 | 0.00048558 |
| 1.4119 | 77.8964052 | 0.84528031 | 0.00048694 |
| 1.41195 | 77.9058705 | 0.84479337 | 0.0004883 |
| 1.412 | 77.9153432 | 0.84430507 | 0.00048966 |
| 1.41205 | 77.9248231 | 0.84381541 | 0.00049103 |
| 1.4121 | 77.9343104 | 0.84332438 | 0.0004924 |
| 1.41215 | 77.9438051 | 0.84283198 | 0.00049378 |
| 1.4122 | 77.9533071 | 0.8423382 | 0.00049515 |
| 1.41225 | 77.9628165 | 0.84184305 | 0.00049654 |
| 1.4123 | 77.9723333 | 0.84134651 | 0.00049792 |
| 1.41235 | 77.9818576 | 0.84084859 | 0.00049931 |
| 1.4124 | 77.9913893 | 0.84034928 | 0.0005007 |
| 1.41245 | 78.0009284 | 0.83984858 | 0.0005021 |
| 1.4125 | 78.010475 | 0.83934648 | 0.0005035 |
| 1.41255 | 78.0200291 | 0.83884297 | 0.00050491 |
| 1.4126 | 78.0295908 | 0.83833807 | 0.00050631 |
| 1.41265 | 78.03916 | 0.83783176 | 0.00050772 |
| 1.4127 | 78.0487367 | 0.83732403 | 0.00050914 |
| 1.41275 | 78.058321 | 0.83681489 | 0.00051056 |
| 1.4128 | 78.0679128 | 0.83630433 | 0.00051198 |
| 1.41285 | 78.0775123 | 0.83579235 | 0.00051341 |
| 1.4129 | 78.0871194 | 0.83527894 | 0.00051484 |
| 1.41295 | 78.0967342 | 0.8347641 | 0.00051628 |
| 1.413 | 78.1063566 | 0.83424782 | 0.00051771 |
| 1.41305 | 78.1159866 | 0.83373011 | 0.00051916 |
| 1.4131 | 78.1256244 | 0.83321095 | 0.0005206 |
| 1.41315 | 78.1352699 | 0.83269035 | 0.00052205 |
| 1.4132 | 78.1449231 | 0.8321683 | 0.00052351 |
| 1.41325 | 78.1545841 | 0.83164479 | 0.00052496 |
| 1.4133 | 78.1642529 | 0.83111983 | 0.00052643 |
| 1.41335 | 78.1739294 | 0.8305934 | 0.00052789 |
| 1.4134 | 78.1836138 | 0.83006551 | 0.00052936 |
| 1.41345 | 78.193306 | 0.82953615 | 0.00053083 |
| 1.4135 | 78.203006 | 0.82900532 | 0.00053231 |
| 1.41355 | 78.2127139 | 0.82847301 | 0.00053379 |
| 1.4136 | 78.2224297 | 0.82793921 | 0.00053528 |
| 1.41365 | 78.2321535 | 0.82740393 | 0.00053677 |
| 1.4137 | 78.2418851 | 0.82686717 | 0.00053826 |
| 1.41375 | 78.2516247 | 0.82632891 | 0.00053976 |
| 1.4138 | 78.2613722 | 0.82578915 | 0.00054126 |
| 1.41385 | 78.2711278 | 0.82524789 | 0.00054276 |
| 1.4139 | 78.2808913 | 0.82470512 | 0.00054427 |
| 1.41395 | 78.2906629 | 0.82416085 | 0.00054579 |
| 1.414 | 78.3004425 | 0.82361506 | 0.0005473 |
| 1.41405 | 78.3102302 | 0.82306776 | 0.00054883 |
| 1.4141 | 78.320026 | 0.82251893 | 0.00055035 |
| 1.41415 | 78.3298298 | 0.82196858 | 0.00055188 |
| 1.4142 | 78.3396419 | 0.8214167 | 0.00055341 |
| 1.41425 | 78.349462 | 0.82086329 | 0.00055495 |
| 1.4143 | 78.3592903 | 0.82030833 | 0.00055649 |
| 1.41435 | 78.3691269 | 0.81975184 | 0.00055804 |
| 1.4144 | 78.3789716 | 0.8191938 | 0.00055959 |
| 1.41445 | 78.3888246 | 0.81863421 | 0.00056114 |
| 1.4145 | 78.3986858 | 0.81807307 | 0.0005627 |
| 1.41455 | 78.4085553 | 0.81751036 | 0.00056427 |
| 1.4146 | 78.4184331 | 0.8169461 | 0.00056583 |
| 1.41465 | 78.4283192 | 0.81638027 | 0.0005674 |
| 1.4147 | 78.4382136 | 0.81581286 | 0.00056898 |
| 1.41475 | 78.4481164 | 0.81524389 | 0.00057056 |
| 1.4148 | 78.4580276 | 0.81467333 | 0.00057214 |
| 1.41485 | 78.4679472 | 0.81410119 | 0.00057373 |
| 1.4149 | 78.4778752 | 0.81352746 | 0.00057532 |
| 1.41495 | 78.4878116 | 0.81295214 | 0.00057692 |
| 1.415 | 78.4977566 | 0.81237522 | 0.00057852 |
| 1.41505 | 78.50771 | 0.81179671 | 0.00058012 |
| 1.4151 | 78.5176719 | 0.81121659 | 0.00058173 |
| 1.41515 | 78.5276423 | 0.81063486 | 0.00058334 |
| 1.4152 | 78.5376214 | 0.81005151 | 0.00058496 |
| 1.41525 | 78.547609 | 0.80946655 | 0.00058658 |
| 1.4153 | 78.5576051 | 0.80887997 | 0.00058821 |
| 1.41535 | 78.56761 | 0.80829176 | 0.00058984 |
| 1.4154 | 78.5776234 | 0.80770192 | 0.00059147 |
| 1.41545 | 78.5876456 | 0.80711044 | 0.00059311 |
| 1.4155 | 78.5976764 | 0.80651733 | 0.00059476 |
| 1.41555 | 78.6077159 | 0.80592257 | 0.00059641 |
| 1.4156 | 78.6177642 | 0.80532617 | 0.00059806 |
| 1.41565 | 78.6278213 | 0.80472811 | 0.00059971 |
| 1.4157 | 78.6378871 | 0.8041284 | 0.00060138 |
| 1.41575 | 78.6479617 | 0.80352702 | 0.00060304 |
| 1.4158 | 78.6580452 | 0.80292398 | 0.00060471 |
| 1.41585 | 78.6681375 | 0.80231927 | 0.00060639 |
| 1.4159 | 78.6782387 | 0.80171289 | 0.00060806 |
| 1.41595 | 78.6883488 | 0.80110482 | 0.00060975 |
| 1.416 | 78.6984678 | 0.80049507 | 0.00061144 |
| 1.41605 | 78.7085958 | 0.79988364 | 0.00061313 |
| 1.4161 | 78.7187327 | 0.79927051 | 0.00061482 |
| 1.41615 | 78.7288787 | 0.79865569 | 0.00061653 |
| 1.4162 | 78.7390337 | 0.79803916 | 0.00061823 |
| 1.41625 | 78.7491977 | 0.79742093 | 0.00061994 |
| 1.4163 | 78.7593708 | 0.79680099 | 0.00062166 |
| 1.41635 | 78.769553 | 0.79617933 | 0.00062338 |
| 1.4164 | 78.7797443 | 0.79555595 | 0.0006251 |
| 1.41645 | 78.7899447 | 0.79493085 | 0.00062683 |
| 1.4165 | 78.8001544 | 0.79430403 | 0.00062856 |
| 1.41655 | 78.8103732 | 0.79367546 | 0.0006303 |
| 1.4166 | 78.8206012 | 0.79304516 | 0.00063204 |
| 1.41665 | 78.8308385 | 0.79241312 | 0.00063379 |
| 1.4167 | 78.8410851 | 0.79177933 | 0.00063554 |
| 1.41675 | 78.851341 | 0.79114379 | 0.0006373 |
| 1.4168 | 78.8616062 | 0.79050649 | 0.00063906 |
| 1.41685 | 78.8718807 | 0.78986744 | 0.00064082 |
| 1.4169 | 78.8821646 | 0.78922661 | 0.00064259 |
| 1.41695 | 78.8924579 | 0.78858402 | 0.00064437 |
| 1.417 | 78.9027607 | 0.78793965 | 0.00064615 |
| 1.41705 | 78.9130729 | 0.7872935 | 0.00064793 |
| 1.4171 | 78.9233945 | 0.78664556 | 0.00064972 |
| 1.41715 | 78.9337257 | 0.78599584 | 0.00065152 |
| 1.4172 | 78.9440664 | 0.78534432 | 0.00065332 |
| 1.41725 | 78.9544167 | 0.78469101 | 0.00065512 |
| 1.4173 | 78.9647766 | 0.78403589 | 0.00065693 |
| 1.41735 | 78.975146 | 0.78337896 | 0.00065874 |
| 1.4174 | 78.9855252 | 0.78272022 | 0.00066056 |
| 1.41745 | 78.995914 | 0.78205966 | 0.00066238 |
| 1.4175 | 79.0063124 | 0.78139728 | 0.00066421 |
| 1.41755 | 79.0167206 | 0.78073307 | 0.00066604 |
| 1.4176 | 79.0271386 | 0.78006703 | 0.00066788 |
| 1.41765 | 79.0375663 | 0.77939915 | 0.00066972 |
| 1.4177 | 79.0480039 | 0.77872943 | 0.00067157 |
| 1.41775 | 79.0584513 | 0.77805787 | 0.00067342 |
| 1.4178 | 79.0689085 | 0.77738445 | 0.00067528 |
| 1.41785 | 79.0793756 | 0.77670917 | 0.00067714 |
| 1.4179 | 79.0898527 | 0.77603203 | 0.000679 |
| 1.41795 | 79.1003397 | 0.77535303 | 0.00068088 |
| 1.418 | 79.1108367 | 0.77467215 | 0.00068275 |
| 1.41805 | 79.1213437 | 0.7739894 | 0.00068463 |
| 1.4181 | 79.1318607 | 0.77330477 | 0.00068652 |
| 1.41815 | 79.1423878 | 0.77261825 | 0.00068841 |
| 1.4182 | 79.152925 | 0.77192983 | 0.00069031 |
| 1.41825 | 79.1634723 | 0.77123953 | 0.00069221 |
| 1.4183 | 79.1740297 | 0.77054732 | 0.00069412 |
| 1.41835 | 79.1845974 | 0.7698532 | 0.00069603 |
| 1.4184 | 79.1951752 | 0.76915717 | 0.00069794 |
| 1.41845 | 79.2057633 | 0.76845923 | 0.00069987 |
| 1.4185 | 79.2163617 | 0.76775936 | 0.00070179 |
| 1.41855 | 79.2269703 | 0.76705757 | 0.00070372 |
| 1.4186 | 79.2375893 | 0.76635384 | 0.00070566 |
| 1.41865 | 79.2482187 | 0.76564818 | 0.0007076 |
| 1.4187 | 79.2588585 | 0.76494058 | 0.00070955 |
| 1.41875 | 79.2695087 | 0.76423103 | 0.0007115 |
| 1.4188 | 79.2801693 | 0.76351952 | 0.00071346 |
| 1.41885 | 79.2908404 | 0.76280606 | 0.00071542 |
| 1.4189 | 79.3015221 | 0.76209064 | 0.00071739 |
| 1.41895 | 79.3122143 | 0.76137325 | 0.00071936 |
| 1.419 | 79.3229171 | 0.76065388 | 0.00072134 |
| 1.41905 | 79.3336305 | 0.75993254 | 0.00072333 |
| 1.4191 | 79.3443545 | 0.75920921 | 0.00072532 |
| 1.41915 | 79.3550893 | 0.7584839 | 0.00072731 |
| 1.4192 | 79.3658347 | 0.75775659 | 0.00072931 |
| 1.41925 | 79.3765909 | 0.75702728 | 0.00073131 |
| 1.4193 | 79.3873578 | 0.75629597 | 0.00073332 |
| 1.41935 | 79.3981356 | 0.75556264 | 0.00073534 |
| 1.4194 | 79.4089242 | 0.75482731 | 0.00073736 |
| 1.41945 | 79.4197237 | 0.75408995 | 0.00073938 |
| 1.4195 | 79.4305341 | 0.75335056 | 0.00074142 |
| 1.41955 | 79.4413554 | 0.75260915 | 0.00074345 |
| 1.4196 | 79.4521877 | 0.7518657 | 0.00074549 |
| 1.41965 | 79.4630311 | 0.7511202 | 0.00074754 |
| 1.4197 | 79.4738854 | 0.75037266 | 0.00074959 |
| 1.41975 | 79.4847509 | 0.74962307 | 0.00075165 |
| 1.4198 | 79.4956274 | 0.74887142 | 0.00075372 |
| 1.41985 | 79.5065151 | 0.7481177 | 0.00075578 |
| 1.4199 | 79.517414 | 0.74736192 | 0.00075786 |
| 1.41995 | 79.5283241 | 0.74660406 | 0.00075994 |
| 1.42 | 79.5392455 | 0.74584412 | 0.00076202 |
| 1.42005 | 79.5501781 | 0.74508209 | 0.00076411 |
| 1.4201 | 79.5611221 | 0.74431798 | 0.00076621 |
| 1.42015 | 79.5720774 | 0.74355177 | 0.00076831 |
| 1.4202 | 79.5830441 | 0.74278346 | 0.00077042 |
| 1.42025 | 79.5940222 | 0.74201304 | 0.00077253 |
| 1.4203 | 79.6050118 | 0.7412405 | 0.00077465 |
| 1.42035 | 79.616013 | 0.74046585 | 0.00077678 |
| 1.4204 | 79.6270256 | 0.73968907 | 0.00077891 |
| 1.42045 | 79.6380498 | 0.73891017 | 0.00078104 |
| 1.4205 | 79.6490857 | 0.73812913 | 0.00078318 |
| 1.42055 | 79.6601332 | 0.73734595 | 0.00078533 |
| 1.4206 | 79.6711923 | 0.73656062 | 0.00078748 |
| 1.42065 | 79.6822632 | 0.73577314 | 0.00078964 |
| 1.4207 | 79.6933459 | 0.7349835 | 0.0007918 |
| 1.42075 | 79.7044404 | 0.7341917 | 0.00079397 |
| 1.4208 | 79.7155467 | 0.73339773 | 0.00079615 |
| 1.42085 | 79.7266649 | 0.73260158 | 0.00079833 |
| 1.4209 | 79.7377949 | 0.73180325 | 0.00080051 |
| 1.42095 | 79.748937 | 0.73100274 | 0.00080271 |
| 1.421 | 79.760091 | 0.73020003 | 0.0008049 |
| 1.42105 | 79.7712571 | 0.72939513 | 0.00080711 |
| 1.4211 | 79.7824353 | 0.72858802 | 0.00080932 |
| 1.42115 | 79.7936255 | 0.72777871 | 0.00081153 |
| 1.4212 | 79.804828 | 0.72696717 | 0.00081375 |
| 1.42125 | 79.8160426 | 0.72615342 | 0.00081598 |
| 1.4213 | 79.8272694 | 0.72533744 | 0.00081821 |
| 1.42135 | 79.8385085 | 0.72451923 | 0.00082045 |
| 1.4214 | 79.84976 | 0.72369878 | 0.0008227 |
| 1.42145 | 79.8610237 | 0.72287608 | 0.00082495 |
| 1.4215 | 79.8722999 | 0.72205113 | 0.0008272 |
| 1.42155 | 79.8835885 | 0.72122393 | 0.00082947 |
| 1.4216 | 79.8948896 | 0.72039446 | 0.00083173 |
| 1.42165 | 79.9062033 | 0.71956273 | 0.00083401 |
| 1.4217 | 79.9175294 | 0.71872872 | 0.00083629 |
| 1.42175 | 79.9288682 | 0.71789243 | 0.00083857 |
| 1.4218 | 79.9402197 | 0.71705386 | 0.00084087 |
| 1.42185 | 79.9515838 | 0.71621299 | 0.00084316 |
| 1.4219 | 79.9629606 | 0.71536983 | 0.00084547 |
| 1.42195 | 79.9743503 | 0.71452436 | 0.00084778 |
| 1.422 | 79.9857527 | 0.71367658 | 0.0008501 |
| 1.42205 | 79.9971681 | 0.71282649 | 0.00085242 |
| 1.4221 | 80.0085963 | 0.71197407 | 0.00085475 |
| 1.42215 | 80.0200375 | 0.71111932 | 0.00085708 |
| 1.4222 | 80.0314917 | 0.71026224 | 0.00085942 |
| 1.42225 | 80.0429589 | 0.70940282 | 0.00086177 |
| 1.4223 | 80.0544392 | 0.70854105 | 0.00086412 |
| 1.42235 | 80.0659327 | 0.70767693 | 0.00086648 |
| 1.4224 | 80.0774393 | 0.70681045 | 0.00086885 |
| 1.42245 | 80.0889592 | 0.7059416 | 0.00087122 |
| 1.4225 | 80.1004923 | 0.70507039 | 0.0008736 |
| 1.42255 | 80.1120387 | 0.70419679 | 0.00087598 |
| 1.4226 | 80.1235985 | 0.70332081 | 0.00087837 |
| 1.42265 | 80.1351718 | 0.70244244 | 0.00088077 |
| 1.4227 | 80.1467584 | 0.70156168 | 0.00088317 |
| 1.42275 | 80.1583586 | 0.70067851 | 0.00088558 |
| 1.4228 | 80.1699724 | 0.69979293 | 0.00088799 |
| 1.42285 | 80.1815997 | 0.69890494 | 0.00089042 |
| 1.4229 | 80.1932407 | 0.69801452 | 0.00089284 |
| 1.42295 | 80.2048954 | 0.69712168 | 0.00089528 |
| 1.423 | 80.2165638 | 0.6962264 | 0.00089772 |
| 1.42305 | 80.2282461 | 0.69532868 | 0.00090017 |
| 1.4231 | 80.2399421 | 0.69442852 | 0.00090262 |
| 1.42315 | 80.2516521 | 0.6935259 | 0.00090508 |
| 1.4232 | 80.263376 | 0.69262082 | 0.00090755 |
| 1.42325 | 80.275114 | 0.69171327 | 0.00091002 |
| 1.4233 | 80.2868659 | 0.69080325 | 0.0009125 |
| 1.42335 | 80.298632 | 0.68989076 | 0.00091498 |
| 1.4234 | 80.3104122 | 0.68897577 | 0.00091748 |
| 1.42345 | 80.3222067 | 0.6880583 | 0.00091998 |
| 1.4235 | 80.3340153 | 0.68713832 | 0.00092248 |
| 1.42355 | 80.3458383 | 0.68621584 | 0.00092499 |
| 1.4236 | 80.3576757 | 0.68529085 | 0.00092751 |
| 1.42365 | 80.3695274 | 0.68436334 | 0.00093004 |
| 1.4237 | 80.3813937 | 0.6834333 | 0.00093257 |
| 1.42375 | 80.3932744 | 0.68250073 | 0.00093511 |
| 1.4238 | 80.4051697 | 0.68156563 | 0.00093765 |
| 1.42385 | 80.4170796 | 0.68062798 | 0.0009402 |
| 1.4239 | 80.4290043 | 0.67968777 | 0.00094276 |
| 1.42395 | 80.4409436 | 0.67874501 | 0.00094533 |
| 1.424 | 80.4528978 | 0.67779969 | 0.0009479 |
| 1.42405 | 80.4648668 | 0.67685179 | 0.00095048 |
| 1.4241 | 80.4768507 | 0.67590131 | 0.00095306 |
| 1.42415 | 80.4888495 | 0.67494825 | 0.00095565 |
| 1.4242 | 80.5008634 | 0.6739926 | 0.00095825 |
| 1.42425 | 80.5128924 | 0.67303435 | 0.00096086 |
| 1.4243 | 80.5249365 | 0.67207349 | 0.00096347 |
| 1.42435 | 80.5369957 | 0.67111002 | 0.00096609 |
| 1.4244 | 80.5490703 | 0.67014393 | 0.00096871 |
| 1.42445 | 80.5611601 | 0.66917522 | 0.00097135 |
| 1.4245 | 80.5732653 | 0.66820387 | 0.00097399 |
| 1.42455 | 80.5853859 | 0.66722988 | 0.00097663 |
| 1.4246 | 80.597522 | 0.66625325 | 0.00097929 |
| 1.42465 | 80.6096736 | 0.66527396 | 0.00098195 |
| 1.4247 | 80.6218409 | 0.66429202 | 0.00098461 |
| 1.42475 | 80.6340238 | 0.6633074 | 0.00098729 |
| 1.4248 | 80.6462224 | 0.66232011 | 0.00098997 |
| 1.42485 | 80.6584369 | 0.66133014 | 0.00099266 |
| 1.4249 | 80.6706671 | 0.66033748 | 0.00099535 |
| 1.42495 | 80.6829133 | 0.65934213 | 0.00099806 |
| 1.425 | 80.6951755 | 0.65834408 | 0.00100076 |
| 1.42505 | 80.7074537 | 0.65734331 | 0.00100348 |
| 1.4251 | 80.719748 | 0.65633983 | 0.0010062 |
| 1.42515 | 80.7320584 | 0.65533363 | 0.00100893 |
| 1.4252 | 80.7443851 | 0.65432469 | 0.00101167 |
| 1.42525 | 80.7567281 | 0.65331302 | 0.00101442 |
| 1.4253 | 80.7690875 | 0.6522986 | 0.00101717 |
| 1.42535 | 80.7814633 | 0.65128143 | 0.00101993 |
| 1.4254 | 80.7938555 | 0.65026151 | 0.00102269 |
| 1.42545 | 80.8062644 | 0.64923881 | 0.00102547 |
| 1.4255 | 80.8186898 | 0.64821334 | 0.00102825 |
| 1.42555 | 80.831132 | 0.6471851 | 0.00103104 |
| 1.4256 | 80.8435909 | 0.64615406 | 0.00103383 |
| 1.42565 | 80.8560666 | 0.64512023 | 0.00103663 |
| 1.4257 | 80.8685593 | 0.64408359 | 0.00103944 |
| 1.42575 | 80.8810689 | 0.64304415 | 0.00104226 |
| 1.4258 | 80.8935956 | 0.64200189 | 0.00104508 |
| 1.42585 | 80.9061393 | 0.64095681 | 0.00104792 |
| 1.4259 | 80.9187003 | 0.63990889 | 0.00105076 |
| 1.42595 | 80.9312785 | 0.63885813 | 0.0010536 |
| 1.426 | 80.943874 | 0.63780453 | 0.00105646 |
| 1.42605 | 80.9564869 | 0.63674808 | 0.00105932 |
| 1.4261 | 80.9691173 | 0.63568876 | 0.00106219 |
| 1.42615 | 80.9817652 | 0.63462657 | 0.00106506 |
| 1.4262 | 80.9944307 | 0.63356151 | 0.00106795 |
| 1.42625 | 81.007114 | 0.63249356 | 0.00107084 |
| 1.4263 | 81.019815 | 0.63142273 | 0.00107374 |
| 1.42635 | 81.0325338 | 0.63034899 | 0.00107664 |
| 1.4264 | 81.0452706 | 0.62927235 | 0.00107956 |
| 1.42645 | 81.0580253 | 0.62819279 | 0.00108248 |
| 1.4265 | 81.0707981 | 0.62711032 | 0.00108541 |
| 1.42655 | 81.0835891 | 0.62602491 | 0.00108834 |
| 1.4266 | 81.0963983 | 0.62493657 | 0.00109129 |
| 1.42665 | 81.1092258 | 0.62384528 | 0.00109424 |
| 1.4267 | 81.1220717 | 0.62275104 | 0.0010972 |
| 1.42675 | 81.134936 | 0.62165385 | 0.00110016 |
| 1.4268 | 81.1478189 | 0.62055368 | 0.00110314 |
| 1.42685 | 81.1607205 | 0.61945054 | 0.00110612 |
| 1.4269 | 81.1736407 | 0.61834442 | 0.00110911 |
| 1.42695 | 81.1865797 | 0.61723531 | 0.00111211 |
| 1.427 | 81.1995376 | 0.6161232 | 0.00111512 |
| 1.42705 | 81.2125145 | 0.61500808 | 0.00111813 |
| 1.4271 | 81.2255104 | 0.61388995 | 0.00112115 |
| 1.42715 | 81.2385254 | 0.6127688 | 0.00112418 |
| 1.4272 | 81.2515596 | 0.61164462 | 0.00112722 |
| 1.42725 | 81.2646131 | 0.6105174 | 0.00113026 |
| 1.4273 | 81.2776861 | 0.60938714 | 0.00113332 |
| 1.42735 | 81.2907784 | 0.60825382 | 0.00113638 |
| 1.4274 | 81.3038904 | 0.60711744 | 0.00113945 |
| 1.42745 | 81.317022 | 0.605978 | 0.00114252 |
| 1.4275 | 81.3301733 | 0.60483547 | 0.00114561 |
| 1.42755 | 81.3433444 | 0.60368987 | 0.0011487 |
| 1.4276 | 81.3565355 | 0.60254117 | 0.0011518 |
| 1.42765 | 81.3697466 | 0.60138937 | 0.00115491 |
| 1.4277 | 81.3829778 | 0.60023446 | 0.00115803 |
| 1.42775 | 81.3962291 | 0.59907643 | 0.00116115 |
| 1.4278 | 81.4095008 | 0.59791528 | 0.00116428 |
| 1.42785 | 81.4227928 | 0.596751 | 0.00116742 |
| 1.4279 | 81.4361054 | 0.59558357 | 0.00117057 |
| 1.42795 | 81.4494384 | 0.594413 | 0.00117373 |
| 1.428 | 81.4627922 | 0.59323927 | 0.0011769 |
| 1.42805 | 81.4761667 | 0.59206237 | 0.00118007 |
| 1.4281 | 81.4895621 | 0.5908823 | 0.00118325 |
| 1.42815 | 81.5029784 | 0.58969905 | 0.00118644 |
| 1.4282 | 81.5164159 | 0.5885126 | 0.00118964 |
| 1.42825 | 81.5298744 | 0.58732296 | 0.00119285 |
| 1.4283 | 81.5433543 | 0.58613011 | 0.00119606 |
| 1.42835 | 81.5568554 | 0.58493405 | 0.00119929 |
| 1.4284 | 81.5703781 | 0.58373476 | 0.00120252 |
| 1.42845 | 81.5839223 | 0.58253225 | 0.00120576 |
| 1.4285 | 81.5974882 | 0.58132649 | 0.00120901 |
| 1.42855 | 81.6110759 | 0.58011748 | 0.00121226 |
| 1.4286 | 81.6246855 | 0.57890522 | 0.00121553 |
| 1.42865 | 81.6383171 | 0.57768969 | 0.0012188 |
| 1.4287 | 81.6519707 | 0.57647089 | 0.00122208 |
| 1.42875 | 81.6656466 | 0.5752488 | 0.00122538 |
| 1.4288 | 81.6793448 | 0.57402343 | 0.00122867 |
| 1.42885 | 81.6930654 | 0.57279475 | 0.00123198 |
| 1.4289 | 81.7068086 | 0.57156277 | 0.0012353 |
| 1.42895 | 81.7205744 | 0.57032747 | 0.00123862 |
| 1.429 | 81.734363 | 0.56908885 | 0.00124196 |
| 1.42905 | 81.7481745 | 0.56784689 | 0.0012453 |
| 1.4291 | 81.7620089 | 0.56660159 | 0.00124865 |
| 1.42915 | 81.7758665 | 0.56535294 | 0.00125201 |
| 1.4292 | 81.7897473 | 0.56410094 | 0.00125538 |
| 1.42925 | 81.8036514 | 0.56284556 | 0.00125875 |
| 1.4293 | 81.817579 | 0.56158681 | 0.00126214 |
| 1.42935 | 81.8315302 | 0.56032467 | 0.00126553 |
| 1.4294 | 81.8455051 | 0.55905914 | 0.00126894 |
| 1.42945 | 81.8595039 | 0.5577902 | 0.00127235 |
| 1.4295 | 81.8735265 | 0.55651786 | 0.00127577 |
| 1.42955 | 81.8875733 | 0.55524209 | 0.0012792 |
| 1.4296 | 81.9016442 | 0.55396289 | 0.00128263 |
| 1.42965 | 81.9157395 | 0.55268026 | 0.00128608 |
| 1.4297 | 81.9298593 | 0.55139418 | 0.00128954 |
| 1.42975 | 81.9440036 | 0.55010464 | 0.001293 |
| 1.4298 | 81.9581726 | 0.54881164 | 0.00129648 |
| 1.42985 | 81.9723665 | 0.54751516 | 0.00129996 |
| 1.4299 | 81.9865854 | 0.5462152 | 0.00130345 |
| 1.42995 | 82.0008293 | 0.54491175 | 0.00130695 |
| 1.43 | 82.0150985 | 0.5436048 | 0.00131046 |
| 1.43005 | 82.0293931 | 0.54229434 | 0.00131398 |
| 1.4301 | 82.0437132 | 0.54098036 | 0.00131751 |
| 1.43015 | 82.058059 | 0.53966286 | 0.00132104 |
| 1.4302 | 82.0724305 | 0.53834181 | 0.00132459 |
| 1.43025 | 82.086828 | 0.53701722 | 0.00132814 |
| 1.4303 | 82.1012516 | 0.53568908 | 0.00133171 |
| 1.43035 | 82.1157014 | 0.53435737 | 0.00133528 |
| 1.4304 | 82.1301775 | 0.53302209 | 0.00133887 |
| 1.43045 | 82.1446802 | 0.53168322 | 0.00134246 |
| 1.4305 | 82.1592095 | 0.53034076 | 0.00134606 |
| 1.43055 | 82.1737656 | 0.5289947 | 0.00134967 |
| 1.4306 | 82.1883486 | 0.52764504 | 0.00135329 |
| 1.43065 | 82.2029588 | 0.52629175 | 0.00135692 |
| 1.4307 | 82.2175962 | 0.52493483 | 0.00136056 |
| 1.43075 | 82.2322611 | 0.52357427 | 0.0013642 |
| 1.4308 | 82.2469535 | 0.52221007 | 0.00136786 |
| 1.43085 | 82.2616736 | 0.52084221 | 0.00137153 |
| 1.4309 | 82.2764217 | 0.51947068 | 0.0013752 |
| 1.43095 | 82.2911977 | 0.51809548 | 0.00137889 |
| 1.431 | 82.306002 | 0.51671659 | 0.00138258 |
| 1.43105 | 82.3208346 | 0.51533401 | 0.00138629 |
| 1.4311 | 82.3356958 | 0.51394772 | 0.00139 |
| 1.43115 | 82.3505857 | 0.51255772 | 0.00139373 |
| 1.4312 | 82.3655044 | 0.51116399 | 0.00139746 |
| 1.43125 | 82.3804522 | 0.50976654 | 0.0014012 |
| 1.4313 | 82.3954291 | 0.50836533 | 0.00140495 |
| 1.43135 | 82.4104355 | 0.50696038 | 0.00140871 |
| 1.4314 | 82.4254714 | 0.50555167 | 0.00141249 |
| 1.43145 | 82.4405371 | 0.50413918 | 0.00141627 |
| 1.4315 | 82.4556326 | 0.50272291 | 0.00142006 |
| 1.43155 | 82.4707583 | 0.50130285 | 0.00142386 |
| 1.4316 | 82.4859142 | 0.499879 | 0.00142767 |
| 1.43165 | 82.5011006 | 0.49845133 | 0.00143149 |
| 1.4317 | 82.5163176 | 0.49701984 | 0.00143532 |
| 1.43175 | 82.5315654 | 0.49558452 | 0.00143916 |
| 1.4318 | 82.5468443 | 0.49414536 | 0.00144301 |
| 1.43185 | 82.5621544 | 0.49270235 | 0.00144687 |
| 1.4319 | 82.5774958 | 0.49125548 | 0.00145074 |
| 1.43195 | 82.5928689 | 0.48980474 | 0.00145462 |
| 1.432 | 82.6082738 | 0.48835012 | 0.00145851 |
| 1.43205 | 82.6237106 | 0.48689161 | 0.00146241 |
| 1.4321 | 82.6391797 | 0.4854292 | 0.00146632 |
| 1.43215 | 82.6546811 | 0.48396289 | 0.00147024 |
| 1.4322 | 82.6702152 | 0.48249265 | 0.00147417 |
| 1.43225 | 82.685782 | 0.48101849 | 0.00147811 |
| 1.4323 | 82.7013819 | 0.47954038 | 0.00148205 |
| 1.43235 | 82.717015 | 0.47805833 | 0.00148601 |
| 1.4324 | 82.7326816 | 0.47657231 | 0.00148998 |
| 1.43245 | 82.7483818 | 0.47508233 | 0.00149396 |
| 1.4325 | 82.7641159 | 0.47358836 | 0.00149795 |
| 1.43255 | 82.7798841 | 0.47209041 | 0.00150196 |
| 1.4326 | 82.7956867 | 0.47058845 | 0.00150597 |
| 1.43265 | 82.8115238 | 0.46908249 | 0.00150999 |
| 1.4327 | 82.8273956 | 0.4675725 | 0.00151402 |
| 1.43275 | 82.8433025 | 0.46605848 | 0.00151806 |
| 1.4328 | 82.8592447 | 0.46454042 | 0.00152211 |
| 1.43285 | 82.8752223 | 0.46301831 | 0.00152618 |
| 1.4329 | 82.8912357 | 0.46149213 | 0.00153025 |
| 1.43295 | 82.907285 | 0.45996188 | 0.00153433 |
| 1.433 | 82.9233705 | 0.45842755 | 0.00153842 |
| 1.43305 | 82.9394925 | 0.45688913 | 0.00154253 |
| 1.4331 | 82.9556512 | 0.4553466 | 0.00154664 |
| 1.43315 | 82.9718469 | 0.45379996 | 0.00155077 |
| 1.4332 | 82.9880798 | 0.45224919 | 0.0015549 |
| 1.43325 | 83.0043502 | 0.45069428 | 0.00155905 |
| 1.4333 | 83.0206583 | 0.44913523 | 0.00156321 |
| 1.43335 | 83.0370044 | 0.44757202 | 0.00156738 |
| 1.4334 | 83.0533888 | 0.44600465 | 0.00157155 |
| 1.43345 | 83.0698117 | 0.44443309 | 0.00157574 |
| 1.4335 | 83.0862735 | 0.44285735 | 0.00157994 |
| 1.43355 | 83.1027743 | 0.4412774 | 0.00158415 |
| 1.4336 | 83.1193145 | 0.43969325 | 0.00158837 |
| 1.43365 | 83.1358944 | 0.43810488 | 0.00159261 |
| 1.4337 | 83.1525143 | 0.43651227 | 0.00159685 |
| 1.43375 | 83.1691744 | 0.43491542 | 0.0016011 |
| 1.4338 | 83.185875 | 0.43331432 | 0.00160537 |
| 1.43385 | 83.2026165 | 0.43170895 | 0.00160964 |
| 1.4339 | 83.2193991 | 0.43009931 | 0.00161393 |
| 1.43395 | 83.2362231 | 0.42848538 | 0.00161823 |
| 1.434 | 83.253089 | 0.42686715 | 0.00162254 |
| 1.43405 | 83.2699968 | 0.42524461 | 0.00162685 |
| 1.4341 | 83.2869471 | 0.42361776 | 0.00163118 |
| 1.43415 | 83.3039401 | 0.42198657 | 0.00163553 |
| 1.4342 | 83.3209761 | 0.42035105 | 0.00163988 |
| 1.43425 | 83.3380555 | 0.41871117 | 0.00164424 |
| 1.4343 | 83.3551786 | 0.41706693 | 0.00164862 |
| 1.43435 | 83.3723457 | 0.41541831 | 0.001653 |
| 1.4344 | 83.3895572 | 0.41376531 | 0.0016574 |
| 1.43445 | 83.4068135 | 0.41210791 | 0.00166181 |
| 1.4345 | 83.4241148 | 0.4104461 | 0.00166623 |
| 1.43455 | 83.4414615 | 0.40877987 | 0.00167066 |
| 1.4346 | 83.4588541 | 0.40710921 | 0.0016751 |
| 1.43465 | 83.4762928 | 0.40543411 | 0.00167955 |
| 1.4347 | 83.4937781 | 0.40375455 | 0.00168402 |
| 1.43475 | 83.5113103 | 0.40207053 | 0.0016885 |
| 1.4348 | 83.5288898 | 0.40038204 | 0.00169298 |
| 1.43485 | 83.5465169 | 0.39868906 | 0.00169748 |
| 1.4349 | 83.5641922 | 0.39699157 | 0.00170199 |
| 1.43495 | 83.5819159 | 0.39528958 | 0.00170652 |
| 1.435 | 83.5996884 | 0.39358306 | 0.00171105 |
| 1.43505 | 83.6175103 | 0.39187202 | 0.00171559 |
| 1.4351 | 83.6353819 | 0.39015642 | 0.00172015 |
| 1.43515 | 83.6533035 | 0.38843627 | 0.00172472 |
| 1.4352 | 83.6712758 | 0.38671155 | 0.0017293 |
| 1.43525 | 83.689299 | 0.38498225 | 0.00173389 |
| 1.4353 | 83.7073736 | 0.38324836 | 0.00173849 |
| 1.43535 | 83.7255 | 0.38150987 | 0.00174311 |
| 1.4354 | 83.7436788 | 0.37976676 | 0.00174774 |
| 1.43545 | 83.7619103 | 0.37801902 | 0.00175237 |
| 1.4355 | 83.7801951 | 0.37626665 | 0.00175703 |
| 1.43555 | 83.7985335 | 0.37450962 | 0.00176169 |
| 1.4356 | 83.8169262 | 0.37274793 | 0.00176636 |
| 1.43565 | 83.8353735 | 0.37098157 | 0.00177105 |
| 1.4357 | 83.8538759 | 0.36921052 | 0.00177575 |
| 1.43575 | 83.872434 | 0.36743478 | 0.00178046 |
| 1.4358 | 83.8910483 | 0.36565432 | 0.00178518 |
| 1.43585 | 83.9097192 | 0.36386915 | 0.00178991 |
| 1.4359 | 83.9284474 | 0.36207923 | 0.00179466 |
| 1.43595 | 83.9472332 | 0.36028458 | 0.00179942 |
| 1.436 | 83.9660774 | 0.35848516 | 0.00180419 |
| 1.43605 | 83.9849803 | 0.35668098 | 0.00180897 |
| 1.4361 | 84.0039427 | 0.35487201 | 0.00181376 |
| 1.43615 | 84.0229649 | 0.35305824 | 0.00181857 |
| 1.4362 | 84.0420477 | 0.35123968 | 0.00182339 |
| 1.43625 | 84.0611916 | 0.34941629 | 0.00182822 |
| 1.4363 | 84.0803971 | 0.34758807 | 0.00183306 |
| 1.43635 | 84.099665 | 0.34575501 | 0.00183792 |
| 1.4364 | 84.1189957 | 0.34391709 | 0.00184279 |
| 1.43645 | 84.13839 | 0.3420743 | 0.00184767 |
| 1.4365 | 84.1578485 | 0.34022663 | 0.00185256 |
| 1.43655 | 84.1773717 | 0.33837408 | 0.00185746 |
| 1.4366 | 84.1969604 | 0.33651661 | 0.00186238 |
| 1.43665 | 84.2166152 | 0.33465423 | 0.00186731 |
| 1.4367 | 84.2363368 | 0.33278692 | 0.00187226 |
| 1.43675 | 84.2561259 | 0.33091466 | 0.00187721 |
| 1.4368 | 84.2759832 | 0.32903745 | 0.00188218 |
| 1.43685 | 84.2959093 | 0.32715527 | 0.00188716 |
| 1.4369 | 84.3159051 | 0.32526811 | 0.00189215 |
| 1.43695 | 84.3359713 | 0.32337596 | 0.00189716 |
| 1.437 | 84.3561086 | 0.3214788 | 0.00190218 |
| 1.43705 | 84.3763177 | 0.31957663 | 0.00190721 |
| 1.4371 | 84.3965995 | 0.31766942 | 0.00191225 |
| 1.43715 | 84.4169547 | 0.31575717 | 0.00191731 |
| 1.4372 | 84.4373842 | 0.31383986 | 0.00192238 |
| 1.43725 | 84.4578887 | 0.31191748 | 0.00192746 |
| 1.4373 | 84.4784692 | 0.30999002 | 0.00193256 |
| 1.43735 | 84.4991264 | 0.30805746 | 0.00193766 |
| 1.4374 | 84.5198612 | 0.3061198 | 0.00194279 |
| 1.43745 | 84.5406746 | 0.30417701 | 0.00194792 |
| 1.4375 | 84.5615673 | 0.3022291 | 0.00195307 |
| 1.43755 | 84.5825404 | 0.30027603 | 0.00195823 |
| 1.4376 | 84.6035948 | 0.2983178 | 0.0019634 |
| 1.43765 | 84.6247313 | 0.2963544 | 0.00196859 |
| 1.4377 | 84.6459511 | 0.29438581 | 0.00197379 |
| 1.43775 | 84.6672551 | 0.29241203 | 0.001979 |
| 1.4378 | 84.6886442 | 0.29043303 | 0.00198423 |
| 1.43785 | 84.7101196 | 0.2884488 | 0.00198946 |
| 1.4379 | 84.7316823 | 0.28645934 | 0.00199472 |
| 1.43795 | 84.7533334 | 0.28446462 | 0.00199998 |
| 1.438 | 84.7750739 | 0.28246464 | 0.00200526 |
| 1.43805 | 84.796905 | 0.28045938 | 0.00201055 |
| 1.4381 | 84.8188278 | 0.27844882 | 0.00201586 |
| 1.43815 | 84.8408436 | 0.27643296 | 0.00202118 |
| 1.4382 | 84.8629534 | 0.27441178 | 0.00202651 |
| 1.43825 | 84.8851586 | 0.27238527 | 0.00203186 |
| 1.4383 | 84.9074603 | 0.27035341 | 0.00203722 |
| 1.43835 | 84.9298598 | 0.26831619 | 0.00204259 |
| 1.4384 | 84.9523585 | 0.2662736 | 0.00204798 |
| 1.43845 | 84.9749577 | 0.26422562 | 0.00205338 |
| 1.4385 | 84.9976587 | 0.26217224 | 0.00205879 |
| 1.43855 | 85.0204629 | 0.26011345 | 0.00206422 |
| 1.4386 | 85.0433718 | 0.25804922 | 0.00206966 |
| 1.43865 | 85.0663867 | 0.25597956 | 0.00207512 |
| 1.4387 | 85.0895093 | 0.25390444 | 0.00208059 |
| 1.43875 | 85.112741 | 0.25182385 | 0.00208607 |
| 1.4388 | 85.1360834 | 0.24973778 | 0.00209157 |
| 1.43885 | 85.1595381 | 0.24764621 | 0.00209708 |
| 1.4389 | 85.1831067 | 0.24554913 | 0.0021026 |
| 1.43895 | 85.2067909 | 0.24344653 | 0.00210814 |
| 1.439 | 85.2305925 | 0.24133838 | 0.0021137 |
| 1.43905 | 85.2545132 | 0.23922469 | 0.00211926 |
| 1.4391 | 85.2785547 | 0.23710543 | 0.00212484 |
| 1.43915 | 85.3027191 | 0.23498058 | 0.00213044 |
| 1.4392 | 85.3270081 | 0.23285014 | 0.00213605 |
| 1.43925 | 85.3514237 | 0.2307141 | 0.00214167 |
| 1.4393 | 85.375968 | 0.22857243 | 0.00214731 |
| 1.43935 | 85.400643 | 0.22642512 | 0.00215296 |
| 1.4394 | 85.4254508 | 0.22427216 | 0.00215863 |
| 1.43945 | 85.4503935 | 0.22211353 | 0.00216431 |
| 1.4395 | 85.4754735 | 0.21994923 | 0.00217 |
| 1.43955 | 85.5006929 | 0.21777923 | 0.00217571 |
| 1.4396 | 85.5260543 | 0.21560352 | 0.00218143 |
| 1.43965 | 85.5515599 | 0.21342208 | 0.00218717 |
| 1.4397 | 85.5772123 | 0.21123491 | 0.00219292 |
| 1.43975 | 85.6030141 | 0.20904199 | 0.00219869 |
| 1.4398 | 85.6289679 | 0.2068433 | 0.00220447 |
| 1.43985 | 85.6550764 | 0.20463883 | 0.00221027 |
| 1.4399 | 85.6813424 | 0.20242856 | 0.00221608 |
| 1.43995 | 85.7077688 | 0.20021249 | 0.0022219 |
| 1.44 | 85.7343587 | 0.19799058 | 0.00222774 |
| 1.44005 | 85.761115 | 0.19576284 | 0.0022336 |
| 1.4401 | 85.788041 | 0.19352925 | 0.00223947 |
| 1.44015 | 85.81514 | 0.19128978 | 0.00224535 |
| 1.4402 | 85.8424152 | 0.18904443 | 0.00225125 |
| 1.44025 | 85.8698702 | 0.18679318 | 0.00225716 |
| 1.4403 | 85.8975087 | 0.18453602 | 0.00226309 |
| 1.44035 | 85.9253342 | 0.18227293 | 0.00226904 |
| 1.4404 | 85.9533508 | 0.18000389 | 0.00227499 |
| 1.44045 | 85.9815624 | 0.1777289 | 0.00228097 |
| 1.4405 | 86.0099732 | 0.17544793 | 0.00228696 |
| 1.44055 | 86.0385873 | 0.17316097 | 0.00229296 |
| 1.4406 | 86.0674093 | 0.17086801 | 0.00229898 |
| 1.44065 | 86.0964438 | 0.16856903 | 0.00230501 |
| 1.4407 | 86.1256955 | 0.16626402 | 0.00231106 |
| 1.44075 | 86.1551695 | 0.16395296 | 0.00231713 |
| 1.4408 | 86.1848707 | 0.16163583 | 0.00232321 |
| 1.44085 | 86.2148047 | 0.15931263 | 0.0023293 |
| 1.4409 | 86.244977 | 0.15698332 | 0.00233541 |
| 1.44095 | 86.2753933 | 0.15464791 | 0.00234154 |
| 1.441 | 86.3060596 | 0.15230637 | 0.00234768 |
| 1.44105 | 86.3369824 | 0.1499587 | 0.00235384 |
| 1.4411 | 86.3681681 | 0.14760486 | 0.00236001 |
| 1.44115 | 86.3996235 | 0.14524485 | 0.00236619 |
| 1.4412 | 86.4313558 | 0.14287866 | 0.0023724 |
| 1.44125 | 86.4633724 | 0.14050626 | 0.00237862 |
| 1.4413 | 86.4956812 | 0.13812764 | 0.00238485 |
| 1.44135 | 86.5282902 | 0.13574279 | 0.0023911 |
| 1.4414 | 86.5612081 | 0.13335169 | 0.00239737 |
| 1.44145 | 86.5944438 | 0.13095433 | 0.00240365 |
| 1.4415 | 86.6280066 | 0.12855068 | 0.00240995 |
| 1.44155 | 86.6619065 | 0.12614073 | 0.00241626 |
| 1.4416 | 86.6961538 | 0.12372447 | 0.00242259 |
| 1.44165 | 86.7307594 | 0.12130189 | 0.00242893 |
| 1.4417 | 86.7657349 | 0.11887295 | 0.00243529 |
| 1.44175 | 86.8010924 | 0.11643766 | 0.00244167 |
| 1.4418 | 86.8368446 | 0.11399599 | 0.00244806 |
| 1.44185 | 86.8730052 | 0.11154793 | 0.00245447 |
| 1.4419 | 86.9095884 | 0.10909345 | 0.0024609 |
| 1.44195 | 86.9466095 | 0.10663256 | 0.00246734 |
| 1.442 | 86.9840845 | 0.10416522 | 0.0024738 |
| 1.44205 | 87.0220306 | 0.10169142 | 0.00248027 |
| 1.4421 | 87.0604662 | 0.09921116 | 0.00248676 |
| 1.44215 | 87.0994105 | 0.0967244 | 0.00249326 |
| 1.4422 | 87.1388844 | 0.09423113 | 0.00249979 |
| 1.44225 | 87.1789101 | 0.09173135 | 0.00250633 |
| 1.4423 | 87.2195115 | 0.08922502 | 0.00251288 |
| 1.44235 | 87.2607141 | 0.08671214 | 0.00251945 |
| 1.4424 | 87.3025455 | 0.08419269 | 0.00252604 |
| 1.44245 | 87.3450354 | 0.08166665 | 0.00253264 |
| 1.4425 | 87.388216 | 0.07913401 | 0.00253927 |
| 1.44255 | 87.432122 | 0.07659474 | 0.0025459 |
| 1.4426 | 87.4767915 | 0.07404884 | 0.00255256 |
| 1.44265 | 87.5222656 | 0.07149628 | 0.00255923 |
| 1.4427 | 87.5685894 | 0.06893705 | 0.00256592 |
| 1.44275 | 87.6158127 | 0.06637114 | 0.00257262 |
| 1.4428 | 87.6639898 | 0.06379852 | 0.00257934 |
| 1.44285 | 87.7131811 | 0.06121917 | 0.00258608 |
| 1.4429 | 87.7634534 | 0.05863309 | 0.00259283 |
| 1.44295 | 87.8148815 | 0.05604026 | 0.00259961 |
| 1.443 | 87.8675488 | 0.05344065 | 0.0026064 |
| 1.44305 | 87.9215497 | 0.05083426 | 0.0026132 |
| 1.4431 | 87.9769908 | 0.04822106 | 0.00262002 |
| 1.44315 | 88.0339942 | 0.04560103 | 0.00262686 |
| 1.4432 | 88.0926997 | 0.04297417 | 0.00263372 |
| 1.44325 | 88.1532697 | 0.04034045 | 0.0026406 |
| 1.4433 | 88.2158942 | 0.03769985 | 0.00264749 |
| 1.44335 | 88.2807976 | 0.03505236 | 0.0026544 |
| 1.4434 | 88.3482485 | 0.03239797 | 0.00266132 |
| 1.44345 | 88.4185729 | 0.02973665 | 0.00266827 |
| 1.4435 | 88.4921728 | 0.02706838 | 0.00267523 |
| 1.44355 | 88.5695537 | 0.02439315 | 0.0026822 |
| 1.4436 | 88.6513662 | 0.02171095 | 0.0026892 |
| 1.44365 | 88.7384722 | 0.01902175 | 0.00269621 |
| 1.4437 | 88.8320556 | 0.01632554 | 0.00270324 |
| 1.44375 | 88.9338206 | 0.01362229 | 0.00271029 |
| 1.4438 | 89.0463829 | 0.010912 | 0.00271736 |
| 1.44385 | 89.1741457 | 0.00819464 | 0.00272444 |
| 1.4439 | 89.3256948 | 0.0054702 | 0.00273154 |
| 1.44395 | 89.5231956 | 0.00273866 | 0.00273866 |

**Table 5.** Simulation results for 1-cm sensor over three sensing zones.

| RI @1550 nm | Normalized power (a.u.) |
| --- | --- |
| 1.3165 | 0.990170988 |
| 1.319 | 0.989931496 |
| 1.3215 | 0.98967778 |
| 1.324 | 0.989408581 |
| 1.3265 | 0.989122489 |
| 1.329 | 0.988817912 |
| 1.3315 | 0.988493049 |
| 1.334 | 0.988145848 |
| 1.3365 | 0.987773959 |
| 1.339 | 0.987374679 |
| 1.3415 | 0.986944865 |
| 1.344 | 0.986480838 |
| 1.3465 | 0.985978247 |
| 1.349 | 0.985431875 |
| 1.3515 | 0.984835377 |
| 1.354 | 0.984180871 |
| 1.3565 | 0.983458307 |
| 1.359 | 0.982654379 |
| 1.3615 | 0.98175047 |
| 1.364 | 0.980718085 |
| 1.3665 | 0.979505765 |
| 1.369 | 0.977973493 |
| 1.37 | 0.977151212 |
| 1.37005 | 0.977087678 |
| 1.3701 | 0.977023986 |
| 1.37015 | 0.976960136 |
| 1.3702 | 0.976896127 |
| 1.37025 | 0.976831959 |
| 1.3703 | 0.976767632 |
| 1.37035 | 0.976703144 |
| 1.3704 | 0.976638496 |
| 1.37045 | 0.976573687 |
| 1.3705 | 0.976508717 |
| 1.37055 | 0.976443585 |
| 1.3706 | 0.976378291 |
| 1.37065 | 0.976312834 |
| 1.3707 | 0.976247215 |
| 1.37075 | 0.976181432 |
| 1.3708 | 0.976115485 |
| 1.37085 | 0.976049374 |
| 1.3709 | 0.975983098 |
| 1.37095 | 0.975916658 |
| 1.371 | 0.975850051 |
| 1.37105 | 0.975783279 |
| 1.3711 | 0.97571634 |
| 1.37115 | 0.975649234 |
| 1.3712 | 0.975581961 |
| 1.37125 | 0.97551452 |
| 1.3713 | 0.975446911 |
| 1.37135 | 0.975379134 |
| 1.3714 | 0.975311187 |
| 1.37145 | 0.975243071 |
| 1.3715 | 0.975174784 |
| 1.37155 | 0.975106328 |
| 1.3716 | 0.9750377 |
| 1.37165 | 0.974968901 |
| 1.3717 | 0.974899931 |
| 1.37175 | 0.974830788 |
| 1.3718 | 0.974761472 |
| 1.37185 | 0.974691984 |
| 1.3719 | 0.974622322 |
| 1.37195 | 0.974552485 |
| 1.372 | 0.974482475 |
| 1.37205 | 0.974412289 |
| 1.3721 | 0.974341928 |
| 1.37215 | 0.974271391 |
| 1.3722 | 0.974200678 |
| 1.37225 | 0.974129787 |
| 1.3723 | 0.97405872 |
| 1.37235 | 0.973987475 |
| 1.3724 | 0.973916052 |
| 1.37245 | 0.97384445 |
| 1.3725 | 0.973772668 |
| 1.37255 | 0.973700708 |
| 1.3726 | 0.973628567 |
| 1.37265 | 0.973556246 |
| 1.3727 | 0.973483743 |
| 1.37275 | 0.973411059 |
| 1.3728 | 0.973338193 |
| 1.37285 | 0.973265145 |
| 1.3729 | 0.973191914 |
| 1.37295 | 0.973118499 |
| 1.373 | 0.9730449 |
| 1.37305 | 0.972971117 |
| 1.3731 | 0.972897149 |
| 1.37315 | 0.972822996 |
| 1.3732 | 0.972748657 |
| 1.37325 | 0.972674132 |
| 1.3733 | 0.97259942 |
| 1.37335 | 0.97252452 |
| 1.3734 | 0.972449433 |
| 1.37345 | 0.972374157 |
| 1.3735 | 0.972298693 |
| 1.37355 | 0.972223039 |
| 1.3736 | 0.972147195 |
| 1.37365 | 0.972071162 |
| 1.3737 | 0.971994937 |
| 1.37375 | 0.971918521 |
| 1.3738 | 0.971841913 |
| 1.37385 | 0.971765113 |
| 1.3739 | 0.971688121 |
| 1.37395 | 0.971610935 |
| 1.374 | 0.971533555 |
| 1.37405 | 0.971455981 |
| 1.3741 | 0.971378212 |
| 1.37415 | 0.971300247 |
| 1.3742 | 0.971222087 |
| 1.37425 | 0.97114373 |
| 1.3743 | 0.971065177 |
| 1.37435 | 0.970986426 |
| 1.3744 | 0.970907477 |
| 1.37445 | 0.97082833 |
| 1.3745 | 0.970748984 |
| 1.37455 | 0.970669438 |
| 1.3746 | 0.970589692 |
| 1.37465 | 0.970509746 |
| 1.3747 | 0.970429599 |
| 1.37475 | 0.97034925 |
| 1.3748 | 0.970268699 |
| 1.37485 | 0.970187945 |
| 1.3749 | 0.970106988 |
| 1.37495 | 0.970025828 |
| 1.375 | 0.969944463 |
| 1.37505 | 0.969862893 |
| 1.3751 | 0.969781119 |
| 1.37515 | 0.969699138 |
| 1.3752 | 0.969616951 |
| 1.37525 | 0.969534557 |
| 1.3753 | 0.969451955 |
| 1.37535 | 0.969369145 |
| 1.3754 | 0.969286127 |
| 1.37545 | 0.9692029 |
| 1.3755 | 0.969119463 |
| 1.37555 | 0.969035816 |
| 1.3756 | 0.968951958 |
| 1.37565 | 0.968867888 |
| 1.3757 | 0.968783607 |
| 1.37575 | 0.968699114 |
| 1.3758 | 0.968614407 |
| 1.37585 | 0.968529487 |
| 1.3759 | 0.968444352 |
| 1.37595 | 0.968359003 |
| 1.376 | 0.968273439 |
| 1.37605 | 0.968187659 |
| 1.3761 | 0.968101663 |
| 1.37615 | 0.96801545 |
| 1.3762 | 0.967929019 |
| 1.37625 | 0.96784237 |
| 1.3763 | 0.967755502 |
| 1.37635 | 0.967668416 |
| 1.3764 | 0.967581109 |
| 1.37645 | 0.967493582 |
| 1.3765 | 0.967405834 |
| 1.37655 | 0.967317865 |
| 1.3766 | 0.967229673 |
| 1.37665 | 0.967141259 |
| 1.3767 | 0.967052622 |
| 1.37675 | 0.96696376 |
| 1.3768 | 0.966874675 |
| 1.37685 | 0.966785364 |
| 1.3769 | 0.966695827 |
| 1.37695 | 0.966606065 |
| 1.377 | 0.966516075 |
| 1.37705 | 0.966425858 |
| 1.3771 | 0.966335413 |
| 1.37715 | 0.96624474 |
| 1.3772 | 0.966153837 |
| 1.37725 | 0.966062705 |
| 1.3773 | 0.965971342 |
| 1.37735 | 0.965879748 |
| 1.3774 | 0.965787922 |
| 1.37745 | 0.965695865 |
| 1.3775 | 0.965603574 |
| 1.37755 | 0.96551105 |
| 1.3776 | 0.965418292 |
| 1.37765 | 0.9653253 |
| 1.3777 | 0.965232072 |
| 1.37775 | 0.965138608 |
| 1.3778 | 0.965044908 |
| 1.37785 | 0.96495097 |
| 1.3779 | 0.964856795 |
| 1.37795 | 0.964762381 |
| 1.378 | 0.964667729 |
| 1.37805 | 0.964572837 |
| 1.3781 | 0.964477704 |
| 1.37815 | 0.964382331 |
| 1.3782 | 0.964286716 |
| 1.37825 | 0.964190859 |
| 1.3783 | 0.964094759 |
| 1.37835 | 0.963998416 |
| 1.3784 | 0.963901829 |
| 1.37845 | 0.963804997 |
| 1.3785 | 0.96370792 |
| 1.37855 | 0.963610597 |
| 1.3786 | 0.963513027 |
| 1.37865 | 0.96341521 |
| 1.3787 | 0.963317145 |
| 1.37875 | 0.963218832 |
| 1.3788 | 0.963120269 |
| 1.37885 | 0.963021457 |
| 1.3789 | 0.962922394 |
| 1.37895 | 0.962823079 |
| 1.379 | 0.962723513 |
| 1.37905 | 0.962623695 |
| 1.3791 | 0.962523623 |
| 1.37915 | 0.962423298 |
| 1.3792 | 0.962322718 |
| 1.37925 | 0.962221883 |
| 1.3793 | 0.962120792 |
| 1.37935 | 0.962019445 |
| 1.3794 | 0.961917841 |
| 1.37945 | 0.961815978 |
| 1.3795 | 0.961713858 |
| 1.37955 | 0.961611478 |
| 1.3796 | 0.961508838 |
| 1.37965 | 0.961405938 |
| 1.3797 | 0.961302776 |
| 1.37975 | 0.961199353 |
| 1.3798 | 0.961095667 |
| 1.37985 | 0.960991718 |
| 1.3799 | 0.960887505 |
| 1.37995 | 0.960783027 |
| 1.38 | 0.960678284 |
| 1.38005 | 0.960573275 |
| 1.3801 | 0.960468 |
| 1.38015 | 0.960362456 |
| 1.3802 | 0.960256645 |
| 1.38025 | 0.960150565 |
| 1.3803 | 0.960044216 |
| 1.38035 | 0.959937596 |
| 1.3804 | 0.959830705 |
| 1.38045 | 0.959723543 |
| 1.3805 | 0.959616108 |
| 1.38055 | 0.959508401 |
| 1.3806 | 0.959400419 |
| 1.38065 | 0.959292163 |
| 1.3807 | 0.959183632 |
| 1.38075 | 0.959074825 |
| 1.3808 | 0.958965741 |
| 1.38085 | 0.95885638 |
| 1.3809 | 0.958746741 |
| 1.38095 | 0.958636823 |
| 1.381 | 0.958526626 |
| 1.38105 | 0.958416148 |
| 1.3811 | 0.95830539 |
| 1.38115 | 0.958194349 |
| 1.3812 | 0.958083026 |
| 1.38125 | 0.95797142 |
| 1.3813 | 0.95785953 |
| 1.38135 | 0.957747356 |
| 1.3814 | 0.957634896 |
| 1.38145 | 0.957522149 |
| 1.3815 | 0.957409116 |
| 1.38155 | 0.957295795 |
| 1.3816 | 0.957182186 |
| 1.38165 | 0.957068288 |
| 1.3817 | 0.9569541 |
| 1.38175 | 0.956839621 |
| 1.3818 | 0.95672485 |
| 1.38185 | 0.956609788 |
| 1.3819 | 0.956494432 |
| 1.38195 | 0.956378783 |
| 1.382 | 0.956262839 |
| 1.38205 | 0.9561466 |
| 1.3821 | 0.956030065 |
| 1.38215 | 0.955913233 |
| 1.3822 | 0.955796104 |
| 1.38225 | 0.955678676 |
| 1.3823 | 0.955560949 |
| 1.38235 | 0.955442922 |
| 1.3824 | 0.955324595 |
| 1.38245 | 0.955205966 |
| 1.3825 | 0.955087035 |
| 1.38255 | 0.954967801 |
| 1.3826 | 0.954848263 |
| 1.38265 | 0.95472842 |
| 1.3827 | 0.954608272 |
| 1.38275 | 0.954487818 |
| 1.3828 | 0.954367056 |
| 1.38285 | 0.954245987 |
| 1.3829 | 0.95412461 |
| 1.38295 | 0.954002923 |
| 1.383 | 0.953880925 |
| 1.38305 | 0.953758617 |
| 1.3831 | 0.953635997 |
| 1.38315 | 0.953513064 |
| 1.3832 | 0.953389818 |
| 1.38325 | 0.953266257 |
| 1.3833 | 0.953142381 |
| 1.38335 | 0.95301819 |
| 1.3834 | 0.952893682 |
| 1.38345 | 0.952768856 |
| 1.3835 | 0.952643711 |
| 1.38355 | 0.952518248 |
| 1.3836 | 0.952392464 |
| 1.38365 | 0.95226636 |
| 1.3837 | 0.952139934 |
| 1.38375 | 0.952013185 |
| 1.3838 | 0.951886113 |
| 1.38385 | 0.951758717 |
| 1.3839 | 0.951630995 |
| 1.38395 | 0.951502948 |
| 1.384 | 0.951374574 |
| 1.38405 | 0.951245872 |
| 1.3841 | 0.951116842 |
| 1.38415 | 0.950987482 |
| 1.3842 | 0.950857793 |
| 1.38425 | 0.950727772 |
| 1.3843 | 0.950597419 |
| 1.38435 | 0.950466734 |
| 1.3844 | 0.950335715 |
| 1.38445 | 0.950204361 |
| 1.3845 | 0.950072672 |
| 1.38455 | 0.949940647 |
| 1.3846 | 0.949808285 |
| 1.38465 | 0.949675585 |
| 1.3847 | 0.949542545 |
| 1.38475 | 0.949409167 |
| 1.3848 | 0.949275447 |
| 1.38485 | 0.949141386 |
| 1.3849 | 0.949006983 |
| 1.38495 | 0.948872236 |
| 1.385 | 0.948737145 |
| 1.38505 | 0.948601709 |
| 1.3851 | 0.948465927 |
| 1.38515 | 0.948329798 |
| 1.3852 | 0.948193321 |
| 1.38525 | 0.948056496 |
| 1.3853 | 0.947919321 |
| 1.38535 | 0.947781796 |
| 1.3854 | 0.947643919 |
| 1.38545 | 0.94750569 |
| 1.3855 | 0.947367107 |
| 1.38555 | 0.947228171 |
| 1.3856 | 0.947088879 |
| 1.38565 | 0.946949232 |
| 1.3857 | 0.946809227 |
| 1.38575 | 0.946668865 |
| 1.3858 | 0.946528144 |
| 1.38585 | 0.946387063 |
| 1.3859 | 0.946245622 |
| 1.38595 | 0.946103819 |
| 1.386 | 0.945961653 |
| 1.38605 | 0.945819124 |
| 1.3861 | 0.945676231 |
| 1.38615 | 0.945532973 |
| 1.3862 | 0.945389348 |
| 1.38625 | 0.945245355 |
| 1.3863 | 0.945100995 |
| 1.38635 | 0.944956266 |
| 1.3864 | 0.944811166 |
| 1.38645 | 0.944665696 |
| 1.3865 | 0.944519853 |
| 1.38655 | 0.944373637 |
| 1.3866 | 0.944227048 |
| 1.38665 | 0.944080083 |
| 1.3867 | 0.943932743 |
| 1.38675 | 0.943785026 |
| 1.3868 | 0.943636931 |
| 1.38685 | 0.943488457 |
| 1.3869 | 0.943339603 |
| 1.38695 | 0.943190369 |
| 1.387 | 0.943040753 |
| 1.38705 | 0.942890754 |
| 1.3871 | 0.942740371 |
| 1.38715 | 0.942589604 |
| 1.3872 | 0.94243845 |
| 1.38725 | 0.942286911 |
| 1.3873 | 0.942134983 |
| 1.38735 | 0.941982666 |
| 1.3874 | 0.94182996 |
| 1.38745 | 0.941676863 |
| 1.3875 | 0.941523375 |
| 1.38755 | 0.941369493 |
| 1.3876 | 0.941215218 |
| 1.38765 | 0.941060548 |
| 1.3877 | 0.940905482 |
| 1.38775 | 0.940750019 |
| 1.3878 | 0.940594159 |
| 1.38785 | 0.940437899 |
| 1.3879 | 0.94028124 |
| 1.38795 | 0.940124179 |
| 1.388 | 0.939966717 |
| 1.38805 | 0.939808851 |
| 1.3881 | 0.939650581 |
| 1.38815 | 0.939491907 |
| 1.3882 | 0.939332826 |
| 1.38825 | 0.939173337 |
| 1.3883 | 0.939013441 |
| 1.38835 | 0.938853135 |
| 1.3884 | 0.938692418 |
| 1.38845 | 0.938531291 |
| 1.3885 | 0.93836975 |
| 1.38855 | 0.938207796 |
| 1.3886 | 0.938045428 |
| 1.38865 | 0.937882643 |
| 1.3887 | 0.937719442 |
| 1.38875 | 0.937555823 |
| 1.3888 | 0.937391785 |
| 1.38885 | 0.937227327 |
| 1.3889 | 0.937062447 |
| 1.38895 | 0.936897146 |
| 1.389 | 0.936731421 |
| 1.38905 | 0.936565272 |
| 1.3891 | 0.936398697 |
| 1.38915 | 0.936231696 |
| 1.3892 | 0.936064267 |
| 1.38925 | 0.935896409 |
| 1.3893 | 0.935728121 |
| 1.38935 | 0.935559402 |
| 1.3894 | 0.935390251 |
| 1.38945 | 0.935220667 |
| 1.3895 | 0.935050649 |
| 1.38955 | 0.934880194 |
| 1.3896 | 0.934709304 |
| 1.38965 | 0.934537975 |
| 1.3897 | 0.934366208 |
| 1.38975 | 0.934194001 |
| 1.3898 | 0.934021352 |
| 1.38985 | 0.933848262 |
| 1.3899 | 0.933674727 |
| 1.38995 | 0.933500749 |
| 1.39 | 0.933326324 |
| 1.39005 | 0.933151453 |
| 1.3901 | 0.932976134 |
| 1.39015 | 0.932800365 |
| 1.3902 | 0.932624147 |
| 1.39025 | 0.932447476 |
| 1.3903 | 0.932270354 |
| 1.39035 | 0.932092777 |
| 1.3904 | 0.931914745 |
| 1.39045 | 0.931736257 |
| 1.3905 | 0.931557312 |
| 1.39055 | 0.931377909 |
| 1.3906 | 0.931198045 |
| 1.39065 | 0.931017721 |
| 1.3907 | 0.930836935 |
| 1.39075 | 0.930655686 |
| 1.3908 | 0.930473972 |
| 1.39085 | 0.930291792 |
| 1.3909 | 0.930109146 |
| 1.39095 | 0.929926031 |
| 1.391 | 0.929742448 |
| 1.39105 | 0.929558394 |
| 1.3911 | 0.929373868 |
| 1.39115 | 0.929188869 |
| 1.3912 | 0.929003397 |
| 1.39125 | 0.928817449 |
| 1.3913 | 0.928631024 |
| 1.39135 | 0.928444122 |
| 1.3914 | 0.928256741 |
| 1.39145 | 0.928068879 |
| 1.3915 | 0.927880536 |
| 1.39155 | 0.927691711 |
| 1.3916 | 0.927502401 |
| 1.39165 | 0.927312607 |
| 1.3917 | 0.927122325 |
| 1.39175 | 0.926931557 |
| 1.3918 | 0.926740299 |
| 1.39185 | 0.926548551 |
| 1.3919 | 0.926356312 |
| 1.39195 | 0.92616358 |
| 1.392 | 0.925970354 |
| 1.39205 | 0.925776633 |
| 1.3921 | 0.925582415 |
| 1.39215 | 0.9253877 |
| 1.3922 | 0.925192485 |
| 1.39225 | 0.92499677 |
| 1.3923 | 0.924800554 |
| 1.39235 | 0.924603835 |
| 1.3924 | 0.924406611 |
| 1.39245 | 0.924208882 |
| 1.3925 | 0.924010646 |
| 1.39255 | 0.923811902 |
| 1.3926 | 0.923612649 |
| 1.39265 | 0.923412885 |
| 1.3927 | 0.923212609 |
| 1.39275 | 0.923011819 |
| 1.3928 | 0.922810515 |
| 1.39285 | 0.922608695 |
| 1.3929 | 0.922406358 |
| 1.39295 | 0.922203502 |
| 1.393 | 0.922000126 |
| 1.39305 | 0.921796229 |
| 1.3931 | 0.921591809 |
| 1.39315 | 0.921386866 |
| 1.3932 | 0.921181397 |
| 1.39325 | 0.920975401 |
| 1.3933 | 0.920768877 |
| 1.39335 | 0.920561824 |
| 1.3934 | 0.920354241 |
| 1.39345 | 0.920146125 |
| 1.3935 | 0.919937476 |
| 1.39355 | 0.919728292 |
| 1.3936 | 0.919518572 |
| 1.39365 | 0.919308314 |
| 1.3937 | 0.919097518 |
| 1.39375 | 0.918886181 |
| 1.3938 | 0.918674302 |
| 1.39385 | 0.918461881 |
| 1.3939 | 0.918248915 |
| 1.39395 | 0.918035403 |
| 1.394 | 0.917821344 |
| 1.39405 | 0.917606736 |
| 1.3941 | 0.917391579 |
| 1.39415 | 0.917175869 |
| 1.3942 | 0.916959607 |
| 1.39425 | 0.916742791 |
| 1.3943 | 0.916525419 |
| 1.39435 | 0.91630749 |
| 1.3944 | 0.916089002 |
| 1.39445 | 0.915869955 |
| 1.3945 | 0.915650346 |
| 1.39455 | 0.915430174 |
| 1.3946 | 0.915209438 |
| 1.39465 | 0.914988136 |
| 1.3947 | 0.914766267 |
| 1.39475 | 0.914543829 |
| 1.3948 | 0.914320822 |
| 1.39485 | 0.914097242 |
| 1.3949 | 0.91387309 |
| 1.39495 | 0.913648364 |
| 1.395 | 0.913423061 |
| 1.39505 | 0.913197181 |
| 1.3951 | 0.912970723 |
| 1.39515 | 0.912743684 |
| 1.3952 | 0.912516063 |
| 1.39525 | 0.912287859 |
| 1.3953 | 0.91205907 |
| 1.39535 | 0.911829695 |
| 1.3954 | 0.911599732 |
| 1.39545 | 0.91136918 |
| 1.3955 | 0.911138037 |
| 1.39555 | 0.910906301 |
| 1.3956 | 0.910673972 |
| 1.39565 | 0.910441048 |
| 1.3957 | 0.910207527 |
| 1.39575 | 0.909973407 |
| 1.3958 | 0.909738688 |
| 1.39585 | 0.909503367 |
| 1.3959 | 0.909267443 |
| 1.39595 | 0.909030915 |
| 1.396 | 0.908793781 |
| 1.39605 | 0.908556039 |
| 1.3961 | 0.908317688 |
| 1.39615 | 0.908078726 |
| 1.3962 | 0.907839153 |
| 1.39625 | 0.907598965 |
| 1.3963 | 0.907358162 |
| 1.39635 | 0.907116743 |
| 1.3964 | 0.906874704 |
| 1.39645 | 0.906632046 |
| 1.3965 | 0.906388766 |
| 1.39655 | 0.906144863 |
| 1.3966 | 0.905900335 |
| 1.39665 | 0.905655181 |
| 1.3967 | 0.905409399 |
| 1.39675 | 0.905162987 |
| 1.3968 | 0.904915944 |
| 1.39685 | 0.904668268 |
| 1.3969 | 0.904419958 |
| 1.39695 | 0.904171012 |
| 1.397 | 0.903921428 |
| 1.39705 | 0.903671205 |
| 1.3971 | 0.903420341 |
| 1.39715 | 0.903168835 |
| 1.3972 | 0.902916684 |
| 1.39725 | 0.902663888 |
| 1.3973 | 0.902410445 |
| 1.39735 | 0.902156352 |
| 1.3974 | 0.901901608 |
| 1.39745 | 0.901646213 |
| 1.3975 | 0.901390163 |
| 1.39755 | 0.901133457 |
| 1.3976 | 0.900876095 |
| 1.39765 | 0.900618073 |
| 1.3977 | 0.90035939 |
| 1.39775 | 0.900100045 |
| 1.3978 | 0.899840037 |
| 1.39785 | 0.899579362 |
| 1.3979 | 0.89931802 |
| 1.39795 | 0.899056009 |
| 1.398 | 0.898793327 |
| 1.39805 | 0.898529973 |
| 1.3981 | 0.898265944 |
| 1.39815 | 0.898001239 |
| 1.3982 | 0.897735857 |
| 1.39825 | 0.897469796 |
| 1.3983 | 0.897203053 |
| 1.39835 | 0.896935628 |
| 1.3984 | 0.896667518 |
| 1.39845 | 0.896398722 |
| 1.3985 | 0.896129237 |
| 1.39855 | 0.895859064 |
| 1.3986 | 0.895588198 |
| 1.39865 | 0.895316639 |
| 1.3987 | 0.895044386 |
| 1.39875 | 0.894771435 |
| 1.3988 | 0.894497786 |
| 1.39885 | 0.894223437 |
| 1.3989 | 0.893948386 |
| 1.39895 | 0.893672631 |
| 1.399 | 0.89339617 |
| 1.39905 | 0.893119002 |
| 1.3991 | 0.892841124 |
| 1.39915 | 0.892562536 |
| 1.3992 | 0.892283234 |
| 1.39925 | 0.892003219 |
| 1.3993 | 0.891722486 |
| 1.39935 | 0.891441036 |
| 1.3994 | 0.891158865 |
| 1.39945 | 0.890875973 |
| 1.3995 | 0.890592357 |
| 1.39955 | 0.890308016 |
| 1.3996 | 0.890022947 |
| 1.39965 | 0.889737149 |
| 1.3997 | 0.88945062 |
| 1.39975 | 0.889163358 |
| 1.3998 | 0.888875361 |
| 1.39985 | 0.888586628 |
| 1.3999 | 0.888297156 |
| 1.39995 | 0.888006944 |
| 1.4 | 0.88771599 |
| 1.40005 | 0.887424292 |
| 1.4001 | 0.887131848 |
| 1.40015 | 0.886838656 |
| 1.4002 | 0.886544714 |
| 1.40025 | 0.886250021 |
| 1.4003 | 0.885954574 |
| 1.40035 | 0.885658372 |
| 1.4004 | 0.885361413 |
| 1.40045 | 0.885063695 |
| 1.4005 | 0.884765215 |
| 1.40055 | 0.884465972 |
| 1.4006 | 0.884165965 |
| 1.40065 | 0.88386519 |
| 1.4007 | 0.883563647 |
| 1.40075 | 0.883261333 |
| 1.4008 | 0.882958246 |
| 1.40085 | 0.882654385 |
| 1.4009 | 0.882349747 |
| 1.40095 | 0.88204433 |
| 1.401 | 0.881738133 |
| 1.40105 | 0.881431154 |
| 1.4011 | 0.88112339 |
| 1.40115 | 0.880814839 |
| 1.4012 | 0.880505501 |
| 1.40125 | 0.880195371 |
| 1.4013 | 0.87988445 |
| 1.40135 | 0.879572734 |
| 1.4014 | 0.879260222 |
| 1.40145 | 0.878946911 |
| 1.4015 | 0.8786328 |
| 1.40155 | 0.878317887 |
| 1.4016 | 0.878002169 |
| 1.40165 | 0.877685645 |
| 1.4017 | 0.877368313 |
| 1.40175 | 0.877050169 |
| 1.4018 | 0.876731214 |
| 1.40185 | 0.876411444 |
| 1.4019 | 0.876090857 |
| 1.40195 | 0.875769451 |
| 1.402 | 0.875447225 |
| 1.40205 | 0.875124176 |
| 1.4021 | 0.874800303 |
| 1.40215 | 0.874475602 |
| 1.4022 | 0.874150072 |
| 1.40225 | 0.873823712 |
| 1.4023 | 0.873496518 |
| 1.40235 | 0.873168489 |
| 1.4024 | 0.872839622 |
| 1.40245 | 0.872509916 |
| 1.4025 | 0.872179369 |
| 1.40255 | 0.871847978 |
| 1.4026 | 0.871515741 |
| 1.40265 | 0.871182656 |
| 1.4027 | 0.870848721 |
| 1.40275 | 0.870513934 |
| 1.4028 | 0.870178293 |
| 1.40285 | 0.869841795 |
| 1.4029 | 0.869504439 |
| 1.40295 | 0.869166222 |
| 1.403 | 0.868827142 |
| 1.40305 | 0.868487198 |
| 1.4031 | 0.868146386 |
| 1.40315 | 0.867804704 |
| 1.4032 | 0.867462151 |
| 1.40325 | 0.867118725 |
| 1.4033 | 0.866774422 |
| 1.40335 | 0.866429241 |
| 1.4034 | 0.86608318 |
| 1.40345 | 0.865736237 |
| 1.4035 | 0.865388409 |
| 1.40355 | 0.865039693 |
| 1.4036 | 0.864690089 |
| 1.40365 | 0.864339593 |
| 1.4037 | 0.863988204 |
| 1.40375 | 0.863635919 |
| 1.4038 | 0.863282736 |
| 1.40385 | 0.862928653 |
| 1.4039 | 0.862573667 |
| 1.40395 | 0.862217776 |
| 1.404 | 0.861860978 |
| 1.40405 | 0.861503271 |
| 1.4041 | 0.861144652 |
| 1.40415 | 0.860785119 |
| 1.4042 | 0.86042467 |
| 1.40425 | 0.860063303 |
| 1.4043 | 0.859701015 |
| 1.40435 | 0.859337804 |
| 1.4044 | 0.858973667 |
| 1.40445 | 0.858608603 |
| 1.4045 | 0.858242609 |
| 1.40455 | 0.857875683 |
| 1.4046 | 0.857507822 |
| 1.40465 | 0.857139024 |
| 1.4047 | 0.856769287 |
| 1.40475 | 0.856398608 |
| 1.4048 | 0.856026986 |
| 1.40485 | 0.855654417 |
| 1.4049 | 0.855280899 |
| 1.40495 | 0.85490643 |
| 1.405 | 0.854531008 |
| 1.40505 | 0.854154631 |
| 1.4051 | 0.853777295 |
| 1.40515 | 0.853398998 |
| 1.4052 | 0.853019739 |
| 1.40525 | 0.852639514 |
| 1.4053 | 0.852258322 |
| 1.40535 | 0.851876159 |
| 1.4054 | 0.851493024 |
| 1.40545 | 0.851108914 |
| 1.4055 | 0.850723827 |
| 1.40555 | 0.85033776 |
| 1.4056 | 0.84995071 |
| 1.40565 | 0.849562676 |
| 1.4057 | 0.849173655 |
| 1.40575 | 0.848783644 |
| 1.4058 | 0.848392641 |
| 1.40585 | 0.848000644 |
| 1.4059 | 0.84760765 |
| 1.40595 | 0.847213656 |
| 1.406 | 0.846818661 |
| 1.40605 | 0.846422661 |
| 1.4061 | 0.846025654 |
| 1.40615 | 0.845627638 |
| 1.4062 | 0.845228609 |
| 1.40625 | 0.844828567 |
| 1.4063 | 0.844427507 |
| 1.40635 | 0.844025428 |
| 1.4064 | 0.843622327 |
| 1.40645 | 0.843218202 |
| 1.4065 | 0.84281305 |
| 1.40655 | 0.842406868 |
| 1.4066 | 0.841999654 |
| 1.40665 | 0.841591405 |
| 1.4067 | 0.841182119 |
| 1.40675 | 0.840771793 |
| 1.4068 | 0.840360425 |
| 1.40685 | 0.839948011 |
| 1.4069 | 0.839534551 |
| 1.40695 | 0.83912004 |
| 1.407 | 0.838704476 |
| 1.40705 | 0.838287857 |
| 1.4071 | 0.83787018 |
| 1.40715 | 0.837451443 |
| 1.4072 | 0.837031642 |
| 1.40725 | 0.836610775 |
| 1.4073 | 0.83618884 |
| 1.40735 | 0.835765834 |
| 1.4074 | 0.835341755 |
| 1.40745 | 0.834916598 |
| 1.4075 | 0.834490363 |
| 1.40755 | 0.834063046 |
| 1.4076 | 0.833634645 |
| 1.40765 | 0.833205157 |
| 1.4077 | 0.832774578 |
| 1.40775 | 0.832342908 |
| 1.4078 | 0.831910142 |
| 1.40785 | 0.831476279 |
| 1.4079 | 0.831041315 |
| 1.40795 | 0.830605248 |
| 1.408 | 0.830168075 |
| 1.40805 | 0.829729793 |
| 1.4081 | 0.8292904 |
| 1.40815 | 0.828849892 |
| 1.4082 | 0.828408268 |
| 1.40825 | 0.827965524 |
| 1.4083 | 0.827521658 |
| 1.40835 | 0.827076666 |
| 1.4084 | 0.826630547 |
| 1.40845 | 0.826183297 |
| 1.4085 | 0.825734913 |
| 1.40855 | 0.825285393 |
| 1.4086 | 0.824834734 |
| 1.40865 | 0.824382933 |
| 1.4087 | 0.823929988 |
| 1.40875 | 0.823475895 |
| 1.4088 | 0.823020652 |
| 1.40885 | 0.822564255 |
| 1.4089 | 0.822106703 |
| 1.40895 | 0.821647992 |
| 1.409 | 0.821188119 |
| 1.40905 | 0.820727081 |
| 1.4091 | 0.820264877 |
| 1.40915 | 0.819801502 |
| 1.4092 | 0.819336954 |
| 1.40925 | 0.81887123 |
| 1.4093 | 0.818404327 |
| 1.40935 | 0.817936242 |
| 1.4094 | 0.817466973 |
| 1.40945 | 0.816996516 |
| 1.4095 | 0.816524869 |
| 1.40955 | 0.816052028 |
| 1.4096 | 0.815577992 |
| 1.40965 | 0.815102755 |
| 1.4097 | 0.814626317 |
| 1.40975 | 0.814148674 |
| 1.4098 | 0.813669822 |
| 1.40985 | 0.81318976 |
| 1.4099 | 0.812708483 |
| 1.40995 | 0.81222599 |
| 1.41 | 0.811742277 |
| 1.41005 | 0.81125734 |
| 1.4101 | 0.810771178 |
| 1.41015 | 0.810283787 |
| 1.4102 | 0.809795164 |
| 1.41025 | 0.809305306 |
| 1.4103 | 0.808814211 |
| 1.41035 | 0.808321874 |
| 1.4104 | 0.807828293 |
| 1.41045 | 0.807333466 |
| 1.4105 | 0.806837388 |
| 1.41055 | 0.806340057 |
| 1.4106 | 0.80584147 |
| 1.41065 | 0.805341624 |
| 1.4107 | 0.804840516 |
| 1.41075 | 0.804338142 |
| 1.4108 | 0.8038345 |
| 1.41085 | 0.803329586 |
| 1.4109 | 0.802823397 |
| 1.41095 | 0.802315931 |
| 1.411 | 0.801807184 |
| 1.41105 | 0.801297153 |
| 1.4111 | 0.800785835 |
| 1.41115 | 0.800273227 |
| 1.4112 | 0.799759325 |
| 1.41125 | 0.799244127 |
| 1.4113 | 0.798727629 |
| 1.41135 | 0.798209828 |
| 1.4114 | 0.797690721 |
| 1.41145 | 0.797170305 |
| 1.4115 | 0.796648577 |
| 1.41155 | 0.796125533 |
| 1.4116 | 0.79560117 |
| 1.41165 | 0.795075486 |
| 1.4117 | 0.794548476 |
| 1.41175 | 0.794020138 |
| 1.4118 | 0.793490468 |
| 1.41185 | 0.792959464 |
| 1.4119 | 0.792427121 |
| 1.41195 | 0.791893437 |
| 1.412 | 0.791358409 |
| 1.41205 | 0.790822033 |
| 1.4121 | 0.790284305 |
| 1.41215 | 0.789745223 |
| 1.4122 | 0.789204784 |
| 1.41225 | 0.788662984 |
| 1.4123 | 0.788119819 |
| 1.41235 | 0.787575287 |
| 1.4124 | 0.787029385 |
| 1.41245 | 0.786482108 |
| 1.4125 | 0.785933453 |
| 1.41255 | 0.785383418 |
| 1.4126 | 0.784831999 |
| 1.41265 | 0.784279192 |
| 1.4127 | 0.783724994 |
| 1.41275 | 0.783169402 |
| 1.4128 | 0.782612413 |
| 1.41285 | 0.782054022 |
| 1.4129 | 0.781494227 |
| 1.41295 | 0.780933025 |
| 1.413 | 0.780370411 |
| 1.41305 | 0.779806383 |
| 1.4131 | 0.779240937 |
| 1.41315 | 0.77867407 |
| 1.4132 | 0.778105778 |
| 1.41325 | 0.777536057 |
| 1.4133 | 0.776964905 |
| 1.41335 | 0.776392318 |
| 1.4134 | 0.775818293 |
| 1.41345 | 0.775242825 |
| 1.4135 | 0.774665912 |
| 1.41355 | 0.77408755 |
| 1.4136 | 0.773507735 |
| 1.41365 | 0.772926465 |
| 1.4137 | 0.772343735 |
| 1.41375 | 0.771759542 |
| 1.4138 | 0.771173883 |
| 1.41385 | 0.770586753 |
| 1.4139 | 0.76999815 |
| 1.41395 | 0.769408071 |
| 1.414 | 0.76881651 |
| 1.41405 | 0.768223465 |
| 1.4141 | 0.767628933 |
| 1.41415 | 0.767032909 |
| 1.4142 | 0.766435391 |
| 1.41425 | 0.765836374 |
| 1.4143 | 0.765235855 |
| 1.41435 | 0.76463383 |
| 1.4144 | 0.764030296 |
| 1.41445 | 0.763425249 |
| 1.4145 | 0.762818686 |
| 1.41455 | 0.762210603 |
| 1.4146 | 0.761600996 |
| 1.41465 | 0.760989861 |
| 1.4147 | 0.760377196 |
| 1.41475 | 0.759762995 |
| 1.4148 | 0.759147257 |
| 1.41485 | 0.758529976 |
| 1.4149 | 0.75791115 |
| 1.41495 | 0.757290774 |
| 1.415 | 0.756668845 |
| 1.41505 | 0.756045359 |
| 1.4151 | 0.755420312 |
| 1.41515 | 0.754793702 |
| 1.4152 | 0.754165523 |
| 1.41525 | 0.753535773 |
| 1.4153 | 0.752904447 |
| 1.41535 | 0.752271542 |
| 1.4154 | 0.751637054 |
| 1.41545 | 0.751000979 |
| 1.4155 | 0.750363313 |
| 1.41555 | 0.749724054 |
| 1.4156 | 0.749083196 |
| 1.41565 | 0.748440736 |
| 1.4157 | 0.74779667 |
| 1.41575 | 0.747150995 |
| 1.4158 | 0.746503707 |
| 1.41585 | 0.745854801 |
| 1.4159 | 0.745204274 |
| 1.41595 | 0.744552123 |
| 1.416 | 0.743898342 |
| 1.41605 | 0.743242929 |
| 1.4161 | 0.742585879 |
| 1.41615 | 0.74192719 |
| 1.4162 | 0.741266855 |
| 1.41625 | 0.740604873 |
| 1.4163 | 0.739941239 |
| 1.41635 | 0.739275948 |
| 1.4164 | 0.738608998 |
| 1.41645 | 0.737940384 |
| 1.4165 | 0.737270102 |
| 1.41655 | 0.736598148 |
| 1.4166 | 0.735924519 |
| 1.41665 | 0.73524921 |
| 1.4167 | 0.734572218 |
| 1.41675 | 0.733893538 |
| 1.4168 | 0.733213166 |
| 1.41685 | 0.732531099 |
| 1.4169 | 0.731847333 |
| 1.41695 | 0.731161863 |
| 1.417 | 0.730474685 |
| 1.41705 | 0.729785795 |
| 1.4171 | 0.72909519 |
| 1.41715 | 0.728402866 |
| 1.4172 | 0.727708817 |
| 1.41725 | 0.727013041 |
| 1.4173 | 0.726315533 |
| 1.41735 | 0.725616289 |
| 1.4174 | 0.724915305 |
| 1.41745 | 0.724212577 |
| 1.4175 | 0.723508101 |
| 1.41755 | 0.722801873 |
| 1.4176 | 0.722093888 |
| 1.41765 | 0.721384142 |
| 1.4177 | 0.720672632 |
| 1.41775 | 0.719959353 |
| 1.4178 | 0.719244302 |
| 1.41785 | 0.718527473 |
| 1.4179 | 0.717808862 |
| 1.41795 | 0.717088467 |
| 1.418 | 0.716366281 |
| 1.41805 | 0.715642302 |
| 1.4181 | 0.714916525 |
| 1.41815 | 0.714188946 |
| 1.4182 | 0.71345956 |
| 1.41825 | 0.712728364 |
| 1.4183 | 0.711995352 |
| 1.41835 | 0.711260522 |
| 1.4184 | 0.710523868 |
| 1.41845 | 0.709785387 |
| 1.4185 | 0.709045073 |
| 1.41855 | 0.708302924 |
| 1.4186 | 0.707558934 |
| 1.41865 | 0.706813099 |
| 1.4187 | 0.706065415 |
| 1.41875 | 0.705315878 |
| 1.4188 | 0.704564484 |
| 1.41885 | 0.703811227 |
| 1.4189 | 0.703056104 |
| 1.41895 | 0.702299111 |
| 1.419 | 0.701540242 |
| 1.41905 | 0.700779494 |
| 1.4191 | 0.700016863 |
| 1.41915 | 0.699252343 |
| 1.4192 | 0.698485931 |
| 1.41925 | 0.697717622 |
| 1.4193 | 0.696947412 |
| 1.41935 | 0.696175296 |
| 1.4194 | 0.69540127 |
| 1.41945 | 0.69462533 |
| 1.4195 | 0.693847471 |
| 1.41955 | 0.693067688 |
| 1.4196 | 0.692285978 |
| 1.41965 | 0.691502336 |
| 1.4197 | 0.690716756 |
| 1.41975 | 0.689929236 |
| 1.4198 | 0.68913977 |
| 1.41985 | 0.688348354 |
| 1.4199 | 0.687554983 |
| 1.41995 | 0.686759653 |
| 1.42 | 0.685962359 |
| 1.42005 | 0.685163097 |
| 1.4201 | 0.684361863 |
| 1.42015 | 0.683558651 |
| 1.4202 | 0.682753457 |
| 1.42025 | 0.681946277 |
| 1.4203 | 0.681137106 |
| 1.42035 | 0.68032594 |
| 1.4204 | 0.679512774 |
| 1.42045 | 0.678697603 |
| 1.4205 | 0.677880422 |
| 1.42055 | 0.677061228 |
| 1.4206 | 0.676240016 |
| 1.42065 | 0.67541678 |
| 1.4207 | 0.674591516 |
| 1.42075 | 0.673764221 |
| 1.4208 | 0.672934888 |
| 1.42085 | 0.672103513 |
| 1.4209 | 0.671270093 |
| 1.42095 | 0.670434621 |
| 1.421 | 0.669597093 |
| 1.42105 | 0.668757506 |
| 1.4211 | 0.667915853 |
| 1.42115 | 0.66707213 |
| 1.4212 | 0.666226333 |
| 1.42125 | 0.665378457 |
| 1.4213 | 0.664528497 |
| 1.42135 | 0.663676448 |
| 1.4214 | 0.662822306 |
| 1.42145 | 0.661966065 |
| 1.4215 | 0.661107722 |
| 1.42155 | 0.660247271 |
| 1.4216 | 0.659384707 |
| 1.42165 | 0.658520026 |
| 1.4217 | 0.657653223 |
| 1.42175 | 0.656784294 |
| 1.4218 | 0.655913232 |
| 1.42185 | 0.655040034 |
| 1.4219 | 0.654164695 |
| 1.42195 | 0.65328721 |
| 1.422 | 0.652407573 |
| 1.42205 | 0.651525781 |
| 1.4221 | 0.650641828 |
| 1.42215 | 0.64975571 |
| 1.4222 | 0.648867421 |
| 1.42225 | 0.647976956 |
| 1.4223 | 0.647084312 |
| 1.42235 | 0.646189482 |
| 1.4224 | 0.645292462 |
| 1.42245 | 0.644393247 |
| 1.4225 | 0.643491833 |
| 1.42255 | 0.642588213 |
| 1.4226 | 0.641682384 |
| 1.42265 | 0.64077434 |
| 1.4227 | 0.639864076 |
| 1.42275 | 0.638951588 |
| 1.4228 | 0.63803687 |
| 1.42285 | 0.637119917 |
| 1.4229 | 0.636200725 |
| 1.42295 | 0.635279288 |
| 1.423 | 0.634355602 |
| 1.42305 | 0.633429661 |
| 1.4231 | 0.63250146 |
| 1.42315 | 0.631570995 |
| 1.4232 | 0.63063826 |
| 1.42325 | 0.629703251 |
| 1.4233 | 0.628765961 |
| 1.42335 | 0.627826387 |
| 1.4234 | 0.626884522 |
| 1.42345 | 0.625940363 |
| 1.4235 | 0.624993904 |
| 1.42355 | 0.624045139 |
| 1.4236 | 0.623094065 |
| 1.42365 | 0.622140674 |
| 1.4237 | 0.621184964 |
| 1.42375 | 0.620226928 |
| 1.4238 | 0.61926656 |
| 1.42385 | 0.618303858 |
| 1.4239 | 0.617338814 |
| 1.42395 | 0.616371424 |
| 1.424 | 0.615401682 |
| 1.42405 | 0.614429584 |
| 1.4241 | 0.613455125 |
| 1.42415 | 0.612478298 |
| 1.4242 | 0.6114991 |
| 1.42425 | 0.610517524 |
| 1.4243 | 0.609533566 |
| 1.42435 | 0.60854722 |
| 1.4244 | 0.607558481 |
| 1.42445 | 0.606567344 |
| 1.4245 | 0.605573804 |
| 1.42455 | 0.604577855 |
| 1.4246 | 0.603579493 |
| 1.42465 | 0.602578711 |
| 1.4247 | 0.601575505 |
| 1.42475 | 0.600569869 |
| 1.4248 | 0.599561799 |
| 1.42485 | 0.598551288 |
| 1.4249 | 0.597538332 |
| 1.42495 | 0.596522925 |
| 1.425 | 0.595505062 |
| 1.42505 | 0.594484738 |
| 1.4251 | 0.593461947 |
| 1.42515 | 0.592436684 |
| 1.4252 | 0.591408944 |
| 1.42525 | 0.590378721 |
| 1.4253 | 0.58934601 |
| 1.42535 | 0.588310806 |
| 1.4254 | 0.587273102 |
| 1.42545 | 0.586232895 |
| 1.4255 | 0.585190179 |
| 1.42555 | 0.584144947 |
| 1.4256 | 0.583097196 |
| 1.42565 | 0.582046919 |
| 1.4257 | 0.58099411 |
| 1.42575 | 0.579938766 |
| 1.4258 | 0.578880879 |
| 1.42585 | 0.577820446 |
| 1.4259 | 0.57675746 |
| 1.42595 | 0.575691915 |
| 1.426 | 0.574623808 |
| 1.42605 | 0.573553131 |
| 1.4261 | 0.57247988 |
| 1.42615 | 0.571404049 |
| 1.4262 | 0.570325633 |
| 1.42625 | 0.569244626 |
| 1.4263 | 0.568161023 |
| 1.42635 | 0.567074818 |
| 1.4264 | 0.565986007 |
| 1.42645 | 0.564894582 |
| 1.4265 | 0.56380054 |
| 1.42655 | 0.562703873 |
| 1.4266 | 0.561604578 |
| 1.42665 | 0.560502648 |
| 1.4267 | 0.559398078 |
| 1.42675 | 0.558290863 |
| 1.4268 | 0.557180996 |
| 1.42685 | 0.556068473 |
| 1.4269 | 0.554953287 |
| 1.42695 | 0.553835434 |
| 1.427 | 0.552714908 |
| 1.42705 | 0.551591702 |
| 1.4271 | 0.550465813 |
| 1.42715 | 0.549337233 |
| 1.4272 | 0.548205958 |
| 1.42725 | 0.547071982 |
| 1.4273 | 0.5459353 |
| 1.42735 | 0.544795905 |
| 1.4274 | 0.543653793 |
| 1.42745 | 0.542508957 |
| 1.4275 | 0.541361393 |
| 1.42755 | 0.540211094 |
| 1.4276 | 0.539058055 |
| 1.42765 | 0.53790227 |
| 1.4277 | 0.536743734 |
| 1.42775 | 0.535582442 |
| 1.4278 | 0.534418387 |
| 1.42785 | 0.533251564 |
| 1.4279 | 0.532081967 |
| 1.42795 | 0.530909591 |
| 1.428 | 0.529734431 |
| 1.42805 | 0.52855648 |
| 1.4281 | 0.527375733 |
| 1.42815 | 0.526192184 |
| 1.4282 | 0.525005828 |
| 1.42825 | 0.523816658 |
| 1.4283 | 0.522624671 |
| 1.42835 | 0.521429859 |
| 1.4284 | 0.520232217 |
| 1.42845 | 0.51903174 |
| 1.4285 | 0.517828422 |
| 1.42855 | 0.516622257 |
| 1.4286 | 0.51541324 |
| 1.42865 | 0.514201365 |
| 1.4287 | 0.512986626 |
| 1.42875 | 0.511769018 |
| 1.4288 | 0.510548535 |
| 1.42885 | 0.509325171 |
| 1.4289 | 0.508098922 |
| 1.42895 | 0.50686978 |
| 1.429 | 0.505637741 |
| 1.42905 | 0.504402799 |
| 1.4291 | 0.503164948 |
| 1.42915 | 0.501924183 |
| 1.4292 | 0.500680497 |
| 1.42925 | 0.499433886 |
| 1.4293 | 0.498184343 |
| 1.42935 | 0.496931864 |
| 1.4294 | 0.495676442 |
| 1.42945 | 0.494418072 |
| 1.4295 | 0.493156747 |
| 1.42955 | 0.491892464 |
| 1.4296 | 0.490625215 |
| 1.42965 | 0.489354995 |
| 1.4297 | 0.488081799 |
| 1.42975 | 0.48680562 |
| 1.4298 | 0.485526454 |
| 1.42985 | 0.484244295 |
| 1.4299 | 0.482959137 |
| 1.42995 | 0.481670974 |
| 1.43 | 0.480379801 |
| 1.43005 | 0.479085612 |
| 1.4301 | 0.477788402 |
| 1.43015 | 0.476488165 |
| 1.4302 | 0.475184895 |
| 1.43025 | 0.473878587 |
| 1.4303 | 0.472569236 |
| 1.43035 | 0.471256834 |
| 1.4304 | 0.469941378 |
| 1.43045 | 0.468622862 |
| 1.4305 | 0.467301279 |
| 1.43055 | 0.465976624 |
| 1.4306 | 0.464648893 |
| 1.43065 | 0.463318078 |
| 1.4307 | 0.461984175 |
| 1.43075 | 0.460647179 |
| 1.4308 | 0.459307082 |
| 1.43085 | 0.457963881 |
| 1.4309 | 0.45661757 |
| 1.43095 | 0.455268142 |
| 1.431 | 0.453915593 |
| 1.43105 | 0.452559917 |
| 1.4311 | 0.451201109 |
| 1.43115 | 0.449839162 |
| 1.4312 | 0.448474072 |
| 1.43125 | 0.447105834 |
| 1.4313 | 0.44573444 |
| 1.43135 | 0.444359888 |
| 1.4314 | 0.44298217 |
| 1.43145 | 0.441601281 |
| 1.4315 | 0.440217216 |
| 1.43155 | 0.43882997 |
| 1.4316 | 0.437439537 |
| 1.43165 | 0.436045912 |
| 1.4317 | 0.434649089 |
| 1.43175 | 0.433249064 |
| 1.4318 | 0.43184583 |
| 1.43185 | 0.430439383 |
| 1.4319 | 0.429029716 |
| 1.43195 | 0.427616826 |
| 1.432 | 0.426200706 |
| 1.43205 | 0.424781352 |
| 1.4321 | 0.423358758 |
| 1.43215 | 0.421932918 |
| 1.4322 | 0.420503829 |
| 1.43225 | 0.419071483 |
| 1.4323 | 0.417635877 |
| 1.43235 | 0.416197005 |
| 1.4324 | 0.414754862 |
| 1.43245 | 0.413309443 |
| 1.4325 | 0.411860743 |
| 1.43255 | 0.410408756 |
| 1.4326 | 0.408953478 |
| 1.43265 | 0.407494904 |
| 1.4327 | 0.406033028 |
| 1.43275 | 0.404567845 |
| 1.4328 | 0.403099351 |
| 1.43285 | 0.401627541 |
| 1.4329 | 0.40015241 |
| 1.43295 | 0.398673952 |
| 1.433 | 0.397192163 |
| 1.43305 | 0.395707038 |
| 1.4331 | 0.394218573 |
| 1.43315 | 0.392726762 |
| 1.4332 | 0.391231601 |
| 1.43325 | 0.389733084 |
| 1.4333 | 0.388231208 |
| 1.43335 | 0.386725967 |
| 1.4334 | 0.385217358 |
| 1.43345 | 0.383705374 |
| 1.4335 | 0.382190012 |
| 1.43355 | 0.380671267 |
| 1.4336 | 0.379149135 |
| 1.43365 | 0.377623611 |
| 1.4337 | 0.37609469 |
| 1.43375 | 0.374562369 |
| 1.4338 | 0.373026643 |
| 1.43385 | 0.371487507 |
| 1.4339 | 0.369944957 |
| 1.43395 | 0.368398989 |
| 1.434 | 0.366849598 |
| 1.43405 | 0.365296781 |
| 1.4341 | 0.363740534 |
| 1.43415 | 0.362180851 |
| 1.4342 | 0.36061773 |
| 1.43425 | 0.359051166 |
| 1.4343 | 0.357481155 |
| 1.43435 | 0.355907693 |
| 1.4344 | 0.354330777 |
| 1.43445 | 0.352750402 |
| 1.4345 | 0.351166565 |
| 1.43455 | 0.349579262 |
| 1.4346 | 0.347988489 |
| 1.43465 | 0.346394243 |
| 1.4347 | 0.34479652 |
| 1.43475 | 0.343195316 |
| 1.4348 | 0.341590629 |
| 1.43485 | 0.339982455 |
| 1.4349 | 0.33837079 |
| 1.43495 | 0.336755631 |
| 1.435 | 0.335136975 |
| 1.43505 | 0.333514819 |
| 1.4351 | 0.33188916 |
| 1.43515 | 0.330259994 |
| 1.4352 | 0.328627319 |
| 1.43525 | 0.326991132 |
| 1.4353 | 0.32535143 |
| 1.43535 | 0.323708211 |
| 1.4354 | 0.322061471 |
| 1.43545 | 0.320411208 |
| 1.4355 | 0.31875742 |
| 1.43555 | 0.317100104 |
| 1.4356 | 0.315439258 |
| 1.43565 | 0.313774879 |
| 1.4357 | 0.312106966 |
| 1.43575 | 0.310435516 |
| 1.4358 | 0.308760528 |
| 1.43585 | 0.307081999 |
| 1.4359 | 0.305399927 |
| 1.43595 | 0.303714312 |
| 1.436 | 0.302025151 |
| 1.43605 | 0.300332442 |
| 1.4361 | 0.298636185 |
| 1.43615 | 0.296936379 |
| 1.4362 | 0.295233021 |
| 1.43625 | 0.293526111 |
| 1.4363 | 0.291815648 |
| 1.43635 | 0.290101631 |
| 1.4364 | 0.288384059 |
| 1.43645 | 0.286662932 |
| 1.4365 | 0.284938249 |
| 1.43655 | 0.28321001 |
| 1.4366 | 0.281478215 |
| 1.43665 | 0.279742863 |
| 1.4367 | 0.278003955 |
| 1.43675 | 0.276261491 |
| 1.4368 | 0.274515471 |
| 1.43685 | 0.272765896 |
| 1.4369 | 0.271012766 |
| 1.43695 | 0.269256082 |
| 1.437 | 0.267495845 |
| 1.43705 | 0.265732056 |
| 1.4371 | 0.263964717 |
| 1.43715 | 0.262193828 |
| 1.4372 | 0.260419393 |
| 1.43725 | 0.258641411 |
| 1.4373 | 0.256859886 |
| 1.43735 | 0.25507482 |
| 1.4374 | 0.253286214 |
| 1.43745 | 0.251494072 |
| 1.4375 | 0.249698397 |
| 1.43755 | 0.247899191 |
| 1.4376 | 0.246096457 |
| 1.43765 | 0.2442902 |
| 1.4377 | 0.242480422 |
| 1.43775 | 0.240667128 |
| 1.4378 | 0.238850322 |
| 1.43785 | 0.237030008 |
| 1.4379 | 0.235206191 |
| 1.43795 | 0.233378876 |
| 1.438 | 0.231548068 |
| 1.43805 | 0.229713773 |
| 1.4381 | 0.227875996 |
| 1.43815 | 0.226034744 |
| 1.4382 | 0.224190022 |
| 1.43825 | 0.222341837 |
| 1.4383 | 0.220490197 |
| 1.43835 | 0.218635109 |
| 1.4384 | 0.21677658 |
| 1.43845 | 0.214914619 |
| 1.4385 | 0.213049234 |
| 1.43855 | 0.211180433 |
| 1.4386 | 0.209308226 |
| 1.43865 | 0.207432622 |
| 1.4387 | 0.205553631 |
| 1.43875 | 0.203671264 |
| 1.4388 | 0.201785531 |
| 1.43885 | 0.199896444 |
| 1.4389 | 0.198004014 |
| 1.43895 | 0.196108253 |
| 1.439 | 0.194209175 |
| 1.43905 | 0.192306791 |
| 1.4391 | 0.190401117 |
| 1.43915 | 0.188492166 |
| 1.4392 | 0.186579952 |
| 1.43925 | 0.184664492 |
| 1.4393 | 0.182745802 |
| 1.43935 | 0.180823897 |
| 1.4394 | 0.178898795 |
| 1.43945 | 0.176970515 |
| 1.4395 | 0.175039073 |
| 1.43955 | 0.173104491 |
| 1.4396 | 0.171166788 |
| 1.43965 | 0.169225984 |
| 1.4397 | 0.167282101 |
| 1.43975 | 0.165335162 |
| 1.4398 | 0.16338519 |
| 1.43985 | 0.161432209 |
| 1.4399 | 0.159476244 |
| 1.43995 | 0.157517321 |
| 1.44 | 0.155555467 |
| 1.44005 | 0.153590711 |
| 1.4401 | 0.15162308 |
| 1.44015 | 0.149652606 |
| 1.4402 | 0.14767932 |
| 1.44025 | 0.145703254 |
| 1.4403 | 0.143724443 |
| 1.44035 | 0.141742921 |
| 1.4404 | 0.139758726 |
| 1.44045 | 0.137771894 |
| 1.4405 | 0.135782466 |
| 1.44055 | 0.133790483 |
| 1.4406 | 0.131795987 |
| 1.44065 | 0.129799023 |
| 1.4407 | 0.127799637 |
| 1.44075 | 0.125797877 |
| 1.4408 | 0.123793794 |
| 1.44085 | 0.121787439 |
| 1.4409 | 0.119778867 |
| 1.44095 | 0.117768135 |
| 1.441 | 0.115755301 |
| 1.44105 | 0.113740427 |
| 1.4411 | 0.111723577 |
| 1.44115 | 0.109704819 |
| 1.4412 | 0.107684222 |
| 1.44125 | 0.105661859 |
| 1.4413 | 0.103637808 |
| 1.44135 | 0.101612147 |
| 1.4414 | 0.099584961 |
| 1.44145 | 0.097556337 |
| 1.4415 | 0.095526367 |
| 1.44155 | 0.093495147 |
| 1.4416 | 0.091462777 |
| 1.44165 | 0.089429364 |
| 1.4417 | 0.087395019 |
| 1.44175 | 0.085359859 |
| 1.4418 | 0.083324006 |
| 1.44185 | 0.081287591 |
| 1.4419 | 0.079250749 |
| 1.44195 | 0.077213625 |
| 1.442 | 0.075176371 |
| 1.44205 | 0.073139147 |
| 1.4421 | 0.071102123 |
| 1.44215 | 0.06906548 |
| 1.4422 | 0.067029408 |
| 1.44225 | 0.064994111 |
| 1.4423 | 0.062959804 |
| 1.44235 | 0.060926718 |
| 1.4424 | 0.058895097 |
| 1.44245 | 0.056865203 |
| 1.4425 | 0.054837318 |
| 1.44255 | 0.052811742 |
| 1.4426 | 0.050788797 |
| 1.44265 | 0.048768832 |
| 1.4427 | 0.046752223 |
| 1.44275 | 0.044739375 |
| 1.4428 | 0.042730729 |
| 1.44285 | 0.040726764 |
| 1.4429 | 0.038728002 |
| 1.44295 | 0.036735015 |
| 1.443 | 0.034748431 |
| 1.44305 | 0.032768941 |
| 1.4431 | 0.030797312 |
| 1.44315 | 0.028834396 |
| 1.4432 | 0.026881142 |
| 1.44325 | 0.024938621 |
| 1.4433 | 0.023008039 |
| 1.44335 | 0.02109077 |
| 1.4434 | 0.019188387 |
| 1.44345 | 0.017302707 |
| 1.4435 | 0.015435847 |
| 1.44355 | 0.013590294 |
| 1.4436 | 0.011769002 |
| 1.44365 | 0.009975512 |
| 1.4437 | 0.008214097 |
| 1.44375 | 0.006489897 |
| 1.4438 | 0.004808893 |
| 1.44385 | 0.003176902 |
| 1.4439 | 0.001593126 |
| 1.444 | 0 |
| 1.446 | 0.005704331 |
| 1.45 | 0.011328533 |
| 1.454 | 0.015389249 |
| 1.458 | 0.018732861 |
| 1.462 | 0.021630608 |
| 1.466 | 0.024215275 |
| 1.47 | 0.026563987 |
| 1.474 | 0.028726331 |
| 1.478 | 0.030736449 |
| 1.482 | 0.032619056 |
| 1.486 | 0.034392735 |
| 1.49 | 0.036071883 |
| 1.494 | 0.037667931 |
| 1.498 | 0.039190134 |
| 1.502 | 0.040646119 |
| 1.506 | 0.042042258 |
| 1.51 | 0.043383942 |
| 1.514 | 0.044675784 |
| 1.518 | 0.045921764 |
| 1.522 | 0.047125352 |
| 1.526 | 0.048289589 |
| 1.53 | 0.049417163 |
| 1.534 | 0.050510464 |
| 1.538 | 0.051571625 |
| 1.542 | 0.052602565 |
| 1.546 | 0.053605014 |
| 1.55 | 0.054580541 |
| 1.554 | 0.055530571 |
| 1.558 | 0.056456409 |
| 1.562 | 0.057359247 |
| 1.566 | 0.058240182 |
| 1.57 | 0.059100226 |
| 1.574 | 0.059940312 |
| 1.578 | 0.060761307 |
| 1.582 | 0.061564014 |
| 1.586 | 0.062349183 |
| 1.59 | 0.063117511 |
| 1.594 | 0.063869651 |
| 1.598 | 0.064606214 |
| 1.602 | 0.065327774 |
| 1.606 | 0.066034869 |
| 1.61 | 0.066728006 |

**Table 6****.** Simulation results for 2.5-cm sensor over three sensing zones.

| RI @1550 nm | Normalized Power (a.u.) |
| --- | --- |
| 1.3165 | 0.979773325 |
| 1.319 | 0.979188542 |
| 1.3215 | 0.978569628 |
| 1.324 | 0.977913636 |
| 1.3265 | 0.977217268 |
| 1.329 | 0.976476818 |
| 1.3315 | 0.975688106 |
| 1.334 | 0.974846395 |
| 1.3365 | 0.973946286 |
| 1.339 | 0.972981593 |
| 1.3415 | 0.971945179 |
| 1.344 | 0.970828745 |
| 1.3465 | 0.969622553 |
| 1.349 | 0.968315046 |
| 1.3515 | 0.966892323 |
| 1.354 | 0.965337365 |
| 1.3565 | 0.963628857 |
| 1.359 | 0.961739229 |
| 1.3615 | 0.959631123 |
| 1.364 | 0.957249975 |
| 1.3665 | 0.954504587 |
| 1.369 | 0.951186743 |
| 1.37 | 0.949632463 |
| 1.37005 | 0.949542353 |
| 1.3701 | 0.949452054 |
| 1.37015 | 0.949361566 |
| 1.3702 | 0.949270888 |
| 1.37025 | 0.949180019 |
| 1.3703 | 0.94908896 |
| 1.37035 | 0.948997709 |
| 1.3704 | 0.948906267 |
| 1.37045 | 0.948814632 |
| 1.3705 | 0.948722805 |
| 1.37055 | 0.948630785 |
| 1.3706 | 0.948538571 |
| 1.37065 | 0.948446163 |
| 1.3707 | 0.948353561 |
| 1.37075 | 0.948260764 |
| 1.3708 | 0.948167772 |
| 1.37085 | 0.948074584 |
| 1.3709 | 0.947981199 |
| 1.37095 | 0.947887618 |
| 1.371 | 0.94779384 |
| 1.37105 | 0.947699864 |
| 1.3711 | 0.94760569 |
| 1.37115 | 0.947511318 |
| 1.3712 | 0.947416746 |
| 1.37125 | 0.947321975 |
| 1.3713 | 0.947227004 |
| 1.37135 | 0.947131833 |
| 1.3714 | 0.947036461 |
| 1.37145 | 0.946940887 |
| 1.3715 | 0.946845112 |
| 1.37155 | 0.946749134 |
| 1.3716 | 0.946652953 |
| 1.37165 | 0.94655657 |
| 1.3717 | 0.946459982 |
| 1.37175 | 0.94636319 |
| 1.3718 | 0.946266194 |
| 1.37185 | 0.946168992 |
| 1.3719 | 0.946071585 |
| 1.37195 | 0.945973972 |
| 1.372 | 0.945876152 |
| 1.37205 | 0.945778125 |
| 1.3721 | 0.94567989 |
| 1.37215 | 0.945581447 |
| 1.3722 | 0.945482796 |
| 1.37225 | 0.945383936 |
| 1.3723 | 0.945284866 |
| 1.37235 | 0.945185586 |
| 1.3724 | 0.945086096 |
| 1.37245 | 0.944986394 |
| 1.3725 | 0.944886482 |
| 1.37255 | 0.944786357 |
| 1.3726 | 0.944686019 |
| 1.37265 | 0.944585469 |
| 1.3727 | 0.944484705 |
| 1.37275 | 0.944383727 |
| 1.3728 | 0.944282535 |
| 1.37285 | 0.944181128 |
| 1.3729 | 0.944079505 |
| 1.37295 | 0.943977666 |
| 1.373 | 0.943875611 |
| 1.37305 | 0.943773338 |
| 1.3731 | 0.943670848 |
| 1.37315 | 0.943568141 |
| 1.3732 | 0.943465214 |
| 1.37325 | 0.943362069 |
| 1.3733 | 0.943258703 |
| 1.37335 | 0.943155118 |
| 1.3734 | 0.943051312 |
| 1.37345 | 0.942947286 |
| 1.3735 | 0.942843037 |
| 1.37355 | 0.942738566 |
| 1.3736 | 0.942633873 |
| 1.37365 | 0.942528956 |
| 1.3737 | 0.942423816 |
| 1.37375 | 0.942318451 |
| 1.3738 | 0.942212862 |
| 1.37385 | 0.942107048 |
| 1.3739 | 0.942001007 |
| 1.37395 | 0.94189474 |
| 1.374 | 0.941788247 |
| 1.37405 | 0.941681525 |
| 1.3741 | 0.941574576 |
| 1.37415 | 0.941467399 |
| 1.3742 | 0.941359992 |
| 1.37425 | 0.941252356 |
| 1.3743 | 0.94114449 |
| 1.37435 | 0.941036393 |
| 1.3744 | 0.940928065 |
| 1.37445 | 0.940819505 |
| 1.3745 | 0.940710713 |
| 1.37455 | 0.940601688 |
| 1.3746 | 0.94049243 |
| 1.37465 | 0.940382938 |
| 1.3747 | 0.940273211 |
| 1.37475 | 0.94016325 |
| 1.3748 | 0.940053052 |
| 1.37485 | 0.939942619 |
| 1.3749 | 0.939831949 |
| 1.37495 | 0.939721042 |
| 1.375 | 0.939609897 |
| 1.37505 | 0.939498514 |
| 1.3751 | 0.939386892 |
| 1.37515 | 0.939275031 |
| 1.3752 | 0.939162929 |
| 1.37525 | 0.939050587 |
| 1.3753 | 0.938938004 |
| 1.37535 | 0.938825179 |
| 1.3754 | 0.938712112 |
| 1.37545 | 0.938598802 |
| 1.3755 | 0.938485248 |
| 1.37555 | 0.938371451 |
| 1.3756 | 0.938257409 |
| 1.37565 | 0.938143122 |
| 1.3757 | 0.938028589 |
| 1.37575 | 0.93791381 |
| 1.3758 | 0.937798784 |
| 1.37585 | 0.937683511 |
| 1.3759 | 0.937567989 |
| 1.37595 | 0.93745222 |
| 1.376 | 0.937336201 |
| 1.37605 | 0.937219932 |
| 1.3761 | 0.937103413 |
| 1.37615 | 0.936986642 |
| 1.3762 | 0.936869621 |
| 1.37625 | 0.936752347 |
| 1.3763 | 0.936634821 |
| 1.37635 | 0.936517041 |
| 1.3764 | 0.936399007 |
| 1.37645 | 0.936280719 |
| 1.3765 | 0.936162176 |
| 1.37655 | 0.936043377 |
| 1.3766 | 0.935924322 |
| 1.37665 | 0.93580501 |
| 1.3767 | 0.935685441 |
| 1.37675 | 0.935565613 |
| 1.3768 | 0.935445527 |
| 1.37685 | 0.935325182 |
| 1.3769 | 0.935204576 |
| 1.37695 | 0.93508371 |
| 1.377 | 0.934962583 |
| 1.37705 | 0.934841195 |
| 1.3771 | 0.934719544 |
| 1.37715 | 0.934597629 |
| 1.3772 | 0.934475452 |
| 1.37725 | 0.93435301 |
| 1.3773 | 0.934230303 |
| 1.37735 | 0.934107331 |
| 1.3774 | 0.933984093 |
| 1.37745 | 0.933860588 |
| 1.3775 | 0.933736816 |
| 1.37755 | 0.933612776 |
| 1.3776 | 0.933488467 |
| 1.37765 | 0.933363889 |
| 1.3777 | 0.933239042 |
| 1.37775 | 0.933113924 |
| 1.3778 | 0.932988534 |
| 1.37785 | 0.932862873 |
| 1.3779 | 0.93273694 |
| 1.37795 | 0.932610734 |
| 1.378 | 0.932484254 |
| 1.37805 | 0.9323575 |
| 1.3781 | 0.93223047 |
| 1.37815 | 0.932103166 |
| 1.3782 | 0.931975585 |
| 1.37825 | 0.931847727 |
| 1.3783 | 0.931719592 |
| 1.37835 | 0.931591178 |
| 1.3784 | 0.931462486 |
| 1.37845 | 0.931333514 |
| 1.3785 | 0.931204262 |
| 1.37855 | 0.931074729 |
| 1.3786 | 0.930944915 |
| 1.37865 | 0.930814819 |
| 1.3787 | 0.93068444 |
| 1.37875 | 0.930553777 |
| 1.3788 | 0.930422831 |
| 1.37885 | 0.9302916 |
| 1.3789 | 0.930160083 |
| 1.37895 | 0.93002828 |
| 1.379 | 0.92989619 |
| 1.37905 | 0.929763813 |
| 1.3791 | 0.929631148 |
| 1.37915 | 0.929498194 |
| 1.3792 | 0.929364951 |
| 1.37925 | 0.929231418 |
| 1.3793 | 0.929097593 |
| 1.37935 | 0.928963477 |
| 1.3794 | 0.928829069 |
| 1.37945 | 0.928694369 |
| 1.3795 | 0.928559374 |
| 1.37955 | 0.928424085 |
| 1.3796 | 0.928288502 |
| 1.37965 | 0.928152623 |
| 1.3797 | 0.928016447 |
| 1.37975 | 0.927879974 |
| 1.3798 | 0.927743204 |
| 1.37985 | 0.927606135 |
| 1.3799 | 0.927468768 |
| 1.37995 | 0.9273311 |
| 1.38 | 0.927193132 |
| 1.38005 | 0.927054863 |
| 1.3801 | 0.926916291 |
| 1.38015 | 0.926777417 |
| 1.3802 | 0.92663824 |
| 1.38025 | 0.926498759 |
| 1.3803 | 0.926358973 |
| 1.38035 | 0.926218882 |
| 1.3804 | 0.926078484 |
| 1.38045 | 0.925937779 |
| 1.3805 | 0.925796767 |
| 1.38055 | 0.925655447 |
| 1.3806 | 0.925513817 |
| 1.38065 | 0.925371878 |
| 1.3807 | 0.925229628 |
| 1.38075 | 0.925087066 |
| 1.3808 | 0.924944193 |
| 1.38085 | 0.924801007 |
| 1.3809 | 0.924657508 |
| 1.38095 | 0.924513695 |
| 1.381 | 0.924369566 |
| 1.38105 | 0.924225122 |
| 1.3811 | 0.924080362 |
| 1.38115 | 0.923935284 |
| 1.3812 | 0.923789889 |
| 1.38125 | 0.923644175 |
| 1.3813 | 0.923498142 |
| 1.38135 | 0.923351788 |
| 1.3814 | 0.923205114 |
| 1.38145 | 0.923058118 |
| 1.3815 | 0.9229108 |
| 1.38155 | 0.922763158 |
| 1.3816 | 0.922615193 |
| 1.38165 | 0.922466903 |
| 1.3817 | 0.922318288 |
| 1.38175 | 0.922169346 |
| 1.3818 | 0.922020078 |
| 1.38185 | 0.921870482 |
| 1.3819 | 0.921720558 |
| 1.38195 | 0.921570304 |
| 1.382 | 0.92141972 |
| 1.38205 | 0.921268806 |
| 1.3821 | 0.92111756 |
| 1.38215 | 0.920965981 |
| 1.3822 | 0.92081407 |
| 1.38225 | 0.920661825 |
| 1.3823 | 0.920509245 |
| 1.38235 | 0.92035633 |
| 1.3824 | 0.920203078 |
| 1.38245 | 0.92004949 |
| 1.3825 | 0.919895563 |
| 1.38255 | 0.919741298 |
| 1.3826 | 0.919586694 |
| 1.38265 | 0.919431749 |
| 1.3827 | 0.919276464 |
| 1.38275 | 0.919120837 |
| 1.3828 | 0.918964867 |
| 1.38285 | 0.918808554 |
| 1.3829 | 0.918651897 |
| 1.38295 | 0.918494894 |
| 1.383 | 0.918337546 |
| 1.38305 | 0.918179852 |
| 1.3831 | 0.91802181 |
| 1.38315 | 0.91786342 |
| 1.3832 | 0.91770468 |
| 1.38325 | 0.917545591 |
| 1.3833 | 0.917386151 |
| 1.38335 | 0.91722636 |
| 1.3834 | 0.917066217 |
| 1.38345 | 0.91690572 |
| 1.3835 | 0.916744869 |
| 1.38355 | 0.916583664 |
| 1.3836 | 0.916422103 |
| 1.38365 | 0.916260186 |
| 1.3837 | 0.916097911 |
| 1.38375 | 0.915935278 |
| 1.3838 | 0.915772286 |
| 1.38385 | 0.915608935 |
| 1.3839 | 0.915445223 |
| 1.38395 | 0.915281149 |
| 1.384 | 0.915116713 |
| 1.38405 | 0.914951914 |
| 1.3841 | 0.914786751 |
| 1.38415 | 0.914621223 |
| 1.3842 | 0.914455329 |
| 1.38425 | 0.914289069 |
| 1.3843 | 0.914122441 |
| 1.38435 | 0.913955445 |
| 1.3844 | 0.91378808 |
| 1.38445 | 0.913620345 |
| 1.3845 | 0.913452239 |
| 1.38455 | 0.913283761 |
| 1.3846 | 0.913114911 |
| 1.38465 | 0.912945687 |
| 1.3847 | 0.912776089 |
| 1.38475 | 0.912606116 |
| 1.3848 | 0.912435766 |
| 1.38485 | 0.91226504 |
| 1.3849 | 0.912093935 |
| 1.38495 | 0.911922452 |
| 1.385 | 0.91175059 |
| 1.38505 | 0.911578346 |
| 1.3851 | 0.911405722 |
| 1.38515 | 0.911232715 |
| 1.3852 | 0.911059325 |
| 1.38525 | 0.910885551 |
| 1.3853 | 0.910711392 |
| 1.38535 | 0.910536847 |
| 1.3854 | 0.910361915 |
| 1.38545 | 0.910186596 |
| 1.3855 | 0.910010888 |
| 1.38555 | 0.90983479 |
| 1.3856 | 0.909658303 |
| 1.38565 | 0.909481423 |
| 1.3857 | 0.909304152 |
| 1.38575 | 0.909126487 |
| 1.3858 | 0.908948429 |
| 1.38585 | 0.908769975 |
| 1.3859 | 0.908591126 |
| 1.38595 | 0.90841188 |
| 1.386 | 0.908232236 |
| 1.38605 | 0.908052193 |
| 1.3861 | 0.907871751 |
| 1.38615 | 0.907690908 |
| 1.3862 | 0.907509664 |
| 1.38625 | 0.907328017 |
| 1.3863 | 0.907145967 |
| 1.38635 | 0.906963513 |
| 1.3864 | 0.906780654 |
| 1.38645 | 0.906597388 |
| 1.3865 | 0.906413715 |
| 1.38655 | 0.906229634 |
| 1.3866 | 0.906045145 |
| 1.38665 | 0.905860245 |
| 1.3867 | 0.905674934 |
| 1.38675 | 0.905489211 |
| 1.3868 | 0.905303076 |
| 1.38685 | 0.905116527 |
| 1.3869 | 0.904929563 |
| 1.38695 | 0.904742183 |
| 1.387 | 0.904554387 |
| 1.38705 | 0.904366172 |
| 1.3871 | 0.90417754 |
| 1.38715 | 0.903988487 |
| 1.3872 | 0.903799014 |
| 1.38725 | 0.90360912 |
| 1.3873 | 0.903418803 |
| 1.38735 | 0.903228062 |
| 1.3874 | 0.903036897 |
| 1.38745 | 0.902845306 |
| 1.3875 | 0.902653289 |
| 1.38755 | 0.902460844 |
| 1.3876 | 0.902267971 |
| 1.38765 | 0.902074668 |
| 1.3877 | 0.901880935 |
| 1.38775 | 0.90168677 |
| 1.3878 | 0.901492173 |
| 1.38785 | 0.901297143 |
| 1.3879 | 0.901101677 |
| 1.38795 | 0.900905777 |
| 1.388 | 0.900709439 |
| 1.38805 | 0.900512665 |
| 1.3881 | 0.900315451 |
| 1.38815 | 0.900117798 |
| 1.3882 | 0.899919705 |
| 1.38825 | 0.899721169 |
| 1.3883 | 0.899522191 |
| 1.38835 | 0.89932277 |
| 1.3884 | 0.899122903 |
| 1.38845 | 0.898922591 |
| 1.3885 | 0.898721832 |
| 1.38855 | 0.898520626 |
| 1.3886 | 0.89831897 |
| 1.38865 | 0.898116864 |
| 1.3887 | 0.897914308 |
| 1.38875 | 0.897711299 |
| 1.3888 | 0.897507838 |
| 1.38885 | 0.897303922 |
| 1.3889 | 0.897099551 |
| 1.38895 | 0.896894724 |
| 1.389 | 0.89668944 |
| 1.38905 | 0.896483697 |
| 1.3891 | 0.896277495 |
| 1.38915 | 0.896070832 |
| 1.3892 | 0.895863708 |
| 1.38925 | 0.895656121 |
| 1.3893 | 0.89544807 |
| 1.38935 | 0.895239555 |
| 1.3894 | 0.895030573 |
| 1.38945 | 0.894821125 |
| 1.3895 | 0.894611209 |
| 1.38955 | 0.894400824 |
| 1.3896 | 0.894189968 |
| 1.38965 | 0.893978641 |
| 1.3897 | 0.893766842 |
| 1.38975 | 0.89355457 |
| 1.3898 | 0.893341822 |
| 1.38985 | 0.893128599 |
| 1.3899 | 0.8929149 |
| 1.38995 | 0.892700722 |
| 1.39 | 0.892486066 |
| 1.39005 | 0.892270929 |
| 1.3901 | 0.892055311 |
| 1.39015 | 0.891839211 |
| 1.3902 | 0.891622627 |
| 1.39025 | 0.891405559 |
| 1.3903 | 0.891188005 |
| 1.39035 | 0.890969964 |
| 1.3904 | 0.890751435 |
| 1.39045 | 0.890532417 |
| 1.3905 | 0.890312909 |
| 1.39055 | 0.890092909 |
| 1.3906 | 0.889872417 |
| 1.39065 | 0.889651432 |
| 1.3907 | 0.889429951 |
| 1.39075 | 0.889207975 |
| 1.3908 | 0.888985502 |
| 1.39085 | 0.88876253 |
| 1.3909 | 0.888539059 |
| 1.39095 | 0.888315087 |
| 1.391 | 0.888090614 |
| 1.39105 | 0.887865638 |
| 1.3911 | 0.887640158 |
| 1.39115 | 0.887414173 |
| 1.3912 | 0.887187681 |
| 1.39125 | 0.886960682 |
| 1.3913 | 0.886733174 |
| 1.39135 | 0.886505156 |
| 1.3914 | 0.886276627 |
| 1.39145 | 0.886047586 |
| 1.3915 | 0.885818031 |
| 1.39155 | 0.885587962 |
| 1.3916 | 0.885357377 |
| 1.39165 | 0.885126275 |
| 1.3917 | 0.884894655 |
| 1.39175 | 0.884662515 |
| 1.3918 | 0.884429855 |
| 1.39185 | 0.884196673 |
| 1.3919 | 0.883962968 |
| 1.39195 | 0.883728739 |
| 1.392 | 0.883493985 |
| 1.39205 | 0.883258703 |
| 1.3921 | 0.883022894 |
| 1.39215 | 0.882786556 |
| 1.3922 | 0.882549687 |
| 1.39225 | 0.882312287 |
| 1.3923 | 0.882074355 |
| 1.39235 | 0.881835888 |
| 1.3924 | 0.881596886 |
| 1.39245 | 0.881357347 |
| 1.3925 | 0.881117271 |
| 1.39255 | 0.880876656 |
| 1.3926 | 0.880635501 |
| 1.39265 | 0.880393804 |
| 1.3927 | 0.880151565 |
| 1.39275 | 0.879908782 |
| 1.3928 | 0.879665453 |
| 1.39285 | 0.879421578 |
| 1.3929 | 0.879177156 |
| 1.39295 | 0.878932184 |
| 1.393 | 0.878686662 |
| 1.39305 | 0.878440589 |
| 1.3931 | 0.878193963 |
| 1.39315 | 0.877946783 |
| 1.3932 | 0.877699048 |
| 1.39325 | 0.877450756 |
| 1.3933 | 0.877201906 |
| 1.39335 | 0.876952497 |
| 1.3934 | 0.876702527 |
| 1.39345 | 0.876451996 |
| 1.3935 | 0.876200901 |
| 1.39355 | 0.875949243 |
| 1.3936 | 0.875697019 |
| 1.39365 | 0.875444227 |
| 1.3937 | 0.875190868 |
| 1.39375 | 0.874936939 |
| 1.3938 | 0.874682439 |
| 1.39385 | 0.874427367 |
| 1.3939 | 0.874171721 |
| 1.39395 | 0.8739155 |
| 1.394 | 0.873658704 |
| 1.39405 | 0.87340133 |
| 1.3941 | 0.873143377 |
| 1.39415 | 0.872884844 |
| 1.3942 | 0.872625729 |
| 1.39425 | 0.872366032 |
| 1.3943 | 0.872105751 |
| 1.39435 | 0.871844884 |
| 1.3944 | 0.87158343 |
| 1.39445 | 0.871321388 |
| 1.3945 | 0.871058757 |
| 1.39455 | 0.870795534 |
| 1.3946 | 0.87053172 |
| 1.39465 | 0.870267312 |
| 1.3947 | 0.870002308 |
| 1.39475 | 0.869736709 |
| 1.3948 | 0.869470512 |
| 1.39485 | 0.869203715 |
| 1.3949 | 0.868936319 |
| 1.39495 | 0.86866832 |
| 1.395 | 0.868399718 |
| 1.39505 | 0.868130512 |
| 1.3951 | 0.867860699 |
| 1.39515 | 0.867590279 |
| 1.3952 | 0.867319251 |
| 1.39525 | 0.867047612 |
| 1.3953 | 0.866775362 |
| 1.39535 | 0.866502498 |
| 1.3954 | 0.866229021 |
| 1.39545 | 0.865954927 |
| 1.3955 | 0.865680217 |
| 1.39555 | 0.865404887 |
| 1.3956 | 0.865128938 |
| 1.39565 | 0.864852367 |
| 1.3957 | 0.864575174 |
| 1.39575 | 0.864297356 |
| 1.3958 | 0.864018912 |
| 1.39585 | 0.863739841 |
| 1.3959 | 0.863460141 |
| 1.39595 | 0.863179812 |
| 1.396 | 0.862898851 |
| 1.39605 | 0.862617257 |
| 1.3961 | 0.862335028 |
| 1.39615 | 0.862052164 |
| 1.3962 | 0.861768663 |
| 1.39625 | 0.861484522 |
| 1.3963 | 0.861199742 |
| 1.39635 | 0.86091432 |
| 1.3964 | 0.860628254 |
| 1.39645 | 0.860341544 |
| 1.3965 | 0.860054188 |
| 1.39655 | 0.859766185 |
| 1.3966 | 0.859477532 |
| 1.39665 | 0.859188229 |
| 1.3967 | 0.858898274 |
| 1.39675 | 0.858607665 |
| 1.3968 | 0.858316401 |
| 1.39685 | 0.858024481 |
| 1.3969 | 0.857731903 |
| 1.39695 | 0.857438665 |
| 1.397 | 0.857144766 |
| 1.39705 | 0.856850205 |
| 1.3971 | 0.856554979 |
| 1.39715 | 0.856259088 |
| 1.3972 | 0.855962531 |
| 1.39725 | 0.855665304 |
| 1.3973 | 0.855367407 |
| 1.39735 | 0.855068839 |
| 1.3974 | 0.854769597 |
| 1.39745 | 0.854469681 |
| 1.3975 | 0.854169089 |
| 1.39755 | 0.853867819 |
| 1.3976 | 0.853565869 |
| 1.39765 | 0.853263238 |
| 1.3977 | 0.852959925 |
| 1.39775 | 0.852655929 |
| 1.3978 | 0.852351246 |
| 1.39785 | 0.852045876 |
| 1.3979 | 0.851739818 |
| 1.39795 | 0.85143307 |
| 1.398 | 0.851125629 |
| 1.39805 | 0.850817495 |
| 1.3981 | 0.850508667 |
| 1.39815 | 0.850199141 |
| 1.3982 | 0.849888918 |
| 1.39825 | 0.849577995 |
| 1.3983 | 0.84926637 |
| 1.39835 | 0.848954043 |
| 1.3984 | 0.848641011 |
| 1.39845 | 0.848327273 |
| 1.3985 | 0.848012827 |
| 1.39855 | 0.847697672 |
| 1.3986 | 0.847381806 |
| 1.39865 | 0.847065227 |
| 1.3987 | 0.846747935 |
| 1.39875 | 0.846429926 |
| 1.3988 | 0.8461112 |
| 1.39885 | 0.845791755 |
| 1.3989 | 0.845471589 |
| 1.39895 | 0.845150701 |
| 1.399 | 0.844829089 |
| 1.39905 | 0.844506751 |
| 1.3991 | 0.844183686 |
| 1.39915 | 0.843859892 |
| 1.3992 | 0.843535368 |
| 1.39925 | 0.843210111 |
| 1.3993 | 0.842884121 |
| 1.39935 | 0.842557394 |
| 1.3994 | 0.842229931 |
| 1.39945 | 0.841901729 |
| 1.3995 | 0.841572786 |
| 1.39955 | 0.841243101 |
| 1.3996 | 0.840912672 |
| 1.39965 | 0.840581498 |
| 1.3997 | 0.840249576 |
| 1.39975 | 0.839916906 |
| 1.3998 | 0.839583484 |
| 1.39985 | 0.83924931 |
| 1.3999 | 0.838914382 |
| 1.39995 | 0.838578699 |
| 1.4 | 0.838242258 |
| 1.40005 | 0.837905057 |
| 1.4001 | 0.837567096 |
| 1.40015 | 0.837228372 |
| 1.4002 | 0.836888884 |
| 1.40025 | 0.836548629 |
| 1.4003 | 0.836207607 |
| 1.40035 | 0.835865815 |
| 1.4004 | 0.835523252 |
| 1.40045 | 0.835179916 |
| 1.4005 | 0.834835805 |
| 1.40055 | 0.834490918 |
| 1.4006 | 0.834145252 |
| 1.40065 | 0.833798806 |
| 1.4007 | 0.833451578 |
| 1.40075 | 0.833103567 |
| 1.4008 | 0.832754771 |
| 1.40085 | 0.832405187 |
| 1.4009 | 0.832054814 |
| 1.40095 | 0.831703651 |
| 1.401 | 0.831351695 |
| 1.40105 | 0.830998946 |
| 1.4011 | 0.8306454 |
| 1.40115 | 0.830291056 |
| 1.4012 | 0.829935913 |
| 1.40125 | 0.829579968 |
| 1.4013 | 0.82922322 |
| 1.40135 | 0.828865667 |
| 1.4014 | 0.828507307 |
| 1.40145 | 0.828148138 |
| 1.4015 | 0.827788159 |
| 1.40155 | 0.827427368 |
| 1.4016 | 0.827065763 |
| 1.40165 | 0.826703341 |
| 1.4017 | 0.826340102 |
| 1.40175 | 0.825976043 |
| 1.4018 | 0.825611163 |
| 1.40185 | 0.82524546 |
| 1.4019 | 0.824878931 |
| 1.40195 | 0.824511575 |
| 1.402 | 0.82414339 |
| 1.40205 | 0.823774375 |
| 1.4021 | 0.823404527 |
| 1.40215 | 0.823033845 |
| 1.4022 | 0.822662326 |
| 1.40225 | 0.822289969 |
| 1.4023 | 0.821916772 |
| 1.40235 | 0.821542733 |
| 1.4024 | 0.82116785 |
| 1.40245 | 0.820792121 |
| 1.4025 | 0.820415545 |
| 1.40255 | 0.820038119 |
| 1.4026 | 0.819659841 |
| 1.40265 | 0.819280711 |
| 1.4027 | 0.818900725 |
| 1.40275 | 0.818519881 |
| 1.4028 | 0.818138179 |
| 1.40285 | 0.817755616 |
| 1.4029 | 0.81737219 |
| 1.40295 | 0.816987898 |
| 1.403 | 0.81660274 |
| 1.40305 | 0.816216714 |
| 1.4031 | 0.815829816 |
| 1.40315 | 0.815442046 |
| 1.4032 | 0.815053401 |
| 1.40325 | 0.81466388 |
| 1.4033 | 0.81427348 |
| 1.40335 | 0.8138822 |
| 1.4034 | 0.813490038 |
| 1.40345 | 0.813096991 |
| 1.4035 | 0.812703057 |
| 1.40355 | 0.812308235 |
| 1.4036 | 0.811912523 |
| 1.40365 | 0.811515919 |
| 1.4037 | 0.81111842 |
| 1.40375 | 0.810720025 |
| 1.4038 | 0.810320732 |
| 1.40385 | 0.809920538 |
| 1.4039 | 0.809519442 |
| 1.40395 | 0.809117442 |
| 1.404 | 0.808714536 |
| 1.40405 | 0.808310721 |
| 1.4041 | 0.807905995 |
| 1.40415 | 0.807500358 |
| 1.4042 | 0.807093806 |
| 1.40425 | 0.806686337 |
| 1.4043 | 0.80627795 |
| 1.40435 | 0.805868643 |
| 1.4044 | 0.805458413 |
| 1.40445 | 0.805047258 |
| 1.4045 | 0.804635177 |
| 1.40455 | 0.804222166 |
| 1.4046 | 0.803808225 |
| 1.40465 | 0.803393352 |
| 1.4047 | 0.802977543 |
| 1.40475 | 0.802560797 |
| 1.4048 | 0.802143112 |
| 1.40485 | 0.801724486 |
| 1.4049 | 0.801304917 |
| 1.40495 | 0.800884403 |
| 1.405 | 0.800462941 |
| 1.40505 | 0.800040529 |
| 1.4051 | 0.799617166 |
| 1.40515 | 0.799192849 |
| 1.4052 | 0.798767576 |
| 1.40525 | 0.798341346 |
| 1.4053 | 0.797914155 |
| 1.40535 | 0.797486002 |
| 1.4054 | 0.797056885 |
| 1.40545 | 0.796626801 |
| 1.4055 | 0.796195748 |
| 1.40555 | 0.795763725 |
| 1.4056 | 0.795330729 |
| 1.40565 | 0.794896758 |
| 1.4057 | 0.79446181 |
| 1.40575 | 0.794025882 |
| 1.4058 | 0.793588973 |
| 1.40585 | 0.79315108 |
| 1.4059 | 0.792712202 |
| 1.40595 | 0.792272335 |
| 1.406 | 0.791831478 |
| 1.40605 | 0.791389629 |
| 1.4061 | 0.790946785 |
| 1.40615 | 0.790502945 |
| 1.4062 | 0.790058105 |
| 1.40625 | 0.789612265 |
| 1.4063 | 0.789165421 |
| 1.40635 | 0.788717572 |
| 1.4064 | 0.788268714 |
| 1.40645 | 0.787818847 |
| 1.4065 | 0.787367968 |
| 1.40655 | 0.786916074 |
| 1.4066 | 0.786463164 |
| 1.40665 | 0.786009234 |
| 1.4067 | 0.785554284 |
| 1.40675 | 0.78509831 |
| 1.4068 | 0.784641311 |
| 1.40685 | 0.784183283 |
| 1.4069 | 0.783724226 |
| 1.40695 | 0.783264136 |
| 1.407 | 0.782803012 |
| 1.40705 | 0.782340851 |
| 1.4071 | 0.78187765 |
| 1.40715 | 0.781413408 |
| 1.4072 | 0.780948123 |
| 1.40725 | 0.780481791 |
| 1.4073 | 0.780014412 |
| 1.40735 | 0.779545981 |
| 1.4074 | 0.779076498 |
| 1.40745 | 0.778605959 |
| 1.4075 | 0.778134363 |
| 1.40755 | 0.777661708 |
| 1.4076 | 0.77718799 |
| 1.40765 | 0.776713207 |
| 1.4077 | 0.776237358 |
| 1.40775 | 0.77576044 |
| 1.4078 | 0.775282451 |
| 1.40785 | 0.774803387 |
| 1.4079 | 0.774323248 |
| 1.40795 | 0.77384203 |
| 1.408 | 0.773359731 |
| 1.40805 | 0.77287635 |
| 1.4081 | 0.772391882 |
| 1.40815 | 0.771906327 |
| 1.4082 | 0.771419682 |
| 1.40825 | 0.770931944 |
| 1.4083 | 0.770443111 |
| 1.40835 | 0.769953181 |
| 1.4084 | 0.769462151 |
| 1.40845 | 0.768970019 |
| 1.4085 | 0.768476783 |
| 1.40855 | 0.767982439 |
| 1.4086 | 0.767486986 |
| 1.40865 | 0.766990422 |
| 1.4087 | 0.766492743 |
| 1.40875 | 0.765993948 |
| 1.4088 | 0.765494034 |
| 1.40885 | 0.764992998 |
| 1.4089 | 0.764490839 |
| 1.40895 | 0.763987553 |
| 1.409 | 0.763483139 |
| 1.40905 | 0.762977594 |
| 1.4091 | 0.762470915 |
| 1.40915 | 0.7619631 |
| 1.4092 | 0.761454146 |
| 1.40925 | 0.760944052 |
| 1.4093 | 0.760432814 |
| 1.40935 | 0.75992043 |
| 1.4094 | 0.759406898 |
| 1.40945 | 0.758892215 |
| 1.4095 | 0.758376379 |
| 1.40955 | 0.757859387 |
| 1.4096 | 0.757341237 |
| 1.40965 | 0.756821926 |
| 1.4097 | 0.756301452 |
| 1.40975 | 0.755779812 |
| 1.4098 | 0.755257004 |
| 1.40985 | 0.754733025 |
| 1.4099 | 0.754207872 |
| 1.40995 | 0.753681544 |
| 1.41 | 0.753154037 |
| 1.41005 | 0.75262535 |
| 1.4101 | 0.752095479 |
| 1.41015 | 0.751564422 |
| 1.4102 | 0.751032176 |
| 1.41025 | 0.75049874 |
| 1.4103 | 0.74996411 |
| 1.41035 | 0.749428284 |
| 1.4104 | 0.748891259 |
| 1.41045 | 0.748353032 |
| 1.4105 | 0.747813602 |
| 1.41055 | 0.747272966 |
| 1.4106 | 0.74673112 |
| 1.41065 | 0.746188063 |
| 1.4107 | 0.745643791 |
| 1.41075 | 0.745098303 |
| 1.4108 | 0.744551595 |
| 1.41085 | 0.744003666 |
| 1.4109 | 0.743454511 |
| 1.41095 | 0.74290413 |
| 1.411 | 0.742352519 |
| 1.41105 | 0.741799675 |
| 1.4111 | 0.741245596 |
| 1.41115 | 0.74069028 |
| 1.4112 | 0.740133723 |
| 1.41125 | 0.739575923 |
| 1.4113 | 0.739016877 |
| 1.41135 | 0.738456584 |
| 1.4114 | 0.737895039 |
| 1.41145 | 0.737332241 |
| 1.4115 | 0.736768186 |
| 1.41155 | 0.736202873 |
| 1.4116 | 0.735636298 |
| 1.41165 | 0.735068458 |
| 1.4117 | 0.734499352 |
| 1.41175 | 0.733928976 |
| 1.4118 | 0.733357328 |
| 1.41185 | 0.732784405 |
| 1.4119 | 0.732210204 |
| 1.41195 | 0.731634723 |
| 1.412 | 0.731057959 |
| 1.41205 | 0.730479909 |
| 1.4121 | 0.72990057 |
| 1.41215 | 0.72931994 |
| 1.4122 | 0.728738016 |
| 1.41225 | 0.728154796 |
| 1.4123 | 0.727570276 |
| 1.41235 | 0.726984453 |
| 1.4124 | 0.726397326 |
| 1.41245 | 0.725808892 |
| 1.4125 | 0.725219146 |
| 1.41255 | 0.724628088 |
| 1.4126 | 0.724035714 |
| 1.41265 | 0.723442021 |
| 1.4127 | 0.722847006 |
| 1.41275 | 0.722250667 |
| 1.4128 | 0.721653002 |
| 1.41285 | 0.721054006 |
| 1.4129 | 0.720453678 |
| 1.41295 | 0.719852014 |
| 1.413 | 0.719249013 |
| 1.41305 | 0.71864467 |
| 1.4131 | 0.718038983 |
| 1.41315 | 0.71743195 |
| 1.4132 | 0.716823568 |
| 1.41325 | 0.716213833 |
| 1.4133 | 0.715602743 |
| 1.41335 | 0.714990296 |
| 1.4134 | 0.714376487 |
| 1.41345 | 0.713761315 |
| 1.4135 | 0.713144777 |
| 1.41355 | 0.712526869 |
| 1.4136 | 0.71190759 |
| 1.41365 | 0.711286935 |
| 1.4137 | 0.710664903 |
| 1.41375 | 0.71004149 |
| 1.4138 | 0.709416693 |
| 1.41385 | 0.70879051 |
| 1.4139 | 0.708162938 |
| 1.41395 | 0.707533973 |
| 1.414 | 0.706903614 |
| 1.41405 | 0.706271856 |
| 1.4141 | 0.705638698 |
| 1.41415 | 0.705004136 |
| 1.4142 | 0.704368167 |
| 1.41425 | 0.703730789 |
| 1.4143 | 0.703091998 |
| 1.41435 | 0.702451792 |
| 1.4144 | 0.701810168 |
| 1.41445 | 0.701167123 |
| 1.4145 | 0.700522653 |
| 1.41455 | 0.699876756 |
| 1.4146 | 0.699229429 |
| 1.41465 | 0.69858067 |
| 1.4147 | 0.697930474 |
| 1.41475 | 0.697278839 |
| 1.4148 | 0.696625763 |
| 1.41485 | 0.695971242 |
| 1.4149 | 0.695315273 |
| 1.41495 | 0.694657853 |
| 1.415 | 0.69399898 |
| 1.41505 | 0.69333865 |
| 1.4151 | 0.692676861 |
| 1.41515 | 0.692013608 |
| 1.4152 | 0.691348891 |
| 1.41525 | 0.690682704 |
| 1.4153 | 0.690015046 |
| 1.41535 | 0.689345913 |
| 1.4154 | 0.688675303 |
| 1.41545 | 0.688003212 |
| 1.4155 | 0.687329638 |
| 1.41555 | 0.686654576 |
| 1.4156 | 0.685978026 |
| 1.41565 | 0.685299982 |
| 1.4157 | 0.684620443 |
| 1.41575 | 0.683939404 |
| 1.4158 | 0.683256864 |
| 1.41585 | 0.682572819 |
| 1.4159 | 0.681887267 |
| 1.41595 | 0.681200203 |
| 1.416 | 0.680511625 |
| 1.41605 | 0.67982153 |
| 1.4161 | 0.679129915 |
| 1.41615 | 0.678436777 |
| 1.4162 | 0.677742112 |
| 1.41625 | 0.677045918 |
| 1.4163 | 0.676348191 |
| 1.41635 | 0.675648929 |
| 1.4164 | 0.674948129 |
| 1.41645 | 0.674245786 |
| 1.4165 | 0.673541899 |
| 1.41655 | 0.672836464 |
| 1.4166 | 0.672129477 |
| 1.41665 | 0.671420937 |
| 1.4167 | 0.670710839 |
| 1.41675 | 0.669999181 |
| 1.4168 | 0.66928596 |
| 1.41685 | 0.668571171 |
| 1.4169 | 0.667854813 |
| 1.41695 | 0.667136882 |
| 1.417 | 0.666417375 |
| 1.41705 | 0.665696289 |
| 1.4171 | 0.664973621 |
| 1.41715 | 0.664249367 |
| 1.4172 | 0.663523524 |
| 1.41725 | 0.66279609 |
| 1.4173 | 0.66206706 |
| 1.41735 | 0.661336433 |
| 1.4174 | 0.660604204 |
| 1.41745 | 0.659870371 |
| 1.4175 | 0.65913493 |
| 1.41755 | 0.658397879 |
| 1.4176 | 0.657659213 |
| 1.41765 | 0.65691893 |
| 1.4177 | 0.656177027 |
| 1.41775 | 0.6554335 |
| 1.4178 | 0.654688346 |
| 1.41785 | 0.653941563 |
| 1.4179 | 0.653193146 |
| 1.41795 | 0.652443093 |
| 1.418 | 0.6516914 |
| 1.41805 | 0.650938065 |
| 1.4181 | 0.650183083 |
| 1.41815 | 0.649426452 |
| 1.4182 | 0.648668169 |
| 1.41825 | 0.64790823 |
| 1.4183 | 0.647146632 |
| 1.41835 | 0.646383371 |
| 1.4184 | 0.645618446 |
| 1.41845 | 0.644851851 |
| 1.4185 | 0.644083585 |
| 1.41855 | 0.643313643 |
| 1.4186 | 0.642542023 |
| 1.41865 | 0.641768722 |
| 1.4187 | 0.640993735 |
| 1.41875 | 0.64021706 |
| 1.4188 | 0.639438694 |
| 1.41885 | 0.638658633 |
| 1.4189 | 0.637876874 |
| 1.41895 | 0.637093413 |
| 1.419 | 0.636308248 |
| 1.41905 | 0.635521375 |
| 1.4191 | 0.634732791 |
| 1.41915 | 0.633942493 |
| 1.4192 | 0.633150476 |
| 1.41925 | 0.632356739 |
| 1.4193 | 0.631561277 |
| 1.41935 | 0.630764088 |
| 1.4194 | 0.629965167 |
| 1.41945 | 0.629164513 |
| 1.4195 | 0.628362121 |
| 1.41955 | 0.627557988 |
| 1.4196 | 0.62675211 |
| 1.41965 | 0.625944485 |
| 1.4197 | 0.62513511 |
| 1.41975 | 0.62432398 |
| 1.4198 | 0.623511092 |
| 1.41985 | 0.622696444 |
| 1.4199 | 0.621880032 |
| 1.41995 | 0.621061852 |
| 1.42 | 0.620241901 |
| 1.42005 | 0.619420176 |
| 1.4201 | 0.618596674 |
| 1.42015 | 0.61777139 |
| 1.4202 | 0.616944323 |
| 1.42025 | 0.616115468 |
| 1.4203 | 0.615284821 |
| 1.42035 | 0.614452381 |
| 1.4204 | 0.613618143 |
| 1.42045 | 0.612782104 |
| 1.4205 | 0.61194426 |
| 1.42055 | 0.611104609 |
| 1.4206 | 0.610263147 |
| 1.42065 | 0.60941987 |
| 1.4207 | 0.608574775 |
| 1.42075 | 0.607727859 |
| 1.4208 | 0.606879119 |
| 1.42085 | 0.60602855 |
| 1.4209 | 0.60517615 |
| 1.42095 | 0.604321915 |
| 1.421 | 0.603465843 |
| 1.42105 | 0.602607928 |
| 1.4211 | 0.601748169 |
| 1.42115 | 0.600886561 |
| 1.4212 | 0.600023102 |
| 1.42125 | 0.599157788 |
| 1.4213 | 0.598290615 |
| 1.42135 | 0.59742158 |
| 1.4214 | 0.59655068 |
| 1.42145 | 0.595677911 |
| 1.4215 | 0.59480327 |
| 1.42155 | 0.593926754 |
| 1.4216 | 0.593048359 |
| 1.42165 | 0.592168081 |
| 1.4217 | 0.591285918 |
| 1.42175 | 0.590401865 |
| 1.4218 | 0.589515921 |
| 1.42185 | 0.58862808 |
| 1.4219 | 0.587738339 |
| 1.42195 | 0.586846696 |
| 1.422 | 0.585953147 |
| 1.42205 | 0.585057688 |
| 1.4221 | 0.584160317 |
| 1.42215 | 0.583261028 |
| 1.4222 | 0.58235982 |
| 1.42225 | 0.581456689 |
| 1.4223 | 0.580551631 |
| 1.42235 | 0.579644643 |
| 1.4224 | 0.578735721 |
| 1.42245 | 0.577824862 |
| 1.4225 | 0.576912063 |
| 1.42255 | 0.57599732 |
| 1.4226 | 0.57508063 |
| 1.42265 | 0.574161989 |
| 1.4227 | 0.573241394 |
| 1.42275 | 0.572318842 |
| 1.4228 | 0.571394329 |
| 1.42285 | 0.570467851 |
| 1.4229 | 0.569539406 |
| 1.42295 | 0.568608989 |
| 1.423 | 0.567676598 |
| 1.42305 | 0.566742228 |
| 1.4231 | 0.565805878 |
| 1.42315 | 0.564867542 |
| 1.4232 | 0.563927218 |
| 1.42325 | 0.562984902 |
| 1.4233 | 0.562040591 |
| 1.42335 | 0.561094282 |
| 1.4234 | 0.56014597 |
| 1.42345 | 0.559195653 |
| 1.4235 | 0.558243327 |
| 1.42355 | 0.557288989 |
| 1.4236 | 0.556332635 |
| 1.42365 | 0.555374263 |
| 1.4237 | 0.554413867 |
| 1.42375 | 0.553451446 |
| 1.4238 | 0.552486995 |
| 1.42385 | 0.551520512 |
| 1.4239 | 0.550551992 |
| 1.42395 | 0.549581433 |
| 1.424 | 0.54860883 |
| 1.42405 | 0.547634182 |
| 1.4241 | 0.546657483 |
| 1.42415 | 0.545678732 |
| 1.4242 | 0.544697924 |
| 1.42425 | 0.543715055 |
| 1.4243 | 0.542730124 |
| 1.42435 | 0.541743125 |
| 1.4244 | 0.540754056 |
| 1.42445 | 0.539762914 |
| 1.4245 | 0.538769695 |
| 1.42455 | 0.537774395 |
| 1.4246 | 0.536777012 |
| 1.42465 | 0.535777542 |
| 1.4247 | 0.534775981 |
| 1.42475 | 0.533772326 |
| 1.4248 | 0.532766574 |
| 1.42485 | 0.531758721 |
| 1.4249 | 0.530748764 |
| 1.42495 | 0.5297367 |
| 1.425 | 0.528722525 |
| 1.42505 | 0.527706237 |
| 1.4251 | 0.52668783 |
| 1.42515 | 0.525667304 |
| 1.4252 | 0.524644652 |
| 1.42525 | 0.523619874 |
| 1.4253 | 0.522592965 |
| 1.42535 | 0.521563921 |
| 1.4254 | 0.52053274 |
| 1.42545 | 0.519499419 |
| 1.4255 | 0.518463953 |
| 1.42555 | 0.51742634 |
| 1.4256 | 0.516386576 |
| 1.42565 | 0.515344658 |
| 1.4257 | 0.514300583 |
| 1.42575 | 0.513254347 |
| 1.4258 | 0.512205947 |
| 1.42585 | 0.511155381 |
| 1.4259 | 0.510102643 |
| 1.42595 | 0.509047732 |
| 1.426 | 0.507990644 |
| 1.42605 | 0.506931376 |
| 1.4261 | 0.505869924 |
| 1.42615 | 0.504806285 |
| 1.4262 | 0.503740457 |
| 1.42625 | 0.502672435 |
| 1.4263 | 0.501602216 |
| 1.42635 | 0.500529798 |
| 1.4264 | 0.499455177 |
| 1.42645 | 0.49837835 |
| 1.4265 | 0.497299313 |
| 1.42655 | 0.496218064 |
| 1.4266 | 0.495134599 |
| 1.42665 | 0.494048916 |
| 1.4267 | 0.49296101 |
| 1.42675 | 0.491870879 |
| 1.4268 | 0.490778519 |
| 1.42685 | 0.489683928 |
| 1.4269 | 0.488587103 |
| 1.42695 | 0.487488039 |
| 1.427 | 0.486386735 |
| 1.42705 | 0.485283186 |
| 1.4271 | 0.48417739 |
| 1.42715 | 0.483069344 |
| 1.4272 | 0.481959045 |
| 1.42725 | 0.480846489 |
| 1.4273 | 0.479731674 |
| 1.42735 | 0.478614596 |
| 1.4274 | 0.477495253 |
| 1.42745 | 0.476373641 |
| 1.4275 | 0.475249757 |
| 1.42755 | 0.474123599 |
| 1.4276 | 0.472995163 |
| 1.42765 | 0.471864446 |
| 1.4277 | 0.470731446 |
| 1.42775 | 0.469596159 |
| 1.4278 | 0.468458582 |
| 1.42785 | 0.467318713 |
| 1.4279 | 0.466176549 |
| 1.42795 | 0.465032086 |
| 1.428 | 0.463885322 |
| 1.42805 | 0.462736253 |
| 1.4281 | 0.461584878 |
| 1.42815 | 0.460431192 |
| 1.4282 | 0.459275194 |
| 1.42825 | 0.45811688 |
| 1.4283 | 0.456956248 |
| 1.42835 | 0.455793294 |
| 1.4284 | 0.454628017 |
| 1.42845 | 0.453460412 |
| 1.4285 | 0.452290478 |
| 1.42855 | 0.451118211 |
| 1.4286 | 0.44994361 |
| 1.42865 | 0.44876667 |
| 1.4287 | 0.44758739 |
| 1.42875 | 0.446405767 |
| 1.4288 | 0.445221797 |
| 1.42885 | 0.44403548 |
| 1.4289 | 0.44284681 |
| 1.42895 | 0.441655787 |
| 1.429 | 0.440462408 |
| 1.42905 | 0.43926667 |
| 1.4291 | 0.43806857 |
| 1.42915 | 0.436868105 |
| 1.4292 | 0.435665274 |
| 1.42925 | 0.434460074 |
| 1.4293 | 0.433252503 |
| 1.42935 | 0.432042557 |
| 1.4294 | 0.430830234 |
| 1.42945 | 0.429615532 |
| 1.4295 | 0.428398449 |
| 1.42955 | 0.427178982 |
| 1.4296 | 0.425957129 |
| 1.42965 | 0.424732888 |
| 1.4297 | 0.423506255 |
| 1.42975 | 0.422277229 |
| 1.4298 | 0.421045808 |
| 1.42985 | 0.419811989 |
| 1.4299 | 0.41857577 |
| 1.42995 | 0.417337149 |
| 1.43 | 0.416096124 |
| 1.43005 | 0.414852692 |
| 1.4301 | 0.413606851 |
| 1.43015 | 0.4123586 |
| 1.4302 | 0.411107936 |
| 1.43025 | 0.409854857 |
| 1.4303 | 0.408599361 |
| 1.43035 | 0.407341446 |
| 1.4304 | 0.406081111 |
| 1.43045 | 0.404818352 |
| 1.4305 | 0.403553169 |
| 1.43055 | 0.402285559 |
| 1.4306 | 0.401015521 |
| 1.43065 | 0.399743052 |
| 1.4307 | 0.398468152 |
| 1.43075 | 0.397190817 |
| 1.4308 | 0.395911047 |
| 1.43085 | 0.39462884 |
| 1.4309 | 0.393344193 |
| 1.43095 | 0.392057106 |
| 1.431 | 0.390767577 |
| 1.43105 | 0.389475604 |
| 1.4311 | 0.388181186 |
| 1.43115 | 0.386884321 |
| 1.4312 | 0.385585007 |
| 1.43125 | 0.384283244 |
| 1.4313 | 0.38297903 |
| 1.43135 | 0.381672364 |
| 1.4314 | 0.380363243 |
| 1.43145 | 0.379051668 |
| 1.4315 | 0.377737636 |
| 1.43155 | 0.376421147 |
| 1.4316 | 0.375102199 |
| 1.43165 | 0.373780792 |
| 1.4317 | 0.372456923 |
| 1.43175 | 0.371130593 |
| 1.4318 | 0.3698018 |
| 1.43185 | 0.368470544 |
| 1.4319 | 0.367136822 |
| 1.43195 | 0.365800635 |
| 1.432 | 0.364461982 |
| 1.43205 | 0.363120862 |
| 1.4321 | 0.361777273 |
| 1.43215 | 0.360431217 |
| 1.4322 | 0.359082691 |
| 1.43225 | 0.357731695 |
| 1.4323 | 0.35637823 |
| 1.43235 | 0.355022293 |
| 1.4324 | 0.353663886 |
| 1.43245 | 0.352303007 |
| 1.4325 | 0.350939656 |
| 1.43255 | 0.349573834 |
| 1.4326 | 0.348205539 |
| 1.43265 | 0.346834772 |
| 1.4327 | 0.345461532 |
| 1.43275 | 0.34408582 |
| 1.4328 | 0.342707636 |
| 1.43285 | 0.341326979 |
| 1.4329 | 0.33994385 |
| 1.43295 | 0.338558249 |
| 1.433 | 0.337170177 |
| 1.43305 | 0.335779633 |
| 1.4331 | 0.334386618 |
| 1.43315 | 0.332991133 |
| 1.4332 | 0.331593177 |
| 1.43325 | 0.330192753 |
| 1.4333 | 0.32878986 |
| 1.43335 | 0.327384499 |
| 1.4334 | 0.325976671 |
| 1.43345 | 0.324566377 |
| 1.4335 | 0.323153617 |
| 1.43355 | 0.321738394 |
| 1.4336 | 0.320320708 |
| 1.43365 | 0.318900559 |
| 1.4337 | 0.317477951 |
| 1.43375 | 0.316052883 |
| 1.4338 | 0.314625357 |
| 1.43385 | 0.313195375 |
| 1.4339 | 0.311762939 |
| 1.43395 | 0.310328049 |
| 1.434 | 0.308890709 |
| 1.43405 | 0.307450919 |
| 1.4341 | 0.306008682 |
| 1.43415 | 0.304564 |
| 1.4342 | 0.303116875 |
| 1.43425 | 0.301667309 |
| 1.4343 | 0.300215305 |
| 1.43435 | 0.298760864 |
| 1.4344 | 0.29730399 |
| 1.43445 | 0.295844686 |
| 1.4345 | 0.294382953 |
| 1.43455 | 0.292918795 |
| 1.4346 | 0.291452215 |
| 1.43465 | 0.289983216 |
| 1.4347 | 0.2885118 |
| 1.43475 | 0.287037973 |
| 1.4348 | 0.285561735 |
| 1.43485 | 0.284083092 |
| 1.4349 | 0.282602048 |
| 1.43495 | 0.281118604 |
| 1.435 | 0.279632767 |
| 1.43505 | 0.278144539 |
| 1.4351 | 0.276653925 |
| 1.43515 | 0.275160929 |
| 1.4352 | 0.273665555 |
| 1.43525 | 0.272167809 |
| 1.4353 | 0.270667695 |
| 1.43535 | 0.269165217 |
| 1.4354 | 0.267660381 |
| 1.43545 | 0.266153191 |
| 1.4355 | 0.264643654 |
| 1.43555 | 0.263131774 |
| 1.4356 | 0.261617557 |
| 1.43565 | 0.260101009 |
| 1.4357 | 0.258582136 |
| 1.43575 | 0.257060944 |
| 1.4358 | 0.255537439 |
| 1.43585 | 0.254011627 |
| 1.4359 | 0.252483515 |
| 1.43595 | 0.250953111 |
| 1.436 | 0.24942042 |
| 1.43605 | 0.24788545 |
| 1.4361 | 0.246348208 |
| 1.43615 | 0.244808702 |
| 1.4362 | 0.24326694 |
| 1.43625 | 0.241722928 |
| 1.4363 | 0.240176677 |
| 1.43635 | 0.238628193 |
| 1.4364 | 0.237077486 |
| 1.43645 | 0.235524564 |
| 1.4365 | 0.233969436 |
| 1.43655 | 0.232412112 |
| 1.4366 | 0.2308526 |
| 1.43665 | 0.229290911 |
| 1.4367 | 0.227727054 |
| 1.43675 | 0.22616104 |
| 1.4368 | 0.224592878 |
| 1.43685 | 0.223022581 |
| 1.4369 | 0.221450157 |
| 1.43695 | 0.21987562 |
| 1.437 | 0.21829898 |
| 1.43705 | 0.216720249 |
| 1.4371 | 0.215139439 |
| 1.43715 | 0.213556562 |
| 1.4372 | 0.211971632 |
| 1.43725 | 0.21038466 |
| 1.4373 | 0.208795661 |
| 1.43735 | 0.207204647 |
| 1.4374 | 0.205611633 |
| 1.43745 | 0.204016633 |
| 1.4375 | 0.202419662 |
| 1.43755 | 0.200820734 |
| 1.4376 | 0.199219865 |
| 1.43765 | 0.197617071 |
| 1.4377 | 0.196012366 |
| 1.43775 | 0.194405769 |
| 1.4378 | 0.192797296 |
| 1.43785 | 0.191186963 |
| 1.4379 | 0.18957479 |
| 1.43795 | 0.187960793 |
| 1.438 | 0.186344991 |
| 1.43805 | 0.184727403 |
| 1.4381 | 0.183108049 |
| 1.43815 | 0.181486948 |
| 1.4382 | 0.179864122 |
| 1.43825 | 0.17823959 |
| 1.4383 | 0.176613374 |
| 1.43835 | 0.174985496 |
| 1.4384 | 0.173355978 |
| 1.43845 | 0.171724844 |
| 1.4385 | 0.170092117 |
| 1.43855 | 0.168457821 |
| 1.4386 | 0.166821981 |
| 1.43865 | 0.165184622 |
| 1.4387 | 0.163545771 |
| 1.43875 | 0.161905453 |
| 1.4388 | 0.160263696 |
| 1.43885 | 0.158620528 |
| 1.4389 | 0.156975978 |
| 1.43895 | 0.155330075 |
| 1.439 | 0.153682849 |
| 1.43905 | 0.152034332 |
| 1.4391 | 0.150384555 |
| 1.43915 | 0.14873355 |
| 1.4392 | 0.14708135 |
| 1.43925 | 0.145427991 |
| 1.4393 | 0.143773508 |
| 1.43935 | 0.142117935 |
| 1.4394 | 0.140461311 |
| 1.43945 | 0.138803674 |
| 1.4395 | 0.137145061 |
| 1.43955 | 0.135485515 |
| 1.4396 | 0.133825075 |
| 1.43965 | 0.132163783 |
| 1.4397 | 0.130501685 |
| 1.43975 | 0.128838823 |
| 1.4398 | 0.127175244 |
| 1.43985 | 0.125510995 |
| 1.4399 | 0.123846125 |
| 1.43995 | 0.122180683 |
| 1.44 | 0.120514721 |
| 1.44005 | 0.118848292 |
| 1.4401 | 0.117181449 |
| 1.44015 | 0.115514249 |
| 1.4402 | 0.11384675 |
| 1.44025 | 0.11217901 |
| 1.4403 | 0.110511091 |
| 1.44035 | 0.108843056 |
| 1.4404 | 0.107174969 |
| 1.44045 | 0.105506897 |
| 1.4405 | 0.10383891 |
| 1.44055 | 0.102171079 |
| 1.4406 | 0.100503476 |
| 1.44065 | 0.098836178 |
| 1.4407 | 0.097169263 |
| 1.44075 | 0.095502812 |
| 1.4408 | 0.093836908 |
| 1.44085 | 0.092171638 |
| 1.4409 | 0.09050709 |
| 1.44095 | 0.088843359 |
| 1.441 | 0.087180538 |
| 1.44105 | 0.085518727 |
| 1.4411 | 0.083858029 |
| 1.44115 | 0.082198549 |
| 1.4412 | 0.080540399 |
| 1.44125 | 0.078883693 |
| 1.4413 | 0.077228548 |
| 1.44135 | 0.075575089 |
| 1.4414 | 0.073923444 |
| 1.44145 | 0.072273746 |
| 1.4415 | 0.070626133 |
| 1.44155 | 0.06898075 |
| 1.4416 | 0.067337747 |
| 1.44165 | 0.065697281 |
| 1.4417 | 0.064059515 |
| 1.44175 | 0.06242462 |
| 1.4418 | 0.060792775 |
| 1.44185 | 0.059164166 |
| 1.4419 | 0.057538988 |
| 1.44195 | 0.055917445 |
| 1.442 | 0.054299753 |
| 1.44205 | 0.052686136 |
| 1.4421 | 0.051076831 |
| 1.44215 | 0.049472087 |
| 1.4422 | 0.047872166 |
| 1.44225 | 0.046277345 |
| 1.4423 | 0.044687915 |
| 1.44235 | 0.043104184 |
| 1.4424 | 0.041526481 |
| 1.44245 | 0.039955151 |
| 1.4425 | 0.038390562 |
| 1.44255 | 0.036833106 |
| 1.4426 | 0.035283202 |
| 1.44265 | 0.033741294 |
| 1.4427 | 0.032207862 |
| 1.44275 | 0.030683417 |
| 1.4428 | 0.029168511 |
| 1.44285 | 0.027663739 |
| 1.4429 | 0.026169744 |
| 1.44295 | 0.024687225 |
| 1.443 | 0.023216943 |
| 1.44305 | 0.021759728 |
| 1.4431 | 0.020316494 |
| 1.44315 | 0.018888247 |
| 1.4432 | 0.017476103 |
| 1.44325 | 0.016081308 |
| 1.4433 | 0.014705259 |
| 1.44335 | 0.013349538 |
| 1.4434 | 0.012015951 |
| 1.44345 | 0.010706579 |
| 1.4435 | 0.009423857 |
| 1.44355 | 0.00817067 |
| 1.4436 | 0.006950509 |
| 1.44365 | 0.005767697 |
| 1.4437 | 0.004627764 |
| 1.44375 | 0.003538102 |
| 1.4438 | 0.002509202 |
| 1.44385 | 0.001557248 |
| 1.4439 | 0.0007096 |
| 1.444 | 0 |
| 1.446 | 0.001252721 |
| 1.45 | 0.003324612 |
| 1.454 | 0.004762731 |
| 1.458 | 0.005939231 |
| 1.462 | 0.006969339 |
| 1.466 | 0.007901037 |
| 1.47 | 0.008759232 |
| 1.474 | 0.00955896 |
| 1.478 | 0.01031028 |
| 1.482 | 0.011020412 |
| 1.486 | 0.011694809 |
| 1.49 | 0.012337735 |
| 1.494 | 0.012952621 |
| 1.498 | 0.013542283 |
| 1.502 | 0.014109074 |
| 1.506 | 0.014654988 |
| 1.51 | 0.015181732 |
| 1.514 | 0.015690785 |
| 1.518 | 0.016183437 |
| 1.522 | 0.016660824 |
| 1.526 | 0.01712395 |
| 1.53 | 0.017573711 |
| 1.534 | 0.01801091 |
| 1.538 | 0.01843627 |
| 1.542 | 0.018850444 |
| 1.546 | 0.019254025 |
| 1.55 | 0.019647554 |
| 1.554 | 0.020031526 |
| 1.558 | 0.020406396 |
| 1.562 | 0.020772581 |
| 1.566 | 0.021130467 |
| 1.57 | 0.021480412 |
| 1.574 | 0.021822746 |
| 1.578 | 0.022157779 |
| 1.582 | 0.022485798 |
| 1.586 | 0.022807071 |
| 1.59 | 0.02312185 |
| 1.594 | 0.023430369 |
| 1.598 | 0.023732851 |
| 1.602 | 0.024029504 |
| 1.606 | 0.024320523 |
| 1.61 | 0.024606093 |

**Table 7.** Simulation results for 4-cm sensor over three sensing zones.

| RI @1550 nm | Normalized Power (a.u.) |
| --- | --- |
| 1.3165 | 0.969598681 |
| 1.319 | 0.968684601 |
| 1.3215 | 0.967718091 |
| 1.324 | 0.966694728 |
| 1.3265 | 0.965609577 |
| 1.329 | 0.964457112 |
| 1.3315 | 0.963231126 |
| 1.334 | 0.961924611 |
| 1.3365 | 0.960529622 |
| 1.339 | 0.959037101 |
| 1.3415 | 0.957436663 |
| 1.344 | 0.955716313 |
| 1.3465 | 0.953862093 |
| 1.349 | 0.951857591 |
| 1.3515 | 0.949683292 |
| 1.354 | 0.947315638 |
| 1.3565 | 0.944725633 |
| 1.359 | 0.941876632 |
| 1.3615 | 0.938720516 |
| 1.364 | 0.935190206 |
| 1.3665 | 0.93118196 |
| 1.369 | 0.926496967 |
| 1.37 | 0.924451435 |
| 1.37005 | 0.924339309 |
| 1.3701 | 0.924226971 |
| 1.37015 | 0.924114422 |
| 1.3702 | 0.924001661 |
| 1.37025 | 0.923888687 |
| 1.3703 | 0.923775501 |
| 1.37035 | 0.923662101 |
| 1.3704 | 0.923548488 |
| 1.37045 | 0.92343466 |
| 1.3705 | 0.923320618 |
| 1.37055 | 0.92320636 |
| 1.3706 | 0.923091887 |
| 1.37065 | 0.922977198 |
| 1.3707 | 0.922862292 |
| 1.37075 | 0.922747169 |
| 1.3708 | 0.922631829 |
| 1.37085 | 0.92251627 |
| 1.3709 | 0.922400494 |
| 1.37095 | 0.922284498 |
| 1.371 | 0.922168283 |
| 1.37105 | 0.922051848 |
| 1.3711 | 0.921935193 |
| 1.37115 | 0.921818318 |
| 1.3712 | 0.921701221 |
| 1.37125 | 0.921583902 |
| 1.3713 | 0.921466361 |
| 1.37135 | 0.921348597 |
| 1.3714 | 0.921230611 |
| 1.37145 | 0.9211124 |
| 1.3715 | 0.920993966 |
| 1.37155 | 0.920875307 |
| 1.3716 | 0.920756423 |
| 1.37165 | 0.920637313 |
| 1.3717 | 0.920517977 |
| 1.37175 | 0.920398415 |
| 1.3718 | 0.920278626 |
| 1.37185 | 0.920158609 |
| 1.3719 | 0.920038365 |
| 1.37195 | 0.919917892 |
| 1.372 | 0.91979719 |
| 1.37205 | 0.919676258 |
| 1.3721 | 0.919555097 |
| 1.37215 | 0.919433705 |
| 1.3722 | 0.919312082 |
| 1.37225 | 0.919190228 |
| 1.3723 | 0.919068142 |
| 1.37235 | 0.918945823 |
| 1.3724 | 0.918823272 |
| 1.37245 | 0.918700487 |
| 1.3725 | 0.918577468 |
| 1.37255 | 0.918454215 |
| 1.3726 | 0.918330727 |
| 1.37265 | 0.918207003 |
| 1.3727 | 0.918083043 |
| 1.37275 | 0.917958847 |
| 1.3728 | 0.917834415 |
| 1.37285 | 0.917709744 |
| 1.3729 | 0.917584836 |
| 1.37295 | 0.917459689 |
| 1.373 | 0.917334303 |
| 1.37305 | 0.917208678 |
| 1.3731 | 0.917082813 |
| 1.37315 | 0.916956707 |
| 1.3732 | 0.91683036 |
| 1.37325 | 0.916703772 |
| 1.3733 | 0.916576941 |
| 1.37335 | 0.916449868 |
| 1.3734 | 0.916322552 |
| 1.37345 | 0.916194992 |
| 1.3735 | 0.916067188 |
| 1.37355 | 0.915939139 |
| 1.3736 | 0.915810845 |
| 1.37365 | 0.915682305 |
| 1.3737 | 0.915553519 |
| 1.37375 | 0.915424486 |
| 1.3738 | 0.915295206 |
| 1.37385 | 0.915165678 |
| 1.3739 | 0.915035901 |
| 1.37395 | 0.914905876 |
| 1.374 | 0.914775601 |
| 1.37405 | 0.914645076 |
| 1.3741 | 0.914514301 |
| 1.37415 | 0.914383274 |
| 1.3742 | 0.914251996 |
| 1.37425 | 0.914120466 |
| 1.3743 | 0.913988683 |
| 1.37435 | 0.913856647 |
| 1.3744 | 0.913724357 |
| 1.37445 | 0.913591812 |
| 1.3745 | 0.913459013 |
| 1.37455 | 0.913325958 |
| 1.3746 | 0.913192648 |
| 1.37465 | 0.91305908 |
| 1.3747 | 0.912925256 |
| 1.37475 | 0.912791174 |
| 1.3748 | 0.912656834 |
| 1.37485 | 0.912522235 |
| 1.3749 | 0.912387377 |
| 1.37495 | 0.912252259 |
| 1.375 | 0.912116881 |
| 1.37505 | 0.911981241 |
| 1.3751 | 0.91184534 |
| 1.37515 | 0.911709177 |
| 1.3752 | 0.911572752 |
| 1.37525 | 0.911436063 |
| 1.3753 | 0.91129911 |
| 1.37535 | 0.911161893 |
| 1.3754 | 0.911024411 |
| 1.37545 | 0.910886663 |
| 1.3755 | 0.91074865 |
| 1.37555 | 0.91061037 |
| 1.3756 | 0.910471822 |
| 1.37565 | 0.910333007 |
| 1.3757 | 0.910193923 |
| 1.37575 | 0.910054571 |
| 1.3758 | 0.909914949 |
| 1.37585 | 0.909775057 |
| 1.3759 | 0.909634894 |
| 1.37595 | 0.90949446 |
| 1.376 | 0.909353754 |
| 1.37605 | 0.909212775 |
| 1.3761 | 0.909071524 |
| 1.37615 | 0.908929999 |
| 1.3762 | 0.9087882 |
| 1.37625 | 0.908646126 |
| 1.3763 | 0.908503777 |
| 1.37635 | 0.908361151 |
| 1.3764 | 0.908218249 |
| 1.37645 | 0.90807507 |
| 1.3765 | 0.907931614 |
| 1.37655 | 0.907787879 |
| 1.3766 | 0.907643864 |
| 1.37665 | 0.907499571 |
| 1.3767 | 0.907354997 |
| 1.37675 | 0.907210143 |
| 1.3768 | 0.907065007 |
| 1.37685 | 0.906919589 |
| 1.3769 | 0.906773889 |
| 1.37695 | 0.906627905 |
| 1.377 | 0.906481638 |
| 1.37705 | 0.906335086 |
| 1.3771 | 0.906188249 |
| 1.37715 | 0.906041127 |
| 1.3772 | 0.905893718 |
| 1.37725 | 0.905746023 |
| 1.3773 | 0.90559804 |
| 1.37735 | 0.905449769 |
| 1.3774 | 0.90530121 |
| 1.37745 | 0.905152361 |
| 1.3775 | 0.905003222 |
| 1.37755 | 0.904853792 |
| 1.3776 | 0.904704072 |
| 1.37765 | 0.904554059 |
| 1.3777 | 0.904403754 |
| 1.37775 | 0.904253156 |
| 1.3778 | 0.904102265 |
| 1.37785 | 0.903951078 |
| 1.3779 | 0.903799597 |
| 1.37795 | 0.903647821 |
| 1.378 | 0.903495748 |
| 1.37805 | 0.903343378 |
| 1.3781 | 0.903190711 |
| 1.37815 | 0.903037746 |
| 1.3782 | 0.902884481 |
| 1.37825 | 0.902730918 |
| 1.3783 | 0.902577054 |
| 1.37835 | 0.90242289 |
| 1.3784 | 0.902268424 |
| 1.37845 | 0.902113656 |
| 1.3785 | 0.901958585 |
| 1.37855 | 0.901803212 |
| 1.3786 | 0.901647534 |
| 1.37865 | 0.901491551 |
| 1.3787 | 0.901335264 |
| 1.37875 | 0.90117867 |
| 1.3788 | 0.90102177 |
| 1.37885 | 0.900864562 |
| 1.3789 | 0.900707047 |
| 1.37895 | 0.900549223 |
| 1.379 | 0.900391089 |
| 1.37905 | 0.900232646 |
| 1.3791 | 0.900073893 |
| 1.37915 | 0.899914828 |
| 1.3792 | 0.899755451 |
| 1.37925 | 0.899595761 |
| 1.3793 | 0.899435758 |
| 1.37935 | 0.899275442 |
| 1.3794 | 0.89911481 |
| 1.37945 | 0.898953864 |
| 1.3795 | 0.898792601 |
| 1.37955 | 0.898631022 |
| 1.3796 | 0.898469126 |
| 1.37965 | 0.898306911 |
| 1.3797 | 0.898144378 |
| 1.37975 | 0.897981526 |
| 1.3798 | 0.897818353 |
| 1.37985 | 0.89765486 |
| 1.3799 | 0.897491045 |
| 1.37995 | 0.897326908 |
| 1.38 | 0.897162448 |
| 1.38005 | 0.896997665 |
| 1.3801 | 0.896832558 |
| 1.38015 | 0.896667125 |
| 1.3802 | 0.896501368 |
| 1.38025 | 0.896335283 |
| 1.3803 | 0.896168872 |
| 1.38035 | 0.896002133 |
| 1.3804 | 0.895835066 |
| 1.38045 | 0.895667669 |
| 1.3805 | 0.895499943 |
| 1.38055 | 0.895331886 |
| 1.3806 | 0.895163498 |
| 1.38065 | 0.894994778 |
| 1.3807 | 0.894825726 |
| 1.38075 | 0.894656339 |
| 1.3808 | 0.894486619 |
| 1.38085 | 0.894316564 |
| 1.3809 | 0.894146174 |
| 1.38095 | 0.893975447 |
| 1.381 | 0.893804383 |
| 1.38105 | 0.893632982 |
| 1.3811 | 0.893461242 |
| 1.38115 | 0.893289163 |
| 1.3812 | 0.893116744 |
| 1.38125 | 0.892943984 |
| 1.3813 | 0.892770884 |
| 1.38135 | 0.892597441 |
| 1.3814 | 0.892423655 |
| 1.38145 | 0.892249525 |
| 1.3815 | 0.892075052 |
| 1.38155 | 0.891900233 |
| 1.3816 | 0.891725069 |
| 1.38165 | 0.891549558 |
| 1.3817 | 0.8913737 |
| 1.38175 | 0.891197493 |
| 1.3818 | 0.891020938 |
| 1.38185 | 0.890844034 |
| 1.3819 | 0.890666779 |
| 1.38195 | 0.890489174 |
| 1.382 | 0.890311216 |
| 1.38205 | 0.890132906 |
| 1.3821 | 0.889954243 |
| 1.38215 | 0.889775226 |
| 1.3822 | 0.889595854 |
| 1.38225 | 0.889416126 |
| 1.3823 | 0.889236042 |
| 1.38235 | 0.889055601 |
| 1.3824 | 0.888874802 |
| 1.38245 | 0.888693645 |
| 1.3825 | 0.888512128 |
| 1.38255 | 0.888330251 |
| 1.3826 | 0.888148013 |
| 1.38265 | 0.887965413 |
| 1.3827 | 0.887782451 |
| 1.38275 | 0.887599126 |
| 1.3828 | 0.887415437 |
| 1.38285 | 0.887231382 |
| 1.3829 | 0.887046963 |
| 1.38295 | 0.886862176 |
| 1.383 | 0.886677023 |
| 1.38305 | 0.886491502 |
| 1.3831 | 0.886305612 |
| 1.38315 | 0.886119352 |
| 1.3832 | 0.885932722 |
| 1.38325 | 0.885745721 |
| 1.3833 | 0.885558348 |
| 1.38335 | 0.885370602 |
| 1.3834 | 0.885182483 |
| 1.38345 | 0.88499399 |
| 1.3835 | 0.884805121 |
| 1.38355 | 0.884615876 |
| 1.3836 | 0.884426255 |
| 1.38365 | 0.884236256 |
| 1.3837 | 0.884045879 |
| 1.38375 | 0.883855123 |
| 1.3838 | 0.883663986 |
| 1.38385 | 0.883472469 |
| 1.3839 | 0.88328057 |
| 1.38395 | 0.883088289 |
| 1.384 | 0.882895625 |
| 1.38405 | 0.882702576 |
| 1.3841 | 0.882509143 |
| 1.38415 | 0.882315324 |
| 1.3842 | 0.882121118 |
| 1.38425 | 0.881926525 |
| 1.3843 | 0.881731544 |
| 1.38435 | 0.881536173 |
| 1.3844 | 0.881340413 |
| 1.38445 | 0.881144262 |
| 1.3845 | 0.88094772 |
| 1.38455 | 0.880750785 |
| 1.3846 | 0.880553457 |
| 1.38465 | 0.880355735 |
| 1.3847 | 0.880157618 |
| 1.38475 | 0.879959106 |
| 1.3848 | 0.879760196 |
| 1.38485 | 0.87956089 |
| 1.3849 | 0.879361185 |
| 1.38495 | 0.87916108 |
| 1.385 | 0.878960576 |
| 1.38505 | 0.878759671 |
| 1.3851 | 0.878558364 |
| 1.38515 | 0.878356655 |
| 1.3852 | 0.878154542 |
| 1.38525 | 0.877952025 |
| 1.3853 | 0.877749103 |
| 1.38535 | 0.877545774 |
| 1.3854 | 0.877342039 |
| 1.38545 | 0.877137896 |
| 1.3855 | 0.876933344 |
| 1.38555 | 0.876728383 |
| 1.3856 | 0.876523011 |
| 1.38565 | 0.876317228 |
| 1.3857 | 0.876111033 |
| 1.38575 | 0.875904425 |
| 1.3858 | 0.875697403 |
| 1.38585 | 0.875489966 |
| 1.3859 | 0.875282113 |
| 1.38595 | 0.875073844 |
| 1.386 | 0.874865158 |
| 1.38605 | 0.874656053 |
| 1.3861 | 0.874446528 |
| 1.38615 | 0.874236584 |
| 1.3862 | 0.874026218 |
| 1.38625 | 0.873815431 |
| 1.3863 | 0.87360422 |
| 1.38635 | 0.873392586 |
| 1.3864 | 0.873180527 |
| 1.38645 | 0.872968043 |
| 1.3865 | 0.872755132 |
| 1.38655 | 0.872541793 |
| 1.3866 | 0.872328027 |
| 1.38665 | 0.872113831 |
| 1.3867 | 0.871899205 |
| 1.38675 | 0.871684148 |
| 1.3868 | 0.871468659 |
| 1.38685 | 0.871252736 |
| 1.3869 | 0.871036381 |
| 1.38695 | 0.87081959 |
| 1.387 | 0.870602364 |
| 1.38705 | 0.870384701 |
| 1.3871 | 0.8701666 |
| 1.38715 | 0.869948061 |
| 1.3872 | 0.869729083 |
| 1.38725 | 0.869509664 |
| 1.3873 | 0.869289804 |
| 1.38735 | 0.869069501 |
| 1.3874 | 0.868848755 |
| 1.38745 | 0.868627566 |
| 1.3875 | 0.868405931 |
| 1.38755 | 0.86818385 |
| 1.3876 | 0.867961322 |
| 1.38765 | 0.867738346 |
| 1.3877 | 0.867514922 |
| 1.38775 | 0.867291047 |
| 1.3878 | 0.867066722 |
| 1.38785 | 0.866841944 |
| 1.3879 | 0.866616715 |
| 1.38795 | 0.866391031 |
| 1.388 | 0.866164893 |
| 1.38805 | 0.865938299 |
| 1.3881 | 0.865711248 |
| 1.38815 | 0.86548374 |
| 1.3882 | 0.865255773 |
| 1.38825 | 0.865027347 |
| 1.3883 | 0.86479846 |
| 1.38835 | 0.864569112 |
| 1.3884 | 0.864339301 |
| 1.38845 | 0.864109027 |
| 1.3885 | 0.863878288 |
| 1.38855 | 0.863647084 |
| 1.3886 | 0.863415413 |
| 1.38865 | 0.863183275 |
| 1.3887 | 0.862950669 |
| 1.38875 | 0.862717593 |
| 1.3888 | 0.862484046 |
| 1.38885 | 0.862250028 |
| 1.3889 | 0.862015538 |
| 1.38895 | 0.861780575 |
| 1.389 | 0.861545136 |
| 1.38905 | 0.861309223 |
| 1.3891 | 0.861072833 |
| 1.38915 | 0.860835965 |
| 1.3892 | 0.860598619 |
| 1.38925 | 0.860360794 |
| 1.3893 | 0.860122488 |
| 1.38935 | 0.8598837 |
| 1.3894 | 0.85964443 |
| 1.38945 | 0.859404676 |
| 1.3895 | 0.859164437 |
| 1.38955 | 0.858923713 |
| 1.3896 | 0.858682502 |
| 1.38965 | 0.858440804 |
| 1.3897 | 0.858198616 |
| 1.38975 | 0.857955939 |
| 1.3898 | 0.857712771 |
| 1.38985 | 0.857469111 |
| 1.3899 | 0.857224958 |
| 1.38995 | 0.856980311 |
| 1.39 | 0.856735169 |
| 1.39005 | 0.856489531 |
| 1.3901 | 0.856243396 |
| 1.39015 | 0.855996763 |
| 1.3902 | 0.85574963 |
| 1.39025 | 0.855501997 |
| 1.3903 | 0.855253863 |
| 1.39035 | 0.855005226 |
| 1.3904 | 0.854756086 |
| 1.39045 | 0.854506441 |
| 1.3905 | 0.85425629 |
| 1.39055 | 0.854005633 |
| 1.3906 | 0.853754468 |
| 1.39065 | 0.853502794 |
| 1.3907 | 0.85325061 |
| 1.39075 | 0.852997915 |
| 1.3908 | 0.852744708 |
| 1.39085 | 0.852490988 |
| 1.3909 | 0.852236753 |
| 1.39095 | 0.851982003 |
| 1.391 | 0.851726737 |
| 1.39105 | 0.851470953 |
| 1.3911 | 0.85121465 |
| 1.39115 | 0.850957828 |
| 1.3912 | 0.850700484 |
| 1.39125 | 0.850442619 |
| 1.3913 | 0.85018423 |
| 1.39135 | 0.849925318 |
| 1.3914 | 0.84966588 |
| 1.39145 | 0.849405916 |
| 1.3915 | 0.849145424 |
| 1.39155 | 0.848884403 |
| 1.3916 | 0.848622853 |
| 1.39165 | 0.848360772 |
| 1.3917 | 0.848098159 |
| 1.39175 | 0.847835013 |
| 1.3918 | 0.847571332 |
| 1.39185 | 0.847307116 |
| 1.3919 | 0.847042364 |
| 1.39195 | 0.846777074 |
| 1.392 | 0.846511245 |
| 1.39205 | 0.846244876 |
| 1.3921 | 0.845977966 |
| 1.39215 | 0.845710514 |
| 1.3922 | 0.845442518 |
| 1.39225 | 0.845173978 |
| 1.3923 | 0.844904893 |
| 1.39235 | 0.84463526 |
| 1.3924 | 0.84436508 |
| 1.39245 | 0.84409435 |
| 1.3925 | 0.84382307 |
| 1.39255 | 0.843551239 |
| 1.3926 | 0.843278855 |
| 1.39265 | 0.843005917 |
| 1.3927 | 0.842732424 |
| 1.39275 | 0.842458375 |
| 1.3928 | 0.842183769 |
| 1.39285 | 0.841908604 |
| 1.3929 | 0.84163288 |
| 1.39295 | 0.841356595 |
| 1.393 | 0.841079748 |
| 1.39305 | 0.840802338 |
| 1.3931 | 0.840524363 |
| 1.39315 | 0.840245823 |
| 1.3932 | 0.839966716 |
| 1.39325 | 0.83968704 |
| 1.3933 | 0.839406796 |
| 1.39335 | 0.839125982 |
| 1.3934 | 0.838844596 |
| 1.39345 | 0.838562637 |
| 1.3935 | 0.838280104 |
| 1.39355 | 0.837996996 |
| 1.3936 | 0.837713311 |
| 1.39365 | 0.837429049 |
| 1.3937 | 0.837144208 |
| 1.39375 | 0.836858787 |
| 1.3938 | 0.836572785 |
| 1.39385 | 0.8362862 |
| 1.3939 | 0.835999032 |
| 1.39395 | 0.835711279 |
| 1.394 | 0.835422939 |
| 1.39405 | 0.835134012 |
| 1.3941 | 0.834844497 |
| 1.39415 | 0.834554391 |
| 1.3942 | 0.834263695 |
| 1.39425 | 0.833972406 |
| 1.3943 | 0.833680523 |
| 1.39435 | 0.833388046 |
| 1.3944 | 0.833094973 |
| 1.39445 | 0.832801302 |
| 1.3945 | 0.832507032 |
| 1.39455 | 0.832212163 |
| 1.3946 | 0.831916693 |
| 1.39465 | 0.83162062 |
| 1.3947 | 0.831323944 |
| 1.39475 | 0.831026662 |
| 1.3948 | 0.830728775 |
| 1.39485 | 0.83043028 |
| 1.3949 | 0.830131176 |
| 1.39495 | 0.829831462 |
| 1.395 | 0.829531137 |
| 1.39505 | 0.8292302 |
| 1.3951 | 0.828928648 |
| 1.39515 | 0.828626482 |
| 1.3952 | 0.828323699 |
| 1.39525 | 0.828020298 |
| 1.3953 | 0.827716279 |
| 1.39535 | 0.827411639 |
| 1.3954 | 0.827106377 |
| 1.39545 | 0.826800493 |
| 1.3955 | 0.826493984 |
| 1.39555 | 0.82618685 |
| 1.3956 | 0.825879089 |
| 1.39565 | 0.825570699 |
| 1.3957 | 0.825261681 |
| 1.39575 | 0.824952031 |
| 1.3958 | 0.82464175 |
| 1.39585 | 0.824330835 |
| 1.3959 | 0.824019285 |
| 1.39595 | 0.8237071 |
| 1.396 | 0.823394277 |
| 1.39605 | 0.823080815 |
| 1.3961 | 0.822766713 |
| 1.39615 | 0.82245197 |
| 1.3962 | 0.822136584 |
| 1.39625 | 0.821820554 |
| 1.3963 | 0.821503878 |
| 1.39635 | 0.821186556 |
| 1.3964 | 0.820868586 |
| 1.39645 | 0.820549966 |
| 1.3965 | 0.820230696 |
| 1.39655 | 0.819910773 |
| 1.3966 | 0.819590196 |
| 1.39665 | 0.819268965 |
| 1.3967 | 0.818947078 |
| 1.39675 | 0.818624533 |
| 1.3968 | 0.818301329 |
| 1.39685 | 0.817977464 |
| 1.3969 | 0.817652938 |
| 1.39695 | 0.817327749 |
| 1.397 | 0.817001895 |
| 1.39705 | 0.816675375 |
| 1.3971 | 0.816348188 |
| 1.39715 | 0.816020333 |
| 1.3972 | 0.815691807 |
| 1.39725 | 0.81536261 |
| 1.3973 | 0.81503274 |
| 1.39735 | 0.814702196 |
| 1.3974 | 0.814370976 |
| 1.39745 | 0.814039079 |
| 1.3975 | 0.813706503 |
| 1.39755 | 0.813373248 |
| 1.3976 | 0.813039312 |
| 1.39765 | 0.812704693 |
| 1.3977 | 0.812369389 |
| 1.39775 | 0.8120334 |
| 1.3978 | 0.811696725 |
| 1.39785 | 0.81135936 |
| 1.3979 | 0.811021307 |
| 1.39795 | 0.810682561 |
| 1.398 | 0.810343124 |
| 1.39805 | 0.810002992 |
| 1.3981 | 0.809662164 |
| 1.39815 | 0.80932064 |
| 1.3982 | 0.808978417 |
| 1.39825 | 0.808635494 |
| 1.3983 | 0.80829187 |
| 1.39835 | 0.807947543 |
| 1.3984 | 0.807602512 |
| 1.39845 | 0.807256775 |
| 1.3985 | 0.806910331 |
| 1.39855 | 0.806563179 |
| 1.3986 | 0.806215316 |
| 1.39865 | 0.805866742 |
| 1.3987 | 0.805517455 |
| 1.39875 | 0.805167453 |
| 1.3988 | 0.804816736 |
| 1.39885 | 0.804465301 |
| 1.3989 | 0.804113147 |
| 1.39895 | 0.803760272 |
| 1.399 | 0.803406676 |
| 1.39905 | 0.803052356 |
| 1.3991 | 0.802697312 |
| 1.39915 | 0.802341541 |
| 1.3992 | 0.801985042 |
| 1.39925 | 0.801627814 |
| 1.3993 | 0.801269855 |
| 1.39935 | 0.800911163 |
| 1.3994 | 0.800551738 |
| 1.39945 | 0.800191577 |
| 1.3995 | 0.79983068 |
| 1.39955 | 0.799469044 |
| 1.3996 | 0.799106668 |
| 1.39965 | 0.79874355 |
| 1.3997 | 0.79837969 |
| 1.39975 | 0.798015085 |
| 1.3998 | 0.797649734 |
| 1.39985 | 0.797283635 |
| 1.3999 | 0.796916787 |
| 1.39995 | 0.796549188 |
| 1.4 | 0.796180838 |
| 1.40005 | 0.795811733 |
| 1.4001 | 0.795441873 |
| 1.40015 | 0.795071257 |
| 1.4002 | 0.794699882 |
| 1.40025 | 0.794327747 |
| 1.4003 | 0.79395485 |
| 1.40035 | 0.79358119 |
| 1.4004 | 0.793206766 |
| 1.40045 | 0.792831575 |
| 1.4005 | 0.792455617 |
| 1.40055 | 0.792078889 |
| 1.4006 | 0.791701391 |
| 1.40065 | 0.791323119 |
| 1.4007 | 0.790944074 |
| 1.40075 | 0.790564253 |
| 1.4008 | 0.790183655 |
| 1.40085 | 0.789802278 |
| 1.4009 | 0.78942012 |
| 1.40095 | 0.78903718 |
| 1.401 | 0.788653457 |
| 1.40105 | 0.788268948 |
| 1.4011 | 0.787883653 |
| 1.40115 | 0.787497569 |
| 1.4012 | 0.787110695 |
| 1.40125 | 0.786723029 |
| 1.4013 | 0.78633457 |
| 1.40135 | 0.785945316 |
| 1.4014 | 0.785555266 |
| 1.40145 | 0.785164417 |
| 1.4015 | 0.784772769 |
| 1.40155 | 0.784380319 |
| 1.4016 | 0.783987066 |
| 1.40165 | 0.783593008 |
| 1.4017 | 0.783198144 |
| 1.40175 | 0.782802472 |
| 1.4018 | 0.78240599 |
| 1.40185 | 0.782008697 |
| 1.4019 | 0.781610592 |
| 1.40195 | 0.781211671 |
| 1.402 | 0.780811935 |
| 1.40205 | 0.78041138 |
| 1.4021 | 0.780010006 |
| 1.40215 | 0.779607811 |
| 1.4022 | 0.779204793 |
| 1.40225 | 0.77880095 |
| 1.4023 | 0.778396281 |
| 1.40235 | 0.777990784 |
| 1.4024 | 0.777584457 |
| 1.40245 | 0.7771773 |
| 1.4025 | 0.776769309 |
| 1.40255 | 0.776360484 |
| 1.4026 | 0.775950822 |
| 1.40265 | 0.775540322 |
| 1.4027 | 0.775128983 |
| 1.40275 | 0.774716802 |
| 1.4028 | 0.774303778 |
| 1.40285 | 0.773889909 |
| 1.4029 | 0.773475194 |
| 1.40295 | 0.77305963 |
| 1.403 | 0.772643217 |
| 1.40305 | 0.772225952 |
| 1.4031 | 0.771807833 |
| 1.40315 | 0.771388859 |
| 1.4032 | 0.770969029 |
| 1.40325 | 0.770548339 |
| 1.4033 | 0.77012679 |
| 1.40335 | 0.769704378 |
| 1.4034 | 0.769281103 |
| 1.40345 | 0.768856962 |
| 1.4035 | 0.768431954 |
| 1.40355 | 0.768006077 |
| 1.4036 | 0.767579329 |
| 1.40365 | 0.767151709 |
| 1.4037 | 0.766723214 |
| 1.40375 | 0.766293843 |
| 1.4038 | 0.765863595 |
| 1.40385 | 0.765432466 |
| 1.4039 | 0.765000457 |
| 1.40395 | 0.764567565 |
| 1.404 | 0.764133787 |
| 1.40405 | 0.763699123 |
| 1.4041 | 0.763263571 |
| 1.40415 | 0.762827128 |
| 1.4042 | 0.762389793 |
| 1.40425 | 0.761951565 |
| 1.4043 | 0.761512441 |
| 1.40435 | 0.76107242 |
| 1.4044 | 0.760631499 |
| 1.40445 | 0.760189678 |
| 1.4045 | 0.759746954 |
| 1.40455 | 0.759303325 |
| 1.4046 | 0.75885879 |
| 1.40465 | 0.758413346 |
| 1.4047 | 0.757966993 |
| 1.40475 | 0.757519728 |
| 1.4048 | 0.757071549 |
| 1.40485 | 0.756622455 |
| 1.4049 | 0.756172443 |
| 1.40495 | 0.755721512 |
| 1.405 | 0.755269661 |
| 1.40505 | 0.754816886 |
| 1.4051 | 0.754363187 |
| 1.40515 | 0.753908562 |
| 1.4052 | 0.753453008 |
| 1.40525 | 0.752996524 |
| 1.4053 | 0.752539108 |
| 1.40535 | 0.752080758 |
| 1.4054 | 0.751621472 |
| 1.40545 | 0.751161249 |
| 1.4055 | 0.750700087 |
| 1.40555 | 0.750237983 |
| 1.4056 | 0.749774936 |
| 1.40565 | 0.749310943 |
| 1.4057 | 0.748846004 |
| 1.40575 | 0.748380116 |
| 1.4058 | 0.747913278 |
| 1.40585 | 0.747445487 |
| 1.4059 | 0.746976741 |
| 1.40595 | 0.746507039 |
| 1.406 | 0.746036379 |
| 1.40605 | 0.745564759 |
| 1.4061 | 0.745092177 |
| 1.40615 | 0.744618631 |
| 1.4062 | 0.744144119 |
| 1.40625 | 0.743668639 |
| 1.4063 | 0.74319219 |
| 1.40635 | 0.742714769 |
| 1.4064 | 0.742236374 |
| 1.40645 | 0.741757004 |
| 1.4065 | 0.741276657 |
| 1.40655 | 0.740795331 |
| 1.4066 | 0.740313023 |
| 1.40665 | 0.739829733 |
| 1.4067 | 0.739345457 |
| 1.40675 | 0.738860195 |
| 1.4068 | 0.738373943 |
| 1.40685 | 0.737886701 |
| 1.4069 | 0.737398466 |
| 1.40695 | 0.736909236 |
| 1.407 | 0.736419009 |
| 1.40705 | 0.735927784 |
| 1.4071 | 0.735435558 |
| 1.40715 | 0.73494233 |
| 1.4072 | 0.734448097 |
| 1.40725 | 0.733952857 |
| 1.4073 | 0.733456609 |
| 1.40735 | 0.732959351 |
| 1.4074 | 0.73246108 |
| 1.40745 | 0.731961795 |
| 1.4075 | 0.731461493 |
| 1.40755 | 0.730960173 |
| 1.4076 | 0.730457833 |
| 1.40765 | 0.729954471 |
| 1.4077 | 0.729450084 |
| 1.40775 | 0.728944671 |
| 1.4078 | 0.728438229 |
| 1.40785 | 0.727930757 |
| 1.4079 | 0.727422253 |
| 1.40795 | 0.726912714 |
| 1.408 | 0.726402139 |
| 1.40805 | 0.725890526 |
| 1.4081 | 0.725377872 |
| 1.40815 | 0.724864176 |
| 1.4082 | 0.724349436 |
| 1.40825 | 0.723833649 |
| 1.4083 | 0.723316814 |
| 1.40835 | 0.722798928 |
| 1.4084 | 0.722279989 |
| 1.40845 | 0.721759996 |
| 1.4085 | 0.721238947 |
| 1.40855 | 0.720716838 |
| 1.4086 | 0.720193669 |
| 1.40865 | 0.719669437 |
| 1.4087 | 0.719144141 |
| 1.40875 | 0.718617777 |
| 1.4088 | 0.718090345 |
| 1.40885 | 0.717561842 |
| 1.4089 | 0.717032265 |
| 1.40895 | 0.716501614 |
| 1.409 | 0.715969885 |
| 1.40905 | 0.715437077 |
| 1.4091 | 0.714903188 |
| 1.40915 | 0.714368215 |
| 1.4092 | 0.713832157 |
| 1.40925 | 0.713295011 |
| 1.4093 | 0.712756776 |
| 1.40935 | 0.712217448 |
| 1.4094 | 0.711677027 |
| 1.40945 | 0.711135511 |
| 1.4095 | 0.710592896 |
| 1.40955 | 0.71004918 |
| 1.4096 | 0.709504363 |
| 1.40965 | 0.708958441 |
| 1.4097 | 0.708411413 |
| 1.40975 | 0.707863277 |
| 1.4098 | 0.707314029 |
| 1.40985 | 0.706763669 |
| 1.4099 | 0.706212194 |
| 1.40995 | 0.705659602 |
| 1.41 | 0.705105891 |
| 1.41005 | 0.704551058 |
| 1.4101 | 0.703995102 |
| 1.41015 | 0.70343802 |
| 1.4102 | 0.702879811 |
| 1.41025 | 0.702320472 |
| 1.4103 | 0.701760001 |
| 1.41035 | 0.701198396 |
| 1.4104 | 0.700635654 |
| 1.41045 | 0.700071774 |
| 1.4105 | 0.699506754 |
| 1.41055 | 0.69894059 |
| 1.4106 | 0.698373282 |
| 1.41065 | 0.697804827 |
| 1.4107 | 0.697235222 |
| 1.41075 | 0.696664466 |
| 1.4108 | 0.696092557 |
| 1.41085 | 0.695519492 |
| 1.4109 | 0.694945269 |
| 1.41095 | 0.694369885 |
| 1.411 | 0.69379334 |
| 1.41105 | 0.69321563 |
| 1.4111 | 0.692636753 |
| 1.41115 | 0.692056708 |
| 1.4112 | 0.691475492 |
| 1.41125 | 0.690893102 |
| 1.4113 | 0.690309537 |
| 1.41135 | 0.689724794 |
| 1.4114 | 0.689138871 |
| 1.41145 | 0.688551767 |
| 1.4115 | 0.687963478 |
| 1.41155 | 0.687374003 |
| 1.4116 | 0.686783339 |
| 1.41165 | 0.686191484 |
| 1.4117 | 0.685598436 |
| 1.41175 | 0.685004193 |
| 1.4118 | 0.684408752 |
| 1.41185 | 0.683812111 |
| 1.4119 | 0.683214268 |
| 1.41195 | 0.682615222 |
| 1.412 | 0.682014968 |
| 1.41205 | 0.681413506 |
| 1.4121 | 0.680810833 |
| 1.41215 | 0.680206947 |
| 1.4122 | 0.679601846 |
| 1.41225 | 0.678995526 |
| 1.4123 | 0.678387987 |
| 1.41235 | 0.677779226 |
| 1.4124 | 0.67716924 |
| 1.41245 | 0.676558027 |
| 1.4125 | 0.675945586 |
| 1.41255 | 0.675331913 |
| 1.4126 | 0.674717007 |
| 1.41265 | 0.674100865 |
| 1.4127 | 0.673483485 |
| 1.41275 | 0.672864865 |
| 1.4128 | 0.672245002 |
| 1.41285 | 0.671623895 |
| 1.4129 | 0.67100154 |
| 1.41295 | 0.670377936 |
| 1.413 | 0.66975308 |
| 1.41305 | 0.66912697 |
| 1.4131 | 0.668499604 |
| 1.41315 | 0.66787098 |
| 1.4132 | 0.667241094 |
| 1.41325 | 0.666609946 |
| 1.4133 | 0.665977532 |
| 1.41335 | 0.66534385 |
| 1.4134 | 0.664708898 |
| 1.41345 | 0.664072674 |
| 1.4135 | 0.663435176 |
| 1.41355 | 0.6627964 |
| 1.4136 | 0.662156345 |
| 1.41365 | 0.661515008 |
| 1.4137 | 0.660872388 |
| 1.41375 | 0.660228481 |
| 1.4138 | 0.659583286 |
| 1.41385 | 0.6589368 |
| 1.4139 | 0.658289021 |
| 1.41395 | 0.657639946 |
| 1.414 | 0.656989573 |
| 1.41405 | 0.656337901 |
| 1.4141 | 0.655684925 |
| 1.41415 | 0.655030645 |
| 1.4142 | 0.654375058 |
| 1.41425 | 0.653718161 |
| 1.4143 | 0.653059953 |
| 1.41435 | 0.65240043 |
| 1.4144 | 0.65173959 |
| 1.41445 | 0.651077432 |
| 1.4145 | 0.650413953 |
| 1.41455 | 0.64974915 |
| 1.4146 | 0.649083021 |
| 1.41465 | 0.648415563 |
| 1.4147 | 0.647746775 |
| 1.41475 | 0.647076654 |
| 1.4148 | 0.646405198 |
| 1.41485 | 0.645732404 |
| 1.4149 | 0.645058269 |
| 1.41495 | 0.644382793 |
| 1.415 | 0.643705971 |
| 1.41505 | 0.643027802 |
| 1.4151 | 0.642348284 |
| 1.41515 | 0.641667413 |
| 1.4152 | 0.640985189 |
| 1.41525 | 0.640301607 |
| 1.4153 | 0.639616667 |
| 1.41535 | 0.638930365 |
| 1.4154 | 0.638242699 |
| 1.41545 | 0.637553667 |
| 1.4155 | 0.636863267 |
| 1.41555 | 0.636171495 |
| 1.4156 | 0.63547835 |
| 1.41565 | 0.634783829 |
| 1.4157 | 0.63408793 |
| 1.41575 | 0.63339065 |
| 1.4158 | 0.632691988 |
| 1.41585 | 0.63199194 |
| 1.4159 | 0.631290504 |
| 1.41595 | 0.630587678 |
| 1.416 | 0.62988346 |
| 1.41605 | 0.629177846 |
| 1.4161 | 0.628470835 |
| 1.41615 | 0.627762425 |
| 1.4162 | 0.627052612 |
| 1.41625 | 0.626341395 |
| 1.4163 | 0.62562877 |
| 1.41635 | 0.624914736 |
| 1.4164 | 0.624199291 |
| 1.41645 | 0.623482431 |
| 1.4165 | 0.622764154 |
| 1.41655 | 0.622044459 |
| 1.4166 | 0.621323341 |
| 1.41665 | 0.6206008 |
| 1.4167 | 0.619876833 |
| 1.41675 | 0.619151437 |
| 1.4168 | 0.61842461 |
| 1.41685 | 0.617696349 |
| 1.4169 | 0.616966652 |
| 1.41695 | 0.616235517 |
| 1.417 | 0.61550294 |
| 1.41705 | 0.614768921 |
| 1.4171 | 0.614033456 |
| 1.41715 | 0.613296542 |
| 1.4172 | 0.612558178 |
| 1.41725 | 0.611818361 |
| 1.4173 | 0.611077089 |
| 1.41735 | 0.610334359 |
| 1.4174 | 0.609590168 |
| 1.41745 | 0.608844515 |
| 1.4175 | 0.608097396 |
| 1.41755 | 0.60734881 |
| 1.4176 | 0.606598754 |
| 1.41765 | 0.605847225 |
| 1.4177 | 0.605094222 |
| 1.41775 | 0.604339741 |
| 1.4178 | 0.60358378 |
| 1.41785 | 0.602826337 |
| 1.4179 | 0.60206741 |
| 1.41795 | 0.601306995 |
| 1.418 | 0.600545091 |
| 1.41805 | 0.599781694 |
| 1.4181 | 0.599016804 |
| 1.41815 | 0.598250416 |
| 1.4182 | 0.597482529 |
| 1.41825 | 0.596713141 |
| 1.4183 | 0.595942248 |
| 1.41835 | 0.595169848 |
| 1.4184 | 0.59439594 |
| 1.41845 | 0.59362052 |
| 1.4185 | 0.592843586 |
| 1.41855 | 0.592065135 |
| 1.4186 | 0.591285166 |
| 1.41865 | 0.590503676 |
| 1.4187 | 0.589720661 |
| 1.41875 | 0.588936121 |
| 1.4188 | 0.588150052 |
| 1.41885 | 0.587362452 |
| 1.4189 | 0.586573318 |
| 1.41895 | 0.585782649 |
| 1.419 | 0.584990441 |
| 1.41905 | 0.584196692 |
| 1.4191 | 0.5834014 |
| 1.41915 | 0.582604562 |
| 1.4192 | 0.581806176 |
| 1.41925 | 0.58100624 |
| 1.4193 | 0.580204751 |
| 1.41935 | 0.579401706 |
| 1.4194 | 0.578597103 |
| 1.41945 | 0.57779094 |
| 1.4195 | 0.576983214 |
| 1.41955 | 0.576173923 |
| 1.4196 | 0.575363064 |
| 1.41965 | 0.574550636 |
| 1.4197 | 0.573736634 |
| 1.41975 | 0.572921058 |
| 1.4198 | 0.572103905 |
| 1.41985 | 0.571285171 |
| 1.4199 | 0.570464856 |
| 1.41995 | 0.569642956 |
| 1.42 | 0.568819468 |
| 1.42005 | 0.567994392 |
| 1.4201 | 0.567167723 |
| 1.42015 | 0.56633946 |
| 1.4202 | 0.5655096 |
| 1.42025 | 0.564678141 |
| 1.4203 | 0.563845081 |
| 1.42035 | 0.563010416 |
| 1.4204 | 0.562174145 |
| 1.42045 | 0.561336265 |
| 1.4205 | 0.560496774 |
| 1.42055 | 0.559655669 |
| 1.4206 | 0.558812948 |
| 1.42065 | 0.557968609 |
| 1.4207 | 0.557122649 |
| 1.42075 | 0.556275065 |
| 1.4208 | 0.555425856 |
| 1.42085 | 0.554575019 |
| 1.4209 | 0.553722551 |
| 1.42095 | 0.55286845 |
| 1.421 | 0.552012714 |
| 1.42105 | 0.551155341 |
| 1.4211 | 0.550296327 |
| 1.42115 | 0.549435671 |
| 1.4212 | 0.54857337 |
| 1.42125 | 0.547709422 |
| 1.4213 | 0.546843824 |
| 1.42135 | 0.545976574 |
| 1.4214 | 0.54510767 |
| 1.42145 | 0.54423711 |
| 1.4215 | 0.54336489 |
| 1.42155 | 0.542491009 |
| 1.4216 | 0.541615464 |
| 1.42165 | 0.540738252 |
| 1.4217 | 0.539859373 |
| 1.42175 | 0.538978822 |
| 1.4218 | 0.538096598 |
| 1.42185 | 0.537212699 |
| 1.4219 | 0.536327122 |
| 1.42195 | 0.535439864 |
| 1.422 | 0.534550924 |
| 1.42205 | 0.5336603 |
| 1.4221 | 0.532767987 |
| 1.42215 | 0.531873986 |
| 1.4222 | 0.530978292 |
| 1.42225 | 0.530080905 |
| 1.4223 | 0.52918182 |
| 1.42235 | 0.528281037 |
| 1.4224 | 0.527378553 |
| 1.42245 | 0.526474365 |
| 1.4225 | 0.525568472 |
| 1.42255 | 0.524660871 |
| 1.4226 | 0.523751559 |
| 1.42265 | 0.522840535 |
| 1.4227 | 0.521927796 |
| 1.42275 | 0.52101334 |
| 1.4228 | 0.520097164 |
| 1.42285 | 0.519179267 |
| 1.4229 | 0.518259646 |
| 1.42295 | 0.517338299 |
| 1.423 | 0.516415223 |
| 1.42305 | 0.515490417 |
| 1.4231 | 0.514563877 |
| 1.42315 | 0.513635603 |
| 1.4232 | 0.512705591 |
| 1.42325 | 0.51177384 |
| 1.4233 | 0.510840347 |
| 1.42335 | 0.509905109 |
| 1.4234 | 0.508968126 |
| 1.42345 | 0.508029394 |
| 1.4235 | 0.507088912 |
| 1.42355 | 0.506146676 |
| 1.4236 | 0.505202686 |
| 1.42365 | 0.504256939 |
| 1.4237 | 0.503309432 |
| 1.42375 | 0.502360164 |
| 1.4238 | 0.501409132 |
| 1.42385 | 0.500456334 |
| 1.4239 | 0.499501769 |
| 1.42395 | 0.498545433 |
| 1.424 | 0.497587325 |
| 1.42405 | 0.496627443 |
| 1.4241 | 0.495665785 |
| 1.42415 | 0.494702347 |
| 1.4242 | 0.49373713 |
| 1.42425 | 0.492770129 |
| 1.4243 | 0.491801344 |
| 1.42435 | 0.490830772 |
| 1.4244 | 0.489858411 |
| 1.42445 | 0.488884259 |
| 1.4245 | 0.487908314 |
| 1.42455 | 0.486930573 |
| 1.4246 | 0.485951036 |
| 1.42465 | 0.4849697 |
| 1.4247 | 0.483986562 |
| 1.42475 | 0.483001621 |
| 1.4248 | 0.482014875 |
| 1.42485 | 0.481026322 |
| 1.4249 | 0.480035959 |
| 1.42495 | 0.479043786 |
| 1.425 | 0.478049799 |
| 1.42505 | 0.477053998 |
| 1.4251 | 0.476056379 |
| 1.42515 | 0.475056942 |
| 1.4252 | 0.474055683 |
| 1.42525 | 0.473052602 |
| 1.4253 | 0.472047697 |
| 1.42535 | 0.471040964 |
| 1.4254 | 0.470032404 |
| 1.42545 | 0.469022013 |
| 1.4255 | 0.468009789 |
| 1.42555 | 0.466995732 |
| 1.4256 | 0.465979839 |
| 1.42565 | 0.464962109 |
| 1.4257 | 0.463942539 |
| 1.42575 | 0.462921127 |
| 1.4258 | 0.461897873 |
| 1.42585 | 0.460872773 |
| 1.4259 | 0.459845828 |
| 1.42595 | 0.458817033 |
| 1.426 | 0.457786389 |
| 1.42605 | 0.456753893 |
| 1.4261 | 0.455719543 |
| 1.42615 | 0.454683338 |
| 1.4262 | 0.453645276 |
| 1.42625 | 0.452605355 |
| 1.4263 | 0.451563574 |
| 1.42635 | 0.450519931 |
| 1.4264 | 0.449474425 |
| 1.42645 | 0.448427053 |
| 1.4265 | 0.447377815 |
| 1.42655 | 0.446326708 |
| 1.4266 | 0.445273731 |
| 1.42665 | 0.444218883 |
| 1.4267 | 0.443162161 |
| 1.42675 | 0.442103565 |
| 1.4268 | 0.441043093 |
| 1.42685 | 0.439980743 |
| 1.4269 | 0.438916514 |
| 1.42695 | 0.437850405 |
| 1.427 | 0.436782413 |
| 1.42705 | 0.435712538 |
| 1.4271 | 0.434640778 |
| 1.42715 | 0.433567132 |
| 1.4272 | 0.432491598 |
| 1.42725 | 0.431414175 |
| 1.4273 | 0.430334861 |
| 1.42735 | 0.429253656 |
| 1.4274 | 0.428170558 |
| 1.42745 | 0.427085565 |
| 1.4275 | 0.425998677 |
| 1.42755 | 0.424909891 |
| 1.4276 | 0.423819208 |
| 1.42765 | 0.422726625 |
| 1.4277 | 0.421632142 |
| 1.42775 | 0.420535757 |
| 1.4278 | 0.419437469 |
| 1.42785 | 0.418337277 |
| 1.4279 | 0.41723518 |
| 1.42795 | 0.416131177 |
| 1.428 | 0.415025266 |
| 1.42805 | 0.413917447 |
| 1.4281 | 0.412807718 |
| 1.42815 | 0.411696079 |
| 1.4282 | 0.410582528 |
| 1.42825 | 0.409467065 |
| 1.4283 | 0.408349689 |
| 1.42835 | 0.407230398 |
| 1.4284 | 0.406109193 |
| 1.42845 | 0.404986071 |
| 1.4285 | 0.403861032 |
| 1.42855 | 0.402734075 |
| 1.4286 | 0.4016052 |
| 1.42865 | 0.400474406 |
| 1.4287 | 0.399341691 |
| 1.42875 | 0.398207056 |
| 1.4288 | 0.397070499 |
| 1.42885 | 0.39593202 |
| 1.4289 | 0.394791618 |
| 1.42895 | 0.393649293 |
| 1.429 | 0.392505044 |
| 1.42905 | 0.39135887 |
| 1.4291 | 0.39021077 |
| 1.42915 | 0.389060745 |
| 1.4292 | 0.387908794 |
| 1.42925 | 0.386754917 |
| 1.4293 | 0.385599112 |
| 1.42935 | 0.38444138 |
| 1.4294 | 0.38328172 |
| 1.42945 | 0.382120132 |
| 1.4295 | 0.380956616 |
| 1.42955 | 0.37979117 |
| 1.4296 | 0.378623796 |
| 1.42965 | 0.377454493 |
| 1.4297 | 0.37628326 |
| 1.42975 | 0.375110098 |
| 1.4298 | 0.373935007 |
| 1.42985 | 0.372757985 |
| 1.4299 | 0.371579034 |
| 1.42995 | 0.370398154 |
| 1.43 | 0.369215343 |
| 1.43005 | 0.368030603 |
| 1.4301 | 0.366843934 |
| 1.43015 | 0.365655335 |
| 1.4302 | 0.364464807 |
| 1.43025 | 0.36327235 |
| 1.4303 | 0.362077964 |
| 1.43035 | 0.36088165 |
| 1.4304 | 0.359683408 |
| 1.43045 | 0.358483238 |
| 1.4305 | 0.357281141 |
| 1.43055 | 0.356077117 |
| 1.4306 | 0.354871166 |
| 1.43065 | 0.35366329 |
| 1.4307 | 0.352453488 |
| 1.43075 | 0.351241762 |
| 1.4308 | 0.350028112 |
| 1.43085 | 0.348812538 |
| 1.4309 | 0.347595042 |
| 1.43095 | 0.346375625 |
| 1.431 | 0.345154286 |
| 1.43105 | 0.343931027 |
| 1.4311 | 0.342705849 |
| 1.43115 | 0.341478753 |
| 1.4312 | 0.34024974 |
| 1.43125 | 0.339018811 |
| 1.4313 | 0.337785968 |
| 1.43135 | 0.33655121 |
| 1.4314 | 0.33531454 |
| 1.43145 | 0.334075959 |
| 1.4315 | 0.332835468 |
| 1.43155 | 0.331593069 |
| 1.4316 | 0.330348762 |
| 1.43165 | 0.32910255 |
| 1.4317 | 0.327854434 |
| 1.43175 | 0.326604416 |
| 1.4318 | 0.325352497 |
| 1.43185 | 0.324098679 |
| 1.4319 | 0.322842964 |
| 1.43195 | 0.321585354 |
| 1.432 | 0.32032585 |
| 1.43205 | 0.319064455 |
| 1.4321 | 0.31780117 |
| 1.43215 | 0.316535998 |
| 1.4322 | 0.31526894 |
| 1.43225 | 0.314 |
| 1.4323 | 0.312729178 |
| 1.43235 | 0.311456479 |
| 1.4324 | 0.310181903 |
| 1.43245 | 0.308905454 |
| 1.4325 | 0.307627134 |
| 1.43255 | 0.306346945 |
| 1.4326 | 0.305064891 |
| 1.43265 | 0.303780974 |
| 1.4327 | 0.302495196 |
| 1.43275 | 0.301207562 |
| 1.4328 | 0.299918073 |
| 1.43285 | 0.298626733 |
| 1.4329 | 0.297333546 |
| 1.43295 | 0.296038513 |
| 1.433 | 0.294741639 |
| 1.43305 | 0.293442926 |
| 1.4331 | 0.292142379 |
| 1.43315 | 0.290840001 |
| 1.4332 | 0.289535796 |
| 1.43325 | 0.288229766 |
| 1.4333 | 0.286921917 |
| 1.43335 | 0.285612251 |
| 1.4334 | 0.284300773 |
| 1.43345 | 0.282987487 |
| 1.4335 | 0.281672397 |
| 1.43355 | 0.280355508 |
| 1.4336 | 0.279036823 |
| 1.43365 | 0.277716347 |
| 1.4337 | 0.276394084 |
| 1.43375 | 0.27507004 |
| 1.4338 | 0.273744219 |
| 1.43385 | 0.272416625 |
| 1.4339 | 0.271087265 |
| 1.43395 | 0.269756142 |
| 1.434 | 0.268423262 |
| 1.43405 | 0.26708863 |
| 1.4341 | 0.265752252 |
| 1.43415 | 0.264414133 |
| 1.4342 | 0.263074278 |
| 1.43425 | 0.261732694 |
| 1.4343 | 0.260389386 |
| 1.43435 | 0.25904436 |
| 1.4344 | 0.257697622 |
| 1.43445 | 0.256349179 |
| 1.4345 | 0.254999036 |
| 1.43455 | 0.253647201 |
| 1.4346 | 0.252293679 |
| 1.43465 | 0.250938477 |
| 1.4347 | 0.249581603 |
| 1.43475 | 0.248223064 |
| 1.4348 | 0.246862865 |
| 1.43485 | 0.245501015 |
| 1.4349 | 0.244137521 |
| 1.43495 | 0.242772391 |
| 1.435 | 0.241405632 |
| 1.43505 | 0.240037252 |
| 1.4351 | 0.238667259 |
| 1.43515 | 0.237295661 |
| 1.4352 | 0.235922466 |
| 1.43525 | 0.234547684 |
| 1.4353 | 0.233171322 |
| 1.43535 | 0.231793389 |
| 1.4354 | 0.230413895 |
| 1.43545 | 0.229032848 |
| 1.4355 | 0.227650258 |
| 1.43555 | 0.226266134 |
| 1.4356 | 0.224880486 |
| 1.43565 | 0.223493324 |
| 1.4357 | 0.222104657 |
| 1.43575 | 0.220714496 |
| 1.4358 | 0.219322852 |
| 1.43585 | 0.217929734 |
| 1.4359 | 0.216535155 |
| 1.43595 | 0.215139124 |
| 1.436 | 0.213741653 |
| 1.43605 | 0.212342753 |
| 1.4361 | 0.210942437 |
| 1.43615 | 0.209540715 |
| 1.4362 | 0.208137601 |
| 1.43625 | 0.206733106 |
| 1.4363 | 0.205327243 |
| 1.43635 | 0.203920024 |
| 1.4364 | 0.202511463 |
| 1.43645 | 0.201101573 |
| 1.4365 | 0.199690368 |
| 1.43655 | 0.198277861 |
| 1.4366 | 0.196864067 |
| 1.43665 | 0.195448999 |
| 1.4367 | 0.194032672 |
| 1.43675 | 0.192615101 |
| 1.4368 | 0.191196302 |
| 1.43685 | 0.189776289 |
| 1.4369 | 0.188355079 |
| 1.43695 | 0.186932687 |
| 1.437 | 0.18550913 |
| 1.43705 | 0.184084424 |
| 1.4371 | 0.182658587 |
| 1.43715 | 0.181231635 |
| 1.4372 | 0.179803586 |
| 1.43725 | 0.178374459 |
| 1.4373 | 0.176944271 |
| 1.43735 | 0.175513041 |
| 1.4374 | 0.174080788 |
| 1.43745 | 0.172647532 |
| 1.4375 | 0.171213292 |
| 1.43755 | 0.169778088 |
| 1.4376 | 0.168341941 |
| 1.43765 | 0.166904872 |
| 1.4377 | 0.165466903 |
| 1.43775 | 0.164028054 |
| 1.4378 | 0.162588349 |
| 1.43785 | 0.161147809 |
| 1.4379 | 0.159706459 |
| 1.43795 | 0.158264321 |
| 1.438 | 0.156821419 |
| 1.43805 | 0.155377779 |
| 1.4381 | 0.153933425 |
| 1.43815 | 0.152488382 |
| 1.4382 | 0.151042677 |
| 1.43825 | 0.149596336 |
| 1.4383 | 0.148149386 |
| 1.43835 | 0.146701856 |
| 1.4384 | 0.145253772 |
| 1.43845 | 0.143805165 |
| 1.4385 | 0.142356063 |
| 1.43855 | 0.140906497 |
| 1.4386 | 0.139456497 |
| 1.43865 | 0.138006095 |
| 1.4387 | 0.136555322 |
| 1.43875 | 0.135104212 |
| 1.4388 | 0.133652797 |
| 1.43885 | 0.132201113 |
| 1.4389 | 0.130749192 |
| 1.43895 | 0.129297073 |
| 1.439 | 0.12784479 |
| 1.43905 | 0.126392381 |
| 1.4391 | 0.124939884 |
| 1.43915 | 0.123487338 |
| 1.4392 | 0.122034782 |
| 1.43925 | 0.120582258 |
| 1.4393 | 0.119129808 |
| 1.43935 | 0.117677472 |
| 1.4394 | 0.116225296 |
| 1.43945 | 0.114773324 |
| 1.4395 | 0.113321601 |
| 1.43955 | 0.111870174 |
| 1.4396 | 0.110419091 |
| 1.43965 | 0.108968402 |
| 1.4397 | 0.107518155 |
| 1.43975 | 0.106068403 |
| 1.4398 | 0.104619198 |
| 1.43985 | 0.103170594 |
| 1.4399 | 0.101722646 |
| 1.43995 | 0.100275412 |
| 1.44 | 0.098828949 |
| 1.44005 | 0.097383318 |
| 1.4401 | 0.095938578 |
| 1.44015 | 0.094494794 |
| 1.4402 | 0.09305203 |
| 1.44025 | 0.091610351 |
| 1.4403 | 0.090169826 |
| 1.44035 | 0.088730525 |
| 1.4404 | 0.087292519 |
| 1.44045 | 0.085855883 |
| 1.4405 | 0.084420692 |
| 1.44055 | 0.082987024 |
| 1.4406 | 0.081554959 |
| 1.44065 | 0.080124581 |
| 1.4407 | 0.078695974 |
| 1.44075 | 0.077269226 |
| 1.4408 | 0.075844427 |
| 1.44085 | 0.074421669 |
| 1.4409 | 0.07300105 |
| 1.44095 | 0.071582668 |
| 1.441 | 0.070166624 |
| 1.44105 | 0.068753025 |
| 1.4411 | 0.067341978 |
| 1.44115 | 0.065933597 |
| 1.4412 | 0.064527998 |
| 1.44125 | 0.0631253 |
| 1.4413 | 0.061725628 |
| 1.44135 | 0.060329111 |
| 1.4414 | 0.058935882 |
| 1.44145 | 0.057546079 |
| 1.4415 | 0.056159845 |
| 1.44155 | 0.054777329 |
| 1.4416 | 0.053398685 |
| 1.44165 | 0.052024074 |
| 1.4417 | 0.050653662 |
| 1.44175 | 0.049287623 |
| 1.4418 | 0.047926137 |
| 1.44185 | 0.046569393 |
| 1.4419 | 0.045217586 |
| 1.44195 | 0.043870923 |
| 1.442 | 0.042529616 |
| 1.44205 | 0.041193889 |
| 1.4421 | 0.039863978 |
| 1.44215 | 0.038540127 |
| 1.4422 | 0.037222594 |
| 1.44225 | 0.03591165 |
| 1.4423 | 0.03460758 |
| 1.44235 | 0.033310682 |
| 1.4424 | 0.032021274 |
| 1.44245 | 0.03073969 |
| 1.4425 | 0.029466281 |
| 1.44255 | 0.028201423 |
| 1.4426 | 0.026945513 |
| 1.44265 | 0.025698973 |
| 1.4427 | 0.024462253 |
| 1.44275 | 0.023235835 |
| 1.4428 | 0.022020234 |
| 1.44285 | 0.020816004 |
| 1.4429 | 0.019623739 |
| 1.44295 | 0.018444083 |
| 1.443 | 0.017277735 |
| 1.44305 | 0.016125454 |
| 1.4431 | 0.01498807 |
| 1.44315 | 0.013866496 |
| 1.4432 | 0.01276174 |
| 1.44325 | 0.011674923 |
| 1.4433 | 0.010607299 |
| 1.44335 | 0.009560288 |
| 1.4434 | 0.00853551 |
| 1.44345 | 0.007534838 |
| 1.4435 | 0.006560473 |
| 1.44355 | 0.005615048 |
| 1.4436 | 0.004701801 |
| 1.44365 | 0.003824837 |
| 1.4437 | 0.002989608 |
| 1.44375 | 0.00220381 |
| 1.4438 | 0.001479266 |
| 1.44385 | 0.000836408 |
| 1.4439 | 0.000316065 |
| 1.444 | 0 |
| 1.446 | 0.00036307 |
| 1.45 | 0.00145506 |
| 1.454 | 0.00233888 |
| 1.458 | 0.00306393 |
| 1.462 | 0.0036866 |
| 1.466 | 0.00423978 |
| 1.47 | 0.00474291 |
| 1.474 | 0.00520805 |
| 1.478 | 0.00564312 |
| 1.482 | 0.00605355 |
| 1.486 | 0.00644323 |
| 1.49 | 0.00681504 |
| 1.494 | 0.00717118 |
| 1.498 | 0.00751339 |
| 1.502 | 0.00784305 |
| 1.506 | 0.00816131 |
| 1.51 | 0.00846913 |
| 1.514 | 0.00876732 |
| 1.518 | 0.00905657 |
| 1.522 | 0.00933749 |
| 1.526 | 0.00961061 |
| 1.53 | 0.0098764 |
| 1.534 | 0.01013528 |
| 1.538 | 0.01038763 |
| 1.542 | 0.01063379 |
| 1.546 | 0.01087407 |
| 1.55 | 0.01110874 |
| 1.554 | 0.01133807 |
| 1.558 | 0.01156229 |
| 1.562 | 0.01178163 |
| 1.566 | 0.01199628 |
| 1.57 | 0.01220643 |
| 1.574 | 0.01241226 |
| 1.578 | 0.01261393 |
| 1.582 | 0.0128116 |
| 1.586 | 0.0130054 |
| 1.59 | 0.01319548 |
| 1.594 | 0.01338195 |
| 1.598 | 0.01356494 |
| 1.602 | 0.01374455 |
| 1.606 | 0.0139209 |
| 1.61 | 0.01409409 |
