# Supplementary material for: Author Correction: Comprehensive Modeling of Multimode Fiber Sensors for Refractive Index Measurement and Experimental Validation
Source: Sci Rep. 2021 Nov 9;11:22212. doi: 10.1038/s41598-021-99941-2 (PMC8578204; doi:10.1038/s41598-021-99941-2)
Supplement: Supplementary file 1 — Supplementary Information. [file 41598_2021_99941_MOESM1_ESM.docx]

**Comprehensive Modeling of Multimode Fiber Sensors for Refractive Index Measurement and Experimental Validation**

Haris Apriyanto^1,2^, Gautier Ravet^1^, Olivier D. Bernal^1^, Michel Cattoen^1^, Han Cheng Seat^1*^, Valérie Chavagnac^3^, Frédéric Surre^4^, James H. Sharp^5^

^1^LAAS-CNRS, Université de Toulouse, CNRS, INP, Toulouse, France.

^2^Politeknik Negeri Indramayu, Indramayu, Indonesia.

^3^GET - UMR5563, OMP, Université de Toulouse, CNRS, IRD, Toulouse, France.

^4^Photonics & Instrumentation Research Centre, School of Mathematics, Computer Science and Engineering, City, University of London, UK.

^5^Systems, Power and Energy Research Division, School of Engineering, University of Glasgow, Glasgow G12 8QQ, UK.

^*^seat@enseeiht.fr

**Table 1.** Experimental results for 1-cm sensing length over the three sensing zones. All measurements are repeated over 20 times with 100,000 sampling points.

| RI @589 nm | Glycerol concentration | RI @1550 nm | Normalized Power (a.u.) | Sensitivity (a.u./RIU) | Normalized Vrms noise (a.u.) | Resolution (RIU) |
| --- | --- | --- | --- | --- | --- | --- |
| 1.33299 | 0 | 1.316482 | 0.99119794 | -0.10418137 | 0.00030767 | -0.017719003 |
| 1.35062 | 0.14609008 | 1.333793 | 0.98033255 | -0.16024358 | 0.00022878 | -0.008566247 |
| 1.3594 | 0.21514446 | 1.342592 | 0.97757856 | -0.20790896 | 0.00018095 | -0.00522194 |
| 1.37026 | 0.29789025 | 1.353584 | 0.97468488 | -0.30688483 | 0.00039283 | -0.00768038 |
| 1.37936 | 0.36517441 | 1.362838 | 0.97159856 | -0.48727478 | 0.00042192 | -0.005195249 |
| 1.38753 | 0.42415596 | 1.371152 | 0.9665174 | -1.36280427 | 0.00041823 | -0.001841353 |
| 1.39908 | 0.50551309 | 1.382885 | 0.95576725 | -2.44384452 | 0.00030238 | -0.000742393 |
| 1.40457 | 0.54345164 | 1.388444 | 0.94757097 | -3.23639349 | 0.00039544 | -0.000733107 |
| 1.4114 | 0.59007608 | 1.395341 | 0.92870093 | -4.59342106 | 0.00030788 | -0.000402159 |
| 1.41925 | 0.64297519 | 1.403237 | 0.89583139 | -6.85973283 | 0.00017827 | -0.000155923 |
| 1.42582 | 0.68676777 | 1.409819 | 0.85443335 | -9.56768923 | 0.00041745 | -0.000261785 |
| 1.4308 | 0.71971722 | 1.414792 | 0.80337262 | -12.2783203 | 0.0002199 | -0.000107459 |
| 1.43698 | 0.76036419 | 1.420944 | 0.72592745 | -16.6560336 | 0.00042991 | -0.000154866 |
| 1.44193 | 0.79276526 | 1.425857 | 0.64425953 | -21.1554802 | 3.1700E-04 | -8.99058E-05 |
| 1.44719 | 0.82708058 | 1.431065 | 0.49093881 | -27.0658892 | 2.1300E-04 | -4.72181E-05 |
| 1.45301 | 0.86495485 | 1.436811 | 0.28962989 | -34.8796196 | 2.2600E-04 | -3.88766E-05 |
| 1.45798 | 0.89725547 | 1.441705 | 0.10581093 | -40.6280902 | 1.5200E-04 | -2.24475E-05 |
| 1.45989 | 0.90966549 | 1.443584 | 0.0264071 | -36.0266705 | 1.3600E-04 | -2.26499E-05 |
| 1.46109 | 0.91746269 | 1.444763 | 0.00420827 | 0.53640825 | 0.00012968 | 0.001450492 |
| 1.46448 | 0.93949546 | 1.448091 | 0.01019219 | 1.20172233 | 0.0002907 | 0.001451402 |
| 1.4736 | 0.99887693 | 1.457025 | 0.01619856 | 0.75160683 | 0.00012567 | 0.001003179 |
|  |  | 1.477 | 0.02685969 | 0.47862122 | 0.0001316 | 0.001649726 |
|  |  | 1.557 | 0.03605211 | 0.22714696 | 0.00012443 | 0.003286718 |
|  |  | 1.608 | 0.05457648 | 0.10736517 | 0.00012593 | 0.007037422 |

**Table 2.** Experimental results for 2.5-cm sensing length over the three sensing zones. All measurements are repeated over 20 times with 100,000 sampling points.

| RI @589 nm | Glycerol concentration | RI @1550 nm | Normalized Power (a.u.) | Sensitivity (a.u./RIU) | Normalized Vrms noise (a.u.) | Resolution (RIU) |
| --- | --- | --- | --- | --- | --- | --- |
| 1.33299 | 0 | 1.316482 | 0.96068347 | -0.252438 | 0.00045499 | -0.010814323 |
| 1.34507 | 0.10136031 | 1.328294 | 0.95498554 | -0.33344481 | 0.00064901 | -0.011678184 |
| 1.35345 | 0.16857047 | 1.336617 | 0.95089827 | -0.4153119 | 0.00060594 | -0.008753999 |
| 1.36318 | 0.24426603 | 1.346408 | 0.94426718 | -0.55733337 | 0.00121668 | -0.013098229 |
| 1.37389 | 0.32494212 | 1.357273 | 0.93487927 | -0.8328196 | 0.00032077 | -0.002310997 |
| 1.38459 | 0.40307741 | 1.368161 | 0.92451354 | -1.53777907 | 0.00044756 | -0.001746266 |
| 1.39088 | 0.447984 | 1.374559 | 0.91478435 | -2.20540543 | 0.00034708 | -0.00094427 |
| 1.39947 | 0.50822275 | 1.38328 | 0.89624265 | -3.19790922 | 0.00031519 | -0.00059136 |
| 1.40636 | 0.55572971 | 1.390254 | 0.8762907 | -4.33787043 | 0.00063704 | -0.000881129 |
| 1.41283 | 0.59976411 | 1.396782 | 0.85324622 | -5.79452009 | 0.00047364 | -0.000490436 |
| 1.42051 | 0.65140516 | 1.404501 | 0.80933941 | -8.18081545 | 0.00043739 | -0.000320788 |
| 1.42711 | 0.69532156 | 1.411108 | 0.76363633 | -10.9864365 | 0.00018484 | -0.000100945 |
| 1.43432 | 0.74289841 | 1.418299 | 0.6990874 | -15.0861139 | 0.00083712 | -0.000332936 |
| 1.44216 | 0.79426796 | 1.426085 | 0.52923788 | -21.0272104 | 0.00088798 | -0.000253381 |
| 1.44834 | 0.83457074 | 1.432201 | 0.40223545 | -26.7718657 | 0.00079974 | -0.000179234 |
| 1.45277 | 0.86339437 | 1.436574 | 0.24614318 | -30.9866065 | 0.00054629 | -0.000105779 |
| 1.4587 | 0.9019336 | 1.442414 | 0.04695708 | -31.3075808 | 0.00015612 | -2.9919E-05 |
| 1.46158 | 0.92064675 | 1.445244 | 0.00029497 | 0.32217908 | 0.00013396 | 0.002494687 |
| 1.46546 | 0.94586738 | 1.449053 | 0.00114947 | 0.39704103 | 0.00011958 | 0.001807007 |
| 1.4736 | 0.99887693 | 1.457025 | 0.00230721 | 0.26644789 | 0.00012084 | 0.002721132 |
|  |  | 1.477 | 0.00723175 | 0.18010735 | 0.00014666 | 0.00488582 |
|  |  | 1.513 | 0.01017297 | 0.12418806 | 0.00013151 | 0.006353751 |
|  |  | 1.6 | 0.01469334 | 0.0734589 | 3.2066E-05 | 0.002619089 |

**Table 3.** Experimental results for 4-cm sensing length over the three sensing zones. All measurements are repeated over 20 times with 100,000 sampling points.

| RI @589 nm | Glycerol concentration | RI @1550 nm | Normalized Power (a.u.) | Sensitivity (a.u./RIU) | Normalized Vrms noise (a.u.) | Resolution (RIU) |
| --- | --- | --- | --- | --- | --- | --- |
| 1.33299 | 0 | 1.316482 | 0.95978558 | -0.39163525 | 0.00054459 | -0.008343309 |
| 1.34205 | 0.07665072 | 1.325329 | 0.94635342 | -0.47883046 | 0.00025364 | -0.003178244 |
| 1.35729 | 0.19873311 | 1.340469 | 0.93757111 | -0.70925956 | 0.00066059 | -0.005588245 |
| 1.36788 | 0.27999401 | 1.351168 | 0.92771297 | -0.82270051 | 0.00087589 | -0.006387914 |
| 1.37494 | 0.33271362 | 1.358341 | 0.91780905 | -1.29104321 | 0.00033218 | -0.001543789 |
| 1.38368 | 0.39652043 | 1.367235 | 0.89666189 | -1.9782062 | 0.00101767 | -0.003086636 |
| 1.39333 | 0.46528756 | 1.377049 | 0.87315602 | -2.94010685 | 0.00037632 | -0.000767974 |
| 1.40221 | 0.52719616 | 1.386056 | 0.84457005 | -4.16591543 | 0.00036702 | -0.00052861 |
| 1.41099 | 0.58729388 | 1.394927 | 0.81228951 | -5.93127206 | 0.00061391 | -0.00062102 |
| 1.4182 | 0.63593805 | 1.402182 | 0.77948725 | -7.95212594 | 0.00059494 | -0.000448889 |
| 1.42539 | 0.68391344 | 1.409389 | 0.7283047 | -10.6467143 | 0.00059226 | -0.000333772 |
| 1.42928 | 0.70968053 | 1.413275 | 0.68864847 | -12.4466615 | 0.00038604 | -0.000186093 |
| 1.43587 | 0.75308089 | 1.419841 | 0.59023452 | -16.1270989 | 0.0004976 | -0.00018513 |
| 1.44238 | 0.79570512 | 1.426303 | 0.46469603 | -20.5581468 | 0.00061064 | -0.000178218 |
| 1.44904 | 0.8391282 | 1.432893 | 0.29156052 | -25.5809703 | 0.00042345 | -9.93188E-05 |
| 1.45395 | 0.87106592 | 1.437737 | 0.14468315 | -28.5422816 | 0.00035917 | -7.55031E-05 |
| 1.45846 | 0.90037424 | 1.442178 | 0.03202889 | -26.1830757 | 0.00014241 | -3.26345E-05 |
| 1.46109 | 0.91746269 | 1.444763 | 0.00014526 | 0.10414824 | 0.00013303 | 0.007663999 |
| 1.46448 | 0.93949546 | 1.448091 | 0.00096785 | 0.24579291 | 0.00013076 | 0.003192029 |
| 1.4736 | 0.99887693 | 1.457025 | 0.00213901 | 0.16190594 | 0.00015811 | 0.005859328 |
|  |  | 1.477 | 0.00267084 | 0.10414819 | 0.00012702 | 0.007317362 |
|  |  | 1.513 | 0.00358808 | 0.07287114 | 0.0001421 | 0.011699941 |
|  |  | 1.591 | 0.00488893 | 0.04640004 | 0.00042248 | 0.054630986 |

**Table 4.** Simulation results for normalized initial power (P_0_)

| RI @1550 nm | Incident angle, θ (°) | Normalized power (a.u.) | ΔP, power for each ray (a.u.) |
| --- | --- | --- | --- |
| 1.37 | 71.5777682 | 0.99996886 | 4.2098E-05 |
| 1.37005 | 71.5840471 | 0.99992676 | 4.2227E-05 |
| 1.3701 | 71.5903281 | 0.99988453 | 4.2357E-05 |
| 1.37015 | 71.5966112 | 0.99984217 | 4.2486E-05 |
| 1.3702 | 71.6028964 | 0.99979969 | 4.2616E-05 |
| 1.37025 | 71.6091836 | 0.99975707 | 4.2747E-05 |
| 1.3703 | 71.6154729 | 0.99971432 | 4.2878E-05 |
| 1.37035 | 71.6217642 | 0.99967145 | 4.3009E-05 |
| 1.3704 | 71.6280577 | 0.99962844 | 4.3141E-05 |
| 1.37045 | 71.6343532 | 0.9995853 | 4.3273E-05 |
| 1.3705 | 71.6406508 | 0.99954202 | 4.3405E-05 |
| 1.37055 | 71.6469506 | 0.99949862 | 4.3538E-05 |
| 1.3706 | 71.6532523 | 0.99945508 | 4.3671E-05 |
| 1.37065 | 71.6595562 | 0.99941141 | 4.3805E-05 |
| 1.3707 | 71.6658622 | 0.9993676 | 4.3939E-05 |
| 1.37075 | 71.6721703 | 0.99932366 | 4.4074E-05 |
| 1.3708 | 71.6784805 | 0.99927959 | 4.4208E-05 |
| 1.37085 | 71.6847927 | 0.99923538 | 4.4344E-05 |
| 1.3709 | 71.6911071 | 0.99919104 | 4.4479E-05 |
| 1.37095 | 71.6974236 | 0.99914656 | 4.4615E-05 |
| 1.371 | 71.7037422 | 0.99910194 | 4.4752E-05 |
| 1.37105 | 71.7100628 | 0.99905719 | 4.4888E-05 |
| 1.3711 | 71.7163856 | 0.9990123 | 4.5026E-05 |
| 1.37115 | 71.7227106 | 0.99896728 | 4.5163E-05 |
| 1.3712 | 71.7290376 | 0.99892212 | 4.5301E-05 |
| 1.37125 | 71.7353667 | 0.99887681 | 4.544E-05 |
| 1.3713 | 71.741698 | 0.99883137 | 4.5578E-05 |
| 1.37135 | 71.7480314 | 0.9987858 | 4.5718E-05 |
| 1.3714 | 71.7543669 | 0.99874008 | 4.5857E-05 |
| 1.37145 | 71.7607045 | 0.99869422 | 4.5997E-05 |
| 1.3715 | 71.7670443 | 0.99864822 | 4.6138E-05 |
| 1.37155 | 71.7733862 | 0.99860209 | 4.6279E-05 |
| 1.3716 | 71.7797302 | 0.99855581 | 4.642E-05 |
| 1.37165 | 71.7860764 | 0.99850939 | 4.6562E-05 |
| 1.3717 | 71.7924246 | 0.99846283 | 4.6704E-05 |
| 1.37175 | 71.7987751 | 0.99841612 | 4.6847E-05 |
| 1.3718 | 71.8051277 | 0.99836927 | 4.6989E-05 |
| 1.37185 | 71.8114824 | 0.99832229 | 4.7133E-05 |
| 1.3719 | 71.8178392 | 0.99827515 | 4.7277E-05 |
| 1.37195 | 71.8241983 | 0.99822788 | 4.7421E-05 |
| 1.372 | 71.8305594 | 0.99818045 | 4.7566E-05 |
| 1.37205 | 71.8369227 | 0.99813289 | 4.7711E-05 |
| 1.3721 | 71.8432882 | 0.99808518 | 4.7856E-05 |
| 1.37215 | 71.8496558 | 0.99803732 | 4.8002E-05 |
| 1.3722 | 71.8560256 | 0.99798932 | 4.8148E-05 |
| 1.37225 | 71.8623976 | 0.99794117 | 4.8295E-05 |
| 1.3723 | 71.8687717 | 0.99789288 | 4.8443E-05 |
| 1.37235 | 71.875148 | 0.99784443 | 4.859E-05 |
| 1.3724 | 71.8815264 | 0.99779584 | 4.8738E-05 |
| 1.37245 | 71.887907 | 0.99774711 | 4.8887E-05 |
| 1.3725 | 71.8942898 | 0.99769822 | 4.9036E-05 |
| 1.37255 | 71.9006748 | 0.99764918 | 4.9185E-05 |
| 1.3726 | 71.907062 | 0.9976 | 4.9335E-05 |
| 1.37265 | 71.9134513 | 0.99755066 | 4.9485E-05 |
| 1.3727 | 71.9198428 | 0.99750118 | 4.9636E-05 |
| 1.37275 | 71.9262365 | 0.99745154 | 4.9787E-05 |
| 1.3728 | 71.9326324 | 0.99740175 | 4.9939E-05 |
| 1.37285 | 71.9390304 | 0.99735182 | 5.0091E-05 |
| 1.3729 | 71.9454307 | 0.99730172 | 5.0243E-05 |
| 1.37295 | 71.9518331 | 0.99725148 | 5.0396E-05 |
| 1.373 | 71.9582378 | 0.99720109 | 5.055E-05 |
| 1.37305 | 71.9646447 | 0.99715054 | 5.0704E-05 |
| 1.3731 | 71.9710537 | 0.99709983 | 5.0858E-05 |
| 1.37315 | 71.977465 | 0.99704897 | 5.1013E-05 |
| 1.3732 | 71.9838784 | 0.99699796 | 5.1168E-05 |
| 1.37325 | 71.9902941 | 0.99694679 | 5.1323E-05 |
| 1.3733 | 71.996712 | 0.99689547 | 5.148E-05 |
| 1.37335 | 72.0031321 | 0.99684399 | 5.1636E-05 |
| 1.3734 | 72.0095544 | 0.99679235 | 5.1793E-05 |
| 1.37345 | 72.0159789 | 0.99674056 | 5.1951E-05 |
| 1.3735 | 72.0224056 | 0.99668861 | 5.2109E-05 |
| 1.37355 | 72.0288346 | 0.9966365 | 5.2267E-05 |
| 1.3736 | 72.0352658 | 0.99658424 | 5.2426E-05 |
| 1.37365 | 72.0416992 | 0.99653181 | 5.2585E-05 |
| 1.3737 | 72.0481349 | 0.99647922 | 5.2745E-05 |
| 1.37375 | 72.0545727 | 0.99642648 | 5.2905E-05 |
| 1.3738 | 72.0610129 | 0.99637357 | 5.3066E-05 |
| 1.37385 | 72.0674552 | 0.99632051 | 5.3227E-05 |
| 1.3739 | 72.0738998 | 0.99626728 | 5.3389E-05 |
| 1.37395 | 72.0803466 | 0.99621389 | 5.3551E-05 |
| 1.374 | 72.0867957 | 0.99616034 | 5.3714E-05 |
| 1.37405 | 72.093247 | 0.99610663 | 5.3877E-05 |
| 1.3741 | 72.0997006 | 0.99605275 | 5.404E-05 |
| 1.37415 | 72.1061564 | 0.99599871 | 5.4204E-05 |
| 1.3742 | 72.1126145 | 0.99594451 | 5.4369E-05 |
| 1.37425 | 72.1190748 | 0.99589014 | 5.4534E-05 |
| 1.3743 | 72.1255374 | 0.9958356 | 5.4699E-05 |
| 1.37435 | 72.1320023 | 0.9957809 | 5.4865E-05 |
| 1.3744 | 72.1384694 | 0.99572604 | 5.5032E-05 |
| 1.37445 | 72.1449388 | 0.99567101 | 5.5199E-05 |
| 1.3745 | 72.1514104 | 0.99561581 | 5.5366E-05 |
| 1.37455 | 72.1578843 | 0.99556044 | 5.5534E-05 |
| 1.3746 | 72.1643605 | 0.99550491 | 5.5703E-05 |
| 1.37465 | 72.170839 | 0.9954492 | 5.5872E-05 |
| 1.3747 | 72.1773198 | 0.99539333 | 5.6041E-05 |
| 1.37475 | 72.1838028 | 0.99533729 | 5.6211E-05 |
| 1.3748 | 72.1902881 | 0.99528108 | 5.6381E-05 |
| 1.37485 | 72.1967757 | 0.9952247 | 5.6552E-05 |
| 1.3749 | 72.2032656 | 0.99516815 | 5.6724E-05 |
| 1.37495 | 72.2097578 | 0.99511142 | 5.6896E-05 |
| 1.375 | 72.2162522 | 0.99505453 | 5.7068E-05 |
| 1.37505 | 72.222749 | 0.99499746 | 5.7241E-05 |
| 1.3751 | 72.2292481 | 0.99494022 | 5.7414E-05 |
| 1.37515 | 72.2357494 | 0.9948828 | 5.7588E-05 |
| 1.3752 | 72.2422531 | 0.99482522 | 5.7763E-05 |
| 1.37525 | 72.2487591 | 0.99476745 | 5.7938E-05 |
| 1.3753 | 72.2552674 | 0.99470952 | 5.8113E-05 |
| 1.37535 | 72.261778 | 0.9946514 | 5.8289E-05 |
| 1.3754 | 72.2682909 | 0.99459311 | 5.8465E-05 |
| 1.37545 | 72.2748061 | 0.99453465 | 5.8642E-05 |
| 1.3755 | 72.2813236 | 0.99447601 | 5.882E-05 |
| 1.37555 | 72.2878435 | 0.99441719 | 5.8998E-05 |
| 1.3756 | 72.2943657 | 0.99435819 | 5.9176E-05 |
| 1.37565 | 72.3008902 | 0.99429901 | 5.9355E-05 |
| 1.3757 | 72.3074171 | 0.99423966 | 5.9535E-05 |
| 1.37575 | 72.3139462 | 0.99418012 | 5.9715E-05 |
| 1.3758 | 72.3204778 | 0.99412041 | 5.9896E-05 |
| 1.37585 | 72.3270116 | 0.99406051 | 6.0077E-05 |
| 1.3759 | 72.3335478 | 0.99400043 | 6.0259E-05 |
| 1.37595 | 72.3400863 | 0.99394018 | 6.0441E-05 |
| 1.376 | 72.3466272 | 0.99387974 | 6.0623E-05 |
| 1.37605 | 72.3531704 | 0.99381911 | 6.0807E-05 |
| 1.3761 | 72.359716 | 0.99375831 | 6.0991E-05 |
| 1.37615 | 72.3662639 | 0.99369731 | 6.1175E-05 |
| 1.3762 | 72.3728142 | 0.99363614 | 6.136E-05 |
| 1.37625 | 72.3793668 | 0.99357478 | 6.1545E-05 |
| 1.3763 | 72.3859218 | 0.99351323 | 6.1731E-05 |
| 1.37635 | 72.3924792 | 0.9934515 | 6.1918E-05 |
| 1.3764 | 72.3990389 | 0.99338959 | 6.2105E-05 |
| 1.37645 | 72.405601 | 0.99332748 | 6.2292E-05 |
| 1.3765 | 72.4121654 | 0.99326519 | 6.248E-05 |
| 1.37655 | 72.4187323 | 0.99320271 | 6.2669E-05 |
| 1.3766 | 72.4253015 | 0.99314004 | 6.2858E-05 |
| 1.37665 | 72.4318731 | 0.99307718 | 6.3048E-05 |
| 1.3767 | 72.4384471 | 0.99301413 | 6.3238E-05 |
| 1.37675 | 72.4450234 | 0.9929509 | 6.3429E-05 |
| 1.3768 | 72.4516022 | 0.99288747 | 6.3621E-05 |
| 1.37685 | 72.4581833 | 0.99282385 | 6.3813E-05 |
| 1.3769 | 72.4647668 | 0.99276003 | 6.4005E-05 |
| 1.37695 | 72.4713527 | 0.99269603 | 6.4198E-05 |
| 1.377 | 72.4779411 | 0.99263183 | 6.4392E-05 |
| 1.37705 | 72.4845318 | 0.99256744 | 6.4586E-05 |
| 1.3771 | 72.4911249 | 0.99250285 | 6.4781E-05 |
| 1.37715 | 72.4977204 | 0.99243807 | 6.4976E-05 |
| 1.3772 | 72.5043184 | 0.99237309 | 6.5172E-05 |
| 1.37725 | 72.5109187 | 0.99230792 | 6.5369E-05 |
| 1.3773 | 72.5175215 | 0.99224255 | 6.5566E-05 |
| 1.37735 | 72.5241267 | 0.99217699 | 6.5763E-05 |
| 1.3774 | 72.5307343 | 0.99211122 | 6.5962E-05 |
| 1.37745 | 72.5373443 | 0.99204526 | 6.616E-05 |
| 1.3775 | 72.5439567 | 0.9919791 | 6.636E-05 |
| 1.37755 | 72.5505716 | 0.99191274 | 6.656E-05 |
| 1.3776 | 72.5571889 | 0.99184618 | 6.676E-05 |
| 1.37765 | 72.5638086 | 0.99177942 | 6.6961E-05 |
| 1.3777 | 72.5704308 | 0.99171246 | 6.7163E-05 |
| 1.37775 | 72.5770554 | 0.9916453 | 6.7365E-05 |
| 1.3778 | 72.5836825 | 0.99157793 | 6.7568E-05 |
| 1.37785 | 72.590312 | 0.99151037 | 6.7771E-05 |
| 1.3779 | 72.5969439 | 0.99144259 | 6.7976E-05 |
| 1.37795 | 72.6035783 | 0.99137462 | 6.818E-05 |
| 1.378 | 72.6102152 | 0.99130644 | 6.8385E-05 |
| 1.37805 | 72.6168545 | 0.99123805 | 6.8591E-05 |
| 1.3781 | 72.6234962 | 0.99116946 | 6.8798E-05 |
| 1.37815 | 72.6301405 | 0.99110066 | 6.9005E-05 |
| 1.3782 | 72.6367871 | 0.99103166 | 6.9212E-05 |
| 1.37825 | 72.6434363 | 0.99096245 | 6.942E-05 |
| 1.3783 | 72.6500879 | 0.99089303 | 6.9629E-05 |
| 1.37835 | 72.656742 | 0.9908234 | 6.9839E-05 |
| 1.3784 | 72.6633986 | 0.99075356 | 7.0049E-05 |
| 1.37845 | 72.6700576 | 0.99068351 | 7.0259E-05 |
| 1.3785 | 72.6767192 | 0.99061325 | 7.047E-05 |
| 1.37855 | 72.6833832 | 0.99054278 | 7.0682E-05 |
| 1.3786 | 72.6900497 | 0.9904721 | 7.0895E-05 |
| 1.37865 | 72.6967187 | 0.9904012 | 7.1108E-05 |
| 1.3787 | 72.7033902 | 0.9903301 | 7.1322E-05 |
| 1.37875 | 72.7100641 | 0.99025877 | 7.1536E-05 |
| 1.3788 | 72.7167406 | 0.99018724 | 7.1751E-05 |
| 1.37885 | 72.7234196 | 0.99011549 | 7.1966E-05 |
| 1.3789 | 72.7301011 | 0.99004352 | 7.2183E-05 |
| 1.37895 | 72.7367851 | 0.98997134 | 7.2399E-05 |
| 1.379 | 72.7434716 | 0.98989894 | 7.2617E-05 |
| 1.37905 | 72.7501606 | 0.98982632 | 7.2835E-05 |
| 1.3791 | 72.7568521 | 0.98975349 | 7.3053E-05 |
| 1.37915 | 72.7635462 | 0.98968043 | 7.3273E-05 |
| 1.3792 | 72.7702427 | 0.98960716 | 7.3493E-05 |
| 1.37925 | 72.7769418 | 0.98953367 | 7.3713E-05 |
| 1.3793 | 72.7836435 | 0.98945996 | 7.3935E-05 |
| 1.37935 | 72.7903476 | 0.98938602 | 7.4156E-05 |
| 1.3794 | 72.7970543 | 0.98931186 | 7.4379E-05 |
| 1.37945 | 72.8037635 | 0.98923749 | 7.4602E-05 |
| 1.3795 | 72.8104753 | 0.98916288 | 7.4826E-05 |
| 1.37955 | 72.8171896 | 0.98908806 | 7.505E-05 |
| 1.3796 | 72.8239065 | 0.98901301 | 7.5275E-05 |
| 1.37965 | 72.8306259 | 0.98893773 | 7.5501E-05 |
| 1.3797 | 72.8373478 | 0.98886223 | 7.5728E-05 |
| 1.37975 | 72.8440723 | 0.9887865 | 7.5955E-05 |
| 1.3798 | 72.8507994 | 0.98871055 | 7.6182E-05 |
| 1.37985 | 72.857529 | 0.98863437 | 7.6411E-05 |
| 1.3799 | 72.8642612 | 0.98855796 | 7.664E-05 |
| 1.37995 | 72.8709959 | 0.98848132 | 7.6869E-05 |
| 1.38 | 72.8777333 | 0.98840445 | 7.71E-05 |
| 1.38005 | 72.8844732 | 0.98832735 | 7.7331E-05 |
| 1.3801 | 72.8912156 | 0.98825002 | 7.7562E-05 |
| 1.38015 | 72.8979607 | 0.98817245 | 7.7795E-05 |
| 1.3802 | 72.9047083 | 0.98809466 | 7.8028E-05 |
| 1.38025 | 72.9114585 | 0.98801663 | 7.8261E-05 |
| 1.3803 | 72.9182113 | 0.98793837 | 7.8496E-05 |
| 1.38035 | 72.9249667 | 0.98785987 | 7.8731E-05 |
| 1.3804 | 72.9317247 | 0.98778114 | 7.8966E-05 |
| 1.38045 | 72.9384853 | 0.98770218 | 7.9203E-05 |
| 1.3805 | 72.9452485 | 0.98762297 | 7.944E-05 |
| 1.38055 | 72.9520143 | 0.98754353 | 7.9678E-05 |
| 1.3806 | 72.9587827 | 0.98746386 | 7.9916E-05 |
| 1.38065 | 72.9655537 | 0.98738394 | 8.0155E-05 |
| 1.3807 | 72.9723273 | 0.98730379 | 8.0395E-05 |
| 1.38075 | 72.9791036 | 0.98722339 | 8.0635E-05 |
| 1.3808 | 72.9858824 | 0.98714276 | 8.0877E-05 |
| 1.38085 | 72.9926639 | 0.98706188 | 8.1119E-05 |
| 1.3809 | 72.999448 | 0.98698076 | 8.1361E-05 |
| 1.38095 | 73.0062347 | 0.9868994 | 8.1604E-05 |
| 1.381 | 73.0130241 | 0.98681779 | 8.1848E-05 |
| 1.38105 | 73.0198161 | 0.98673595 | 8.2093E-05 |
| 1.3811 | 73.0266107 | 0.98665385 | 8.2338E-05 |
| 1.38115 | 73.033408 | 0.98657151 | 8.2585E-05 |
| 1.3812 | 73.0402079 | 0.98648893 | 8.2831E-05 |
| 1.38125 | 73.0470105 | 0.9864061 | 8.3079E-05 |
| 1.3813 | 73.0538157 | 0.98632302 | 8.3327E-05 |
| 1.38135 | 73.0606235 | 0.98623969 | 8.3576E-05 |
| 1.3814 | 73.0674341 | 0.98615612 | 8.3826E-05 |
| 1.38145 | 73.0742473 | 0.98607229 | 8.4076E-05 |
| 1.3815 | 73.0810631 | 0.98598821 | 8.4327E-05 |
| 1.38155 | 73.0878816 | 0.98590389 | 8.4579E-05 |
| 1.3816 | 73.0947028 | 0.98581931 | 8.4832E-05 |
| 1.38165 | 73.1015267 | 0.98573448 | 8.5085E-05 |
| 1.3817 | 73.1083532 | 0.98564939 | 8.5339E-05 |
| 1.38175 | 73.1151824 | 0.98556405 | 8.5593E-05 |
| 1.3818 | 73.1220143 | 0.98547846 | 8.5849E-05 |
| 1.38185 | 73.1288489 | 0.98539261 | 8.6105E-05 |
| 1.3819 | 73.1356862 | 0.98530651 | 8.6362E-05 |
| 1.38195 | 73.1425262 | 0.98522014 | 8.662E-05 |
| 1.382 | 73.1493688 | 0.98513352 | 8.6878E-05 |
| 1.38205 | 73.1562142 | 0.98504665 | 8.7137E-05 |
| 1.3821 | 73.1630622 | 0.98495951 | 8.7397E-05 |
| 1.38215 | 73.169913 | 0.98487211 | 8.7658E-05 |
| 1.3822 | 73.1767665 | 0.98478445 | 8.7919E-05 |
| 1.38225 | 73.1836227 | 0.98469653 | 8.8181E-05 |
| 1.3823 | 73.1904816 | 0.98460835 | 8.8444E-05 |
| 1.38235 | 73.1973432 | 0.98451991 | 8.8708E-05 |
| 1.3824 | 73.2042076 | 0.9844312 | 8.8972E-05 |
| 1.38245 | 73.2110746 | 0.98434223 | 8.9237E-05 |
| 1.3825 | 73.2179444 | 0.98425299 | 8.9503E-05 |
| 1.38255 | 73.224817 | 0.98416349 | 8.977E-05 |
| 1.3826 | 73.2316922 | 0.98407372 | 9.0037E-05 |
| 1.38265 | 73.2385703 | 0.98398368 | 9.0306E-05 |
| 1.3827 | 73.245451 | 0.98389338 | 9.0575E-05 |
| 1.38275 | 73.2523345 | 0.9838028 | 9.0844E-05 |
| 1.3828 | 73.2592207 | 0.98371196 | 9.1115E-05 |
| 1.38285 | 73.2661097 | 0.98362084 | 9.1386E-05 |
| 1.3829 | 73.2730015 | 0.98352946 | 9.1658E-05 |
| 1.38295 | 73.279896 | 0.9834378 | 9.1931E-05 |
| 1.383 | 73.2867933 | 0.98334587 | 9.2205E-05 |
| 1.38305 | 73.2936933 | 0.98325366 | 9.2479E-05 |
| 1.3831 | 73.3005962 | 0.98316118 | 9.2754E-05 |
| 1.38315 | 73.3075017 | 0.98306843 | 9.303E-05 |
| 1.3832 | 73.3144101 | 0.9829754 | 9.3307E-05 |
| 1.38325 | 73.3213213 | 0.98288209 | 9.3585E-05 |
| 1.3833 | 73.3282352 | 0.98278851 | 9.3863E-05 |
| 1.38335 | 73.3351519 | 0.98269464 | 9.4142E-05 |
| 1.3834 | 73.3420714 | 0.9826005 | 9.4422E-05 |
| 1.38345 | 73.3489937 | 0.98250608 | 9.4703E-05 |
| 1.3835 | 73.3559188 | 0.98241138 | 9.4984E-05 |
| 1.38355 | 73.3628467 | 0.98231639 | 9.5267E-05 |
| 1.3836 | 73.3697774 | 0.98222113 | 9.555E-05 |
| 1.38365 | 73.3767109 | 0.98212558 | 9.5834E-05 |
| 1.3837 | 73.3836472 | 0.98202974 | 9.6119E-05 |
| 1.38375 | 73.3905864 | 0.98193362 | 9.6404E-05 |
| 1.3838 | 73.3975283 | 0.98183722 | 9.6691E-05 |
| 1.38385 | 73.4044731 | 0.98174053 | 9.6978E-05 |
| 1.3839 | 73.4114207 | 0.98164355 | 9.7266E-05 |
| 1.38395 | 73.4183711 | 0.98154628 | 9.7555E-05 |
| 1.384 | 73.4253244 | 0.98144873 | 9.7845E-05 |
| 1.38405 | 73.4322805 | 0.98135088 | 9.8135E-05 |
| 1.3841 | 73.4392394 | 0.98125275 | 9.8427E-05 |
| 1.38415 | 73.4462012 | 0.98115432 | 9.8719E-05 |
| 1.3842 | 73.4531659 | 0.9810556 | 9.9012E-05 |
| 1.38425 | 73.4601333 | 0.98095659 | 9.9306E-05 |
| 1.3843 | 73.4671037 | 0.98085728 | 9.9601E-05 |
| 1.38435 | 73.4740769 | 0.98075768 | 9.9896E-05 |
| 1.3844 | 73.4810529 | 0.98065779 | 0.00010019 |
| 1.38445 | 73.4880319 | 0.98055759 | 0.00010049 |
| 1.3845 | 73.4950136 | 0.9804571 | 0.00010079 |
| 1.38455 | 73.5019983 | 0.98035632 | 0.00010109 |
| 1.3846 | 73.5089858 | 0.98025523 | 0.00010139 |
| 1.38465 | 73.5159763 | 0.98015384 | 0.00010169 |
| 1.3847 | 73.5229696 | 0.98005215 | 0.00010199 |
| 1.38475 | 73.5299658 | 0.97995016 | 0.00010229 |
| 1.3848 | 73.5369648 | 0.97984787 | 0.00010259 |
| 1.38485 | 73.5439668 | 0.97974528 | 0.0001029 |
| 1.3849 | 73.5509717 | 0.97964238 | 0.0001032 |
| 1.38495 | 73.5579794 | 0.97953918 | 0.00010351 |
| 1.385 | 73.5649901 | 0.97943567 | 0.00010382 |
| 1.38505 | 73.5720037 | 0.97933185 | 0.00010412 |
| 1.3851 | 73.5790202 | 0.97922773 | 0.00010443 |
| 1.38515 | 73.5860396 | 0.97912329 | 0.00010474 |
| 1.3852 | 73.593062 | 0.97901855 | 0.00010505 |
| 1.38525 | 73.6000872 | 0.9789135 | 0.00010536 |
| 1.3853 | 73.6071154 | 0.97880814 | 0.00010568 |
| 1.38535 | 73.6141466 | 0.97870246 | 0.00010599 |
| 1.3854 | 73.6211806 | 0.97859647 | 0.0001063 |
| 1.38545 | 73.6282176 | 0.97849017 | 0.00010662 |
| 1.3855 | 73.6352575 | 0.97838355 | 0.00010693 |
| 1.38555 | 73.6423004 | 0.97827662 | 0.00010725 |
| 1.3856 | 73.6493463 | 0.97816937 | 0.00010757 |
| 1.38565 | 73.6563951 | 0.97806181 | 0.00010788 |
| 1.3857 | 73.6634468 | 0.97795392 | 0.0001082 |
| 1.38575 | 73.6705015 | 0.97784572 | 0.00010852 |
| 1.3858 | 73.6775592 | 0.97773719 | 0.00010884 |
| 1.38585 | 73.6846199 | 0.97762835 | 0.00010917 |
| 1.3859 | 73.6916835 | 0.97751918 | 0.00010949 |
| 1.38595 | 73.6987501 | 0.97740969 | 0.00010981 |
| 1.386 | 73.7058197 | 0.97729988 | 0.00011014 |
| 1.38605 | 73.7128922 | 0.97718974 | 0.00011046 |
| 1.3861 | 73.7199678 | 0.97707928 | 0.00011079 |
| 1.38615 | 73.7270463 | 0.97696849 | 0.00011112 |
| 1.3862 | 73.7341279 | 0.97685737 | 0.00011145 |
| 1.38625 | 73.7412124 | 0.97674592 | 0.00011178 |
| 1.3863 | 73.7483 | 0.97663415 | 0.00011211 |
| 1.38635 | 73.7553906 | 0.97652204 | 0.00011244 |
| 1.3864 | 73.7624841 | 0.97640961 | 0.00011277 |
| 1.38645 | 73.7695807 | 0.97629684 | 0.0001131 |
| 1.3865 | 73.7766803 | 0.97618374 | 0.00011344 |
| 1.38655 | 73.783783 | 0.9760703 | 0.00011377 |
| 1.3866 | 73.7908886 | 0.97595653 | 0.00011411 |
| 1.38665 | 73.7979973 | 0.97584242 | 0.00011444 |
| 1.3867 | 73.8051091 | 0.97572798 | 0.00011478 |
| 1.38675 | 73.8122239 | 0.9756132 | 0.00011512 |
| 1.3868 | 73.8193417 | 0.97549807 | 0.00011546 |
| 1.38685 | 73.8264625 | 0.97538261 | 0.0001158 |
| 1.3869 | 73.8335865 | 0.97526681 | 0.00011614 |
| 1.38695 | 73.8407134 | 0.97515067 | 0.00011649 |
| 1.387 | 73.8478435 | 0.97503418 | 0.00011683 |
| 1.38705 | 73.8549766 | 0.97491736 | 0.00011717 |
| 1.3871 | 73.8621128 | 0.97480018 | 0.00011752 |
| 1.38715 | 73.869252 | 0.97468266 | 0.00011787 |
| 1.3872 | 73.8763943 | 0.9745648 | 0.00011821 |
| 1.38725 | 73.8835397 | 0.97444658 | 0.00011856 |
| 1.3873 | 73.8906882 | 0.97432802 | 0.00011891 |
| 1.38735 | 73.8978398 | 0.97420911 | 0.00011926 |
| 1.3874 | 73.9049945 | 0.97408985 | 0.00011961 |
| 1.38745 | 73.9121523 | 0.97397023 | 0.00011997 |
| 1.3875 | 73.9193131 | 0.97385027 | 0.00012032 |
| 1.38755 | 73.9264771 | 0.97372995 | 0.00012067 |
| 1.3876 | 73.9336442 | 0.97360927 | 0.00012103 |
| 1.38765 | 73.9408144 | 0.97348824 | 0.00012139 |
| 1.3877 | 73.9479877 | 0.97336686 | 0.00012174 |
| 1.38775 | 73.9551642 | 0.97324511 | 0.0001221 |
| 1.3878 | 73.9623437 | 0.97312301 | 0.00012246 |
| 1.38785 | 73.9695264 | 0.97300055 | 0.00012282 |
| 1.3879 | 73.9767123 | 0.97287772 | 0.00012318 |
| 1.38795 | 73.9839013 | 0.97275454 | 0.00012355 |
| 1.388 | 73.9910934 | 0.97263099 | 0.00012391 |
| 1.38805 | 73.9982887 | 0.97250708 | 0.00012428 |
| 1.3881 | 74.0054871 | 0.97238281 | 0.00012464 |
| 1.38815 | 74.0126887 | 0.97225817 | 0.00012501 |
| 1.3882 | 74.0198934 | 0.97213316 | 0.00012538 |
| 1.38825 | 74.0271013 | 0.97200778 | 0.00012574 |
| 1.3883 | 74.0343124 | 0.97188204 | 0.00012611 |
| 1.38835 | 74.0415266 | 0.97175592 | 0.00012649 |
| 1.3884 | 74.048744 | 0.97162944 | 0.00012686 |
| 1.38845 | 74.0559646 | 0.97150258 | 0.00012723 |
| 1.3885 | 74.0631884 | 0.97137535 | 0.0001276 |
| 1.38855 | 74.0704154 | 0.97124775 | 0.00012798 |
| 1.3886 | 74.0776456 | 0.97111977 | 0.00012836 |
| 1.38865 | 74.084879 | 0.97099141 | 0.00012873 |
| 1.3887 | 74.0921156 | 0.97086268 | 0.00012911 |
| 1.38875 | 74.0993554 | 0.97073356 | 0.00012949 |
| 1.3888 | 74.1065984 | 0.97060407 | 0.00012987 |
| 1.38885 | 74.1138446 | 0.9704742 | 0.00013025 |
| 1.3889 | 74.121094 | 0.97034395 | 0.00013064 |
| 1.38895 | 74.1283467 | 0.97021331 | 0.00013102 |
| 1.389 | 74.1356026 | 0.97008229 | 0.0001314 |
| 1.38905 | 74.1428617 | 0.96995089 | 0.00013179 |
| 1.3891 | 74.1501241 | 0.9698191 | 0.00013218 |
| 1.38915 | 74.1573897 | 0.96968692 | 0.00013257 |
| 1.3892 | 74.1646586 | 0.96955436 | 0.00013295 |
| 1.38925 | 74.1719307 | 0.9694214 | 0.00013334 |
| 1.3893 | 74.1792061 | 0.96928806 | 0.00013374 |
| 1.38935 | 74.1864847 | 0.96915432 | 0.00013413 |
| 1.3894 | 74.1937666 | 0.96902019 | 0.00013452 |
| 1.38945 | 74.2010518 | 0.96888567 | 0.00013492 |
| 1.3895 | 74.2083402 | 0.96875075 | 0.00013531 |
| 1.38955 | 74.215632 | 0.96861544 | 0.00013571 |
| 1.3896 | 74.222927 | 0.96847973 | 0.00013611 |
| 1.38965 | 74.2302253 | 0.96834362 | 0.00013651 |
| 1.3897 | 74.2375269 | 0.96820712 | 0.00013691 |
| 1.38975 | 74.2448318 | 0.96807021 | 0.00013731 |
| 1.3898 | 74.25214 | 0.9679329 | 0.00013771 |
| 1.38985 | 74.2594515 | 0.96779519 | 0.00013812 |
| 1.3899 | 74.2667663 | 0.96765707 | 0.00013852 |
| 1.38995 | 74.2740844 | 0.96751855 | 0.00013893 |
| 1.39 | 74.2814059 | 0.96737963 | 0.00013933 |
| 1.39005 | 74.2887307 | 0.96724029 | 0.00013974 |
| 1.3901 | 74.2960588 | 0.96710055 | 0.00014015 |
| 1.39015 | 74.3033902 | 0.9669604 | 0.00014056 |
| 1.3902 | 74.310725 | 0.96681984 | 0.00014097 |
| 1.39025 | 74.3180631 | 0.96667887 | 0.00014139 |
| 1.3903 | 74.3254046 | 0.96653748 | 0.0001418 |
| 1.39035 | 74.3327494 | 0.96639568 | 0.00014222 |
| 1.3904 | 74.3400976 | 0.96625347 | 0.00014263 |
| 1.39045 | 74.3474492 | 0.96611083 | 0.00014305 |
| 1.3905 | 74.3548041 | 0.96596778 | 0.00014347 |
| 1.39055 | 74.3621624 | 0.96582432 | 0.00014389 |
| 1.3906 | 74.3695241 | 0.96568043 | 0.00014431 |
| 1.39065 | 74.3768891 | 0.96553612 | 0.00014473 |
| 1.3907 | 74.3842575 | 0.96539139 | 0.00014516 |
| 1.39075 | 74.3916294 | 0.96524623 | 0.00014558 |
| 1.3908 | 74.3990046 | 0.96510065 | 0.00014601 |
| 1.39085 | 74.4063832 | 0.96495465 | 0.00014643 |
| 1.3909 | 74.4137653 | 0.96480821 | 0.00014686 |
| 1.39095 | 74.4211507 | 0.96466135 | 0.00014729 |
| 1.391 | 74.4285396 | 0.96451406 | 0.00014772 |
| 1.39105 | 74.4359319 | 0.96436634 | 0.00014815 |
| 1.3911 | 74.4433276 | 0.96421819 | 0.00014859 |
| 1.39115 | 74.4507267 | 0.9640696 | 0.00014902 |
| 1.3912 | 74.4581293 | 0.96392058 | 0.00014946 |
| 1.39125 | 74.4655353 | 0.96377113 | 0.00014989 |
| 1.3913 | 74.4729448 | 0.96362123 | 0.00015033 |
| 1.39135 | 74.4803577 | 0.9634709 | 0.00015077 |
| 1.3914 | 74.4877741 | 0.96332013 | 0.00015121 |
| 1.39145 | 74.4951939 | 0.96316892 | 0.00015165 |
| 1.3915 | 74.5026172 | 0.96301727 | 0.0001521 |
| 1.39155 | 74.510044 | 0.96286517 | 0.00015254 |
| 1.3916 | 74.5174742 | 0.96271263 | 0.00015298 |
| 1.39165 | 74.5249079 | 0.96255965 | 0.00015343 |
| 1.3917 | 74.5323451 | 0.96240622 | 0.00015388 |
| 1.39175 | 74.5397858 | 0.96225234 | 0.00015433 |
| 1.3918 | 74.54723 | 0.96209801 | 0.00015478 |
| 1.39185 | 74.5546777 | 0.96194323 | 0.00015523 |
| 1.3919 | 74.5621289 | 0.961788 | 0.00015568 |
| 1.39195 | 74.5695836 | 0.96163231 | 0.00015614 |
| 1.392 | 74.5770418 | 0.96147618 | 0.00015659 |
| 1.39205 | 74.5845036 | 0.96131958 | 0.00015705 |
| 1.3921 | 74.5919688 | 0.96116253 | 0.00015751 |
| 1.39215 | 74.5994376 | 0.96100502 | 0.00015797 |
| 1.3922 | 74.60691 | 0.96084705 | 0.00015843 |
| 1.39225 | 74.6143859 | 0.96068863 | 0.00015889 |
| 1.3923 | 74.6218653 | 0.96052973 | 0.00015935 |
| 1.39235 | 74.6293483 | 0.96037038 | 0.00015982 |
| 1.3924 | 74.6368348 | 0.96021056 | 0.00016028 |
| 1.39245 | 74.6443249 | 0.96005028 | 0.00016075 |
| 1.3925 | 74.6518186 | 0.95988952 | 0.00016122 |
| 1.39255 | 74.6593158 | 0.9597283 | 0.00016169 |
| 1.3926 | 74.6668166 | 0.95956661 | 0.00016216 |
| 1.39265 | 74.674321 | 0.95940445 | 0.00016263 |
| 1.3927 | 74.681829 | 0.95924182 | 0.00016311 |
| 1.39275 | 74.6893406 | 0.95907871 | 0.00016358 |
| 1.3928 | 74.6968557 | 0.95891513 | 0.00016406 |
| 1.39285 | 74.7043745 | 0.95875107 | 0.00016454 |
| 1.3929 | 74.7118969 | 0.95858653 | 0.00016502 |
| 1.39295 | 74.7194229 | 0.95842152 | 0.0001655 |
| 1.393 | 74.7269525 | 0.95825602 | 0.00016598 |
| 1.39305 | 74.7344858 | 0.95809004 | 0.00016646 |
| 1.3931 | 74.7420227 | 0.95792358 | 0.00016695 |
| 1.39315 | 74.7495632 | 0.95775663 | 0.00016743 |
| 1.3932 | 74.7571074 | 0.9575892 | 0.00016792 |
| 1.39325 | 74.7646552 | 0.95742128 | 0.00016841 |
| 1.3933 | 74.7722066 | 0.95725288 | 0.0001689 |
| 1.39335 | 74.7797617 | 0.95708398 | 0.00016939 |
| 1.3934 | 74.7873205 | 0.95691459 | 0.00016988 |
| 1.39345 | 74.794883 | 0.95674471 | 0.00017038 |
| 1.3935 | 74.8024491 | 0.95657433 | 0.00017087 |
| 1.39355 | 74.8100189 | 0.95640346 | 0.00017137 |
| 1.3936 | 74.8175924 | 0.95623209 | 0.00017187 |
| 1.39365 | 74.8251696 | 0.95606023 | 0.00017237 |
| 1.3937 | 74.8327505 | 0.95588786 | 0.00017287 |
| 1.39375 | 74.840335 | 0.95571499 | 0.00017337 |
| 1.3938 | 74.8479233 | 0.95554162 | 0.00017387 |
| 1.39385 | 74.8555153 | 0.95536775 | 0.00017438 |
| 1.3939 | 74.863111 | 0.95519337 | 0.00017488 |
| 1.39395 | 74.8707105 | 0.95501849 | 0.00017539 |
| 1.394 | 74.8783136 | 0.95484309 | 0.0001759 |
| 1.39405 | 74.8859206 | 0.95466719 | 0.00017641 |
| 1.3941 | 74.8935312 | 0.95449078 | 0.00017693 |
| 1.39415 | 74.9011456 | 0.95431385 | 0.00017744 |
| 1.3942 | 74.9087637 | 0.95413641 | 0.00017795 |
| 1.39425 | 74.9163857 | 0.95395846 | 0.00017847 |
| 1.3943 | 74.9240113 | 0.95377999 | 0.00017899 |
| 1.39435 | 74.9316408 | 0.953601 | 0.00017951 |
| 1.3944 | 74.939274 | 0.95342149 | 0.00018003 |
| 1.39445 | 74.946911 | 0.95324146 | 0.00018055 |
| 1.3945 | 74.9545518 | 0.95306091 | 0.00018108 |
| 1.39455 | 74.9621963 | 0.95287983 | 0.0001816 |
| 1.3946 | 74.9698447 | 0.95269823 | 0.00018213 |
| 1.39465 | 74.9774969 | 0.9525161 | 0.00018266 |
| 1.3947 | 74.9851529 | 0.95233344 | 0.00018319 |
| 1.39475 | 74.9928127 | 0.95215026 | 0.00018372 |
| 1.3948 | 75.0004763 | 0.95196654 | 0.00018425 |
| 1.39485 | 75.0081438 | 0.95178229 | 0.00018478 |
| 1.3949 | 75.015815 | 0.9515975 | 0.00018532 |
| 1.39495 | 75.0234902 | 0.95141218 | 0.00018586 |
| 1.395 | 75.0311691 | 0.95122633 | 0.0001864 |
| 1.39505 | 75.038852 | 0.95103993 | 0.00018694 |
| 1.3951 | 75.0465386 | 0.95085299 | 0.00018748 |
| 1.39515 | 75.0542292 | 0.95066551 | 0.00018802 |
| 1.3952 | 75.0619236 | 0.95047749 | 0.00018857 |
| 1.39525 | 75.0696219 | 0.95028893 | 0.00018911 |
| 1.3953 | 75.0773241 | 0.95009981 | 0.00018966 |
| 1.39535 | 75.0850301 | 0.94991015 | 0.00019021 |
| 1.3954 | 75.0927401 | 0.94971995 | 0.00019076 |
| 1.39545 | 75.1004539 | 0.94952919 | 0.00019131 |
| 1.3955 | 75.1081717 | 0.94933787 | 0.00019187 |
| 1.39555 | 75.1158933 | 0.94914601 | 0.00019242 |
| 1.3956 | 75.1236189 | 0.94895358 | 0.00019298 |
| 1.39565 | 75.1313484 | 0.94876061 | 0.00019354 |
| 1.3957 | 75.1390818 | 0.94856707 | 0.0001941 |
| 1.39575 | 75.1468192 | 0.94837297 | 0.00019466 |
| 1.3958 | 75.1545605 | 0.94817831 | 0.00019522 |
| 1.39585 | 75.1623058 | 0.94798309 | 0.00019579 |
| 1.3959 | 75.170055 | 0.9477873 | 0.00019635 |
| 1.39595 | 75.1778082 | 0.94759095 | 0.00019692 |
| 1.396 | 75.1855653 | 0.94739403 | 0.00019749 |
| 1.39605 | 75.1933264 | 0.94719654 | 0.00019806 |
| 1.3961 | 75.2010915 | 0.94699847 | 0.00019863 |
| 1.39615 | 75.2088606 | 0.94679984 | 0.00019921 |
| 1.3962 | 75.2166337 | 0.94660063 | 0.00019978 |
| 1.39625 | 75.2244107 | 0.94640085 | 0.00020036 |
| 1.3963 | 75.2321918 | 0.94620048 | 0.00020094 |
| 1.39635 | 75.2399769 | 0.94599954 | 0.00020152 |
| 1.3964 | 75.247766 | 0.94579802 | 0.0002021 |
| 1.39645 | 75.2555591 | 0.94559592 | 0.00020269 |
| 1.3965 | 75.2633563 | 0.94539323 | 0.00020327 |
| 1.39655 | 75.2711575 | 0.94518995 | 0.00020386 |
| 1.3966 | 75.2789627 | 0.94498609 | 0.00020445 |
| 1.39665 | 75.286772 | 0.94478164 | 0.00020504 |
| 1.3967 | 75.2945853 | 0.9445766 | 0.00020563 |
| 1.39675 | 75.3024027 | 0.94437097 | 0.00020623 |
| 1.3968 | 75.3102242 | 0.94416475 | 0.00020682 |
| 1.39685 | 75.3180497 | 0.94395792 | 0.00020742 |
| 1.3969 | 75.3258794 | 0.94375051 | 0.00020802 |
| 1.39695 | 75.3337131 | 0.94354249 | 0.00020862 |
| 1.397 | 75.3415509 | 0.94333387 | 0.00020922 |
| 1.39705 | 75.3493928 | 0.94312465 | 0.00020982 |
| 1.3971 | 75.3572388 | 0.94291483 | 0.00021043 |
| 1.39715 | 75.3650889 | 0.9427044 | 0.00021104 |
| 1.3972 | 75.3729432 | 0.94249337 | 0.00021164 |
| 1.39725 | 75.3808016 | 0.94228172 | 0.00021225 |
| 1.3973 | 75.3886641 | 0.94206947 | 0.00021287 |
| 1.39735 | 75.3965307 | 0.9418566 | 0.00021348 |
| 1.3974 | 75.4044015 | 0.94164312 | 0.0002141 |
| 1.39745 | 75.4122765 | 0.94142903 | 0.00021471 |
| 1.3975 | 75.4201556 | 0.94121431 | 0.00021533 |
| 1.39755 | 75.4280389 | 0.94099898 | 0.00021595 |
| 1.3976 | 75.4359263 | 0.94078303 | 0.00021657 |
| 1.39765 | 75.443818 | 0.94056645 | 0.0002172 |
| 1.3977 | 75.4517138 | 0.94034926 | 0.00021782 |
| 1.39775 | 75.4596138 | 0.94013143 | 0.00021845 |
| 1.3978 | 75.4675181 | 0.93991298 | 0.00021908 |
| 1.39785 | 75.4754265 | 0.9396939 | 0.00021971 |
| 1.3979 | 75.4833391 | 0.93947418 | 0.00022035 |
| 1.39795 | 75.491256 | 0.93925384 | 0.00022098 |
| 1.398 | 75.4991771 | 0.93903286 | 0.00022162 |
| 1.39805 | 75.5071025 | 0.93881124 | 0.00022225 |
| 1.3981 | 75.515032 | 0.93858899 | 0.00022289 |
| 1.39815 | 75.5229659 | 0.9383661 | 0.00022354 |
| 1.3982 | 75.530904 | 0.93814256 | 0.00022418 |
| 1.39825 | 75.5388463 | 0.93791838 | 0.00022482 |
| 1.3983 | 75.5467929 | 0.93769356 | 0.00022547 |
| 1.39835 | 75.5547438 | 0.93746809 | 0.00022612 |
| 1.3984 | 75.562699 | 0.93724197 | 0.00022677 |
| 1.39845 | 75.5706585 | 0.9370152 | 0.00022742 |
| 1.3985 | 75.5786223 | 0.93678778 | 0.00022808 |
| 1.39855 | 75.5865904 | 0.9365597 | 0.00022873 |
| 1.3986 | 75.5945628 | 0.93633097 | 0.00022939 |
| 1.39865 | 75.6025395 | 0.93610158 | 0.00023005 |
| 1.3987 | 75.6105206 | 0.93587153 | 0.00023071 |
| 1.39875 | 75.618506 | 0.93564082 | 0.00023137 |
| 1.3988 | 75.6264957 | 0.93540944 | 0.00023204 |
| 1.39885 | 75.6344898 | 0.9351774 | 0.00023271 |
| 1.3989 | 75.6424882 | 0.9349447 | 0.00023337 |
| 1.39895 | 75.650491 | 0.93471132 | 0.00023405 |
| 1.399 | 75.6584982 | 0.93447728 | 0.00023472 |
| 1.39905 | 75.6665097 | 0.93424256 | 0.00023539 |
| 1.3991 | 75.6745257 | 0.93400717 | 0.00023607 |
| 1.39915 | 75.682546 | 0.9337711 | 0.00023675 |
| 1.3992 | 75.6905707 | 0.93353436 | 0.00023743 |
| 1.39925 | 75.6985999 | 0.93329693 | 0.00023811 |
| 1.3993 | 75.7066334 | 0.93305882 | 0.00023879 |
| 1.39935 | 75.7146714 | 0.93282003 | 0.00023948 |
| 1.3994 | 75.7227138 | 0.93258055 | 0.00024016 |
| 1.39945 | 75.7307607 | 0.93234039 | 0.00024085 |
| 1.3995 | 75.738812 | 0.93209954 | 0.00024154 |
| 1.39955 | 75.7468677 | 0.93185799 | 0.00024224 |
| 1.3996 | 75.754928 | 0.93161576 | 0.00024293 |
| 1.39965 | 75.7629926 | 0.93137282 | 0.00024363 |
| 1.3997 | 75.7710618 | 0.93112919 | 0.00024433 |
| 1.39975 | 75.7791354 | 0.93088486 | 0.00024503 |
| 1.3998 | 75.7872136 | 0.93063984 | 0.00024573 |
| 1.39985 | 75.7952962 | 0.9303941 | 0.00024644 |
| 1.3999 | 75.8033834 | 0.93014767 | 0.00024714 |
| 1.39995 | 75.811475 | 0.92990052 | 0.00024785 |
| 1.4 | 75.8195712 | 0.92965267 | 0.00024856 |
| 1.40005 | 75.8276719 | 0.92940411 | 0.00024927 |
| 1.4001 | 75.8357772 | 0.92915483 | 0.00024999 |
| 1.40015 | 75.8438869 | 0.92890485 | 0.00025071 |
| 1.4002 | 75.8520013 | 0.92865414 | 0.00025142 |
| 1.40025 | 75.8601202 | 0.92840272 | 0.00025214 |
| 1.4003 | 75.8682437 | 0.92815057 | 0.00025287 |
| 1.40035 | 75.8763717 | 0.9278977 | 0.00025359 |
| 1.4004 | 75.8845044 | 0.92764411 | 0.00025432 |
| 1.40045 | 75.8926416 | 0.9273898 | 0.00025505 |
| 1.4005 | 75.9007834 | 0.92713475 | 0.00025578 |
| 1.40055 | 75.9089299 | 0.92687897 | 0.00025651 |
| 1.4006 | 75.9170809 | 0.92662247 | 0.00025724 |
| 1.40065 | 75.9252366 | 0.92636522 | 0.00025798 |
| 1.4007 | 75.9333969 | 0.92610724 | 0.00025872 |
| 1.40075 | 75.9415619 | 0.92584853 | 0.00025946 |
| 1.4008 | 75.9497315 | 0.92558907 | 0.0002602 |
| 1.40085 | 75.9579058 | 0.92532887 | 0.00026094 |
| 1.4009 | 75.9660847 | 0.92506792 | 0.00026169 |
| 1.40095 | 75.9742683 | 0.92480623 | 0.00026244 |
| 1.401 | 75.9824566 | 0.92454379 | 0.00026319 |
| 1.40105 | 75.9906496 | 0.9242806 | 0.00026394 |
| 1.4011 | 75.9988472 | 0.92401666 | 0.0002647 |
| 1.40115 | 76.0070496 | 0.92375196 | 0.00026546 |
| 1.4012 | 76.0152567 | 0.92348651 | 0.00026621 |
| 1.40125 | 76.0234685 | 0.92322029 | 0.00026698 |
| 1.4013 | 76.0316851 | 0.92295332 | 0.00026774 |
| 1.40135 | 76.0399064 | 0.92268558 | 0.0002685 |
| 1.4014 | 76.0481324 | 0.92241708 | 0.00026927 |
| 1.40145 | 76.0563632 | 0.9221478 | 0.00027004 |
| 1.4015 | 76.0645988 | 0.92187776 | 0.00027081 |
| 1.40155 | 76.0728391 | 0.92160695 | 0.00027159 |
| 1.4016 | 76.0810842 | 0.92133537 | 0.00027236 |
| 1.40165 | 76.0893341 | 0.92106301 | 0.00027314 |
| 1.4017 | 76.0975888 | 0.92078987 | 0.00027392 |
| 1.40175 | 76.1058483 | 0.92051595 | 0.0002747 |
| 1.4018 | 76.1141127 | 0.92024125 | 0.00027549 |
| 1.40185 | 76.1223818 | 0.91996576 | 0.00027627 |
| 1.4019 | 76.1306558 | 0.91968949 | 0.00027706 |
| 1.40195 | 76.1389346 | 0.91941243 | 0.00027785 |
| 1.402 | 76.1472183 | 0.91913457 | 0.00027864 |
| 1.40205 | 76.1555068 | 0.91885593 | 0.00027944 |
| 1.4021 | 76.1638002 | 0.91857649 | 0.00028024 |
| 1.40215 | 76.1720985 | 0.91829625 | 0.00028104 |
| 1.4022 | 76.1804017 | 0.91801521 | 0.00028184 |
| 1.40225 | 76.1887098 | 0.91773338 | 0.00028264 |
| 1.4023 | 76.1970227 | 0.91745073 | 0.00028345 |
| 1.40235 | 76.2053406 | 0.91716728 | 0.00028426 |
| 1.4024 | 76.2136634 | 0.91688303 | 0.00028507 |
| 1.40245 | 76.2219911 | 0.91659796 | 0.00028588 |
| 1.4025 | 76.2303238 | 0.91631208 | 0.0002867 |
| 1.40255 | 76.2386614 | 0.91602538 | 0.00028751 |
| 1.4026 | 76.247004 | 0.91573787 | 0.00028833 |
| 1.40265 | 76.2553515 | 0.91544954 | 0.00028915 |
| 1.4027 | 76.2637041 | 0.91516038 | 0.00028998 |
| 1.40275 | 76.2720616 | 0.9148704 | 0.0002908 |
| 1.4028 | 76.2804241 | 0.9145796 | 0.00029163 |
| 1.40285 | 76.2887915 | 0.91428797 | 0.00029246 |
| 1.4029 | 76.2971641 | 0.9139955 | 0.0002933 |
| 1.40295 | 76.3055416 | 0.91370221 | 0.00029413 |
| 1.403 | 76.3139241 | 0.91340807 | 0.00029497 |
| 1.40305 | 76.3223117 | 0.9131131 | 0.00029581 |
| 1.4031 | 76.3307044 | 0.9128173 | 0.00029665 |
| 1.40315 | 76.3391021 | 0.91252064 | 0.0002975 |
| 1.4032 | 76.3475049 | 0.91222315 | 0.00029834 |
| 1.40325 | 76.3559127 | 0.9119248 | 0.00029919 |
| 1.4033 | 76.3643256 | 0.91162561 | 0.00030004 |
| 1.40335 | 76.3727437 | 0.91132557 | 0.0003009 |
| 1.4034 | 76.3811668 | 0.91102467 | 0.00030175 |
| 1.40345 | 76.389595 | 0.91072291 | 0.00030261 |
| 1.4035 | 76.3980284 | 0.9104203 | 0.00030347 |
| 1.40355 | 76.4064669 | 0.91011683 | 0.00030434 |
| 1.4036 | 76.4149106 | 0.90981249 | 0.0003052 |
| 1.40365 | 76.4233594 | 0.90950729 | 0.00030607 |
| 1.4037 | 76.4318133 | 0.90920122 | 0.00030694 |
| 1.40375 | 76.4402725 | 0.90889428 | 0.00030781 |
| 1.4038 | 76.4487368 | 0.90858647 | 0.00030869 |
| 1.40385 | 76.4572063 | 0.90827778 | 0.00030957 |
| 1.4039 | 76.465681 | 0.90796821 | 0.00031045 |
| 1.40395 | 76.4741609 | 0.90765777 | 0.00031133 |
| 1.404 | 76.482646 | 0.90734644 | 0.00031221 |
| 1.40405 | 76.4911364 | 0.90703423 | 0.0003131 |
| 1.4041 | 76.499632 | 0.90672113 | 0.00031399 |
| 1.40415 | 76.5081329 | 0.90640714 | 0.00031488 |
| 1.4042 | 76.516639 | 0.90609226 | 0.00031578 |
| 1.40425 | 76.5251504 | 0.90577649 | 0.00031667 |
| 1.4043 | 76.533667 | 0.90545981 | 0.00031757 |
| 1.40435 | 76.542189 | 0.90514224 | 0.00031847 |
| 1.4044 | 76.5507163 | 0.90482377 | 0.00031938 |
| 1.40445 | 76.5592488 | 0.90450439 | 0.00032028 |
| 1.4045 | 76.5677867 | 0.90418411 | 0.00032119 |
| 1.40455 | 76.5763299 | 0.90386292 | 0.0003221 |
| 1.4046 | 76.5848785 | 0.90354081 | 0.00032302 |
| 1.40465 | 76.5934324 | 0.90321779 | 0.00032394 |
| 1.4047 | 76.6019917 | 0.90289386 | 0.00032485 |
| 1.40475 | 76.6105563 | 0.902569 | 0.00032578 |
| 1.4048 | 76.6191264 | 0.90224323 | 0.0003267 |
| 1.40485 | 76.6277018 | 0.90191653 | 0.00032763 |
| 1.4049 | 76.6362826 | 0.9015889 | 0.00032856 |
| 1.40495 | 76.6448688 | 0.90126035 | 0.00032949 |
| 1.405 | 76.6534605 | 0.90093086 | 0.00033042 |
| 1.40505 | 76.6620576 | 0.90060044 | 0.00033136 |
| 1.4051 | 76.6706601 | 0.90026908 | 0.0003323 |
| 1.40515 | 76.6792681 | 0.89993678 | 0.00033324 |
| 1.4052 | 76.6878815 | 0.89960354 | 0.00033418 |
| 1.40525 | 76.6965005 | 0.89926936 | 0.00033513 |
| 1.4053 | 76.7051249 | 0.89893423 | 0.00033608 |
| 1.40535 | 76.7137548 | 0.89859815 | 0.00033703 |
| 1.4054 | 76.7223902 | 0.89826112 | 0.00033799 |
| 1.40545 | 76.7310312 | 0.89792313 | 0.00033894 |
| 1.4055 | 76.7396776 | 0.89758418 | 0.0003399 |
| 1.40555 | 76.7483297 | 0.89724428 | 0.00034087 |
| 1.4056 | 76.7569872 | 0.89690341 | 0.00034183 |
| 1.40565 | 76.7656503 | 0.89656158 | 0.0003428 |
| 1.4057 | 76.774319 | 0.89621878 | 0.00034377 |
| 1.40575 | 76.7829933 | 0.89587501 | 0.00034474 |
| 1.4058 | 76.7916732 | 0.89553026 | 0.00034572 |
| 1.40585 | 76.8003587 | 0.89518454 | 0.0003467 |
| 1.4059 | 76.8090498 | 0.89483785 | 0.00034768 |
| 1.40595 | 76.8177465 | 0.89449017 | 0.00034866 |
| 1.406 | 76.8264489 | 0.8941415 | 0.00034965 |
| 1.40605 | 76.8351569 | 0.89379186 | 0.00035064 |
| 1.4061 | 76.8438706 | 0.89344122 | 0.00035163 |
| 1.40615 | 76.85259 | 0.89308959 | 0.00035262 |
| 1.4062 | 76.861315 | 0.89273696 | 0.00035362 |
| 1.40625 | 76.8700457 | 0.89238334 | 0.00035462 |
| 1.4063 | 76.8787822 | 0.89202872 | 0.00035562 |
| 1.40635 | 76.8875243 | 0.89167309 | 0.00035663 |
| 1.4064 | 76.8962722 | 0.89131646 | 0.00035764 |
| 1.40645 | 76.9050259 | 0.89095883 | 0.00035865 |
| 1.4065 | 76.9137852 | 0.89060018 | 0.00035966 |
| 1.40655 | 76.9225504 | 0.89024052 | 0.00036068 |
| 1.4066 | 76.9313213 | 0.88987984 | 0.0003617 |
| 1.40665 | 76.940098 | 0.88951814 | 0.00036272 |
| 1.4067 | 76.9488806 | 0.88915542 | 0.00036374 |
| 1.40675 | 76.9576689 | 0.88879168 | 0.00036477 |
| 1.4068 | 76.966463 | 0.88842691 | 0.0003658 |
| 1.40685 | 76.975263 | 0.8880611 | 0.00036683 |
| 1.4069 | 76.9840688 | 0.88769427 | 0.00036787 |
| 1.40695 | 76.9928805 | 0.8873264 | 0.00036891 |
| 1.407 | 77.0016981 | 0.88695749 | 0.00036995 |
| 1.40705 | 77.0105215 | 0.88658754 | 0.00037099 |
| 1.4071 | 77.0193509 | 0.88621655 | 0.00037204 |
| 1.40715 | 77.0281861 | 0.8858445 | 0.00037309 |
| 1.4072 | 77.0370273 | 0.88547141 | 0.00037414 |
| 1.40725 | 77.0458744 | 0.88509727 | 0.0003752 |
| 1.4073 | 77.0547274 | 0.88472207 | 0.00037626 |
| 1.40735 | 77.0635864 | 0.88434581 | 0.00037732 |
| 1.4074 | 77.0724514 | 0.88396849 | 0.00037838 |
| 1.40745 | 77.0813223 | 0.88359011 | 0.00037945 |
| 1.4075 | 77.0901992 | 0.88321065 | 0.00038052 |
| 1.40755 | 77.0990822 | 0.88283013 | 0.00038159 |
| 1.4076 | 77.1079711 | 0.88244854 | 0.00038267 |
| 1.40765 | 77.1168661 | 0.88206587 | 0.00038375 |
| 1.4077 | 77.1257671 | 0.88168212 | 0.00038483 |
| 1.40775 | 77.1346742 | 0.88129729 | 0.00038591 |
| 1.4078 | 77.1435873 | 0.88091138 | 0.000387 |
| 1.40785 | 77.1525065 | 0.88052437 | 0.00038809 |
| 1.4079 | 77.1614319 | 0.88013628 | 0.00038919 |
| 1.40795 | 77.1703633 | 0.87974709 | 0.00039028 |
| 1.408 | 77.1793008 | 0.87935681 | 0.00039138 |
| 1.40805 | 77.1882445 | 0.87896543 | 0.00039248 |
| 1.4081 | 77.1971943 | 0.87857295 | 0.00039359 |
| 1.40815 | 77.2061503 | 0.87817936 | 0.0003947 |
| 1.4082 | 77.2151124 | 0.87778466 | 0.00039581 |
| 1.40825 | 77.2240807 | 0.87738885 | 0.00039692 |
| 1.4083 | 77.2330552 | 0.87699193 | 0.00039804 |
| 1.40835 | 77.2420359 | 0.87659389 | 0.00039916 |
| 1.4084 | 77.2510229 | 0.87619472 | 0.00040028 |
| 1.40845 | 77.2600161 | 0.87579444 | 0.00040141 |
| 1.4085 | 77.2690155 | 0.87539303 | 0.00040254 |
| 1.40855 | 77.2780212 | 0.87499049 | 0.00040367 |
| 1.4086 | 77.2870331 | 0.87458682 | 0.00040481 |
| 1.40865 | 77.2960514 | 0.87418201 | 0.00040595 |
| 1.4087 | 77.3050759 | 0.87377606 | 0.00040709 |
| 1.40875 | 77.3141068 | 0.87336897 | 0.00040823 |
| 1.4088 | 77.323144 | 0.87296074 | 0.00040938 |
| 1.40885 | 77.3321875 | 0.87255136 | 0.00041053 |
| 1.4089 | 77.3412374 | 0.87214083 | 0.00041169 |
| 1.40895 | 77.3502936 | 0.87172914 | 0.00041284 |
| 1.409 | 77.3593563 | 0.87131629 | 0.000414 |
| 1.40905 | 77.3684253 | 0.87090229 | 0.00041517 |
| 1.4091 | 77.3775008 | 0.87048712 | 0.00041633 |
| 1.40915 | 77.3865826 | 0.87007079 | 0.0004175 |
| 1.4092 | 77.3956709 | 0.86965328 | 0.00041868 |
| 1.40925 | 77.4047657 | 0.86923461 | 0.00041985 |
| 1.4093 | 77.4138669 | 0.86881476 | 0.00042103 |
| 1.40935 | 77.4229746 | 0.86839372 | 0.00042221 |
| 1.4094 | 77.4320888 | 0.86797151 | 0.0004234 |
| 1.40945 | 77.4412095 | 0.86754811 | 0.00042459 |
| 1.4095 | 77.4503367 | 0.86712352 | 0.00042578 |
| 1.40955 | 77.4594705 | 0.86669774 | 0.00042698 |
| 1.4096 | 77.4686108 | 0.86627076 | 0.00042818 |
| 1.40965 | 77.4777577 | 0.86584259 | 0.00042938 |
| 1.4097 | 77.4869111 | 0.86541321 | 0.00043058 |
| 1.40975 | 77.4960712 | 0.86498263 | 0.00043179 |
| 1.4098 | 77.5052379 | 0.86455084 | 0.000433 |
| 1.40985 | 77.5144111 | 0.86411783 | 0.00043422 |
| 1.4099 | 77.5235911 | 0.86368362 | 0.00043543 |
| 1.40995 | 77.5327776 | 0.86324818 | 0.00043666 |
| 1.41 | 77.5419709 | 0.86281153 | 0.00043788 |
| 1.41005 | 77.5511708 | 0.86237365 | 0.00043911 |
| 1.4101 | 77.5603774 | 0.86193454 | 0.00044034 |
| 1.41015 | 77.5695907 | 0.8614942 | 0.00044157 |
| 1.4102 | 77.5788108 | 0.86105263 | 0.00044281 |
| 1.41025 | 77.5880376 | 0.86060982 | 0.00044405 |
| 1.4103 | 77.5972711 | 0.86016576 | 0.0004453 |
| 1.41035 | 77.6065115 | 0.85972047 | 0.00044655 |
| 1.4104 | 77.6157586 | 0.85927392 | 0.0004478 |
| 1.41045 | 77.6250125 | 0.85882612 | 0.00044905 |
| 1.4105 | 77.6342732 | 0.85837707 | 0.00045031 |
| 1.41055 | 77.6435408 | 0.85792676 | 0.00045157 |
| 1.4106 | 77.6528152 | 0.85747519 | 0.00045284 |
| 1.41065 | 77.6620964 | 0.85702236 | 0.0004541 |
| 1.4107 | 77.6713846 | 0.85656825 | 0.00045538 |
| 1.41075 | 77.6806796 | 0.85611288 | 0.00045665 |
| 1.4108 | 77.6899816 | 0.85565623 | 0.00045793 |
| 1.41085 | 77.6992905 | 0.8551983 | 0.00045921 |
| 1.4109 | 77.7086063 | 0.85473909 | 0.0004605 |
| 1.41095 | 77.7179291 | 0.85427859 | 0.00046178 |
| 1.411 | 77.7272588 | 0.85381681 | 0.00046308 |
| 1.41105 | 77.7365956 | 0.85335373 | 0.00046437 |
| 1.4111 | 77.7459393 | 0.85288936 | 0.00046567 |
| 1.41115 | 77.7552901 | 0.85242369 | 0.00046697 |
| 1.4112 | 77.7646479 | 0.85195671 | 0.00046828 |
| 1.41125 | 77.7740127 | 0.85148843 | 0.00046959 |
| 1.4113 | 77.7833847 | 0.85101884 | 0.0004709 |
| 1.41135 | 77.7927637 | 0.85054794 | 0.00047222 |
| 1.4114 | 77.8021498 | 0.85007572 | 0.00047354 |
| 1.41145 | 77.811543 | 0.84960218 | 0.00047486 |
| 1.4115 | 77.8209434 | 0.84912732 | 0.00047619 |
| 1.41155 | 77.8303509 | 0.84865112 | 0.00047752 |
| 1.4116 | 77.8397656 | 0.8481736 | 0.00047886 |
| 1.41165 | 77.8491875 | 0.84769474 | 0.0004802 |
| 1.4117 | 77.8586165 | 0.84721455 | 0.00048154 |
| 1.41175 | 77.8680528 | 0.84673301 | 0.00048288 |
| 1.4118 | 77.8774964 | 0.84625013 | 0.00048423 |
| 1.41185 | 77.8869471 | 0.8457659 | 0.00048558 |
| 1.4119 | 77.8964052 | 0.84528031 | 0.00048694 |
| 1.41195 | 77.9058705 | 0.84479337 | 0.0004883 |
| 1.412 | 77.9153432 | 0.84430507 | 0.00048966 |
| 1.41205 | 77.9248231 | 0.84381541 | 0.00049103 |
| 1.4121 | 77.9343104 | 0.84332438 | 0.0004924 |
| 1.41215 | 77.9438051 | 0.84283198 | 0.00049378 |
| 1.4122 | 77.9533071 | 0.8423382 | 0.00049515 |
| 1.41225 | 77.9628165 | 0.84184305 | 0.00049654 |
| 1.4123 | 77.9723333 | 0.84134651 | 0.00049792 |
| 1.41235 | 77.9818576 | 0.84084859 | 0.00049931 |
| 1.4124 | 77.9913893 | 0.84034928 | 0.0005007 |
| 1.41245 | 78.0009284 | 0.83984858 | 0.0005021 |
| 1.4125 | 78.010475 | 0.83934648 | 0.0005035 |
| 1.41255 | 78.0200291 | 0.83884297 | 0.00050491 |
| 1.4126 | 78.0295908 | 0.83833807 | 0.00050631 |
| 1.41265 | 78.03916 | 0.83783176 | 0.00050772 |
| 1.4127 | 78.0487367 | 0.83732403 | 0.00050914 |
| 1.41275 | 78.058321 | 0.83681489 | 0.00051056 |
| 1.4128 | 78.0679128 | 0.83630433 | 0.00051198 |
| 1.41285 | 78.0775123 | 0.83579235 | 0.00051341 |
| 1.4129 | 78.0871194 | 0.83527894 | 0.00051484 |
| 1.41295 | 78.0967342 | 0.8347641 | 0.00051628 |
| 1.413 | 78.1063566 | 0.83424782 | 0.00051771 |
| 1.41305 | 78.1159866 | 0.83373011 | 0.00051916 |
| 1.4131 | 78.1256244 | 0.83321095 | 0.0005206 |
| 1.41315 | 78.1352699 | 0.83269035 | 0.00052205 |
| 1.4132 | 78.1449231 | 0.8321683 | 0.00052351 |
| 1.41325 | 78.1545841 | 0.83164479 | 0.00052496 |
| 1.4133 | 78.1642529 | 0.83111983 | 0.00052643 |
| 1.41335 | 78.1739294 | 0.8305934 | 0.00052789 |
| 1.4134 | 78.1836138 | 0.83006551 | 0.00052936 |
| 1.41345 | 78.193306 | 0.82953615 | 0.00053083 |
| 1.4135 | 78.203006 | 0.82900532 | 0.00053231 |
| 1.41355 | 78.2127139 | 0.82847301 | 0.00053379 |
| 1.4136 | 78.2224297 | 0.82793921 | 0.00053528 |
| 1.41365 | 78.2321535 | 0.82740393 | 0.00053677 |
| 1.4137 | 78.2418851 | 0.82686717 | 0.00053826 |
| 1.41375 | 78.2516247 | 0.82632891 | 0.00053976 |
| 1.4138 | 78.2613722 | 0.82578915 | 0.00054126 |
| 1.41385 | 78.2711278 | 0.82524789 | 0.00054276 |
| 1.4139 | 78.2808913 | 0.82470512 | 0.00054427 |
| 1.41395 | 78.2906629 | 0.82416085 | 0.00054579 |
| 1.414 | 78.3004425 | 0.82361506 | 0.0005473 |
| 1.41405 | 78.3102302 | 0.82306776 | 0.00054883 |
| 1.4141 | 78.320026 | 0.82251893 | 0.00055035 |
| 1.41415 | 78.3298298 | 0.82196858 | 0.00055188 |
| 1.4142 | 78.3396419 | 0.8214167 | 0.00055341 |
| 1.41425 | 78.349462 | 0.82086329 | 0.00055495 |
| 1.4143 | 78.3592903 | 0.82030833 | 0.00055649 |
| 1.41435 | 78.3691269 | 0.81975184 | 0.00055804 |
| 1.4144 | 78.3789716 | 0.8191938 | 0.00055959 |
| 1.41445 | 78.3888246 | 0.81863421 | 0.00056114 |
| 1.4145 | 78.3986858 | 0.81807307 | 0.0005627 |
| 1.41455 | 78.4085553 | 0.81751036 | 0.00056427 |
| 1.4146 | 78.4184331 | 0.8169461 | 0.00056583 |
| 1.41465 | 78.4283192 | 0.81638027 | 0.0005674 |
| 1.4147 | 78.4382136 | 0.81581286 | 0.00056898 |
| 1.41475 | 78.4481164 | 0.81524389 | 0.00057056 |
| 1.4148 | 78.4580276 | 0.81467333 | 0.00057214 |
| 1.41485 | 78.4679472 | 0.81410119 | 0.00057373 |
| 1.4149 | 78.4778752 | 0.81352746 | 0.00057532 |
| 1.41495 | 78.4878116 | 0.81295214 | 0.00057692 |
| 1.415 | 78.4977566 | 0.81237522 | 0.00057852 |
| 1.41505 | 78.50771 | 0.81179671 | 0.00058012 |
| 1.4151 | 78.5176719 | 0.81121659 | 0.00058173 |
| 1.41515 | 78.5276423 | 0.81063486 | 0.00058334 |
| 1.4152 | 78.5376214 | 0.81005151 | 0.00058496 |
| 1.41525 | 78.547609 | 0.80946655 | 0.00058658 |
| 1.4153 | 78.5576051 | 0.80887997 | 0.00058821 |
| 1.41535 | 78.56761 | 0.80829176 | 0.00058984 |
| 1.4154 | 78.5776234 | 0.80770192 | 0.00059147 |
| 1.41545 | 78.5876456 | 0.80711044 | 0.00059311 |
| 1.4155 | 78.5976764 | 0.80651733 | 0.00059476 |
| 1.41555 | 78.6077159 | 0.80592257 | 0.00059641 |
| 1.4156 | 78.6177642 | 0.80532617 | 0.00059806 |
| 1.41565 | 78.6278213 | 0.80472811 | 0.00059971 |
| 1.4157 | 78.6378871 | 0.8041284 | 0.00060138 |
| 1.41575 | 78.6479617 | 0.80352702 | 0.00060304 |
| 1.4158 | 78.6580452 | 0.80292398 | 0.00060471 |
| 1.41585 | 78.6681375 | 0.80231927 | 0.00060639 |
| 1.4159 | 78.6782387 | 0.80171289 | 0.00060806 |
| 1.41595 | 78.6883488 | 0.80110482 | 0.00060975 |
| 1.416 | 78.6984678 | 0.80049507 | 0.00061144 |
| 1.41605 | 78.7085958 | 0.79988364 | 0.00061313 |
| 1.4161 | 78.7187327 | 0.79927051 | 0.00061482 |
| 1.41615 | 78.7288787 | 0.79865569 | 0.00061653 |
| 1.4162 | 78.7390337 | 0.79803916 | 0.00061823 |
| 1.41625 | 78.7491977 | 0.79742093 | 0.00061994 |
| 1.4163 | 78.7593708 | 0.79680099 | 0.00062166 |
| 1.41635 | 78.769553 | 0.79617933 | 0.00062338 |
| 1.4164 | 78.7797443 | 0.79555595 | 0.0006251 |
| 1.41645 | 78.7899447 | 0.79493085 | 0.00062683 |
| 1.4165 | 78.8001544 | 0.79430403 | 0.00062856 |
| 1.41655 | 78.8103732 | 0.79367546 | 0.0006303 |
| 1.4166 | 78.8206012 | 0.79304516 | 0.00063204 |
| 1.41665 | 78.8308385 | 0.79241312 | 0.00063379 |
| 1.4167 | 78.8410851 | 0.79177933 | 0.00063554 |
| 1.41675 | 78.851341 | 0.79114379 | 0.0006373 |
| 1.4168 | 78.8616062 | 0.79050649 | 0.00063906 |
| 1.41685 | 78.8718807 | 0.78986744 | 0.00064082 |
| 1.4169 | 78.8821646 | 0.78922661 | 0.00064259 |
| 1.41695 | 78.8924579 | 0.78858402 | 0.00064437 |
| 1.417 | 78.9027607 | 0.78793965 | 0.00064615 |
| 1.41705 | 78.9130729 | 0.7872935 | 0.00064793 |
| 1.4171 | 78.9233945 | 0.78664556 | 0.00064972 |
| 1.41715 | 78.9337257 | 0.78599584 | 0.00065152 |
| 1.4172 | 78.9440664 | 0.78534432 | 0.00065332 |
| 1.41725 | 78.9544167 | 0.78469101 | 0.00065512 |
| 1.4173 | 78.9647766 | 0.78403589 | 0.00065693 |
| 1.41735 | 78.975146 | 0.78337896 | 0.00065874 |
| 1.4174 | 78.9855252 | 0.78272022 | 0.00066056 |
| 1.41745 | 78.995914 | 0.78205966 | 0.00066238 |
| 1.4175 | 79.0063124 | 0.78139728 | 0.00066421 |
| 1.41755 | 79.0167206 | 0.78073307 | 0.00066604 |
| 1.4176 | 79.0271386 | 0.78006703 | 0.00066788 |
| 1.41765 | 79.0375663 | 0.77939915 | 0.00066972 |
| 1.4177 | 79.0480039 | 0.77872943 | 0.00067157 |
| 1.41775 | 79.0584513 | 0.77805787 | 0.00067342 |
| 1.4178 | 79.0689085 | 0.77738445 | 0.00067528 |
| 1.41785 | 79.0793756 | 0.77670917 | 0.00067714 |
| 1.4179 | 79.0898527 | 0.77603203 | 0.000679 |
| 1.41795 | 79.1003397 | 0.77535303 | 0.00068088 |
| 1.418 | 79.1108367 | 0.77467215 | 0.00068275 |
| 1.41805 | 79.1213437 | 0.7739894 | 0.00068463 |
| 1.4181 | 79.1318607 | 0.77330477 | 0.00068652 |
| 1.41815 | 79.1423878 | 0.77261825 | 0.00068841 |
| 1.4182 | 79.152925 | 0.77192983 | 0.00069031 |
| 1.41825 | 79.1634723 | 0.77123953 | 0.00069221 |
| 1.4183 | 79.1740297 | 0.77054732 | 0.00069412 |
| 1.41835 | 79.1845974 | 0.7698532 | 0.00069603 |
| 1.4184 | 79.1951752 | 0.76915717 | 0.00069794 |
| 1.41845 | 79.2057633 | 0.76845923 | 0.00069987 |
| 1.4185 | 79.2163617 | 0.76775936 | 0.00070179 |
| 1.41855 | 79.2269703 | 0.76705757 | 0.00070372 |
| 1.4186 | 79.2375893 | 0.76635384 | 0.00070566 |
| 1.41865 | 79.2482187 | 0.76564818 | 0.0007076 |
| 1.4187 | 79.2588585 | 0.76494058 | 0.00070955 |
| 1.41875 | 79.2695087 | 0.76423103 | 0.0007115 |
| 1.4188 | 79.2801693 | 0.76351952 | 0.00071346 |
| 1.41885 | 79.2908404 | 0.76280606 | 0.00071542 |
| 1.4189 | 79.3015221 | 0.76209064 | 0.00071739 |
| 1.41895 | 79.3122143 | 0.76137325 | 0.00071936 |
| 1.419 | 79.3229171 | 0.76065388 | 0.00072134 |
| 1.41905 | 79.3336305 | 0.75993254 | 0.00072333 |
| 1.4191 | 79.3443545 | 0.75920921 | 0.00072532 |
| 1.41915 | 79.3550893 | 0.7584839 | 0.00072731 |
| 1.4192 | 79.3658347 | 0.75775659 | 0.00072931 |
| 1.41925 | 79.3765909 | 0.75702728 | 0.00073131 |
| 1.4193 | 79.3873578 | 0.75629597 | 0.00073332 |
| 1.41935 | 79.3981356 | 0.75556264 | 0.00073534 |
| 1.4194 | 79.4089242 | 0.75482731 | 0.00073736 |
| 1.41945 | 79.4197237 | 0.75408995 | 0.00073938 |
| 1.4195 | 79.4305341 | 0.75335056 | 0.00074142 |
| 1.41955 | 79.4413554 | 0.75260915 | 0.00074345 |
| 1.4196 | 79.4521877 | 0.7518657 | 0.00074549 |
| 1.41965 | 79.4630311 | 0.7511202 | 0.00074754 |
| 1.4197 | 79.4738854 | 0.75037266 | 0.00074959 |
| 1.41975 | 79.4847509 | 0.74962307 | 0.00075165 |
| 1.4198 | 79.4956274 | 0.74887142 | 0.00075372 |
| 1.41985 | 79.5065151 | 0.7481177 | 0.00075578 |
| 1.4199 | 79.517414 | 0.74736192 | 0.00075786 |
| 1.41995 | 79.5283241 | 0.74660406 | 0.00075994 |
| 1.42 | 79.5392455 | 0.74584412 | 0.00076202 |
| 1.42005 | 79.5501781 | 0.74508209 | 0.00076411 |
| 1.4201 | 79.5611221 | 0.74431798 | 0.00076621 |
| 1.42015 | 79.5720774 | 0.74355177 | 0.00076831 |
| 1.4202 | 79.5830441 | 0.74278346 | 0.00077042 |
| 1.42025 | 79.5940222 | 0.74201304 | 0.00077253 |
| 1.4203 | 79.6050118 | 0.7412405 | 0.00077465 |
| 1.42035 | 79.616013 | 0.74046585 | 0.00077678 |
| 1.4204 | 79.6270256 | 0.73968907 | 0.00077891 |
| 1.42045 | 79.6380498 | 0.73891017 | 0.00078104 |
| 1.4205 | 79.6490857 | 0.73812913 | 0.00078318 |
| 1.42055 | 79.6601332 | 0.73734595 | 0.00078533 |
| 1.4206 | 79.6711923 | 0.73656062 | 0.00078748 |
| 1.42065 | 79.6822632 | 0.73577314 | 0.00078964 |
| 1.4207 | 79.6933459 | 0.7349835 | 0.0007918 |
| 1.42075 | 79.7044404 | 0.7341917 | 0.00079397 |
| 1.4208 | 79.7155467 | 0.73339773 | 0.00079615 |
| 1.42085 | 79.7266649 | 0.73260158 | 0.00079833 |
| 1.4209 | 79.7377949 | 0.73180325 | 0.00080051 |
| 1.42095 | 79.748937 | 0.73100274 | 0.00080271 |
| 1.421 | 79.760091 | 0.73020003 | 0.0008049 |
| 1.42105 | 79.7712571 | 0.72939513 | 0.00080711 |
| 1.4211 | 79.7824353 | 0.72858802 | 0.00080932 |
| 1.42115 | 79.7936255 | 0.72777871 | 0.00081153 |
| 1.4212 | 79.804828 | 0.72696717 | 0.00081375 |
| 1.42125 | 79.8160426 | 0.72615342 | 0.00081598 |
| 1.4213 | 79.8272694 | 0.72533744 | 0.00081821 |
| 1.42135 | 79.8385085 | 0.72451923 | 0.00082045 |
| 1.4214 | 79.84976 | 0.72369878 | 0.0008227 |
| 1.42145 | 79.8610237 | 0.72287608 | 0.00082495 |
| 1.4215 | 79.8722999 | 0.72205113 | 0.0008272 |
| 1.42155 | 79.8835885 | 0.72122393 | 0.00082947 |
| 1.4216 | 79.8948896 | 0.72039446 | 0.00083173 |
| 1.42165 | 79.9062033 | 0.71956273 | 0.00083401 |
| 1.4217 | 79.9175294 | 0.71872872 | 0.00083629 |
| 1.42175 | 79.9288682 | 0.71789243 | 0.00083857 |
| 1.4218 | 79.9402197 | 0.71705386 | 0.00084087 |
| 1.42185 | 79.9515838 | 0.71621299 | 0.00084316 |
| 1.4219 | 79.9629606 | 0.71536983 | 0.00084547 |
| 1.42195 | 79.9743503 | 0.71452436 | 0.00084778 |
| 1.422 | 79.9857527 | 0.71367658 | 0.0008501 |
| 1.42205 | 79.9971681 | 0.71282649 | 0.00085242 |
| 1.4221 | 80.0085963 | 0.71197407 | 0.00085475 |
| 1.42215 | 80.0200375 | 0.71111932 | 0.00085708 |
| 1.4222 | 80.0314917 | 0.71026224 | 0.00085942 |
| 1.42225 | 80.0429589 | 0.70940282 | 0.00086177 |
| 1.4223 | 80.0544392 | 0.70854105 | 0.00086412 |
| 1.42235 | 80.0659327 | 0.70767693 | 0.00086648 |
| 1.4224 | 80.0774393 | 0.70681045 | 0.00086885 |
| 1.42245 | 80.0889592 | 0.7059416 | 0.00087122 |
| 1.4225 | 80.1004923 | 0.70507039 | 0.0008736 |
| 1.42255 | 80.1120387 | 0.70419679 | 0.00087598 |
| 1.4226 | 80.1235985 | 0.70332081 | 0.00087837 |
| 1.42265 | 80.1351718 | 0.70244244 | 0.00088077 |
| 1.4227 | 80.1467584 | 0.70156168 | 0.00088317 |
| 1.42275 | 80.1583586 | 0.70067851 | 0.00088558 |
| 1.4228 | 80.1699724 | 0.69979293 | 0.00088799 |
| 1.42285 | 80.1815997 | 0.69890494 | 0.00089042 |
| 1.4229 | 80.1932407 | 0.69801452 | 0.00089284 |
| 1.42295 | 80.2048954 | 0.69712168 | 0.00089528 |
| 1.423 | 80.2165638 | 0.6962264 | 0.00089772 |
| 1.42305 | 80.2282461 | 0.69532868 | 0.00090017 |
| 1.4231 | 80.2399421 | 0.69442852 | 0.00090262 |
| 1.42315 | 80.2516521 | 0.6935259 | 0.00090508 |
| 1.4232 | 80.263376 | 0.69262082 | 0.00090755 |
| 1.42325 | 80.275114 | 0.69171327 | 0.00091002 |
| 1.4233 | 80.2868659 | 0.69080325 | 0.0009125 |
| 1.42335 | 80.298632 | 0.68989076 | 0.00091498 |
| 1.4234 | 80.3104122 | 0.68897577 | 0.00091748 |
| 1.42345 | 80.3222067 | 0.6880583 | 0.00091998 |
| 1.4235 | 80.3340153 | 0.68713832 | 0.00092248 |
| 1.42355 | 80.3458383 | 0.68621584 | 0.00092499 |
| 1.4236 | 80.3576757 | 0.68529085 | 0.00092751 |
| 1.42365 | 80.3695274 | 0.68436334 | 0.00093004 |
| 1.4237 | 80.3813937 | 0.6834333 | 0.00093257 |
| 1.42375 | 80.3932744 | 0.68250073 | 0.00093511 |
| 1.4238 | 80.4051697 | 0.68156563 | 0.00093765 |
| 1.42385 | 80.4170796 | 0.68062798 | 0.0009402 |
| 1.4239 | 80.4290043 | 0.67968777 | 0.00094276 |
| 1.42395 | 80.4409436 | 0.67874501 | 0.00094533 |
| 1.424 | 80.4528978 | 0.67779969 | 0.0009479 |
| 1.42405 | 80.4648668 | 0.67685179 | 0.00095048 |
| 1.4241 | 80.4768507 | 0.67590131 | 0.00095306 |
| 1.42415 | 80.4888495 | 0.67494825 | 0.00095565 |
| 1.4242 | 80.5008634 | 0.6739926 | 0.00095825 |
| 1.42425 | 80.5128924 | 0.67303435 | 0.00096086 |
| 1.4243 | 80.5249365 | 0.67207349 | 0.00096347 |
| 1.42435 | 80.5369957 | 0.67111002 | 0.00096609 |
| 1.4244 | 80.5490703 | 0.67014393 | 0.00096871 |
| 1.42445 | 80.5611601 | 0.66917522 | 0.00097135 |
| 1.4245 | 80.5732653 | 0.66820387 | 0.00097399 |
| 1.42455 | 80.5853859 | 0.66722988 | 0.00097663 |
| 1.4246 | 80.597522 | 0.66625325 | 0.00097929 |
| 1.42465 | 80.6096736 | 0.66527396 | 0.00098195 |
| 1.4247 | 80.6218409 | 0.66429202 | 0.00098461 |
| 1.42475 | 80.6340238 | 0.6633074 | 0.00098729 |
| 1.4248 | 80.6462224 | 0.66232011 | 0.00098997 |
| 1.42485 | 80.6584369 | 0.66133014 | 0.00099266 |
| 1.4249 | 80.6706671 | 0.66033748 | 0.00099535 |
| 1.42495 | 80.6829133 | 0.65934213 | 0.00099806 |
| 1.425 | 80.6951755 | 0.65834408 | 0.00100076 |
| 1.42505 | 80.7074537 | 0.65734331 | 0.00100348 |
| 1.4251 | 80.719748 | 0.65633983 | 0.0010062 |
| 1.42515 | 80.7320584 | 0.65533363 | 0.00100893 |
| 1.4252 | 80.7443851 | 0.65432469 | 0.00101167 |
| 1.42525 | 80.7567281 | 0.65331302 | 0.00101442 |
| 1.4253 | 80.7690875 | 0.6522986 | 0.00101717 |
| 1.42535 | 80.7814633 | 0.65128143 | 0.00101993 |
| 1.4254 | 80.7938555 | 0.65026151 | 0.00102269 |
| 1.42545 | 80.8062644 | 0.64923881 | 0.00102547 |
| 1.4255 | 80.8186898 | 0.64821334 | 0.00102825 |
| 1.42555 | 80.831132 | 0.6471851 | 0.00103104 |
| 1.4256 | 80.8435909 | 0.64615406 | 0.00103383 |
| 1.42565 | 80.8560666 | 0.64512023 | 0.00103663 |
| 1.4257 | 80.8685593 | 0.64408359 | 0.00103944 |
| 1.42575 | 80.8810689 | 0.64304415 | 0.00104226 |
| 1.4258 | 80.8935956 | 0.64200189 | 0.00104508 |
| 1.42585 | 80.9061393 | 0.64095681 | 0.00104792 |
| 1.4259 | 80.9187003 | 0.63990889 | 0.00105076 |
| 1.42595 | 80.9312785 | 0.63885813 | 0.0010536 |
| 1.426 | 80.943874 | 0.63780453 | 0.00105646 |
| 1.42605 | 80.9564869 | 0.63674808 | 0.00105932 |
| 1.4261 | 80.9691173 | 0.63568876 | 0.00106219 |
| 1.42615 | 80.9817652 | 0.63462657 | 0.00106506 |
| 1.4262 | 80.9944307 | 0.63356151 | 0.00106795 |
| 1.42625 | 81.007114 | 0.63249356 | 0.00107084 |
| 1.4263 | 81.019815 | 0.63142273 | 0.00107374 |
| 1.42635 | 81.0325338 | 0.63034899 | 0.00107664 |
| 1.4264 | 81.0452706 | 0.62927235 | 0.00107956 |
| 1.42645 | 81.0580253 | 0.62819279 | 0.00108248 |
| 1.4265 | 81.0707981 | 0.62711032 | 0.00108541 |
| 1.42655 | 81.0835891 | 0.62602491 | 0.00108834 |
| 1.4266 | 81.0963983 | 0.62493657 | 0.00109129 |
| 1.42665 | 81.1092258 | 0.62384528 | 0.00109424 |
| 1.4267 | 81.1220717 | 0.62275104 | 0.0010972 |
| 1.42675 | 81.134936 | 0.62165385 | 0.00110016 |
| 1.4268 | 81.1478189 | 0.62055368 | 0.00110314 |
| 1.42685 | 81.1607205 | 0.61945054 | 0.00110612 |
| 1.4269 | 81.1736407 | 0.61834442 | 0.00110911 |
| 1.42695 | 81.1865797 | 0.61723531 | 0.00111211 |
| 1.427 | 81.1995376 | 0.6161232 | 0.00111512 |
| 1.42705 | 81.2125145 | 0.61500808 | 0.00111813 |
| 1.4271 | 81.2255104 | 0.61388995 | 0.00112115 |
| 1.42715 | 81.2385254 | 0.6127688 | 0.00112418 |
| 1.4272 | 81.2515596 | 0.61164462 | 0.00112722 |
| 1.42725 | 81.2646131 | 0.6105174 | 0.00113026 |
| 1.4273 | 81.2776861 | 0.60938714 | 0.00113332 |
| 1.42735 | 81.2907784 | 0.60825382 | 0.00113638 |
| 1.4274 | 81.3038904 | 0.60711744 | 0.00113945 |
| 1.42745 | 81.317022 | 0.605978 | 0.00114252 |
| 1.4275 | 81.3301733 | 0.60483547 | 0.00114561 |
| 1.42755 | 81.3433444 | 0.60368987 | 0.0011487 |
| 1.4276 | 81.3565355 | 0.60254117 | 0.0011518 |
| 1.42765 | 81.3697466 | 0.60138937 | 0.00115491 |
| 1.4277 | 81.3829778 | 0.60023446 | 0.00115803 |
| 1.42775 | 81.3962291 | 0.59907643 | 0.00116115 |
| 1.4278 | 81.4095008 | 0.59791528 | 0.00116428 |
| 1.42785 | 81.4227928 | 0.596751 | 0.00116742 |
| 1.4279 | 81.4361054 | 0.59558357 | 0.00117057 |
| 1.42795 | 81.4494384 | 0.594413 | 0.00117373 |
| 1.428 | 81.4627922 | 0.59323927 | 0.0011769 |
| 1.42805 | 81.4761667 | 0.59206237 | 0.00118007 |
| 1.4281 | 81.4895621 | 0.5908823 | 0.00118325 |
| 1.42815 | 81.5029784 | 0.58969905 | 0.00118644 |
| 1.4282 | 81.5164159 | 0.5885126 | 0.00118964 |
| 1.42825 | 81.5298744 | 0.58732296 | 0.00119285 |
| 1.4283 | 81.5433543 | 0.58613011 | 0.00119606 |
| 1.42835 | 81.5568554 | 0.58493405 | 0.00119929 |
| 1.4284 | 81.5703781 | 0.58373476 | 0.00120252 |
| 1.42845 | 81.5839223 | 0.58253225 | 0.00120576 |
| 1.4285 | 81.5974882 | 0.58132649 | 0.00120901 |
| 1.42855 | 81.6110759 | 0.58011748 | 0.00121226 |
| 1.4286 | 81.6246855 | 0.57890522 | 0.00121553 |
| 1.42865 | 81.6383171 | 0.57768969 | 0.0012188 |
| 1.4287 | 81.6519707 | 0.57647089 | 0.00122208 |
| 1.42875 | 81.6656466 | 0.5752488 | 0.00122538 |
| 1.4288 | 81.6793448 | 0.57402343 | 0.00122867 |
| 1.42885 | 81.6930654 | 0.57279475 | 0.00123198 |
| 1.4289 | 81.7068086 | 0.57156277 | 0.0012353 |
| 1.42895 | 81.7205744 | 0.57032747 | 0.00123862 |
| 1.429 | 81.734363 | 0.56908885 | 0.00124196 |
| 1.42905 | 81.7481745 | 0.56784689 | 0.0012453 |
| 1.4291 | 81.7620089 | 0.56660159 | 0.00124865 |
| 1.42915 | 81.7758665 | 0.56535294 | 0.00125201 |
| 1.4292 | 81.7897473 | 0.56410094 | 0.00125538 |
| 1.42925 | 81.8036514 | 0.56284556 | 0.00125875 |
| 1.4293 | 81.817579 | 0.56158681 | 0.00126214 |
| 1.42935 | 81.8315302 | 0.56032467 | 0.00126553 |
| 1.4294 | 81.8455051 | 0.55905914 | 0.00126894 |
| 1.42945 | 81.8595039 | 0.5577902 | 0.00127235 |
| 1.4295 | 81.8735265 | 0.55651786 | 0.00127577 |
| 1.42955 | 81.8875733 | 0.55524209 | 0.0012792 |
| 1.4296 | 81.9016442 | 0.55396289 | 0.00128263 |
| 1.42965 | 81.9157395 | 0.55268026 | 0.00128608 |
| 1.4297 | 81.9298593 | 0.55139418 | 0.00128954 |
| 1.42975 | 81.9440036 | 0.55010464 | 0.001293 |
| 1.4298 | 81.9581726 | 0.54881164 | 0.00129648 |
| 1.42985 | 81.9723665 | 0.54751516 | 0.00129996 |
| 1.4299 | 81.9865854 | 0.5462152 | 0.00130345 |
| 1.42995 | 82.0008293 | 0.54491175 | 0.00130695 |
| 1.43 | 82.0150985 | 0.5436048 | 0.00131046 |
| 1.43005 | 82.0293931 | 0.54229434 | 0.00131398 |
| 1.4301 | 82.0437132 | 0.54098036 | 0.00131751 |
| 1.43015 | 82.058059 | 0.53966286 | 0.00132104 |
| 1.4302 | 82.0724305 | 0.53834181 | 0.00132459 |
| 1.43025 | 82.086828 | 0.53701722 | 0.00132814 |
| 1.4303 | 82.1012516 | 0.53568908 | 0.00133171 |
| 1.43035 | 82.1157014 | 0.53435737 | 0.00133528 |
| 1.4304 | 82.1301775 | 0.53302209 | 0.00133887 |
| 1.43045 | 82.1446802 | 0.53168322 | 0.00134246 |
| 1.4305 | 82.1592095 | 0.53034076 | 0.00134606 |
| 1.43055 | 82.1737656 | 0.5289947 | 0.00134967 |
| 1.4306 | 82.1883486 | 0.52764504 | 0.00135329 |
| 1.43065 | 82.2029588 | 0.52629175 | 0.00135692 |
| 1.4307 | 82.2175962 | 0.52493483 | 0.00136056 |
| 1.43075 | 82.2322611 | 0.52357427 | 0.0013642 |
| 1.4308 | 82.2469535 | 0.52221007 | 0.00136786 |
| 1.43085 | 82.2616736 | 0.52084221 | 0.00137153 |
| 1.4309 | 82.2764217 | 0.51947068 | 0.0013752 |
| 1.43095 | 82.2911977 | 0.51809548 | 0.00137889 |
| 1.431 | 82.306002 | 0.51671659 | 0.00138258 |
| 1.43105 | 82.3208346 | 0.51533401 | 0.00138629 |
| 1.4311 | 82.3356958 | 0.51394772 | 0.00139 |
| 1.43115 | 82.3505857 | 0.51255772 | 0.00139373 |
| 1.4312 | 82.3655044 | 0.51116399 | 0.00139746 |
| 1.43125 | 82.3804522 | 0.50976654 | 0.0014012 |
| 1.4313 | 82.3954291 | 0.50836533 | 0.00140495 |
| 1.43135 | 82.4104355 | 0.50696038 | 0.00140871 |
| 1.4314 | 82.4254714 | 0.50555167 | 0.00141249 |
| 1.43145 | 82.4405371 | 0.50413918 | 0.00141627 |
| 1.4315 | 82.4556326 | 0.50272291 | 0.00142006 |
| 1.43155 | 82.4707583 | 0.50130285 | 0.00142386 |
| 1.4316 | 82.4859142 | 0.499879 | 0.00142767 |
| 1.43165 | 82.5011006 | 0.49845133 | 0.00143149 |
| 1.4317 | 82.5163176 | 0.49701984 | 0.00143532 |
| 1.43175 | 82.5315654 | 0.49558452 | 0.00143916 |
| 1.4318 | 82.5468443 | 0.49414536 | 0.00144301 |
| 1.43185 | 82.5621544 | 0.49270235 | 0.00144687 |
| 1.4319 | 82.5774958 | 0.49125548 | 0.00145074 |
| 1.43195 | 82.5928689 | 0.48980474 | 0.00145462 |
| 1.432 | 82.6082738 | 0.48835012 | 0.00145851 |
| 1.43205 | 82.6237106 | 0.48689161 | 0.00146241 |
| 1.4321 | 82.6391797 | 0.4854292 | 0.00146632 |
| 1.43215 | 82.6546811 | 0.48396289 | 0.00147024 |
| 1.4322 | 82.6702152 | 0.48249265 | 0.00147417 |
| 1.43225 | 82.685782 | 0.48101849 | 0.00147811 |
| 1.4323 | 82.7013819 | 0.47954038 | 0.00148205 |
| 1.43235 | 82.717015 | 0.47805833 | 0.00148601 |
| 1.4324 | 82.7326816 | 0.47657231 | 0.00148998 |
| 1.43245 | 82.7483818 | 0.47508233 | 0.00149396 |
| 1.4325 | 82.7641159 | 0.47358836 | 0.00149795 |
| 1.43255 | 82.7798841 | 0.47209041 | 0.00150196 |
| 1.4326 | 82.7956867 | 0.47058845 | 0.00150597 |
| 1.43265 | 82.8115238 | 0.46908249 | 0.00150999 |
| 1.4327 | 82.8273956 | 0.4675725 | 0.00151402 |
| 1.43275 | 82.8433025 | 0.46605848 | 0.00151806 |
| 1.4328 | 82.8592447 | 0.46454042 | 0.00152211 |
| 1.43285 | 82.8752223 | 0.46301831 | 0.00152618 |
| 1.4329 | 82.8912357 | 0.46149213 | 0.00153025 |
| 1.43295 | 82.907285 | 0.45996188 | 0.00153433 |
| 1.433 | 82.9233705 | 0.45842755 | 0.00153842 |
| 1.43305 | 82.9394925 | 0.45688913 | 0.00154253 |
| 1.4331 | 82.9556512 | 0.4553466 | 0.00154664 |
| 1.43315 | 82.9718469 | 0.45379996 | 0.00155077 |
| 1.4332 | 82.9880798 | 0.45224919 | 0.0015549 |
| 1.43325 | 83.0043502 | 0.45069428 | 0.00155905 |
| 1.4333 | 83.0206583 | 0.44913523 | 0.00156321 |
| 1.43335 | 83.0370044 | 0.44757202 | 0.00156738 |
| 1.4334 | 83.0533888 | 0.44600465 | 0.00157155 |
| 1.43345 | 83.0698117 | 0.44443309 | 0.00157574 |
| 1.4335 | 83.0862735 | 0.44285735 | 0.00157994 |
| 1.43355 | 83.1027743 | 0.4412774 | 0.00158415 |
| 1.4336 | 83.1193145 | 0.43969325 | 0.00158837 |
| 1.43365 | 83.1358944 | 0.43810488 | 0.00159261 |
| 1.4337 | 83.1525143 | 0.43651227 | 0.00159685 |
| 1.43375 | 83.1691744 | 0.43491542 | 0.0016011 |
| 1.4338 | 83.185875 | 0.43331432 | 0.00160537 |
| 1.43385 | 83.2026165 | 0.43170895 | 0.00160964 |
| 1.4339 | 83.2193991 | 0.43009931 | 0.00161393 |
| 1.43395 | 83.2362231 | 0.42848538 | 0.00161823 |
| 1.434 | 83.253089 | 0.42686715 | 0.00162254 |
| 1.43405 | 83.2699968 | 0.42524461 | 0.00162685 |
| 1.4341 | 83.2869471 | 0.42361776 | 0.00163118 |
| 1.43415 | 83.3039401 | 0.42198657 | 0.00163553 |
| 1.4342 | 83.3209761 | 0.42035105 | 0.00163988 |
| 1.43425 | 83.3380555 | 0.41871117 | 0.00164424 |
| 1.4343 | 83.3551786 | 0.41706693 | 0.00164862 |
| 1.43435 | 83.3723457 | 0.41541831 | 0.001653 |
| 1.4344 | 83.3895572 | 0.41376531 | 0.0016574 |
| 1.43445 | 83.4068135 | 0.41210791 | 0.00166181 |
| 1.4345 | 83.4241148 | 0.4104461 | 0.00166623 |
| 1.43455 | 83.4414615 | 0.40877987 | 0.00167066 |
| 1.4346 | 83.4588541 | 0.40710921 | 0.0016751 |
| 1.43465 | 83.4762928 | 0.40543411 | 0.00167955 |
| 1.4347 | 83.4937781 | 0.40375455 | 0.00168402 |
| 1.43475 | 83.5113103 | 0.40207053 | 0.0016885 |
| 1.4348 | 83.5288898 | 0.40038204 | 0.00169298 |
| 1.43485 | 83.5465169 | 0.39868906 | 0.00169748 |
| 1.4349 | 83.5641922 | 0.39699157 | 0.00170199 |
| 1.43495 | 83.5819159 | 0.39528958 | 0.00170652 |
| 1.435 | 83.5996884 | 0.39358306 | 0.00171105 |
| 1.43505 | 83.6175103 | 0.39187202 | 0.00171559 |
| 1.4351 | 83.6353819 | 0.39015642 | 0.00172015 |
| 1.43515 | 83.6533035 | 0.38843627 | 0.00172472 |
| 1.4352 | 83.6712758 | 0.38671155 | 0.0017293 |
| 1.43525 | 83.689299 | 0.38498225 | 0.00173389 |
| 1.4353 | 83.7073736 | 0.38324836 | 0.00173849 |
| 1.43535 | 83.7255 | 0.38150987 | 0.00174311 |
| 1.4354 | 83.7436788 | 0.37976676 | 0.00174774 |
| 1.43545 | 83.7619103 | 0.37801902 | 0.00175237 |
| 1.4355 | 83.7801951 | 0.37626665 | 0.00175703 |
| 1.43555 | 83.7985335 | 0.37450962 | 0.00176169 |
| 1.4356 | 83.8169262 | 0.37274793 | 0.00176636 |
| 1.43565 | 83.8353735 | 0.37098157 | 0.00177105 |
| 1.4357 | 83.8538759 | 0.36921052 | 0.00177575 |
| 1.43575 | 83.872434 | 0.36743478 | 0.00178046 |
| 1.4358 | 83.8910483 | 0.36565432 | 0.00178518 |
| 1.43585 | 83.9097192 | 0.36386915 | 0.00178991 |
| 1.4359 | 83.9284474 | 0.36207923 | 0.00179466 |
| 1.43595 | 83.9472332 | 0.36028458 | 0.00179942 |
| 1.436 | 83.9660774 | 0.35848516 | 0.00180419 |
| 1.43605 | 83.9849803 | 0.35668098 | 0.00180897 |
| 1.4361 | 84.0039427 | 0.35487201 | 0.00181376 |
| 1.43615 | 84.0229649 | 0.35305824 | 0.00181857 |
| 1.4362 | 84.0420477 | 0.35123968 | 0.00182339 |
| 1.43625 | 84.0611916 | 0.34941629 | 0.00182822 |
| 1.4363 | 84.0803971 | 0.34758807 | 0.00183306 |
| 1.43635 | 84.099665 | 0.34575501 | 0.00183792 |
| 1.4364 | 84.1189957 | 0.34391709 | 0.00184279 |
| 1.43645 | 84.13839 | 0.3420743 | 0.00184767 |
| 1.4365 | 84.1578485 | 0.34022663 | 0.00185256 |
| 1.43655 | 84.1773717 | 0.33837408 | 0.00185746 |
| 1.4366 | 84.1969604 | 0.33651661 | 0.00186238 |
| 1.43665 | 84.2166152 | 0.33465423 | 0.00186731 |
| 1.4367 | 84.2363368 | 0.33278692 | 0.00187226 |
| 1.43675 | 84.2561259 | 0.33091466 | 0.00187721 |
| 1.4368 | 84.2759832 | 0.32903745 | 0.00188218 |
| 1.43685 | 84.2959093 | 0.32715527 | 0.00188716 |
| 1.4369 | 84.3159051 | 0.32526811 | 0.00189215 |
| 1.43695 | 84.3359713 | 0.32337596 | 0.00189716 |
| 1.437 | 84.3561086 | 0.3214788 | 0.00190218 |
| 1.43705 | 84.3763177 | 0.31957663 | 0.00190721 |
| 1.4371 | 84.3965995 | 0.31766942 | 0.00191225 |
| 1.43715 | 84.4169547 | 0.31575717 | 0.00191731 |
| 1.4372 | 84.4373842 | 0.31383986 | 0.00192238 |
| 1.43725 | 84.4578887 | 0.31191748 | 0.00192746 |
| 1.4373 | 84.4784692 | 0.30999002 | 0.00193256 |
| 1.43735 | 84.4991264 | 0.30805746 | 0.00193766 |
| 1.4374 | 84.5198612 | 0.3061198 | 0.00194279 |
| 1.43745 | 84.5406746 | 0.30417701 | 0.00194792 |
| 1.4375 | 84.5615673 | 0.3022291 | 0.00195307 |
| 1.43755 | 84.5825404 | 0.30027603 | 0.00195823 |
| 1.4376 | 84.6035948 | 0.2983178 | 0.0019634 |
| 1.43765 | 84.6247313 | 0.2963544 | 0.00196859 |
| 1.4377 | 84.6459511 | 0.29438581 | 0.00197379 |
| 1.43775 | 84.6672551 | 0.29241203 | 0.001979 |
| 1.4378 | 84.6886442 | 0.29043303 | 0.00198423 |
| 1.43785 | 84.7101196 | 0.2884488 | 0.00198946 |
| 1.4379 | 84.7316823 | 0.28645934 | 0.00199472 |
| 1.43795 | 84.7533334 | 0.28446462 | 0.00199998 |
| 1.438 | 84.7750739 | 0.28246464 | 0.00200526 |
| 1.43805 | 84.796905 | 0.28045938 | 0.00201055 |
| 1.4381 | 84.8188278 | 0.27844882 | 0.00201586 |
| 1.43815 | 84.8408436 | 0.27643296 | 0.00202118 |
| 1.4382 | 84.8629534 | 0.27441178 | 0.00202651 |
| 1.43825 | 84.8851586 | 0.27238527 | 0.00203186 |
| 1.4383 | 84.9074603 | 0.27035341 | 0.00203722 |
| 1.43835 | 84.9298598 | 0.26831619 | 0.00204259 |
| 1.4384 | 84.9523585 | 0.2662736 | 0.00204798 |
| 1.43845 | 84.9749577 | 0.26422562 | 0.00205338 |
| 1.4385 | 84.9976587 | 0.26217224 | 0.00205879 |
| 1.43855 | 85.0204629 | 0.26011345 | 0.00206422 |
| 1.4386 | 85.0433718 | 0.25804922 | 0.00206966 |
| 1.43865 | 85.0663867 | 0.25597956 | 0.00207512 |
| 1.4387 | 85.0895093 | 0.25390444 | 0.00208059 |
| 1.43875 | 85.112741 | 0.25182385 | 0.00208607 |
| 1.4388 | 85.1360834 | 0.24973778 | 0.00209157 |
| 1.43885 | 85.1595381 | 0.24764621 | 0.00209708 |
| 1.4389 | 85.1831067 | 0.24554913 | 0.0021026 |
| 1.43895 | 85.2067909 | 0.24344653 | 0.00210814 |
| 1.439 | 85.2305925 | 0.24133838 | 0.0021137 |
| 1.43905 | 85.2545132 | 0.23922469 | 0.00211926 |
| 1.4391 | 85.2785547 | 0.23710543 | 0.00212484 |
| 1.43915 | 85.3027191 | 0.23498058 | 0.00213044 |
| 1.4392 | 85.3270081 | 0.23285014 | 0.00213605 |
| 1.43925 | 85.3514237 | 0.2307141 | 0.00214167 |
| 1.4393 | 85.375968 | 0.22857243 | 0.00214731 |
| 1.43935 | 85.400643 | 0.22642512 | 0.00215296 |
| 1.4394 | 85.4254508 | 0.22427216 | 0.00215863 |
| 1.43945 | 85.4503935 | 0.22211353 | 0.00216431 |
| 1.4395 | 85.4754735 | 0.21994923 | 0.00217 |
| 1.43955 | 85.5006929 | 0.21777923 | 0.00217571 |
| 1.4396 | 85.5260543 | 0.21560352 | 0.00218143 |
| 1.43965 | 85.5515599 | 0.21342208 | 0.00218717 |
| 1.4397 | 85.5772123 | 0.21123491 | 0.00219292 |
| 1.43975 | 85.6030141 | 0.20904199 | 0.00219869 |
| 1.4398 | 85.6289679 | 0.2068433 | 0.00220447 |
| 1.43985 | 85.6550764 | 0.20463883 | 0.00221027 |
| 1.4399 | 85.6813424 | 0.20242856 | 0.00221608 |
| 1.43995 | 85.7077688 | 0.20021249 | 0.0022219 |
| 1.44 | 85.7343587 | 0.19799058 | 0.00222774 |
| 1.44005 | 85.761115 | 0.19576284 | 0.0022336 |
| 1.4401 | 85.788041 | 0.19352925 | 0.00223947 |
| 1.44015 | 85.81514 | 0.19128978 | 0.00224535 |
| 1.4402 | 85.8424152 | 0.18904443 | 0.00225125 |
| 1.44025 | 85.8698702 | 0.18679318 | 0.00225716 |
| 1.4403 | 85.8975087 | 0.18453602 | 0.00226309 |
| 1.44035 | 85.9253342 | 0.18227293 | 0.00226904 |
| 1.4404 | 85.9533508 | 0.18000389 | 0.00227499 |
| 1.44045 | 85.9815624 | 0.1777289 | 0.00228097 |
| 1.4405 | 86.0099732 | 0.17544793 | 0.00228696 |
| 1.44055 | 86.0385873 | 0.17316097 | 0.00229296 |
| 1.4406 | 86.0674093 | 0.17086801 | 0.00229898 |
| 1.44065 | 86.0964438 | 0.16856903 | 0.00230501 |
| 1.4407 | 86.1256955 | 0.16626402 | 0.00231106 |
| 1.44075 | 86.1551695 | 0.16395296 | 0.00231713 |
| 1.4408 | 86.1848707 | 0.16163583 | 0.00232321 |
| 1.44085 | 86.2148047 | 0.15931263 | 0.0023293 |
| 1.4409 | 86.244977 | 0.15698332 | 0.00233541 |
| 1.44095 | 86.2753933 | 0.15464791 | 0.00234154 |
| 1.441 | 86.3060596 | 0.15230637 | 0.00234768 |
| 1.44105 | 86.3369824 | 0.1499587 | 0.00235384 |
| 1.4411 | 86.3681681 | 0.14760486 | 0.00236001 |
| 1.44115 | 86.3996235 | 0.14524485 | 0.00236619 |
| 1.4412 | 86.4313558 | 0.14287866 | 0.0023724 |
| 1.44125 | 86.4633724 | 0.14050626 | 0.00237862 |
| 1.4413 | 86.4956812 | 0.13812764 | 0.00238485 |
| 1.44135 | 86.5282902 | 0.13574279 | 0.0023911 |
| 1.4414 | 86.5612081 | 0.13335169 | 0.00239737 |
| 1.44145 | 86.5944438 | 0.13095433 | 0.00240365 |
| 1.4415 | 86.6280066 | 0.12855068 | 0.00240995 |
| 1.44155 | 86.6619065 | 0.12614073 | 0.00241626 |
| 1.4416 | 86.6961538 | 0.12372447 | 0.00242259 |
| 1.44165 | 86.7307594 | 0.12130189 | 0.00242893 |
| 1.4417 | 86.7657349 | 0.11887295 | 0.00243529 |
| 1.44175 | 86.8010924 | 0.11643766 | 0.00244167 |
| 1.4418 | 86.8368446 | 0.11399599 | 0.00244806 |
| 1.44185 | 86.8730052 | 0.11154793 | 0.00245447 |
| 1.4419 | 86.9095884 | 0.10909345 | 0.0024609 |
| 1.44195 | 86.9466095 | 0.10663256 | 0.00246734 |
| 1.442 | 86.9840845 | 0.10416522 | 0.0024738 |
| 1.44205 | 87.0220306 | 0.10169142 | 0.00248027 |
| 1.4421 | 87.0604662 | 0.09921116 | 0.00248676 |
| 1.44215 | 87.0994105 | 0.0967244 | 0.00249326 |
| 1.4422 | 87.1388844 | 0.09423113 | 0.00249979 |
| 1.44225 | 87.1789101 | 0.09173135 | 0.00250633 |
| 1.4423 | 87.2195115 | 0.08922502 | 0.00251288 |
| 1.44235 | 87.2607141 | 0.08671214 | 0.00251945 |
| 1.4424 | 87.3025455 | 0.08419269 | 0.00252604 |
| 1.44245 | 87.3450354 | 0.08166665 | 0.00253264 |
| 1.4425 | 87.388216 | 0.07913401 | 0.00253927 |
| 1.44255 | 87.432122 | 0.07659474 | 0.0025459 |
| 1.4426 | 87.4767915 | 0.07404884 | 0.00255256 |
| 1.44265 | 87.5222656 | 0.07149628 | 0.00255923 |
| 1.4427 | 87.5685894 | 0.06893705 | 0.00256592 |
| 1.44275 | 87.6158127 | 0.06637114 | 0.00257262 |
| 1.4428 | 87.6639898 | 0.06379852 | 0.00257934 |
| 1.44285 | 87.7131811 | 0.06121917 | 0.00258608 |
| 1.4429 | 87.7634534 | 0.05863309 | 0.00259283 |
| 1.44295 | 87.8148815 | 0.05604026 | 0.00259961 |
| 1.443 | 87.8675488 | 0.05344065 | 0.0026064 |
| 1.44305 | 87.9215497 | 0.05083426 | 0.0026132 |
| 1.4431 | 87.9769908 | 0.04822106 | 0.00262002 |
| 1.44315 | 88.0339942 | 0.04560103 | 0.00262686 |
| 1.4432 | 88.0926997 | 0.04297417 | 0.00263372 |
| 1.44325 | 88.1532697 | 0.04034045 | 0.0026406 |
| 1.4433 | 88.2158942 | 0.03769985 | 0.00264749 |
| 1.44335 | 88.2807976 | 0.03505236 | 0.0026544 |
| 1.4434 | 88.3482485 | 0.03239797 | 0.00266132 |
| 1.44345 | 88.4185729 | 0.02973665 | 0.00266827 |
| 1.4435 | 88.4921728 | 0.02706838 | 0.00267523 |
| 1.44355 | 88.5695537 | 0.02439315 | 0.0026822 |
| 1.4436 | 88.6513662 | 0.02171095 | 0.0026892 |
| 1.44365 | 88.7384722 | 0.01902175 | 0.00269621 |
| 1.4437 | 88.8320556 | 0.01632554 | 0.00270324 |
| 1.44375 | 88.9338206 | 0.01362229 | 0.00271029 |
| 1.4438 | 89.0463829 | 0.010912 | 0.00271736 |
| 1.44385 | 89.1741457 | 0.00819464 | 0.00272444 |
| 1.4439 | 89.3256948 | 0.0054702 | 0.00273154 |
| 1.44395 | 89.5231956 | 0.00273866 | 0.00273866 |

**Table 5.** Simulation results for 1-cm sensor over three sensing zones.

| RI @1550 nm | Normalized power (a.u.) |
| --- | --- |
| 1.3165 | 0.987936612 |
| 1.319 | 0.987676158 |
| 1.3215 | 0.987400382 |
| 1.324 | 0.987107939 |
| 1.3265 | 0.986797320 |
| 1.329 | 0.986466826 |
| 1.3315 | 0.986114528 |
| 1.334 | 0.985738233 |
| 1.3365 | 0.985335430 |
| 1.339 | 0.984903220 |
| 1.3415 | 0.984438239 |
| 1.344 | 0.983936544 |
| 1.3465 | 0.983393463 |
| 1.349 | 0.982803393 |
| 1.3515 | 0.982159503 |
| 1.354 | 0.981453294 |
| 1.3565 | 0.980673905 |
| 1.359 | 0.979806915 |
| 1.3615 | 0.978832085 |
| 1.364 | 0.977718338 |
| 1.3665 | 0.976409449 |
| 1.369 | 0.974753411 |
| 1.37 | 0.973871488 |
| 1.37005 | 0.973807077 |
| 1.3701 | 0.973742508 |
| 1.37015 | 0.973677781 |
| 1.3702 | 0.973612895 |
| 1.37025 | 0.973547851 |
| 1.3703 | 0.973482648 |
| 1.37035 | 0.973417285 |
| 1.3704 | 0.973351762 |
| 1.37045 | 0.973286079 |
| 1.3705 | 0.973220235 |
| 1.37055 | 0.973154229 |
| 1.3706 | 0.973088062 |
| 1.37065 | 0.973021733 |
| 1.3707 | 0.972955242 |
| 1.37075 | 0.972888587 |
| 1.3708 | 0.972821769 |
| 1.37085 | 0.972754788 |
| 1.3709 | 0.972687642 |
| 1.37095 | 0.972620331 |
| 1.371 | 0.972552856 |
| 1.37105 | 0.972485215 |
| 1.3711 | 0.972417407 |
| 1.37115 | 0.972349434 |
| 1.3712 | 0.972281294 |
| 1.37125 | 0.972212986 |
| 1.3713 | 0.972144511 |
| 1.37135 | 0.972075868 |
| 1.3714 | 0.972007056 |
| 1.37145 | 0.971938075 |
| 1.3715 | 0.971868925 |
| 1.37155 | 0.971799605 |
| 1.3716 | 0.971730115 |
| 1.37165 | 0.971660453 |
| 1.3717 | 0.971590621 |
| 1.37175 | 0.971520617 |
| 1.3718 | 0.971450441 |
| 1.37185 | 0.971380092 |
| 1.3719 | 0.971309570 |
| 1.37195 | 0.971238875 |
| 1.372 | 0.971168006 |
| 1.37205 | 0.971096962 |
| 1.3721 | 0.971025744 |
| 1.37215 | 0.970954350 |
| 1.3722 | 0.970882781 |
| 1.37225 | 0.970811035 |
| 1.3723 | 0.970739113 |
| 1.37235 | 0.970667014 |
| 1.3724 | 0.970594737 |
| 1.37245 | 0.970522282 |
| 1.3725 | 0.970449648 |
| 1.37255 | 0.970376836 |
| 1.3726 | 0.970303844 |
| 1.37265 | 0.970230672 |
| 1.3727 | 0.970157319 |
| 1.37275 | 0.970083786 |
| 1.3728 | 0.970010072 |
| 1.37285 | 0.969936176 |
| 1.3729 | 0.969862097 |
| 1.37295 | 0.969787836 |
| 1.373 | 0.969713391 |
| 1.37305 | 0.969638763 |
| 1.3731 | 0.969563951 |
| 1.37315 | 0.969488954 |
| 1.3732 | 0.969413771 |
| 1.37325 | 0.969338404 |
| 1.3733 | 0.969262850 |
| 1.37335 | 0.969187109 |
| 1.3734 | 0.969111181 |
| 1.37345 | 0.969035066 |
| 1.3735 | 0.968958763 |
| 1.37355 | 0.968882271 |
| 1.3736 | 0.968805590 |
| 1.37365 | 0.968728719 |
| 1.3737 | 0.968651659 |
| 1.37375 | 0.968574407 |
| 1.3738 | 0.968496965 |
| 1.37385 | 0.968419332 |
| 1.3739 | 0.968341506 |
| 1.37395 | 0.968263488 |
| 1.374 | 0.968185276 |
| 1.37405 | 0.968106871 |
| 1.3741 | 0.968028272 |
| 1.37415 | 0.967949479 |
| 1.3742 | 0.967870490 |
| 1.37425 | 0.967791306 |
| 1.3743 | 0.967711926 |
| 1.37435 | 0.967632349 |
| 1.3744 | 0.967552575 |
| 1.37445 | 0.967472604 |
| 1.3745 | 0.967392434 |
| 1.37455 | 0.967312066 |
| 1.3746 | 0.967231498 |
| 1.37465 | 0.967150731 |
| 1.3747 | 0.967069764 |
| 1.37475 | 0.966988595 |
| 1.3748 | 0.966907226 |
| 1.37485 | 0.966825655 |
| 1.3749 | 0.966743881 |
| 1.37495 | 0.966661905 |
| 1.375 | 0.966579725 |
| 1.37505 | 0.966497341 |
| 1.3751 | 0.966414753 |
| 1.37515 | 0.966331960 |
| 1.3752 | 0.966248962 |
| 1.37525 | 0.966165757 |
| 1.3753 | 0.966082346 |
| 1.37535 | 0.965998727 |
| 1.3754 | 0.965914901 |
| 1.37545 | 0.965830867 |
| 1.3755 | 0.965746624 |
| 1.37555 | 0.965662172 |
| 1.3756 | 0.965577510 |
| 1.37565 | 0.965492637 |
| 1.3757 | 0.965407554 |
| 1.37575 | 0.965322259 |
| 1.3758 | 0.965236752 |
| 1.37585 | 0.965151033 |
| 1.3759 | 0.965065100 |
| 1.37595 | 0.964978954 |
| 1.376 | 0.964892593 |
| 1.37605 | 0.964806018 |
| 1.3761 | 0.964719227 |
| 1.37615 | 0.964632220 |
| 1.3762 | 0.964544997 |
| 1.37625 | 0.964457557 |
| 1.3763 | 0.964369899 |
| 1.37635 | 0.964282023 |
| 1.3764 | 0.964193928 |
| 1.37645 | 0.964105614 |
| 1.3765 | 0.964017080 |
| 1.37655 | 0.963928325 |
| 1.3766 | 0.963839349 |
| 1.37665 | 0.963750152 |
| 1.3767 | 0.963660733 |
| 1.37675 | 0.963571090 |
| 1.3768 | 0.963481225 |
| 1.37685 | 0.963391135 |
| 1.3769 | 0.963300821 |
| 1.37695 | 0.963210282 |
| 1.377 | 0.963119517 |
| 1.37705 | 0.963028525 |
| 1.3771 | 0.962937307 |
| 1.37715 | 0.962845861 |
| 1.3772 | 0.962754188 |
| 1.37725 | 0.962662285 |
| 1.3773 | 0.962570154 |
| 1.37735 | 0.962477792 |
| 1.3774 | 0.962385200 |
| 1.37745 | 0.962292377 |
| 1.3775 | 0.962199322 |
| 1.37755 | 0.962106035 |
| 1.3776 | 0.962012516 |
| 1.37765 | 0.961918762 |
| 1.3777 | 0.961824775 |
| 1.37775 | 0.961730553 |
| 1.3778 | 0.961636095 |
| 1.37785 | 0.961541402 |
| 1.3779 | 0.961446472 |
| 1.37795 | 0.961351305 |
| 1.378 | 0.961255900 |
| 1.37805 | 0.961160257 |
| 1.3781 | 0.961064375 |
| 1.37815 | 0.960968253 |
| 1.3782 | 0.960871891 |
| 1.37825 | 0.960775288 |
| 1.3783 | 0.960678443 |
| 1.37835 | 0.960581356 |
| 1.3784 | 0.960484027 |
| 1.37845 | 0.960386454 |
| 1.3785 | 0.960288637 |
| 1.37855 | 0.960190576 |
| 1.3786 | 0.960092269 |
| 1.37865 | 0.959993716 |
| 1.3787 | 0.959894917 |
| 1.37875 | 0.959795870 |
| 1.3788 | 0.959696575 |
| 1.37885 | 0.959597032 |
| 1.3789 | 0.959497240 |
| 1.37895 | 0.959397198 |
| 1.379 | 0.959296906 |
| 1.37905 | 0.959196362 |
| 1.3791 | 0.959095567 |
| 1.37915 | 0.958994519 |
| 1.3792 | 0.958893218 |
| 1.37925 | 0.958791663 |
| 1.3793 | 0.958689854 |
| 1.37935 | 0.958587790 |
| 1.3794 | 0.958485470 |
| 1.37945 | 0.958382894 |
| 1.3795 | 0.958280061 |
| 1.37955 | 0.958176970 |
| 1.3796 | 0.958073620 |
| 1.37965 | 0.957970012 |
| 1.3797 | 0.957866143 |
| 1.37975 | 0.957762015 |
| 1.3798 | 0.957657625 |
| 1.37985 | 0.957552973 |
| 1.3799 | 0.957448059 |
| 1.37995 | 0.957342882 |
| 1.38 | 0.957237441 |
| 1.38005 | 0.957131735 |
| 1.3801 | 0.957025764 |
| 1.38015 | 0.956919528 |
| 1.3802 | 0.956813024 |
| 1.38025 | 0.956706254 |
| 1.3803 | 0.956599215 |
| 1.38035 | 0.956491908 |
| 1.3804 | 0.956384331 |
| 1.38045 | 0.956276485 |
| 1.3805 | 0.956168367 |
| 1.38055 | 0.956059978 |
| 1.3806 | 0.955951317 |
| 1.38065 | 0.955842383 |
| 1.3807 | 0.955733175 |
| 1.38075 | 0.955623694 |
| 1.3808 | 0.955513937 |
| 1.38085 | 0.955403904 |
| 1.3809 | 0.955293595 |
| 1.38095 | 0.955183008 |
| 1.381 | 0.955072144 |
| 1.38105 | 0.954961001 |
| 1.3811 | 0.954849579 |
| 1.38115 | 0.954737877 |
| 1.3812 | 0.954625894 |
| 1.38125 | 0.954513629 |
| 1.3813 | 0.954401082 |
| 1.38135 | 0.954288252 |
| 1.3814 | 0.954175139 |
| 1.38145 | 0.954061740 |
| 1.3815 | 0.953948057 |
| 1.38155 | 0.953834088 |
| 1.3816 | 0.953719832 |
| 1.38165 | 0.953605289 |
| 1.3817 | 0.953490457 |
| 1.38175 | 0.953375337 |
| 1.3818 | 0.953259926 |
| 1.38185 | 0.953144226 |
| 1.3819 | 0.953028234 |
| 1.38195 | 0.952911950 |
| 1.382 | 0.952795374 |
| 1.38205 | 0.952678504 |
| 1.3821 | 0.952561340 |
| 1.38215 | 0.952443881 |
| 1.3822 | 0.952326126 |
| 1.38225 | 0.952208074 |
| 1.3823 | 0.952089726 |
| 1.38235 | 0.951971079 |
| 1.3824 | 0.951852133 |
| 1.38245 | 0.951732888 |
| 1.3825 | 0.951613343 |
| 1.38255 | 0.951493496 |
| 1.3826 | 0.951373347 |
| 1.38265 | 0.951252896 |
| 1.3827 | 0.951132141 |
| 1.38275 | 0.951011081 |
| 1.3828 | 0.950889717 |
| 1.38285 | 0.950768047 |
| 1.3829 | 0.950646070 |
| 1.38295 | 0.950523785 |
| 1.383 | 0.950401192 |
| 1.38305 | 0.950278290 |
| 1.3831 | 0.950155079 |
| 1.38315 | 0.950031556 |
| 1.3832 | 0.949907722 |
| 1.38325 | 0.949783576 |
| 1.3833 | 0.949659117 |
| 1.38335 | 0.949534344 |
| 1.3834 | 0.949409256 |
| 1.38345 | 0.949283852 |
| 1.3835 | 0.949158132 |
| 1.38355 | 0.949032095 |
| 1.3836 | 0.948905740 |
| 1.38365 | 0.948779066 |
| 1.3837 | 0.948652073 |
| 1.38375 | 0.948524759 |
| 1.3838 | 0.948397123 |
| 1.38385 | 0.948269166 |
| 1.3839 | 0.948140885 |
| 1.38395 | 0.948012281 |
| 1.384 | 0.947883352 |
| 1.38405 | 0.947754097 |
| 1.3841 | 0.947624516 |
| 1.38415 | 0.947494608 |
| 1.3842 | 0.947364372 |
| 1.38425 | 0.947233807 |
| 1.3843 | 0.947102912 |
| 1.38435 | 0.946971687 |
| 1.3844 | 0.946840130 |
| 1.38445 | 0.946708241 |
| 1.3845 | 0.946576018 |
| 1.38455 | 0.946443462 |
| 1.3846 | 0.946310570 |
| 1.38465 | 0.946177343 |
| 1.3847 | 0.946043780 |
| 1.38475 | 0.945909878 |
| 1.3848 | 0.945775639 |
| 1.38485 | 0.945641060 |
| 1.3849 | 0.945506141 |
| 1.38495 | 0.945370880 |
| 1.385 | 0.945235278 |
| 1.38505 | 0.945099334 |
| 1.3851 | 0.944963045 |
| 1.38515 | 0.944826412 |
| 1.3852 | 0.944689434 |
| 1.38525 | 0.944552109 |
| 1.3853 | 0.944414437 |
| 1.38535 | 0.944276416 |
| 1.3854 | 0.944138047 |
| 1.38545 | 0.943999328 |
| 1.3855 | 0.943860257 |
| 1.38555 | 0.943720835 |
| 1.3856 | 0.943581061 |
| 1.38565 | 0.943440933 |
| 1.3857 | 0.943300450 |
| 1.38575 | 0.943159612 |
| 1.3858 | 0.943018417 |
| 1.38585 | 0.942876865 |
| 1.3859 | 0.942734956 |
| 1.38595 | 0.942592686 |
| 1.386 | 0.942450057 |
| 1.38605 | 0.942307067 |
| 1.3861 | 0.942163715 |
| 1.38615 | 0.942020000 |
| 1.3862 | 0.941875922 |
| 1.38625 | 0.941731478 |
| 1.3863 | 0.941586669 |
| 1.38635 | 0.941441494 |
| 1.3864 | 0.941295951 |
| 1.38645 | 0.941150039 |
| 1.3865 | 0.941003758 |
| 1.38655 | 0.940857106 |
| 1.3866 | 0.940710083 |
| 1.38665 | 0.940562688 |
| 1.3867 | 0.940414919 |
| 1.38675 | 0.940266776 |
| 1.3868 | 0.940118258 |
| 1.38685 | 0.939969364 |
| 1.3869 | 0.939820093 |
| 1.38695 | 0.939670443 |
| 1.387 | 0.939520414 |
| 1.38705 | 0.939370006 |
| 1.3871 | 0.939219216 |
| 1.38715 | 0.939068044 |
| 1.3872 | 0.938916489 |
| 1.38725 | 0.938764550 |
| 1.3873 | 0.938612226 |
| 1.38735 | 0.938459516 |
| 1.3874 | 0.938306419 |
| 1.38745 | 0.938152933 |
| 1.3875 | 0.937999059 |
| 1.38755 | 0.937844795 |
| 1.3876 | 0.937690140 |
| 1.38765 | 0.937535093 |
| 1.3877 | 0.937379652 |
| 1.38775 | 0.937223818 |
| 1.3878 | 0.937067589 |
| 1.38785 | 0.936910963 |
| 1.3879 | 0.936753940 |
| 1.38795 | 0.936596519 |
| 1.388 | 0.936438699 |
| 1.38805 | 0.936280479 |
| 1.3881 | 0.936121858 |
| 1.38815 | 0.935962834 |
| 1.3882 | 0.935803407 |
| 1.38825 | 0.935643576 |
| 1.3883 | 0.935483339 |
| 1.38835 | 0.935322696 |
| 1.3884 | 0.935161645 |
| 1.38845 | 0.935000186 |
| 1.3885 | 0.934838317 |
| 1.38855 | 0.934676037 |
| 1.3886 | 0.934513346 |
| 1.38865 | 0.934350242 |
| 1.3887 | 0.934186725 |
| 1.38875 | 0.934022792 |
| 1.3888 | 0.933858443 |
| 1.38885 | 0.933693678 |
| 1.3889 | 0.933528494 |
| 1.38895 | 0.933362891 |
| 1.389 | 0.933196868 |
| 1.38905 | 0.933030423 |
| 1.3891 | 0.932863556 |
| 1.38915 | 0.932696266 |
| 1.3892 | 0.932528550 |
| 1.38925 | 0.932360410 |
| 1.3893 | 0.932191842 |
| 1.38935 | 0.932022846 |
| 1.3894 | 0.931853422 |
| 1.38945 | 0.931683567 |
| 1.3895 | 0.931513281 |
| 1.38955 | 0.931342563 |
| 1.3896 | 0.931171411 |
| 1.38965 | 0.930999825 |
| 1.3897 | 0.930827803 |
| 1.38975 | 0.930655345 |
| 1.3898 | 0.930482448 |
| 1.38985 | 0.930309113 |
| 1.3899 | 0.930135337 |
| 1.38995 | 0.929961120 |
| 1.39 | 0.929786460 |
| 1.39005 | 0.929611357 |
| 1.3901 | 0.929435809 |
| 1.39015 | 0.929259816 |
| 1.3902 | 0.929083375 |
| 1.39025 | 0.928906486 |
| 1.3903 | 0.928729148 |
| 1.39035 | 0.928551359 |
| 1.3904 | 0.928373119 |
| 1.39045 | 0.928194426 |
| 1.3905 | 0.928015279 |
| 1.39055 | 0.927835677 |
| 1.3906 | 0.927655619 |
| 1.39065 | 0.927475103 |
| 1.3907 | 0.927294129 |
| 1.39075 | 0.927112694 |
| 1.3908 | 0.926930799 |
| 1.39085 | 0.926748442 |
| 1.3909 | 0.926565621 |
| 1.39095 | 0.926382336 |
| 1.391 | 0.926198585 |
| 1.39105 | 0.926014367 |
| 1.3911 | 0.925829681 |
| 1.39115 | 0.925644525 |
| 1.3912 | 0.925458899 |
| 1.39125 | 0.925272801 |
| 1.3913 | 0.925086230 |
| 1.39135 | 0.924899186 |
| 1.3914 | 0.924711665 |
| 1.39145 | 0.924523668 |
| 1.3915 | 0.924335194 |
| 1.39155 | 0.924146240 |
| 1.3916 | 0.923956806 |
| 1.39165 | 0.923766890 |
| 1.3917 | 0.923576491 |
| 1.39175 | 0.923385609 |
| 1.3918 | 0.923194241 |
| 1.39185 | 0.923002387 |
| 1.3919 | 0.922810045 |
| 1.39195 | 0.922617214 |
| 1.392 | 0.922423893 |
| 1.39205 | 0.922230081 |
| 1.3921 | 0.922035775 |
| 1.39215 | 0.921840976 |
| 1.3922 | 0.921645681 |
| 1.39225 | 0.921449890 |
| 1.3923 | 0.921253601 |
| 1.39235 | 0.921056813 |
| 1.3924 | 0.920859524 |
| 1.39245 | 0.920661734 |
| 1.3925 | 0.920463441 |
| 1.39255 | 0.920264644 |
| 1.3926 | 0.920065341 |
| 1.39265 | 0.919865531 |
| 1.3927 | 0.919665213 |
| 1.39275 | 0.919464386 |
| 1.3928 | 0.919263048 |
| 1.39285 | 0.919061198 |
| 1.3929 | 0.918858834 |
| 1.39295 | 0.918655956 |
| 1.393 | 0.918452562 |
| 1.39305 | 0.918248651 |
| 1.3931 | 0.918044221 |
| 1.39315 | 0.917839271 |
| 1.3932 | 0.917633800 |
| 1.39325 | 0.917427806 |
| 1.3933 | 0.917221288 |
| 1.39335 | 0.917014245 |
| 1.3934 | 0.916806675 |
| 1.39345 | 0.916598578 |
| 1.3935 | 0.916389951 |
| 1.39355 | 0.916180793 |
| 1.3936 | 0.915971103 |
| 1.39365 | 0.915760880 |
| 1.3937 | 0.915550122 |
| 1.39375 | 0.915338828 |
| 1.3938 | 0.915126996 |
| 1.39385 | 0.914914626 |
| 1.3939 | 0.914701715 |
| 1.39395 | 0.914488263 |
| 1.394 | 0.914274268 |
| 1.39405 | 0.914059728 |
| 1.3941 | 0.913844642 |
| 1.39415 | 0.913629009 |
| 1.3942 | 0.913412828 |
| 1.39425 | 0.913196097 |
| 1.3943 | 0.912978814 |
| 1.39435 | 0.912760978 |
| 1.3944 | 0.912542589 |
| 1.39445 | 0.912323643 |
| 1.3945 | 0.912104141 |
| 1.39455 | 0.911884080 |
| 1.3946 | 0.911663459 |
| 1.39465 | 0.911442277 |
| 1.3947 | 0.911220532 |
| 1.39475 | 0.910998223 |
| 1.3948 | 0.910775348 |
| 1.39485 | 0.910551906 |
| 1.3949 | 0.910327896 |
| 1.39495 | 0.910103315 |
| 1.395 | 0.909878164 |
| 1.39505 | 0.909652439 |
| 1.3951 | 0.909426140 |
| 1.39515 | 0.909199265 |
| 1.3952 | 0.908971813 |
| 1.39525 | 0.908743782 |
| 1.3953 | 0.908515171 |
| 1.39535 | 0.908285978 |
| 1.3954 | 0.908056202 |
| 1.39545 | 0.907825841 |
| 1.3955 | 0.907594894 |
| 1.39555 | 0.907363360 |
| 1.3956 | 0.907131236 |
| 1.39565 | 0.906898522 |
| 1.3957 | 0.906665215 |
| 1.39575 | 0.906431315 |
| 1.3958 | 0.906196820 |
| 1.39585 | 0.905961728 |
| 1.3959 | 0.905726037 |
| 1.39595 | 0.905489747 |
| 1.396 | 0.905252856 |
| 1.39605 | 0.905015362 |
| 1.3961 | 0.904777263 |
| 1.39615 | 0.904538559 |
| 1.3962 | 0.904299247 |
| 1.39625 | 0.904059326 |
| 1.3963 | 0.903818795 |
| 1.39635 | 0.903577652 |
| 1.3964 | 0.903335895 |
| 1.39645 | 0.903093523 |
| 1.3965 | 0.902850534 |
| 1.39655 | 0.902606927 |
| 1.3966 | 0.902362700 |
| 1.39665 | 0.902117851 |
| 1.3967 | 0.901872380 |
| 1.39675 | 0.901626284 |
| 1.3968 | 0.901379561 |
| 1.39685 | 0.901132211 |
| 1.3969 | 0.900884231 |
| 1.39695 | 0.900635621 |
| 1.397 | 0.900386378 |
| 1.39705 | 0.900136500 |
| 1.3971 | 0.899885987 |
| 1.39715 | 0.899634836 |
| 1.3972 | 0.899383046 |
| 1.39725 | 0.899130616 |
| 1.3973 | 0.898877543 |
| 1.39735 | 0.898623826 |
| 1.3974 | 0.898369464 |
| 1.39745 | 0.898114455 |
| 1.3975 | 0.897858796 |
| 1.39755 | 0.897602488 |
| 1.3976 | 0.897345527 |
| 1.39765 | 0.897087912 |
| 1.3977 | 0.896829642 |
| 1.39775 | 0.896570714 |
| 1.3978 | 0.896311128 |
| 1.39785 | 0.896050881 |
| 1.3979 | 0.895789972 |
| 1.39795 | 0.895528400 |
| 1.398 | 0.895266162 |
| 1.39805 | 0.895003256 |
| 1.3981 | 0.894739682 |
| 1.39815 | 0.894475437 |
| 1.3982 | 0.894210520 |
| 1.39825 | 0.893944929 |
| 1.3983 | 0.893678663 |
| 1.39835 | 0.893411718 |
| 1.3984 | 0.893144095 |
| 1.39845 | 0.892875791 |
| 1.3985 | 0.892606804 |
| 1.39855 | 0.892337133 |
| 1.3986 | 0.892066776 |
| 1.39865 | 0.891795732 |
| 1.3987 | 0.891523997 |
| 1.39875 | 0.891251572 |
| 1.3988 | 0.890978453 |
| 1.39885 | 0.890704640 |
| 1.3989 | 0.890430130 |
| 1.39895 | 0.890154922 |
| 1.399 | 0.889879014 |
| 1.39905 | 0.889602404 |
| 1.3991 | 0.889325091 |
| 1.39915 | 0.889047072 |
| 1.3992 | 0.888768346 |
| 1.39925 | 0.888488911 |
| 1.3993 | 0.888208766 |
| 1.39935 | 0.887927907 |
| 1.3994 | 0.887646335 |
| 1.39945 | 0.887364046 |
| 1.3995 | 0.887081040 |
| 1.39955 | 0.886797313 |
| 1.3996 | 0.886512865 |
| 1.39965 | 0.886227694 |
| 1.3997 | 0.885941797 |
| 1.39975 | 0.885655174 |
| 1.3998 | 0.885367821 |
| 1.39985 | 0.885079738 |
| 1.3999 | 0.884790922 |
| 1.39995 | 0.884501372 |
| 1.4 | 0.884211085 |
| 1.40005 | 0.883920061 |
| 1.4001 | 0.883628296 |
| 1.40015 | 0.883335789 |
| 1.4002 | 0.883042539 |
| 1.40025 | 0.882748543 |
| 1.4003 | 0.882453800 |
| 1.40035 | 0.882158307 |
| 1.4004 | 0.881862063 |
| 1.40045 | 0.881565065 |
| 1.4005 | 0.881267313 |
| 1.40055 | 0.880968804 |
| 1.4006 | 0.880669535 |
| 1.40065 | 0.880369506 |
| 1.4007 | 0.880068715 |
| 1.40075 | 0.879767158 |
| 1.4008 | 0.879464835 |
| 1.40085 | 0.879161744 |
| 1.4009 | 0.878857882 |
| 1.40095 | 0.878553248 |
| 1.401 | 0.878247839 |
| 1.40105 | 0.877941654 |
| 1.4011 | 0.877634691 |
| 1.40115 | 0.877326948 |
| 1.4012 | 0.877018423 |
| 1.40125 | 0.876709113 |
| 1.4013 | 0.876399018 |
| 1.40135 | 0.876088134 |
| 1.4014 | 0.875776460 |
| 1.40145 | 0.875463995 |
| 1.4015 | 0.875150735 |
| 1.40155 | 0.874836679 |
| 1.4016 | 0.874521825 |
| 1.40165 | 0.874206171 |
| 1.4017 | 0.873889715 |
| 1.40175 | 0.873572456 |
| 1.4018 | 0.873254390 |
| 1.40185 | 0.872935515 |
| 1.4019 | 0.872615831 |
| 1.40195 | 0.872295335 |
| 1.402 | 0.871974024 |
| 1.40205 | 0.871651897 |
| 1.4021 | 0.871328952 |
| 1.40215 | 0.871005186 |
| 1.4022 | 0.870680598 |
| 1.40225 | 0.870355186 |
| 1.4023 | 0.870028947 |
| 1.40235 | 0.869701879 |
| 1.4024 | 0.869373981 |
| 1.40245 | 0.869045249 |
| 1.4025 | 0.868715683 |
| 1.40255 | 0.868385280 |
| 1.4026 | 0.868054038 |
| 1.40265 | 0.867721955 |
| 1.4027 | 0.867389028 |
| 1.40275 | 0.867055256 |
| 1.4028 | 0.866720636 |
| 1.40285 | 0.866385167 |
| 1.4029 | 0.866048846 |
| 1.40295 | 0.865711671 |
| 1.403 | 0.865373640 |
| 1.40305 | 0.865034751 |
| 1.4031 | 0.864695001 |
| 1.40315 | 0.864354389 |
| 1.4032 | 0.864012912 |
| 1.40325 | 0.863670569 |
| 1.4033 | 0.863327356 |
| 1.40335 | 0.862983273 |
| 1.4034 | 0.862638315 |
| 1.40345 | 0.862292483 |
| 1.4035 | 0.861945772 |
| 1.40355 | 0.861598182 |
| 1.4036 | 0.861249710 |
| 1.40365 | 0.860900353 |
| 1.4037 | 0.860550109 |
| 1.40375 | 0.860198977 |
| 1.4038 | 0.859846954 |
| 1.40385 | 0.859494038 |
| 1.4039 | 0.859140226 |
| 1.40395 | 0.858785516 |
| 1.404 | 0.858429907 |
| 1.40405 | 0.858073395 |
| 1.4041 | 0.857715979 |
| 1.40415 | 0.857357657 |
| 1.4042 | 0.856998425 |
| 1.40425 | 0.856638282 |
| 1.4043 | 0.856277225 |
| 1.40435 | 0.855915253 |
| 1.4044 | 0.855552363 |
| 1.40445 | 0.855188552 |
| 1.4045 | 0.854823819 |
| 1.40455 | 0.854458161 |
| 1.4046 | 0.854091575 |
| 1.40465 | 0.853724060 |
| 1.4047 | 0.853355613 |
| 1.40475 | 0.852986232 |
| 1.4048 | 0.852615914 |
| 1.40485 | 0.852244657 |
| 1.4049 | 0.851872459 |
| 1.40495 | 0.851499317 |
| 1.405 | 0.851125230 |
| 1.40505 | 0.850750194 |
| 1.4051 | 0.850374208 |
| 1.40515 | 0.849997268 |
| 1.4052 | 0.849619374 |
| 1.40525 | 0.849240521 |
| 1.4053 | 0.848860708 |
| 1.40535 | 0.848479933 |
| 1.4054 | 0.848098193 |
| 1.40545 | 0.847715485 |
| 1.4055 | 0.847331808 |
| 1.40555 | 0.846947159 |
| 1.4056 | 0.846561535 |
| 1.40565 | 0.846174934 |
| 1.4057 | 0.845787353 |
| 1.40575 | 0.845398791 |
| 1.4058 | 0.845009244 |
| 1.40585 | 0.844618711 |
| 1.4059 | 0.844227188 |
| 1.40595 | 0.843834674 |
| 1.406 | 0.843441166 |
| 1.40605 | 0.843046661 |
| 1.4061 | 0.842651157 |
| 1.40615 | 0.842254651 |
| 1.4062 | 0.841857141 |
| 1.40625 | 0.841458625 |
| 1.4063 | 0.841059100 |
| 1.40635 | 0.840658563 |
| 1.4064 | 0.840257012 |
| 1.40645 | 0.839854445 |
| 1.4065 | 0.839450859 |
| 1.40655 | 0.839046251 |
| 1.4066 | 0.838640618 |
| 1.40665 | 0.838233959 |
| 1.4067 | 0.837826271 |
| 1.40675 | 0.837417551 |
| 1.4068 | 0.837007797 |
| 1.40685 | 0.836597006 |
| 1.4069 | 0.836185176 |
| 1.40695 | 0.835772303 |
| 1.407 | 0.835358386 |
| 1.40705 | 0.834943421 |
| 1.4071 | 0.834527407 |
| 1.40715 | 0.834110340 |
| 1.4072 | 0.833692218 |
| 1.40725 | 0.833273039 |
| 1.4073 | 0.832852800 |
| 1.40735 | 0.832431497 |
| 1.4074 | 0.832009129 |
| 1.40745 | 0.831585693 |
| 1.4075 | 0.831161186 |
| 1.40755 | 0.830735606 |
| 1.4076 | 0.830308950 |
| 1.40765 | 0.829881215 |
| 1.4077 | 0.829452399 |
| 1.40775 | 0.829022498 |
| 1.4078 | 0.828591511 |
| 1.40785 | 0.828159434 |
| 1.4079 | 0.827726266 |
| 1.40795 | 0.827292002 |
| 1.408 | 0.826856641 |
| 1.40805 | 0.826420180 |
| 1.4081 | 0.825982616 |
| 1.40815 | 0.825543946 |
| 1.4082 | 0.825104168 |
| 1.40825 | 0.824663278 |
| 1.4083 | 0.824221275 |
| 1.40835 | 0.823778155 |
| 1.4084 | 0.823333916 |
| 1.40845 | 0.822888555 |
| 1.4085 | 0.822442069 |
| 1.40855 | 0.821994455 |
| 1.4086 | 0.821545711 |
| 1.40865 | 0.821095833 |
| 1.4087 | 0.820644820 |
| 1.40875 | 0.820192667 |
| 1.4088 | 0.819739374 |
| 1.40885 | 0.819284935 |
| 1.4089 | 0.818829350 |
| 1.40895 | 0.818372614 |
| 1.409 | 0.817914726 |
| 1.40905 | 0.817455681 |
| 1.4091 | 0.816995479 |
| 1.40915 | 0.816534115 |
| 1.4092 | 0.816071586 |
| 1.40925 | 0.815607891 |
| 1.4093 | 0.815143025 |
| 1.40935 | 0.814676987 |
| 1.4094 | 0.814209773 |
| 1.40945 | 0.813741380 |
| 1.4095 | 0.813271806 |
| 1.40955 | 0.812801047 |
| 1.4096 | 0.812329101 |
| 1.40965 | 0.811855964 |
| 1.4097 | 0.811381635 |
| 1.40975 | 0.810906109 |
| 1.4098 | 0.810429385 |
| 1.40985 | 0.809951458 |
| 1.4099 | 0.809472327 |
| 1.40995 | 0.808991987 |
| 1.41 | 0.808510437 |
| 1.41005 | 0.808027673 |
| 1.4101 | 0.807543692 |
| 1.41015 | 0.807058492 |
| 1.4102 | 0.806572068 |
| 1.41025 | 0.806084419 |
| 1.4103 | 0.805595542 |
| 1.41035 | 0.805105432 |
| 1.4104 | 0.804614088 |
| 1.41045 | 0.804121506 |
| 1.4105 | 0.803627683 |
| 1.41055 | 0.803132616 |
| 1.4106 | 0.802636303 |
| 1.41065 | 0.802138739 |
| 1.4107 | 0.801639923 |
| 1.41075 | 0.801139850 |
| 1.4108 | 0.800638519 |
| 1.41085 | 0.800135925 |
| 1.4109 | 0.799632066 |
| 1.41095 | 0.799126939 |
| 1.411 | 0.798620540 |
| 1.41105 | 0.798112866 |
| 1.4111 | 0.797603915 |
| 1.41115 | 0.797093683 |
| 1.4112 | 0.796582167 |
| 1.41125 | 0.796069365 |
| 1.4113 | 0.795555272 |
| 1.41135 | 0.795039885 |
| 1.4114 | 0.794523202 |
| 1.41145 | 0.794005220 |
| 1.4115 | 0.793485935 |
| 1.41155 | 0.792965343 |
| 1.4116 | 0.792443443 |
| 1.41165 | 0.791920230 |
| 1.4117 | 0.791395702 |
| 1.41175 | 0.790869855 |
| 1.4118 | 0.790342686 |
| 1.41185 | 0.789814192 |
| 1.4119 | 0.789284369 |
| 1.41195 | 0.788753215 |
| 1.412 | 0.788220726 |
| 1.41205 | 0.787686899 |
| 1.4121 | 0.787151731 |
| 1.41215 | 0.786615218 |
| 1.4122 | 0.786077357 |
| 1.41225 | 0.785538145 |
| 1.4123 | 0.784997579 |
| 1.41235 | 0.784455655 |
| 1.4124 | 0.783912369 |
| 1.41245 | 0.783367720 |
| 1.4125 | 0.782821703 |
| 1.41255 | 0.782274315 |
| 1.4126 | 0.781725553 |
| 1.41265 | 0.781175413 |
| 1.4127 | 0.780623892 |
| 1.41275 | 0.780070987 |
| 1.4128 | 0.779516694 |
| 1.41285 | 0.778961011 |
| 1.4129 | 0.778403933 |
| 1.41295 | 0.777845457 |
| 1.413 | 0.777285580 |
| 1.41305 | 0.776724299 |
| 1.4131 | 0.776161610 |
| 1.41315 | 0.775597509 |
| 1.4132 | 0.775031994 |
| 1.41325 | 0.774465061 |
| 1.4133 | 0.773896706 |
| 1.41335 | 0.773326926 |
| 1.4134 | 0.772755718 |
| 1.41345 | 0.772183077 |
| 1.4135 | 0.771609002 |
| 1.41355 | 0.771033487 |
| 1.4136 | 0.770456531 |
| 1.41365 | 0.769878128 |
| 1.4137 | 0.769298277 |
| 1.41375 | 0.768716972 |
| 1.4138 | 0.768134212 |
| 1.41385 | 0.767549991 |
| 1.4139 | 0.766964308 |
| 1.41395 | 0.766377157 |
| 1.414 | 0.765788537 |
| 1.41405 | 0.765198442 |
| 1.4141 | 0.764606870 |
| 1.41415 | 0.764013817 |
| 1.4142 | 0.763419279 |
| 1.41425 | 0.762823254 |
| 1.4143 | 0.762225737 |
| 1.41435 | 0.761626724 |
| 1.4144 | 0.761026213 |
| 1.41445 | 0.760424199 |
| 1.4145 | 0.759820679 |
| 1.41455 | 0.759215649 |
| 1.4146 | 0.758609106 |
| 1.41465 | 0.758001046 |
| 1.4147 | 0.757391466 |
| 1.41475 | 0.756780361 |
| 1.4148 | 0.756167728 |
| 1.41485 | 0.755553564 |
| 1.4149 | 0.754937865 |
| 1.41495 | 0.754320627 |
| 1.415 | 0.753701846 |
| 1.41505 | 0.753081519 |
| 1.4151 | 0.752459642 |
| 1.41515 | 0.751836211 |
| 1.4152 | 0.751211223 |
| 1.41525 | 0.750584673 |
| 1.4153 | 0.749956559 |
| 1.41535 | 0.749326876 |
| 1.4154 | 0.748695621 |
| 1.41545 | 0.748062790 |
| 1.4155 | 0.747428378 |
| 1.41555 | 0.746792383 |
| 1.4156 | 0.746154801 |
| 1.41565 | 0.745515628 |
| 1.4157 | 0.744874859 |
| 1.41575 | 0.744232492 |
| 1.4158 | 0.743588522 |
| 1.41585 | 0.742942945 |
| 1.4159 | 0.742295758 |
| 1.41595 | 0.741646957 |
| 1.416 | 0.740996538 |
| 1.41605 | 0.740344497 |
| 1.4161 | 0.739690831 |
| 1.41615 | 0.739035535 |
| 1.4162 | 0.738378605 |
| 1.41625 | 0.737720039 |
| 1.4163 | 0.737059831 |
| 1.41635 | 0.736397977 |
| 1.4164 | 0.735734475 |
| 1.41645 | 0.735069320 |
| 1.4165 | 0.734402508 |
| 1.41655 | 0.733734035 |
| 1.4166 | 0.733063898 |
| 1.41665 | 0.732392091 |
| 1.4167 | 0.731718612 |
| 1.41675 | 0.731043457 |
| 1.4168 | 0.730366621 |
| 1.41685 | 0.729688100 |
| 1.4169 | 0.729007891 |
| 1.41695 | 0.728325989 |
| 1.417 | 0.727642390 |
| 1.41705 | 0.726957091 |
| 1.4171 | 0.726270087 |
| 1.41715 | 0.725581375 |
| 1.4172 | 0.724890950 |
| 1.41725 | 0.724198808 |
| 1.4173 | 0.723504945 |
| 1.41735 | 0.722809358 |
| 1.4174 | 0.722112041 |
| 1.41745 | 0.721412992 |
| 1.4175 | 0.720712205 |
| 1.41755 | 0.720009677 |
| 1.4176 | 0.719305404 |
| 1.41765 | 0.718599382 |
| 1.4177 | 0.717891606 |
| 1.41775 | 0.717182072 |
| 1.4178 | 0.716470776 |
| 1.41785 | 0.715757715 |
| 1.4179 | 0.715042883 |
| 1.41795 | 0.714326277 |
| 1.418 | 0.713607892 |
| 1.41805 | 0.712887725 |
| 1.4181 | 0.712165771 |
| 1.41815 | 0.711442026 |
| 1.4182 | 0.710716486 |
| 1.41825 | 0.709989146 |
| 1.4183 | 0.709260003 |
| 1.41835 | 0.708529052 |
| 1.4184 | 0.707796288 |
| 1.41845 | 0.707061708 |
| 1.4185 | 0.706325308 |
| 1.41855 | 0.705587082 |
| 1.4186 | 0.704847028 |
| 1.41865 | 0.704105140 |
| 1.4187 | 0.703361414 |
| 1.41875 | 0.702615846 |
| 1.4188 | 0.701868431 |
| 1.41885 | 0.701119166 |
| 1.4189 | 0.700368046 |
| 1.41895 | 0.699615067 |
| 1.419 | 0.698860224 |
| 1.41905 | 0.698103513 |
| 1.4191 | 0.697344930 |
| 1.41915 | 0.696584469 |
| 1.4192 | 0.695822128 |
| 1.41925 | 0.695057901 |
| 1.4193 | 0.694291784 |
| 1.41935 | 0.693523773 |
| 1.4194 | 0.692753863 |
| 1.41945 | 0.691982051 |
| 1.4195 | 0.691208330 |
| 1.41955 | 0.690432698 |
| 1.4196 | 0.689655149 |
| 1.41965 | 0.688875679 |
| 1.4197 | 0.688094284 |
| 1.41975 | 0.687310959 |
| 1.4198 | 0.686525700 |
| 1.41985 | 0.685738502 |
| 1.4199 | 0.684949360 |
| 1.41995 | 0.684158271 |
| 1.42 | 0.683365230 |
| 1.42005 | 0.682570232 |
| 1.4201 | 0.681773272 |
| 1.42015 | 0.680974347 |
| 1.4202 | 0.680173452 |
| 1.42025 | 0.679370581 |
| 1.4203 | 0.678565731 |
| 1.42035 | 0.677758897 |
| 1.4204 | 0.676950074 |
| 1.42045 | 0.676139257 |
| 1.4205 | 0.675326444 |
| 1.42055 | 0.674511627 |
| 1.4206 | 0.673694804 |
| 1.42065 | 0.672875968 |
| 1.4207 | 0.672055117 |
| 1.42075 | 0.671232245 |
| 1.4208 | 0.670407347 |
| 1.42085 | 0.669580418 |
| 1.4209 | 0.668751455 |
| 1.42095 | 0.667920452 |
| 1.421 | 0.667087405 |
| 1.42105 | 0.666252309 |
| 1.4211 | 0.665415160 |
| 1.42115 | 0.664575952 |
| 1.4212 | 0.663734681 |
| 1.42125 | 0.662891342 |
| 1.4213 | 0.662045931 |
| 1.42135 | 0.661198442 |
| 1.4214 | 0.660348872 |
| 1.42145 | 0.659497215 |
| 1.4215 | 0.658643466 |
| 1.42155 | 0.657787621 |
| 1.4216 | 0.656929674 |
| 1.42165 | 0.656069622 |
| 1.4217 | 0.655207460 |
| 1.42175 | 0.654343182 |
| 1.4218 | 0.653476783 |
| 1.42185 | 0.652608260 |
| 1.4219 | 0.651737606 |
| 1.42195 | 0.650864818 |
| 1.422 | 0.649989890 |
| 1.42205 | 0.649112818 |
| 1.4221 | 0.648233596 |
| 1.42215 | 0.647352220 |
| 1.4222 | 0.646468685 |
| 1.42225 | 0.645582986 |
| 1.4223 | 0.644695118 |
| 1.42235 | 0.643805076 |
| 1.4224 | 0.642912856 |
| 1.42245 | 0.642018452 |
| 1.4225 | 0.641121859 |
| 1.42255 | 0.640223073 |
| 1.4226 | 0.639322089 |
| 1.42265 | 0.638418901 |
| 1.4227 | 0.637513505 |
| 1.42275 | 0.636605895 |
| 1.4228 | 0.635696067 |
| 1.42285 | 0.634784016 |
| 1.4229 | 0.633869737 |
| 1.42295 | 0.632953224 |
| 1.423 | 0.632034473 |
| 1.42305 | 0.631113478 |
| 1.4231 | 0.630190235 |
| 1.42315 | 0.629264739 |
| 1.4232 | 0.628336984 |
| 1.42325 | 0.627406966 |
| 1.4233 | 0.626474679 |
| 1.42335 | 0.625540119 |
| 1.4234 | 0.624603280 |
| 1.42345 | 0.623664157 |
| 1.4235 | 0.622722745 |
| 1.42355 | 0.621779039 |
| 1.4236 | 0.620833034 |
| 1.42365 | 0.619884725 |
| 1.4237 | 0.618934107 |
| 1.42375 | 0.617981174 |
| 1.4238 | 0.617025922 |
| 1.42385 | 0.616068344 |
| 1.4239 | 0.615108437 |
| 1.42395 | 0.614146195 |
| 1.424 | 0.613181612 |
| 1.42405 | 0.612214684 |
| 1.4241 | 0.611245406 |
| 1.42415 | 0.610273772 |
| 1.4242 | 0.609299776 |
| 1.42425 | 0.608323415 |
| 1.4243 | 0.607344682 |
| 1.42435 | 0.606363572 |
| 1.4244 | 0.605380080 |
| 1.42445 | 0.604394202 |
| 1.4245 | 0.603405930 |
| 1.42455 | 0.602415262 |
| 1.4246 | 0.601422190 |
| 1.42465 | 0.600426710 |
| 1.4247 | 0.599428817 |
| 1.42475 | 0.598428505 |
| 1.4248 | 0.597425769 |
| 1.42485 | 0.596420604 |
| 1.4249 | 0.595413004 |
| 1.42495 | 0.594402964 |
| 1.425 | 0.593390479 |
| 1.42505 | 0.592375543 |
| 1.4251 | 0.591358152 |
| 1.42515 | 0.590338299 |
| 1.4252 | 0.589315979 |
| 1.42525 | 0.588291188 |
| 1.4253 | 0.587263919 |
| 1.42535 | 0.586234168 |
| 1.4254 | 0.585201928 |
| 1.42545 | 0.584167195 |
| 1.4255 | 0.583129963 |
| 1.42555 | 0.582090227 |
| 1.4256 | 0.581047981 |
| 1.42565 | 0.580003221 |
| 1.4257 | 0.578955939 |
| 1.42575 | 0.577906132 |
| 1.4258 | 0.576853794 |
| 1.42585 | 0.575798919 |
| 1.4259 | 0.574741502 |
| 1.42595 | 0.573681537 |
| 1.426 | 0.572619018 |
| 1.42605 | 0.571553942 |
| 1.4261 | 0.570486301 |
| 1.42615 | 0.569416091 |
| 1.4262 | 0.568343306 |
| 1.42625 | 0.567267940 |
| 1.4263 | 0.566189989 |
| 1.42635 | 0.565109446 |
| 1.4264 | 0.564026306 |
| 1.42645 | 0.562940564 |
| 1.4265 | 0.561852213 |
| 1.42655 | 0.560761250 |
| 1.4266 | 0.559667667 |
| 1.42665 | 0.558571460 |
| 1.4267 | 0.557472622 |
| 1.42675 | 0.556371149 |
| 1.4268 | 0.555267035 |
| 1.42685 | 0.554160274 |
| 1.4269 | 0.553050861 |
| 1.42695 | 0.551938790 |
| 1.427 | 0.550824056 |
| 1.42705 | 0.549706652 |
| 1.4271 | 0.548586574 |
| 1.42715 | 0.547463816 |
| 1.4272 | 0.546338372 |
| 1.42725 | 0.545210237 |
| 1.4273 | 0.544079405 |
| 1.42735 | 0.542945871 |
| 1.4274 | 0.541809628 |
| 1.42745 | 0.540670672 |
| 1.4275 | 0.539528996 |
| 1.42755 | 0.538384596 |
| 1.4276 | 0.537237465 |
| 1.42765 | 0.536087597 |
| 1.4277 | 0.534934988 |
| 1.42775 | 0.533779632 |
| 1.4278 | 0.532621522 |
| 1.42785 | 0.531460653 |
| 1.4279 | 0.530297021 |
| 1.42795 | 0.529130618 |
| 1.428 | 0.527961439 |
| 1.42805 | 0.526789480 |
| 1.4281 | 0.525614733 |
| 1.42815 | 0.524437193 |
| 1.4282 | 0.523256856 |
| 1.42825 | 0.522073714 |
| 1.4283 | 0.520887762 |
| 1.42835 | 0.519698996 |
| 1.4284 | 0.518507408 |
| 1.42845 | 0.517312994 |
| 1.4285 | 0.516115747 |
| 1.42855 | 0.514915663 |
| 1.4286 | 0.513712734 |
| 1.42865 | 0.512506957 |
| 1.4287 | 0.511298324 |
| 1.42875 | 0.510086830 |
| 1.4288 | 0.508872471 |
| 1.42885 | 0.507655239 |
| 1.4289 | 0.506435129 |
| 1.42895 | 0.505212135 |
| 1.429 | 0.503986253 |
| 1.42905 | 0.502757475 |
| 1.4291 | 0.501525797 |
| 1.42915 | 0.500291213 |
| 1.4292 | 0.499053717 |
| 1.42925 | 0.497813303 |
| 1.4293 | 0.496569966 |
| 1.42935 | 0.495323699 |
| 1.4294 | 0.494074498 |
| 1.42945 | 0.492822357 |
| 1.4295 | 0.491567269 |
| 1.42955 | 0.490309230 |
| 1.4296 | 0.489048233 |
| 1.42965 | 0.487784273 |
| 1.4297 | 0.486517344 |
| 1.42975 | 0.485247440 |
| 1.4298 | 0.483974557 |
| 1.42985 | 0.482698687 |
| 1.4299 | 0.481419826 |
| 1.42995 | 0.480137967 |
| 1.43 | 0.478853105 |
| 1.43005 | 0.477565235 |
| 1.4301 | 0.476274351 |
| 1.43015 | 0.474980446 |
| 1.4302 | 0.473683516 |
| 1.43025 | 0.472383554 |
| 1.4303 | 0.471080556 |
| 1.43035 | 0.469774515 |
| 1.4304 | 0.468465426 |
| 1.43045 | 0.467153283 |
| 1.4305 | 0.465838080 |
| 1.43055 | 0.464519812 |
| 1.4306 | 0.463198473 |
| 1.43065 | 0.461874058 |
| 1.4307 | 0.460546561 |
| 1.43075 | 0.459215977 |
| 1.4308 | 0.457882299 |
| 1.43085 | 0.456545522 |
| 1.4309 | 0.455205641 |
| 1.43095 | 0.453862649 |
| 1.431 | 0.452516542 |
| 1.43105 | 0.451167314 |
| 1.4311 | 0.449814960 |
| 1.43115 | 0.448459472 |
| 1.4312 | 0.447100848 |
| 1.43125 | 0.445739079 |
| 1.4313 | 0.444374162 |
| 1.43135 | 0.443006091 |
| 1.4314 | 0.441634859 |
| 1.43145 | 0.440260462 |
| 1.4315 | 0.438882895 |
| 1.43155 | 0.437502150 |
| 1.4316 | 0.436118224 |
| 1.43165 | 0.434731111 |
| 1.4317 | 0.433340805 |
| 1.43175 | 0.431947301 |
| 1.4318 | 0.430550593 |
| 1.43185 | 0.429150676 |
| 1.4319 | 0.427747545 |
| 1.43195 | 0.426341194 |
| 1.432 | 0.424931618 |
| 1.43205 | 0.423518812 |
| 1.4321 | 0.422102770 |
| 1.43215 | 0.420683486 |
| 1.4322 | 0.419260956 |
| 1.43225 | 0.417835175 |
| 1.4323 | 0.416406136 |
| 1.43235 | 0.414973835 |
| 1.4324 | 0.413538267 |
| 1.43245 | 0.412099426 |
| 1.4325 | 0.410657307 |
| 1.43255 | 0.409211906 |
| 1.4326 | 0.407763215 |
| 1.43265 | 0.406311232 |
| 1.4327 | 0.404855950 |
| 1.43275 | 0.403397365 |
| 1.4328 | 0.401935470 |
| 1.43285 | 0.400470262 |
| 1.4329 | 0.399001736 |
| 1.43295 | 0.397529886 |
| 1.433 | 0.396054706 |
| 1.43305 | 0.394576194 |
| 1.4331 | 0.393094342 |
| 1.43315 | 0.391609147 |
| 1.4332 | 0.390120604 |
| 1.43325 | 0.388628707 |
| 1.4333 | 0.387133453 |
| 1.43335 | 0.385634835 |
| 1.4334 | 0.384132850 |
| 1.43345 | 0.382627492 |
| 1.4335 | 0.381118758 |
| 1.43355 | 0.379606641 |
| 1.4336 | 0.378091138 |
| 1.43365 | 0.376572244 |
| 1.4337 | 0.375049955 |
| 1.43375 | 0.373524265 |
| 1.4338 | 0.371995170 |
| 1.43385 | 0.370462667 |
| 1.4339 | 0.368926750 |
| 1.43395 | 0.367387415 |
| 1.434 | 0.365844657 |
| 1.43405 | 0.364298473 |
| 1.4341 | 0.362748858 |
| 1.43415 | 0.361195808 |
| 1.4342 | 0.359639318 |
| 1.43425 | 0.358079385 |
| 1.4343 | 0.356516004 |
| 1.43435 | 0.354949172 |
| 1.4344 | 0.353378884 |
| 1.43445 | 0.351805136 |
| 1.4345 | 0.350227925 |
| 1.43455 | 0.348647246 |
| 1.4346 | 0.347063096 |
| 1.43465 | 0.345475471 |
| 1.4347 | 0.343884367 |
| 1.43475 | 0.342289781 |
| 1.4348 | 0.340691708 |
| 1.43485 | 0.339090146 |
| 1.4349 | 0.337485091 |
| 1.43495 | 0.335876540 |
| 1.435 | 0.334264489 |
| 1.43505 | 0.332648934 |
| 1.4351 | 0.331029873 |
| 1.43515 | 0.329407303 |
| 1.4352 | 0.327781220 |
| 1.43525 | 0.326151621 |
| 1.4353 | 0.324518504 |
| 1.43535 | 0.322881865 |
| 1.4354 | 0.321241701 |
| 1.43545 | 0.319598011 |
| 1.4355 | 0.317950790 |
| 1.43555 | 0.316300038 |
| 1.4356 | 0.314645750 |
| 1.43565 | 0.312987925 |
| 1.4357 | 0.311326560 |
| 1.43575 | 0.309661654 |
| 1.4358 | 0.307993203 |
| 1.43585 | 0.306321206 |
| 1.4359 | 0.304645661 |
| 1.43595 | 0.302966566 |
| 1.436 | 0.301283919 |
| 1.43605 | 0.299597718 |
| 1.4361 | 0.297907962 |
| 1.43615 | 0.296214650 |
| 1.4362 | 0.294517779 |
| 1.43625 | 0.292817350 |
| 1.4363 | 0.291113360 |
| 1.43635 | 0.289405808 |
| 1.4364 | 0.287694694 |
| 1.43645 | 0.285980017 |
| 1.4365 | 0.284261775 |
| 1.43655 | 0.282539970 |
| 1.4366 | 0.280814599 |
| 1.43665 | 0.279085663 |
| 1.4367 | 0.277353162 |
| 1.43675 | 0.275617096 |
| 1.4368 | 0.273877464 |
| 1.43685 | 0.272134267 |
| 1.4369 | 0.270387506 |
| 1.43695 | 0.268637180 |
| 1.437 | 0.266883292 |
| 1.43705 | 0.265125841 |
| 1.4371 | 0.263364828 |
| 1.43715 | 0.261600256 |
| 1.4372 | 0.259832125 |
| 1.43725 | 0.258060437 |
| 1.4373 | 0.256285194 |
| 1.43735 | 0.254506397 |
| 1.4374 | 0.252724049 |
| 1.43745 | 0.250938152 |
| 1.4375 | 0.249148709 |
| 1.43755 | 0.247355722 |
| 1.4376 | 0.245559194 |
| 1.43765 | 0.243759130 |
| 1.4377 | 0.241955531 |
| 1.43775 | 0.240148402 |
| 1.4378 | 0.238337747 |
| 1.43785 | 0.236523569 |
| 1.4379 | 0.234705873 |
| 1.43795 | 0.232884665 |
| 1.438 | 0.231059948 |
| 1.43805 | 0.229231728 |
| 1.4381 | 0.227400010 |
| 1.43815 | 0.225564801 |
| 1.4382 | 0.223726106 |
| 1.43825 | 0.221883931 |
| 1.4383 | 0.220038284 |
| 1.43835 | 0.218189171 |
| 1.4384 | 0.216336600 |
| 1.43845 | 0.214480579 |
| 1.4385 | 0.212621115 |
| 1.43855 | 0.210758217 |
| 1.4386 | 0.208891893 |
| 1.43865 | 0.207022154 |
| 1.4387 | 0.205149009 |
| 1.43875 | 0.203272466 |
| 1.4388 | 0.201392539 |
| 1.43885 | 0.199509236 |
| 1.4389 | 0.197622569 |
| 1.43895 | 0.195732550 |
| 1.439 | 0.193839192 |
| 1.43905 | 0.191942508 |
| 1.4391 | 0.190042509 |
| 1.43915 | 0.188139212 |
| 1.4392 | 0.186232629 |
| 1.43925 | 0.184322776 |
| 1.4393 | 0.182409669 |
| 1.43935 | 0.180493323 |
| 1.4394 | 0.178573756 |
| 1.43945 | 0.176650985 |
| 1.4395 | 0.174725028 |
| 1.43955 | 0.172795904 |
| 1.4396 | 0.170863633 |
| 1.43965 | 0.168928235 |
| 1.4397 | 0.166989731 |
| 1.43975 | 0.165048144 |
| 1.4398 | 0.163103495 |
| 1.43985 | 0.161155810 |
| 1.4399 | 0.159205112 |
| 1.43995 | 0.157251426 |
| 1.44 | 0.155294781 |
| 1.44005 | 0.153335202 |
| 1.4401 | 0.151372719 |
| 1.44015 | 0.149407362 |
| 1.4402 | 0.147439161 |
| 1.44025 | 0.145468148 |
| 1.4403 | 0.143494358 |
| 1.44035 | 0.141517824 |
| 1.4404 | 0.139538583 |
| 1.44045 | 0.137556672 |
| 1.4405 | 0.135572130 |
| 1.44055 | 0.133584998 |
| 1.4406 | 0.131595318 |
| 1.44065 | 0.129603134 |
| 1.4407 | 0.127608492 |
| 1.44075 | 0.125611438 |
| 1.4408 | 0.123612023 |
| 1.44085 | 0.121610298 |
| 1.4409 | 0.119606318 |
| 1.44095 | 0.117600137 |
| 1.441 | 0.115591815 |
| 1.44105 | 0.113581412 |
| 1.4411 | 0.111568992 |
| 1.44115 | 0.109554621 |
| 1.4412 | 0.107538370 |
| 1.44125 | 0.105520309 |
| 1.4413 | 0.103500516 |
| 1.44135 | 0.101479068 |
| 1.4414 | 0.099456050 |
| 1.44145 | 0.097431548 |
| 1.4415 | 0.095405653 |
| 1.44155 | 0.093378461 |
| 1.4416 | 0.091350071 |
| 1.44165 | 0.089320589 |
| 1.4417 | 0.087290124 |
| 1.44175 | 0.085258794 |
| 1.4418 | 0.083226720 |
| 1.44185 | 0.081194032 |
| 1.4419 | 0.079160863 |
| 1.44195 | 0.077127358 |
| 1.442 | 0.075093668 |
| 1.44205 | 0.073059953 |
| 1.4421 | 0.071026381 |
| 1.44215 | 0.068993132 |
| 1.4422 | 0.066960396 |
| 1.44225 | 0.064928374 |
| 1.4423 | 0.062897282 |
| 1.44235 | 0.060867349 |
| 1.4424 | 0.058838818 |
| 1.44245 | 0.056811951 |
| 1.4425 | 0.054787027 |
| 1.44255 | 0.052764345 |
| 1.4426 | 0.050744227 |
| 1.44265 | 0.048727020 |
| 1.4427 | 0.046713099 |
| 1.44275 | 0.044702867 |
| 1.4428 | 0.042696764 |
| 1.44285 | 0.040695268 |
| 1.4429 | 0.038698899 |
| 1.44295 | 0.036708227 |
| 1.443 | 0.034723880 |
| 1.44305 | 0.032746545 |
| 1.4431 | 0.030776989 |
| 1.44315 | 0.028816061 |
| 1.4432 | 0.026864710 |
| 1.44325 | 0.024924002 |
| 1.4433 | 0.022995143 |
| 1.44335 | 0.021079504 |
| 1.4434 | 0.019178656 |
| 1.44345 | 0.017294414 |
| 1.4435 | 0.015428890 |
| 1.44355 | 0.013584571 |
| 1.4436 | 0.011764407 |
| 1.44365 | 0.009971935 |
| 1.4437 | 0.008211424 |
| 1.44375 | 0.006488013 |
| 1.4438 | 0.004807675 |
| 1.44385 | 0.003176225 |
| 1.4439 | 0.001592858 |
| 1.444 | 0 |
| 1.446 | 0.005704331 |
| 1.45 | 0.011328533 |
| 1.454 | 0.015389249 |
| 1.458 | 0.018732861 |
| 1.462 | 0.021630608 |
| 1.466 | 0.024215275 |
| 1.47 | 0.026563987 |
| 1.474 | 0.028726331 |
| 1.478 | 0.030736449 |
| 1.482 | 0.032619056 |
| 1.486 | 0.034392735 |
| 1.49 | 0.036071883 |
| 1.494 | 0.037667931 |
| 1.498 | 0.039190134 |
| 1.502 | 0.040646119 |
| 1.506 | 0.042042258 |
| 1.51 | 0.043383942 |
| 1.514 | 0.044675784 |
| 1.518 | 0.045921764 |
| 1.522 | 0.047125352 |
| 1.526 | 0.048289589 |
| 1.53 | 0.049417163 |
| 1.534 | 0.050510464 |
| 1.538 | 0.051571625 |
| 1.542 | 0.052602565 |
| 1.546 | 0.053605014 |
| 1.55 | 0.054580541 |
| 1.554 | 0.055530571 |
| 1.558 | 0.056456409 |
| 1.562 | 0.057359247 |
| 1.566 | 0.058240182 |
| 1.57 | 0.059100226 |
| 1.574 | 0.059940312 |
| 1.578 | 0.060761307 |
| 1.582 | 0.061564014 |
| 1.586 | 0.062349183 |
| 1.59 | 0.063117511 |
| 1.594 | 0.063869651 |
| 1.598 | 0.064606214 |
| 1.602 | 0.065327774 |
| 1.606 | 0.066034869 |
| 1.61 | 0.066728006 |

**Table 6****.** Simulation results for 2.5-cm sensor over three sensing zones.

| RI @1550 nm | Normalized Power (a.u.) |
| --- | --- |
| 1.3165 | 0.974326489 |
| 1.319 | 0.973695394 |
| 1.3215 | 0.973027911 |
| 1.324 | 0.972320933 |
| 1.3265 | 0.971570985 |
| 1.329 | 0.970774166 |
| 1.3315 | 0.969926075 |
| 1.334 | 0.969021726 |
| 1.3365 | 0.968055441 |
| 1.339 | 0.967020716 |
| 1.3415 | 0.965910046 |
| 1.344 | 0.964714710 |
| 1.3465 | 0.963424475 |
| 1.349 | 0.962027203 |
| 1.3515 | 0.960508295 |
| 1.354 | 0.958849889 |
| 1.3565 | 0.957029627 |
| 1.359 | 0.955018628 |
| 1.3615 | 0.952777843 |
| 1.364 | 0.950250519 |
| 1.3665 | 0.947342865 |
| 1.369 | 0.943848318 |
| 1.37 | 0.942239842 |
| 1.37005 | 0.942148536 |
| 1.3701 | 0.942057043 |
| 1.37015 | 0.941965362 |
| 1.3702 | 0.941873494 |
| 1.37025 | 0.941781437 |
| 1.3703 | 0.941689191 |
| 1.37035 | 0.941596756 |
| 1.3704 | 0.941504132 |
| 1.37045 | 0.941411317 |
| 1.3705 | 0.941318312 |
| 1.37055 | 0.941225116 |
| 1.3706 | 0.941131729 |
| 1.37065 | 0.941038149 |
| 1.3707 | 0.940944378 |
| 1.37075 | 0.940850413 |
| 1.3708 | 0.940756256 |
| 1.37085 | 0.940661905 |
| 1.3709 | 0.940567360 |
| 1.37095 | 0.940472620 |
| 1.371 | 0.940377686 |
| 1.37105 | 0.940282556 |
| 1.3711 | 0.940187230 |
| 1.37115 | 0.940091707 |
| 1.3712 | 0.939995988 |
| 1.37125 | 0.939900072 |
| 1.3713 | 0.939803958 |
| 1.37135 | 0.939707646 |
| 1.3714 | 0.939611135 |
| 1.37145 | 0.939514426 |
| 1.3715 | 0.939417516 |
| 1.37155 | 0.939320407 |
| 1.3716 | 0.939223097 |
| 1.37165 | 0.939125587 |
| 1.3717 | 0.939027875 |
| 1.37175 | 0.938929961 |
| 1.3718 | 0.938831844 |
| 1.37185 | 0.938733525 |
| 1.3719 | 0.938635003 |
| 1.37195 | 0.938536277 |
| 1.372 | 0.938437347 |
| 1.37205 | 0.938338212 |
| 1.3721 | 0.938238872 |
| 1.37215 | 0.938139326 |
| 1.3722 | 0.938039574 |
| 1.37225 | 0.937939616 |
| 1.3723 | 0.937839450 |
| 1.37235 | 0.937739077 |
| 1.3724 | 0.937638496 |
| 1.37245 | 0.937537706 |
| 1.3725 | 0.937436708 |
| 1.37255 | 0.937335500 |
| 1.3726 | 0.937234082 |
| 1.37265 | 0.937132453 |
| 1.3727 | 0.937030614 |
| 1.37275 | 0.936928563 |
| 1.3728 | 0.936826300 |
| 1.37285 | 0.936723825 |
| 1.3729 | 0.936621137 |
| 1.37295 | 0.936518235 |
| 1.373 | 0.936415120 |
| 1.37305 | 0.936311790 |
| 1.3731 | 0.936208245 |
| 1.37315 | 0.936104485 |
| 1.3732 | 0.936000509 |
| 1.37325 | 0.935896317 |
| 1.3733 | 0.935791908 |
| 1.37335 | 0.935687281 |
| 1.3734 | 0.935582437 |
| 1.37345 | 0.935477374 |
| 1.3735 | 0.935372092 |
| 1.37355 | 0.935266591 |
| 1.3736 | 0.935160869 |
| 1.37365 | 0.935054928 |
| 1.3737 | 0.934948765 |
| 1.37375 | 0.934842381 |
| 1.3738 | 0.934735775 |
| 1.37385 | 0.934628946 |
| 1.3739 | 0.934521895 |
| 1.37395 | 0.934414620 |
| 1.374 | 0.934307121 |
| 1.37405 | 0.934199397 |
| 1.3741 | 0.934091448 |
| 1.37415 | 0.933983274 |
| 1.3742 | 0.933874873 |
| 1.37425 | 0.933766246 |
| 1.3743 | 0.933657391 |
| 1.37435 | 0.933548309 |
| 1.3744 | 0.933438999 |
| 1.37445 | 0.933329460 |
| 1.3745 | 0.933219691 |
| 1.37455 | 0.933109693 |
| 1.3746 | 0.932999464 |
| 1.37465 | 0.932889005 |
| 1.3747 | 0.932778314 |
| 1.37475 | 0.932667390 |
| 1.3748 | 0.932556235 |
| 1.37485 | 0.932444846 |
| 1.3749 | 0.932333224 |
| 1.37495 | 0.932221368 |
| 1.375 | 0.932109276 |
| 1.37505 | 0.931996950 |
| 1.3751 | 0.931884388 |
| 1.37515 | 0.931771589 |
| 1.3752 | 0.931658554 |
| 1.37525 | 0.931545281 |
| 1.3753 | 0.931431771 |
| 1.37535 | 0.931318021 |
| 1.3754 | 0.931204033 |
| 1.37545 | 0.931089805 |
| 1.3755 | 0.930975336 |
| 1.37555 | 0.930860627 |
| 1.3756 | 0.930745677 |
| 1.37565 | 0.930630485 |
| 1.3757 | 0.930515050 |
| 1.37575 | 0.930399372 |
| 1.3758 | 0.930283451 |
| 1.37585 | 0.930167285 |
| 1.3759 | 0.930050875 |
| 1.37595 | 0.929934220 |
| 1.376 | 0.929817318 |
| 1.37605 | 0.929700171 |
| 1.3761 | 0.929582776 |
| 1.37615 | 0.929465134 |
| 1.3762 | 0.929347243 |
| 1.37625 | 0.929229104 |
| 1.3763 | 0.929110716 |
| 1.37635 | 0.928992077 |
| 1.3764 | 0.928873188 |
| 1.37645 | 0.928754048 |
| 1.3765 | 0.928634657 |
| 1.37655 | 0.928515013 |
| 1.3766 | 0.928395117 |
| 1.37665 | 0.928274967 |
| 1.3767 | 0.928154563 |
| 1.37675 | 0.928033904 |
| 1.3768 | 0.927912991 |
| 1.37685 | 0.927791821 |
| 1.3769 | 0.927670395 |
| 1.37695 | 0.927548713 |
| 1.377 | 0.927426772 |
| 1.37705 | 0.927304574 |
| 1.3771 | 0.927182117 |
| 1.37715 | 0.927059400 |
| 1.3772 | 0.926936424 |
| 1.37725 | 0.926813187 |
| 1.3773 | 0.926689689 |
| 1.37735 | 0.926565929 |
| 1.3774 | 0.926441906 |
| 1.37745 | 0.926317621 |
| 1.3775 | 0.926193072 |
| 1.37755 | 0.926068259 |
| 1.3776 | 0.925943181 |
| 1.37765 | 0.925817838 |
| 1.3777 | 0.925692228 |
| 1.37775 | 0.925566352 |
| 1.3778 | 0.925440208 |
| 1.37785 | 0.925313797 |
| 1.3779 | 0.925187117 |
| 1.37795 | 0.925060168 |
| 1.378 | 0.924932949 |
| 1.37805 | 0.924805460 |
| 1.3781 | 0.924677699 |
| 1.37815 | 0.924549667 |
| 1.3782 | 0.924421363 |
| 1.37825 | 0.924292785 |
| 1.3783 | 0.924163934 |
| 1.37835 | 0.924034809 |
| 1.3784 | 0.923905409 |
| 1.37845 | 0.923775733 |
| 1.3785 | 0.923645782 |
| 1.37855 | 0.923515553 |
| 1.3786 | 0.923385047 |
| 1.37865 | 0.923254263 |
| 1.3787 | 0.923123200 |
| 1.37875 | 0.922991858 |
| 1.3788 | 0.922860236 |
| 1.37885 | 0.922728333 |
| 1.3789 | 0.922596149 |
| 1.37895 | 0.922463683 |
| 1.379 | 0.922330934 |
| 1.37905 | 0.922197902 |
| 1.3791 | 0.922064586 |
| 1.37915 | 0.921930985 |
| 1.3792 | 0.921797099 |
| 1.37925 | 0.921662927 |
| 1.3793 | 0.921528469 |
| 1.37935 | 0.921393723 |
| 1.3794 | 0.921258689 |
| 1.37945 | 0.921123367 |
| 1.3795 | 0.920987755 |
| 1.37955 | 0.920851853 |
| 1.3796 | 0.920715661 |
| 1.37965 | 0.920579177 |
| 1.3797 | 0.920442402 |
| 1.37975 | 0.920305334 |
| 1.3798 | 0.920167972 |
| 1.37985 | 0.920030316 |
| 1.3799 | 0.919892366 |
| 1.37995 | 0.919754120 |
| 1.38 | 0.919615578 |
| 1.38005 | 0.919476740 |
| 1.3801 | 0.919337603 |
| 1.38015 | 0.919198169 |
| 1.3802 | 0.919058436 |
| 1.38025 | 0.918918403 |
| 1.3803 | 0.918778070 |
| 1.38035 | 0.918637436 |
| 1.3804 | 0.918496501 |
| 1.38045 | 0.918355263 |
| 1.3805 | 0.918213722 |
| 1.38055 | 0.918071877 |
| 1.3806 | 0.917929728 |
| 1.38065 | 0.917787273 |
| 1.3807 | 0.917644513 |
| 1.38075 | 0.917501446 |
| 1.3808 | 0.917358072 |
| 1.38085 | 0.917214389 |
| 1.3809 | 0.917070398 |
| 1.38095 | 0.916926098 |
| 1.381 | 0.916781487 |
| 1.38105 | 0.916636565 |
| 1.3811 | 0.916491332 |
| 1.38115 | 0.916345786 |
| 1.3812 | 0.916199928 |
| 1.38125 | 0.916053755 |
| 1.3813 | 0.915907268 |
| 1.38135 | 0.915760466 |
| 1.3814 | 0.915613347 |
| 1.38145 | 0.915465912 |
| 1.3815 | 0.915318160 |
| 1.38155 | 0.915170089 |
| 1.3816 | 0.915021699 |
| 1.38165 | 0.914872989 |
| 1.3817 | 0.914723960 |
| 1.38175 | 0.914574608 |
| 1.3818 | 0.914424935 |
| 1.38185 | 0.914274939 |
| 1.3819 | 0.914124620 |
| 1.38195 | 0.913973977 |
| 1.382 | 0.913823008 |
| 1.38205 | 0.913671714 |
| 1.3821 | 0.913520094 |
| 1.38215 | 0.913368146 |
| 1.3822 | 0.913215870 |
| 1.38225 | 0.913063265 |
| 1.3823 | 0.912910331 |
| 1.38235 | 0.912757067 |
| 1.3824 | 0.912603472 |
| 1.38245 | 0.912449544 |
| 1.3825 | 0.912295285 |
| 1.38255 | 0.912140691 |
| 1.3826 | 0.911985764 |
| 1.38265 | 0.911830502 |
| 1.3827 | 0.911674904 |
| 1.38275 | 0.911518969 |
| 1.3828 | 0.911362697 |
| 1.38285 | 0.911206088 |
| 1.3829 | 0.911049139 |
| 1.38295 | 0.910891851 |
| 1.383 | 0.910734222 |
| 1.38305 | 0.910576252 |
| 1.3831 | 0.910417940 |
| 1.38315 | 0.910259286 |
| 1.3832 | 0.910100288 |
| 1.38325 | 0.909940945 |
| 1.3833 | 0.909781257 |
| 1.38335 | 0.909621223 |
| 1.3834 | 0.909460842 |
| 1.38345 | 0.909300114 |
| 1.3835 | 0.909139037 |
| 1.38355 | 0.908977612 |
| 1.3836 | 0.908815836 |
| 1.38365 | 0.908653709 |
| 1.3837 | 0.908491230 |
| 1.38375 | 0.908328399 |
| 1.3838 | 0.908165215 |
| 1.38385 | 0.908001677 |
| 1.3839 | 0.907837783 |
| 1.38395 | 0.907673534 |
| 1.384 | 0.907508928 |
| 1.38405 | 0.907343965 |
| 1.3841 | 0.907178643 |
| 1.38415 | 0.907012963 |
| 1.3842 | 0.906846922 |
| 1.38425 | 0.906680521 |
| 1.3843 | 0.906513758 |
| 1.38435 | 0.906346633 |
| 1.3844 | 0.906179144 |
| 1.38445 | 0.906011291 |
| 1.3845 | 0.905843073 |
| 1.38455 | 0.905674490 |
| 1.3846 | 0.905505539 |
| 1.38465 | 0.905336221 |
| 1.3847 | 0.905166535 |
| 1.38475 | 0.904996480 |
| 1.3848 | 0.904826054 |
| 1.38485 | 0.904655257 |
| 1.3849 | 0.904484089 |
| 1.38495 | 0.904312548 |
| 1.385 | 0.904140633 |
| 1.38505 | 0.903968344 |
| 1.3851 | 0.903795679 |
| 1.38515 | 0.903622639 |
| 1.3852 | 0.903449221 |
| 1.38525 | 0.903275426 |
| 1.3853 | 0.903101251 |
| 1.38535 | 0.902926697 |
| 1.3854 | 0.902751762 |
| 1.38545 | 0.902576446 |
| 1.3855 | 0.902400748 |
| 1.38555 | 0.902224666 |
| 1.3856 | 0.902048200 |
| 1.38565 | 0.901871350 |
| 1.3857 | 0.901694113 |
| 1.38575 | 0.901516489 |
| 1.3858 | 0.901338478 |
| 1.38585 | 0.901160078 |
| 1.3859 | 0.900981289 |
| 1.38595 | 0.900802109 |
| 1.386 | 0.900622538 |
| 1.38605 | 0.900442575 |
| 1.3861 | 0.900262218 |
| 1.38615 | 0.900081468 |
| 1.3862 | 0.899900323 |
| 1.38625 | 0.899718781 |
| 1.3863 | 0.899536843 |
| 1.38635 | 0.899354507 |
| 1.3864 | 0.899171773 |
| 1.38645 | 0.898988639 |
| 1.3865 | 0.898805104 |
| 1.38655 | 0.898621168 |
| 1.3866 | 0.898436830 |
| 1.38665 | 0.898252088 |
| 1.3867 | 0.898066943 |
| 1.38675 | 0.897881392 |
| 1.3868 | 0.897695434 |
| 1.38685 | 0.897509070 |
| 1.3869 | 0.897322298 |
| 1.38695 | 0.897135117 |
| 1.387 | 0.896947526 |
| 1.38705 | 0.896759524 |
| 1.3871 | 0.896571110 |
| 1.38715 | 0.896382284 |
| 1.3872 | 0.896193044 |
| 1.38725 | 0.896003389 |
| 1.3873 | 0.895813318 |
| 1.38735 | 0.895622831 |
| 1.3874 | 0.895431927 |
| 1.38745 | 0.895240603 |
| 1.3875 | 0.895048861 |
| 1.38755 | 0.894856698 |
| 1.3876 | 0.894664113 |
| 1.38765 | 0.894471106 |
| 1.3877 | 0.894277675 |
| 1.38775 | 0.894083821 |
| 1.3878 | 0.893889540 |
| 1.38785 | 0.893694834 |
| 1.3879 | 0.893499700 |
| 1.38795 | 0.893304137 |
| 1.388 | 0.893108146 |
| 1.38805 | 0.892911724 |
| 1.3881 | 0.892714870 |
| 1.38815 | 0.892517585 |
| 1.3882 | 0.892319866 |
| 1.38825 | 0.892121712 |
| 1.3883 | 0.891923124 |
| 1.38835 | 0.891724098 |
| 1.3884 | 0.891524636 |
| 1.38845 | 0.891324735 |
| 1.3885 | 0.891124395 |
| 1.38855 | 0.890923614 |
| 1.3886 | 0.890722392 |
| 1.38865 | 0.890520728 |
| 1.3887 | 0.890318620 |
| 1.38875 | 0.890116067 |
| 1.3888 | 0.889913069 |
| 1.38885 | 0.889709624 |
| 1.3889 | 0.889505732 |
| 1.38895 | 0.889301391 |
| 1.389 | 0.889096600 |
| 1.38905 | 0.888891359 |
| 1.3891 | 0.888685666 |
| 1.38915 | 0.888479520 |
| 1.3892 | 0.888272920 |
| 1.38925 | 0.888065865 |
| 1.3893 | 0.887858354 |
| 1.38935 | 0.887650387 |
| 1.3894 | 0.887441961 |
| 1.38945 | 0.887233076 |
| 1.3895 | 0.887023731 |
| 1.38955 | 0.886813924 |
| 1.3896 | 0.886603655 |
| 1.38965 | 0.886392923 |
| 1.3897 | 0.886181726 |
| 1.38975 | 0.885970064 |
| 1.3898 | 0.885757935 |
| 1.38985 | 0.885545338 |
| 1.3899 | 0.885332273 |
| 1.38995 | 0.885118737 |
| 1.39 | 0.884904731 |
| 1.39005 | 0.884690252 |
| 1.3901 | 0.884475301 |
| 1.39015 | 0.884259875 |
| 1.3902 | 0.884043974 |
| 1.39025 | 0.883827596 |
| 1.3903 | 0.883610741 |
| 1.39035 | 0.883393407 |
| 1.3904 | 0.883175593 |
| 1.39045 | 0.882957299 |
| 1.3905 | 0.882738522 |
| 1.39055 | 0.882519263 |
| 1.3906 | 0.882299519 |
| 1.39065 | 0.882079290 |
| 1.3907 | 0.881858574 |
| 1.39075 | 0.881637371 |
| 1.3908 | 0.881415679 |
| 1.39085 | 0.881193497 |
| 1.3909 | 0.880970824 |
| 1.39095 | 0.880747659 |
| 1.391 | 0.880524001 |
| 1.39105 | 0.880299849 |
| 1.3911 | 0.880075201 |
| 1.39115 | 0.879850056 |
| 1.3912 | 0.879624413 |
| 1.39125 | 0.879398272 |
| 1.3913 | 0.879171630 |
| 1.39135 | 0.878944487 |
| 1.3914 | 0.878716842 |
| 1.39145 | 0.878488693 |
| 1.3915 | 0.878260039 |
| 1.39155 | 0.878030880 |
| 1.3916 | 0.877801213 |
| 1.39165 | 0.877571038 |
| 1.3917 | 0.877340354 |
| 1.39175 | 0.877109159 |
| 1.3918 | 0.876877452 |
| 1.39185 | 0.876645232 |
| 1.3919 | 0.876412498 |
| 1.39195 | 0.876179249 |
| 1.392 | 0.875945483 |
| 1.39205 | 0.875711199 |
| 1.3921 | 0.875476397 |
| 1.39215 | 0.875241074 |
| 1.3922 | 0.875005230 |
| 1.39225 | 0.874768864 |
| 1.3923 | 0.874531974 |
| 1.39235 | 0.874294559 |
| 1.3924 | 0.874056617 |
| 1.39245 | 0.873818149 |
| 1.3925 | 0.873579152 |
| 1.39255 | 0.873339625 |
| 1.3926 | 0.873099567 |
| 1.39265 | 0.872858976 |
| 1.3927 | 0.872617853 |
| 1.39275 | 0.872376194 |
| 1.3928 | 0.872134000 |
| 1.39285 | 0.871891269 |
| 1.3929 | 0.871647999 |
| 1.39295 | 0.871404189 |
| 1.393 | 0.871159839 |
| 1.39305 | 0.870914946 |
| 1.3931 | 0.870669510 |
| 1.39315 | 0.870423530 |
| 1.3932 | 0.870177003 |
| 1.39325 | 0.869929930 |
| 1.3933 | 0.869682308 |
| 1.39335 | 0.869434136 |
| 1.3934 | 0.869185414 |
| 1.39345 | 0.868936139 |
| 1.3935 | 0.868686311 |
| 1.39355 | 0.868435928 |
| 1.3936 | 0.868184989 |
| 1.39365 | 0.867933493 |
| 1.3937 | 0.867681438 |
| 1.39375 | 0.867428823 |
| 1.3938 | 0.867175647 |
| 1.39385 | 0.866921909 |
| 1.3939 | 0.866667607 |
| 1.39395 | 0.866412740 |
| 1.394 | 0.866157306 |
| 1.39405 | 0.865901305 |
| 1.3941 | 0.865644735 |
| 1.39415 | 0.865387595 |
| 1.3942 | 0.865129883 |
| 1.39425 | 0.864871598 |
| 1.3943 | 0.864612739 |
| 1.39435 | 0.864353305 |
| 1.3944 | 0.864093294 |
| 1.39445 | 0.863832704 |
| 1.3945 | 0.863571536 |
| 1.39455 | 0.863309786 |
| 1.3946 | 0.863047454 |
| 1.39465 | 0.862784539 |
| 1.3947 | 0.862521038 |
| 1.39475 | 0.862256952 |
| 1.3948 | 0.861992278 |
| 1.39485 | 0.861727015 |
| 1.3949 | 0.861461162 |
| 1.39495 | 0.861194718 |
| 1.395 | 0.860927680 |
| 1.39505 | 0.860660049 |
| 1.3951 | 0.860391821 |
| 1.39515 | 0.860122997 |
| 1.3952 | 0.859853574 |
| 1.39525 | 0.859583551 |
| 1.3953 | 0.859312927 |
| 1.39535 | 0.859041701 |
| 1.3954 | 0.858769871 |
| 1.39545 | 0.858497436 |
| 1.3955 | 0.858224393 |
| 1.39555 | 0.857950743 |
| 1.3956 | 0.857676483 |
| 1.39565 | 0.857401613 |
| 1.3957 | 0.857126130 |
| 1.39575 | 0.856850034 |
| 1.3958 | 0.856573322 |
| 1.39585 | 0.856295994 |
| 1.3959 | 0.856018048 |
| 1.39595 | 0.855739483 |
| 1.396 | 0.855460297 |
| 1.39605 | 0.855180489 |
| 1.3961 | 0.854900057 |
| 1.39615 | 0.854619000 |
| 1.3962 | 0.854337317 |
| 1.39625 | 0.854055006 |
| 1.3963 | 0.853772066 |
| 1.39635 | 0.853488495 |
| 1.3964 | 0.853204292 |
| 1.39645 | 0.852919455 |
| 1.3965 | 0.852633983 |
| 1.39655 | 0.852347875 |
| 1.3966 | 0.852061128 |
| 1.39665 | 0.851773743 |
| 1.3967 | 0.851485716 |
| 1.39675 | 0.851197047 |
| 1.3968 | 0.850907734 |
| 1.39685 | 0.850617775 |
| 1.3969 | 0.850327170 |
| 1.39695 | 0.850035917 |
| 1.397 | 0.849744014 |
| 1.39705 | 0.849451459 |
| 1.3971 | 0.849158252 |
| 1.39715 | 0.848864391 |
| 1.3972 | 0.848569874 |
| 1.39725 | 0.848274700 |
| 1.3973 | 0.847978867 |
| 1.39735 | 0.847682374 |
| 1.3974 | 0.847385219 |
| 1.39745 | 0.847087401 |
| 1.3975 | 0.846788918 |
| 1.39755 | 0.846489769 |
| 1.3976 | 0.846189952 |
| 1.39765 | 0.845889466 |
| 1.3977 | 0.845588309 |
| 1.39775 | 0.845286479 |
| 1.3978 | 0.844983976 |
| 1.39785 | 0.844680797 |
| 1.3979 | 0.844376942 |
| 1.39795 | 0.844072407 |
| 1.398 | 0.843767193 |
| 1.39805 | 0.843461297 |
| 1.3981 | 0.843154718 |
| 1.39815 | 0.842847454 |
| 1.3982 | 0.842539504 |
| 1.39825 | 0.842230867 |
| 1.3983 | 0.841921539 |
| 1.39835 | 0.841611521 |
| 1.3984 | 0.841300810 |
| 1.39845 | 0.840989405 |
| 1.3985 | 0.840677305 |
| 1.39855 | 0.840364507 |
| 1.3986 | 0.840051010 |
| 1.39865 | 0.839736812 |
| 1.3987 | 0.839421913 |
| 1.39875 | 0.839106310 |
| 1.3988 | 0.838790002 |
| 1.39885 | 0.838472986 |
| 1.3989 | 0.838155263 |
| 1.39895 | 0.837836829 |
| 1.399 | 0.837517684 |
| 1.39905 | 0.837197825 |
| 1.3991 | 0.836877251 |
| 1.39915 | 0.836555961 |
| 1.3992 | 0.836233952 |
| 1.39925 | 0.835911224 |
| 1.3993 | 0.835587774 |
| 1.39935 | 0.835263601 |
| 1.3994 | 0.834938704 |
| 1.39945 | 0.834613080 |
| 1.3995 | 0.834286728 |
| 1.39955 | 0.833959646 |
| 1.3996 | 0.833631833 |
| 1.39965 | 0.833303287 |
| 1.3997 | 0.832974006 |
| 1.39975 | 0.832643989 |
| 1.3998 | 0.832313234 |
| 1.39985 | 0.831981739 |
| 1.3999 | 0.831649503 |
| 1.39995 | 0.831316524 |
| 1.4 | 0.830982800 |
| 1.40005 | 0.830648330 |
| 1.4001 | 0.830313112 |
| 1.40015 | 0.829977144 |
| 1.4002 | 0.829640424 |
| 1.40025 | 0.829302951 |
| 1.4003 | 0.828964724 |
| 1.40035 | 0.828625739 |
| 1.4004 | 0.828285997 |
| 1.40045 | 0.827945494 |
| 1.4005 | 0.827604230 |
| 1.40055 | 0.827262202 |
| 1.4006 | 0.826919409 |
| 1.40065 | 0.826575849 |
| 1.4007 | 0.826231520 |
| 1.40075 | 0.825886421 |
| 1.4008 | 0.825540550 |
| 1.40085 | 0.825193904 |
| 1.4009 | 0.824846484 |
| 1.40095 | 0.824498285 |
| 1.401 | 0.824149308 |
| 1.40105 | 0.823799550 |
| 1.4011 | 0.823449009 |
| 1.40115 | 0.823097683 |
| 1.4012 | 0.822745572 |
| 1.40125 | 0.822392672 |
| 1.4013 | 0.822038983 |
| 1.40135 | 0.821684502 |
| 1.4014 | 0.821329227 |
| 1.40145 | 0.820973158 |
| 1.4015 | 0.820616291 |
| 1.40155 | 0.820258626 |
| 1.4016 | 0.819900161 |
| 1.40165 | 0.819540893 |
| 1.4017 | 0.819180821 |
| 1.40175 | 0.818819943 |
| 1.4018 | 0.818458257 |
| 1.40185 | 0.818095761 |
| 1.4019 | 0.817732455 |
| 1.40195 | 0.817368335 |
| 1.402 | 0.817003400 |
| 1.40205 | 0.816637648 |
| 1.4021 | 0.816271077 |
| 1.40215 | 0.815903685 |
| 1.4022 | 0.815535472 |
| 1.40225 | 0.815166434 |
| 1.4023 | 0.814796569 |
| 1.40235 | 0.814425877 |
| 1.4024 | 0.814054355 |
| 1.40245 | 0.813682001 |
| 1.4025 | 0.813308814 |
| 1.40255 | 0.812934791 |
| 1.4026 | 0.812559931 |
| 1.40265 | 0.812184232 |
| 1.4027 | 0.811807691 |
| 1.40275 | 0.811430308 |
| 1.4028 | 0.811052079 |
| 1.40285 | 0.810673004 |
| 1.4029 | 0.810293080 |
| 1.40295 | 0.809912306 |
| 1.403 | 0.809530679 |
| 1.40305 | 0.809148198 |
| 1.4031 | 0.808764860 |
| 1.40315 | 0.808380664 |
| 1.4032 | 0.807995608 |
| 1.40325 | 0.807609690 |
| 1.4033 | 0.807222908 |
| 1.40335 | 0.806835259 |
| 1.4034 | 0.806446743 |
| 1.40345 | 0.806057357 |
| 1.4035 | 0.805667100 |
| 1.40355 | 0.805275968 |
| 1.4036 | 0.804883961 |
| 1.40365 | 0.804491076 |
| 1.4037 | 0.804097312 |
| 1.40375 | 0.803702666 |
| 1.4038 | 0.803307136 |
| 1.40385 | 0.802910721 |
| 1.4039 | 0.802513418 |
| 1.40395 | 0.802115226 |
| 1.404 | 0.801716142 |
| 1.40405 | 0.801316165 |
| 1.4041 | 0.800915292 |
| 1.40415 | 0.800513522 |
| 1.4042 | 0.800110853 |
| 1.40425 | 0.799707281 |
| 1.4043 | 0.799302807 |
| 1.40435 | 0.798897427 |
| 1.4044 | 0.798491139 |
| 1.40445 | 0.798083942 |
| 1.4045 | 0.797675833 |
| 1.40455 | 0.797266811 |
| 1.4046 | 0.796856872 |
| 1.40465 | 0.796446017 |
| 1.4047 | 0.796034241 |
| 1.40475 | 0.795621544 |
| 1.4048 | 0.795207922 |
| 1.40485 | 0.794793375 |
| 1.4049 | 0.794377900 |
| 1.40495 | 0.793961495 |
| 1.405 | 0.793544158 |
| 1.40505 | 0.793125887 |
| 1.4051 | 0.792706679 |
| 1.40515 | 0.792286533 |
| 1.4052 | 0.791865447 |
| 1.40525 | 0.791443418 |
| 1.4053 | 0.791020444 |
| 1.40535 | 0.790596524 |
| 1.4054 | 0.790171655 |
| 1.40545 | 0.789745835 |
| 1.4055 | 0.789319062 |
| 1.40555 | 0.788891334 |
| 1.4056 | 0.788462649 |
| 1.40565 | 0.788033005 |
| 1.4057 | 0.787602398 |
| 1.40575 | 0.787170829 |
| 1.4058 | 0.786738293 |
| 1.40585 | 0.786304790 |
| 1.4059 | 0.785870316 |
| 1.40595 | 0.785434870 |
| 1.406 | 0.784998450 |
| 1.40605 | 0.784561054 |
| 1.4061 | 0.784122678 |
| 1.40615 | 0.783683322 |
| 1.4062 | 0.783242983 |
| 1.40625 | 0.782801659 |
| 1.4063 | 0.782359347 |
| 1.40635 | 0.781916046 |
| 1.4064 | 0.781471753 |
| 1.40645 | 0.781026466 |
| 1.4065 | 0.780580183 |
| 1.40655 | 0.780132902 |
| 1.4066 | 0.779684620 |
| 1.40665 | 0.779235335 |
| 1.4067 | 0.778785046 |
| 1.40675 | 0.778333749 |
| 1.4068 | 0.777881443 |
| 1.40685 | 0.777428125 |
| 1.4069 | 0.776973794 |
| 1.40695 | 0.776518446 |
| 1.407 | 0.776062080 |
| 1.40705 | 0.775604694 |
| 1.4071 | 0.775146285 |
| 1.40715 | 0.774686850 |
| 1.4072 | 0.774226389 |
| 1.40725 | 0.773764898 |
| 1.4073 | 0.773302375 |
| 1.40735 | 0.772838818 |
| 1.4074 | 0.772374224 |
| 1.40745 | 0.771908592 |
| 1.4075 | 0.771441919 |
| 1.40755 | 0.770974203 |
| 1.4076 | 0.770505441 |
| 1.40765 | 0.770035631 |
| 1.4077 | 0.769564771 |
| 1.40775 | 0.769092859 |
| 1.4078 | 0.768619892 |
| 1.40785 | 0.768145868 |
| 1.4079 | 0.767670785 |
| 1.40795 | 0.767194640 |
| 1.408 | 0.766717431 |
| 1.40805 | 0.766239156 |
| 1.4081 | 0.765759812 |
| 1.40815 | 0.765279397 |
| 1.4082 | 0.764797909 |
| 1.40825 | 0.764315345 |
| 1.4083 | 0.763831703 |
| 1.40835 | 0.763346980 |
| 1.4084 | 0.762861175 |
| 1.40845 | 0.762374285 |
| 1.4085 | 0.761886307 |
| 1.40855 | 0.761397239 |
| 1.4086 | 0.760907079 |
| 1.40865 | 0.760415825 |
| 1.4087 | 0.759923473 |
| 1.40875 | 0.759430022 |
| 1.4088 | 0.758935469 |
| 1.40885 | 0.758439812 |
| 1.4089 | 0.757943048 |
| 1.40895 | 0.757445175 |
| 1.409 | 0.756946191 |
| 1.40905 | 0.756446093 |
| 1.4091 | 0.755944878 |
| 1.40915 | 0.755442545 |
| 1.4092 | 0.754939091 |
| 1.40925 | 0.754434513 |
| 1.4093 | 0.753928809 |
| 1.40935 | 0.753421976 |
| 1.4094 | 0.752914013 |
| 1.40945 | 0.752404916 |
| 1.4095 | 0.751894683 |
| 1.40955 | 0.751383312 |
| 1.4096 | 0.750870801 |
| 1.40965 | 0.750357145 |
| 1.4097 | 0.749842345 |
| 1.40975 | 0.749326396 |
| 1.4098 | 0.748809296 |
| 1.40985 | 0.748291043 |
| 1.4099 | 0.747771634 |
| 1.40995 | 0.747251067 |
| 1.41 | 0.746729340 |
| 1.41005 | 0.746206449 |
| 1.4101 | 0.745682392 |
| 1.41015 | 0.745157167 |
| 1.4102 | 0.744630772 |
| 1.41025 | 0.744103203 |
| 1.4103 | 0.743574458 |
| 1.41035 | 0.743044535 |
| 1.4104 | 0.742513431 |
| 1.41045 | 0.741981143 |
| 1.4105 | 0.741447669 |
| 1.41055 | 0.740913007 |
| 1.4106 | 0.740377154 |
| 1.41065 | 0.739840107 |
| 1.4107 | 0.739301863 |
| 1.41075 | 0.738762421 |
| 1.4108 | 0.738221778 |
| 1.41085 | 0.737679930 |
| 1.4109 | 0.737136876 |
| 1.41095 | 0.736592612 |
| 1.411 | 0.736047137 |
| 1.41105 | 0.735500447 |
| 1.4111 | 0.734952541 |
| 1.41115 | 0.734403414 |
| 1.4112 | 0.733853066 |
| 1.41125 | 0.733301492 |
| 1.4113 | 0.732748692 |
| 1.41135 | 0.732194661 |
| 1.4114 | 0.731639397 |
| 1.41145 | 0.731082898 |
| 1.4115 | 0.730525161 |
| 1.41155 | 0.729966183 |
| 1.4116 | 0.729405962 |
| 1.41165 | 0.728844495 |
| 1.4117 | 0.728281779 |
| 1.41175 | 0.727717812 |
| 1.4118 | 0.727152591 |
| 1.41185 | 0.726586113 |
| 1.4119 | 0.726018376 |
| 1.41195 | 0.725449377 |
| 1.412 | 0.724879114 |
| 1.41205 | 0.724307582 |
| 1.4121 | 0.723734781 |
| 1.41215 | 0.723160707 |
| 1.4122 | 0.722585357 |
| 1.41225 | 0.722008729 |
| 1.4123 | 0.721430821 |
| 1.41235 | 0.720851628 |
| 1.4124 | 0.720271149 |
| 1.41245 | 0.719689381 |
| 1.4125 | 0.719106321 |
| 1.41255 | 0.718521967 |
| 1.4126 | 0.717936315 |
| 1.41265 | 0.717349363 |
| 1.4127 | 0.716761108 |
| 1.41275 | 0.716171547 |
| 1.4128 | 0.715580679 |
| 1.41285 | 0.714988499 |
| 1.4129 | 0.714395005 |
| 1.41295 | 0.713800194 |
| 1.413 | 0.713204064 |
| 1.41305 | 0.712606611 |
| 1.4131 | 0.712007834 |
| 1.41315 | 0.711407729 |
| 1.4132 | 0.710806293 |
| 1.41325 | 0.710203523 |
| 1.4133 | 0.709599417 |
| 1.41335 | 0.708993973 |
| 1.4134 | 0.708387186 |
| 1.41345 | 0.707779054 |
| 1.4135 | 0.707169575 |
| 1.41355 | 0.706558746 |
| 1.4136 | 0.705946563 |
| 1.41365 | 0.705333025 |
| 1.4137 | 0.704718127 |
| 1.41375 | 0.704101868 |
| 1.4138 | 0.703484244 |
| 1.41385 | 0.702865252 |
| 1.4139 | 0.702244890 |
| 1.41395 | 0.701623155 |
| 1.414 | 0.701000044 |
| 1.41405 | 0.700375554 |
| 1.4141 | 0.699749682 |
| 1.41415 | 0.699122425 |
| 1.4142 | 0.698493780 |
| 1.41425 | 0.697863745 |
| 1.4143 | 0.697232317 |
| 1.41435 | 0.696599492 |
| 1.4144 | 0.695965268 |
| 1.41445 | 0.695329642 |
| 1.4145 | 0.694692611 |
| 1.41455 | 0.694054172 |
| 1.4146 | 0.693414321 |
| 1.41465 | 0.692773057 |
| 1.4147 | 0.692130376 |
| 1.41475 | 0.691486276 |
| 1.4148 | 0.690840752 |
| 1.41485 | 0.690193803 |
| 1.4149 | 0.689545426 |
| 1.41495 | 0.688895617 |
| 1.415 | 0.688244373 |
| 1.41505 | 0.687591692 |
| 1.4151 | 0.686937570 |
| 1.41515 | 0.686282005 |
| 1.4152 | 0.685624993 |
| 1.41525 | 0.684966532 |
| 1.4153 | 0.684306619 |
| 1.41535 | 0.683645250 |
| 1.4154 | 0.682982423 |
| 1.41545 | 0.682318134 |
| 1.4155 | 0.681652381 |
| 1.41555 | 0.680985161 |
| 1.4156 | 0.680316470 |
| 1.41565 | 0.679646305 |
| 1.4157 | 0.678974665 |
| 1.41575 | 0.678301544 |
| 1.4158 | 0.677626941 |
| 1.41585 | 0.676950852 |
| 1.4159 | 0.676273275 |
| 1.41595 | 0.675594206 |
| 1.416 | 0.674913642 |
| 1.41605 | 0.674231581 |
| 1.4161 | 0.673548018 |
| 1.41615 | 0.672862952 |
| 1.4162 | 0.672176379 |
| 1.41625 | 0.671488295 |
| 1.4163 | 0.670798698 |
| 1.41635 | 0.670107586 |
| 1.4164 | 0.669414953 |
| 1.41645 | 0.668720799 |
| 1.4165 | 0.668025119 |
| 1.41655 | 0.667327910 |
| 1.4166 | 0.666629169 |
| 1.41665 | 0.665928894 |
| 1.4167 | 0.665227081 |
| 1.41675 | 0.664523726 |
| 1.4168 | 0.663818828 |
| 1.41685 | 0.663112382 |
| 1.4169 | 0.662404386 |
| 1.41695 | 0.661694836 |
| 1.417 | 0.660983729 |
| 1.41705 | 0.660271063 |
| 1.4171 | 0.659556833 |
| 1.41715 | 0.658841038 |
| 1.4172 | 0.658123673 |
| 1.41725 | 0.657404735 |
| 1.4173 | 0.656684223 |
| 1.41735 | 0.655962131 |
| 1.4174 | 0.655238457 |
| 1.41745 | 0.654513198 |
| 1.4175 | 0.653786351 |
| 1.41755 | 0.653057912 |
| 1.4176 | 0.652327879 |
| 1.41765 | 0.651596248 |
| 1.4177 | 0.650863016 |
| 1.41775 | 0.650128179 |
| 1.4178 | 0.649391735 |
| 1.41785 | 0.648653680 |
| 1.4179 | 0.647914012 |
| 1.41795 | 0.647172726 |
| 1.418 | 0.646429820 |
| 1.41805 | 0.645685291 |
| 1.4181 | 0.644939135 |
| 1.41815 | 0.644191348 |
| 1.4182 | 0.643441929 |
| 1.41825 | 0.642690873 |
| 1.4183 | 0.641938177 |
| 1.41835 | 0.641183839 |
| 1.4184 | 0.640427854 |
| 1.41845 | 0.639670220 |
| 1.4185 | 0.638910933 |
| 1.41855 | 0.638149990 |
| 1.4186 | 0.637387388 |
| 1.41865 | 0.636623124 |
| 1.4187 | 0.635857193 |
| 1.41875 | 0.635089594 |
| 1.4188 | 0.634320322 |
| 1.41885 | 0.633549375 |
| 1.4189 | 0.632776749 |
| 1.41895 | 0.632002441 |
| 1.419 | 0.631226447 |
| 1.41905 | 0.630448765 |
| 1.4191 | 0.629669390 |
| 1.41915 | 0.628888320 |
| 1.4192 | 0.628105552 |
| 1.41925 | 0.627321081 |
| 1.4193 | 0.626534906 |
| 1.41935 | 0.625747022 |
| 1.4194 | 0.624957425 |
| 1.41945 | 0.624166114 |
| 1.4195 | 0.623373084 |
| 1.41955 | 0.622578332 |
| 1.4196 | 0.621781855 |
| 1.41965 | 0.620983650 |
| 1.4197 | 0.620183713 |
| 1.41975 | 0.619382040 |
| 1.4198 | 0.618578629 |
| 1.41985 | 0.617773476 |
| 1.4199 | 0.616966577 |
| 1.41995 | 0.616157931 |
| 1.42 | 0.615347532 |
| 1.42005 | 0.614535378 |
| 1.4201 | 0.613721465 |
| 1.42015 | 0.612905790 |
| 1.4202 | 0.612088350 |
| 1.42025 | 0.611269140 |
| 1.4203 | 0.610448159 |
| 1.42035 | 0.609625402 |
| 1.4204 | 0.608800867 |
| 1.42045 | 0.607974549 |
| 1.4205 | 0.607146445 |
| 1.42055 | 0.606316553 |
| 1.4206 | 0.605484868 |
| 1.42065 | 0.604651387 |
| 1.4207 | 0.603816106 |
| 1.42075 | 0.602979024 |
| 1.4208 | 0.602140135 |
| 1.42085 | 0.601299437 |
| 1.4209 | 0.600456926 |
| 1.42095 | 0.599612598 |
| 1.421 | 0.598766451 |
| 1.42105 | 0.597918481 |
| 1.4211 | 0.597068685 |
| 1.42115 | 0.596217059 |
| 1.4212 | 0.595363599 |
| 1.42125 | 0.594508303 |
| 1.4213 | 0.593651166 |
| 1.42135 | 0.592792186 |
| 1.4214 | 0.591931359 |
| 1.42145 | 0.591068682 |
| 1.4215 | 0.590204151 |
| 1.42155 | 0.589337762 |
| 1.4216 | 0.588469513 |
| 1.42165 | 0.587599400 |
| 1.4217 | 0.586727419 |
| 1.42175 | 0.585853567 |
| 1.4218 | 0.584977840 |
| 1.42185 | 0.584100236 |
| 1.4219 | 0.583220751 |
| 1.42195 | 0.582339380 |
| 1.422 | 0.581456122 |
| 1.42205 | 0.580570971 |
| 1.4221 | 0.579683926 |
| 1.42215 | 0.578794982 |
| 1.4222 | 0.577904136 |
| 1.42225 | 0.577011384 |
| 1.4223 | 0.576116724 |
| 1.42235 | 0.575220151 |
| 1.4224 | 0.574321663 |
| 1.42245 | 0.573421255 |
| 1.4225 | 0.572518924 |
| 1.42255 | 0.571614667 |
| 1.4226 | 0.570708481 |
| 1.42265 | 0.569800361 |
| 1.4227 | 0.568890305 |
| 1.42275 | 0.567978308 |
| 1.4228 | 0.567064368 |
| 1.42285 | 0.566148481 |
| 1.4229 | 0.565230644 |
| 1.42295 | 0.564310853 |
| 1.423 | 0.563389104 |
| 1.42305 | 0.562465395 |
| 1.4231 | 0.561539721 |
| 1.42315 | 0.560612080 |
| 1.4232 | 0.559682467 |
| 1.42325 | 0.558750880 |
| 1.4233 | 0.557817314 |
| 1.42335 | 0.556881767 |
| 1.4234 | 0.555944234 |
| 1.42345 | 0.555004713 |
| 1.4235 | 0.554063201 |
| 1.42355 | 0.553119692 |
| 1.4236 | 0.552174185 |
| 1.42365 | 0.551226675 |
| 1.4237 | 0.550277159 |
| 1.42375 | 0.549325634 |
| 1.4238 | 0.548372097 |
| 1.42385 | 0.547416542 |
| 1.4239 | 0.546458969 |
| 1.42395 | 0.545499372 |
| 1.424 | 0.544537748 |
| 1.42405 | 0.543574094 |
| 1.4241 | 0.542608407 |
| 1.42415 | 0.541640683 |
| 1.4242 | 0.540670918 |
| 1.42425 | 0.539699109 |
| 1.4243 | 0.538725253 |
| 1.42435 | 0.537749346 |
| 1.4244 | 0.536771385 |
| 1.42445 | 0.535791366 |
| 1.4245 | 0.534809286 |
| 1.42455 | 0.533825142 |
| 1.4246 | 0.532838929 |
| 1.42465 | 0.531850645 |
| 1.4247 | 0.530860286 |
| 1.42475 | 0.529867849 |
| 1.4248 | 0.528873330 |
| 1.42485 | 0.527876725 |
| 1.4249 | 0.526878032 |
| 1.42495 | 0.525877247 |
| 1.425 | 0.524874367 |
| 1.42505 | 0.523869387 |
| 1.4251 | 0.522862306 |
| 1.42515 | 0.521853118 |
| 1.4252 | 0.520841822 |
| 1.42525 | 0.519828413 |
| 1.4253 | 0.518812888 |
| 1.42535 | 0.517795244 |
| 1.4254 | 0.516775477 |
| 1.42545 | 0.515753583 |
| 1.4255 | 0.514729561 |
| 1.42555 | 0.513703405 |
| 1.4256 | 0.512675113 |
| 1.42565 | 0.511644682 |
| 1.4257 | 0.510612108 |
| 1.42575 | 0.509577387 |
| 1.4258 | 0.508540516 |
| 1.42585 | 0.507501492 |
| 1.4259 | 0.506460312 |
| 1.42595 | 0.505416972 |
| 1.426 | 0.504371469 |
| 1.42605 | 0.503323800 |
| 1.4261 | 0.502273961 |
| 1.42615 | 0.501221948 |
| 1.4262 | 0.500167759 |
| 1.42625 | 0.499111390 |
| 1.4263 | 0.498052839 |
| 1.42635 | 0.496992101 |
| 1.4264 | 0.495929173 |
| 1.42645 | 0.494864052 |
| 1.4265 | 0.493796735 |
| 1.42655 | 0.492727218 |
| 1.4266 | 0.491655499 |
| 1.42665 | 0.490581573 |
| 1.4267 | 0.489505439 |
| 1.42675 | 0.488427091 |
| 1.4268 | 0.487346528 |
| 1.42685 | 0.486263746 |
| 1.4269 | 0.485178741 |
| 1.42695 | 0.484091511 |
| 1.427 | 0.483002053 |
| 1.42705 | 0.481910362 |
| 1.4271 | 0.480816436 |
| 1.42715 | 0.479720272 |
| 1.4272 | 0.478621867 |
| 1.42725 | 0.477521216 |
| 1.4273 | 0.476418318 |
| 1.42735 | 0.475313169 |
| 1.4274 | 0.474205766 |
| 1.42745 | 0.473096106 |
| 1.4275 | 0.471984185 |
| 1.42755 | 0.470870001 |
| 1.4276 | 0.469753550 |
| 1.42765 | 0.468634830 |
| 1.4277 | 0.467513837 |
| 1.42775 | 0.466390568 |
| 1.4278 | 0.465265020 |
| 1.42785 | 0.464137190 |
| 1.4279 | 0.463007076 |
| 1.42795 | 0.461874673 |
| 1.428 | 0.460739979 |
| 1.42805 | 0.459602991 |
| 1.4281 | 0.458463707 |
| 1.42815 | 0.457322122 |
| 1.4282 | 0.456178234 |
| 1.42825 | 0.455032041 |
| 1.4283 | 0.453883538 |
| 1.42835 | 0.452732724 |
| 1.4284 | 0.451579595 |
| 1.42845 | 0.450424149 |
| 1.4285 | 0.449266382 |
| 1.42855 | 0.448106291 |
| 1.4286 | 0.446943875 |
| 1.42865 | 0.445779130 |
| 1.4287 | 0.444612052 |
| 1.42875 | 0.443442640 |
| 1.4288 | 0.442270891 |
| 1.42885 | 0.441096801 |
| 1.4289 | 0.439920369 |
| 1.42895 | 0.438741590 |
| 1.429 | 0.437560463 |
| 1.42905 | 0.436376985 |
| 1.4291 | 0.435191153 |
| 1.42915 | 0.434002965 |
| 1.4292 | 0.432812417 |
| 1.42925 | 0.431619508 |
| 1.4293 | 0.430424234 |
| 1.42935 | 0.429226593 |
| 1.4294 | 0.428026582 |
| 1.42945 | 0.426824199 |
| 1.4295 | 0.425619441 |
| 1.42955 | 0.424412306 |
| 1.4296 | 0.423202791 |
| 1.42965 | 0.421990894 |
| 1.4297 | 0.420776612 |
| 1.42975 | 0.419559943 |
| 1.4298 | 0.418340884 |
| 1.42985 | 0.417119434 |
| 1.4299 | 0.415895588 |
| 1.42995 | 0.414669347 |
| 1.43 | 0.413440706 |
| 1.43005 | 0.412209663 |
| 1.4301 | 0.410976217 |
| 1.43015 | 0.409740366 |
| 1.4302 | 0.408502106 |
| 1.43025 | 0.407261435 |
| 1.4303 | 0.406018352 |
| 1.43035 | 0.404772855 |
| 1.4304 | 0.403524941 |
| 1.43045 | 0.402274607 |
| 1.4305 | 0.401021853 |
| 1.43055 | 0.399766676 |
| 1.4306 | 0.398509074 |
| 1.43065 | 0.397249045 |
| 1.4307 | 0.395986586 |
| 1.43075 | 0.394721697 |
| 1.4308 | 0.393454375 |
| 1.43085 | 0.392184619 |
| 1.4309 | 0.390912426 |
| 1.43095 | 0.389637794 |
| 1.431 | 0.388360723 |
| 1.43105 | 0.387081209 |
| 1.4311 | 0.385799252 |
| 1.43115 | 0.384514850 |
| 1.4312 | 0.383228001 |
| 1.43125 | 0.381938704 |
| 1.4313 | 0.380646957 |
| 1.43135 | 0.379352758 |
| 1.4314 | 0.378056106 |
| 1.43145 | 0.376756999 |
| 1.4315 | 0.375455437 |
| 1.43155 | 0.374151417 |
| 1.4316 | 0.372844939 |
| 1.43165 | 0.371536001 |
| 1.4317 | 0.370224601 |
| 1.43175 | 0.368910739 |
| 1.4318 | 0.367594414 |
| 1.43185 | 0.366275624 |
| 1.4319 | 0.364954368 |
| 1.43195 | 0.363630645 |
| 1.432 | 0.362304454 |
| 1.43205 | 0.360975794 |
| 1.4321 | 0.359644665 |
| 1.43215 | 0.358311065 |
| 1.4322 | 0.356974993 |
| 1.43225 | 0.355636449 |
| 1.4323 | 0.354295432 |
| 1.43235 | 0.352951942 |
| 1.4324 | 0.351605977 |
| 1.43245 | 0.350257538 |
| 1.4325 | 0.348906623 |
| 1.43255 | 0.347553232 |
| 1.4326 | 0.346197365 |
| 1.43265 | 0.344839022 |
| 1.4327 | 0.343478201 |
| 1.43275 | 0.342114904 |
| 1.4328 | 0.340749129 |
| 1.43285 | 0.339380876 |
| 1.4329 | 0.338010147 |
| 1.43295 | 0.336636939 |
| 1.433 | 0.335261254 |
| 1.43305 | 0.333883092 |
| 1.4331 | 0.332502452 |
| 1.43315 | 0.331119336 |
| 1.4332 | 0.329733743 |
| 1.43325 | 0.328345673 |
| 1.4333 | 0.326955128 |
| 1.43335 | 0.325562107 |
| 1.4334 | 0.324166612 |
| 1.43345 | 0.322768642 |
| 1.4335 | 0.321368199 |
| 1.43355 | 0.319965284 |
| 1.4336 | 0.318559897 |
| 1.43365 | 0.317152039 |
| 1.4337 | 0.315741711 |
| 1.43375 | 0.314328915 |
| 1.4338 | 0.312913651 |
| 1.43385 | 0.311495921 |
| 1.4339 | 0.310075726 |
| 1.43395 | 0.308653068 |
| 1.434 | 0.307227948 |
| 1.43405 | 0.305800368 |
| 1.4341 | 0.304370329 |
| 1.43415 | 0.302937833 |
| 1.4342 | 0.301502883 |
| 1.43425 | 0.300065480 |
| 1.4343 | 0.298625625 |
| 1.43435 | 0.297183322 |
| 1.4344 | 0.295738573 |
| 1.43445 | 0.294291379 |
| 1.4345 | 0.292841744 |
| 1.43455 | 0.291389670 |
| 1.4346 | 0.289935160 |
| 1.43465 | 0.288478216 |
| 1.4347 | 0.287018841 |
| 1.43475 | 0.285557039 |
| 1.4348 | 0.284092812 |
| 1.43485 | 0.282626164 |
| 1.4349 | 0.281157097 |
| 1.43495 | 0.279685617 |
| 1.435 | 0.278211725 |
| 1.43505 | 0.276735427 |
| 1.4351 | 0.275256725 |
| 1.43515 | 0.273775623 |
| 1.4352 | 0.272292127 |
| 1.43525 | 0.270806239 |
| 1.4353 | 0.269317965 |
| 1.43535 | 0.267827309 |
| 1.4354 | 0.266334275 |
| 1.43545 | 0.264838869 |
| 1.4355 | 0.263341095 |
| 1.43555 | 0.261840959 |
| 1.4356 | 0.260338465 |
| 1.43565 | 0.258833619 |
| 1.4357 | 0.257326427 |
| 1.43575 | 0.255816895 |
| 1.4358 | 0.254305027 |
| 1.43585 | 0.252790831 |
| 1.4359 | 0.251274313 |
| 1.43595 | 0.249755479 |
| 1.436 | 0.248234335 |
| 1.43605 | 0.246710889 |
| 1.4361 | 0.245185147 |
| 1.43615 | 0.243657117 |
| 1.4362 | 0.242126805 |
| 1.43625 | 0.240594220 |
| 1.4363 | 0.239059369 |
| 1.43635 | 0.237522260 |
| 1.4364 | 0.235982902 |
| 1.43645 | 0.234441302 |
| 1.4365 | 0.232897469 |
| 1.43655 | 0.231351412 |
| 1.4366 | 0.229803141 |
| 1.43665 | 0.228252663 |
| 1.4367 | 0.226699990 |
| 1.43675 | 0.225145130 |
| 1.4368 | 0.223588094 |
| 1.43685 | 0.222028892 |
| 1.4369 | 0.220467534 |
| 1.43695 | 0.218904032 |
| 1.437 | 0.217338396 |
| 1.43705 | 0.215770637 |
| 1.4371 | 0.214200768 |
| 1.43715 | 0.212628800 |
| 1.4372 | 0.211054745 |
| 1.43725 | 0.209478616 |
| 1.4373 | 0.207900426 |
| 1.43735 | 0.206320188 |
| 1.4374 | 0.204737915 |
| 1.43745 | 0.203153621 |
| 1.4375 | 0.201567320 |
| 1.43755 | 0.199979027 |
| 1.4376 | 0.198388756 |
| 1.43765 | 0.196796524 |
| 1.4377 | 0.195202344 |
| 1.43775 | 0.193606234 |
| 1.4378 | 0.192008210 |
| 1.43785 | 0.190408288 |
| 1.4379 | 0.188806485 |
| 1.43795 | 0.187202820 |
| 1.438 | 0.185597310 |
| 1.43805 | 0.183989973 |
| 1.4381 | 0.182380829 |
| 1.43815 | 0.180769897 |
| 1.4382 | 0.179157196 |
| 1.43825 | 0.177542748 |
| 1.4383 | 0.175926573 |
| 1.43835 | 0.174308692 |
| 1.4384 | 0.172689128 |
| 1.43845 | 0.171067902 |
| 1.4385 | 0.169445038 |
| 1.43855 | 0.167820560 |
| 1.4386 | 0.166194491 |
| 1.43865 | 0.164566857 |
| 1.4387 | 0.162937682 |
| 1.43875 | 0.161306993 |
| 1.4388 | 0.159674817 |
| 1.43885 | 0.158041180 |
| 1.4389 | 0.156406112 |
| 1.43895 | 0.154769641 |
| 1.439 | 0.153131796 |
| 1.43905 | 0.151492608 |
| 1.4391 | 0.149852108 |
| 1.43915 | 0.148210328 |
| 1.4392 | 0.146567301 |
| 1.43925 | 0.144923060 |
| 1.4393 | 0.143277640 |
| 1.43935 | 0.141631077 |
| 1.4394 | 0.139983406 |
| 1.43945 | 0.138334665 |
| 1.4395 | 0.136684893 |
| 1.43955 | 0.135034128 |
| 1.4396 | 0.133382412 |
| 1.43965 | 0.131729787 |
| 1.4397 | 0.130076294 |
| 1.43975 | 0.128421978 |
| 1.4398 | 0.126766884 |
| 1.43985 | 0.125111059 |
| 1.4399 | 0.123454550 |
| 1.43995 | 0.121797407 |
| 1.44 | 0.120139679 |
| 1.44005 | 0.118481421 |
| 1.4401 | 0.116822684 |
| 1.44015 | 0.115163524 |
| 1.4402 | 0.113503998 |
| 1.44025 | 0.111844164 |
| 1.4403 | 0.110184084 |
| 1.44035 | 0.108523818 |
| 1.4404 | 0.106863432 |
| 1.44045 | 0.105202991 |
| 1.4405 | 0.103542563 |
| 1.44055 | 0.101882219 |
| 1.4406 | 0.100222031 |
| 1.44065 | 0.098562075 |
| 1.4407 | 0.096902428 |
| 1.44075 | 0.095243170 |
| 1.4408 | 0.093584383 |
| 1.44085 | 0.091926154 |
| 1.4409 | 0.090268570 |
| 1.44095 | 0.088611723 |
| 1.441 | 0.086955708 |
| 1.44105 | 0.085300623 |
| 1.4411 | 0.083646570 |
| 1.44115 | 0.081993655 |
| 1.4412 | 0.080341986 |
| 1.44125 | 0.078691676 |
| 1.4413 | 0.077042845 |
| 1.44135 | 0.075395614 |
| 1.4414 | 0.073750111 |
| 1.44145 | 0.072106467 |
| 1.4415 | 0.070464820 |
| 1.44155 | 0.068825314 |
| 1.4416 | 0.067188098 |
| 1.44165 | 0.065553328 |
| 1.4417 | 0.063921166 |
| 1.44175 | 0.062291782 |
| 1.4418 | 0.060665354 |
| 1.44185 | 0.059042066 |
| 1.4419 | 0.057422113 |
| 1.44195 | 0.055805699 |
| 1.442 | 0.054193037 |
| 1.44205 | 0.052584350 |
| 1.4421 | 0.050979875 |
| 1.44215 | 0.049379860 |
| 1.4422 | 0.047784564 |
| 1.44225 | 0.046194265 |
| 1.4423 | 0.044609251 |
| 1.44235 | 0.043029831 |
| 1.4424 | 0.041456331 |
| 1.44245 | 0.039889095 |
| 1.4425 | 0.038328490 |
| 1.44255 | 0.036774908 |
| 1.4426 | 0.035228765 |
| 1.44265 | 0.033690505 |
| 1.4427 | 0.032160605 |
| 1.44275 | 0.030639577 |
| 1.4428 | 0.029127970 |
| 1.44285 | 0.027626379 |
| 1.4429 | 0.026135444 |
| 1.44295 | 0.024655864 |
| 1.443 | 0.023188398 |
| 1.44305 | 0.021733875 |
| 1.4431 | 0.020293208 |
| 1.44315 | 0.018867400 |
| 1.4432 | 0.017457568 |
| 1.44325 | 0.016064955 |
| 1.4433 | 0.014690957 |
| 1.44335 | 0.013337154 |
| 1.4434 | 0.012005351 |
| 1.44345 | 0.010697629 |
| 1.4435 | 0.009416420 |
| 1.44355 | 0.008164610 |
| 1.4436 | 0.006945688 |
| 1.44365 | 0.005763975 |
| 1.4437 | 0.004625002 |
| 1.44375 | 0.003536161 |
| 1.4438 | 0.002507942 |
| 1.44385 | 0.001556535 |
| 1.4439 | 0.000709302 |
| 1.444 | 0 |
| 1.446 | 0.001252721 |
| 1.45 | 0.003324612 |
| 1.454 | 0.004762731 |
| 1.458 | 0.005939231 |
| 1.462 | 0.006969339 |
| 1.466 | 0.007901037 |
| 1.47 | 0.008759232 |
| 1.474 | 0.009558960 |
| 1.478 | 0.010310280 |
| 1.482 | 0.011020412 |
| 1.486 | 0.011694809 |
| 1.49 | 0.012337735 |
| 1.494 | 0.012952621 |
| 1.498 | 0.013542283 |
| 1.502 | 0.014109074 |
| 1.506 | 0.014654988 |
| 1.51 | 0.015181732 |
| 1.514 | 0.015690785 |
| 1.518 | 0.016183437 |
| 1.522 | 0.016660824 |
| 1.526 | 0.017123950 |
| 1.53 | 0.017573711 |
| 1.534 | 0.018010910 |
| 1.538 | 0.018436270 |
| 1.542 | 0.018850444 |
| 1.546 | 0.019254025 |
| 1.55 | 0.019647554 |
| 1.554 | 0.020031526 |
| 1.558 | 0.020406396 |
| 1.562 | 0.020772581 |
| 1.566 | 0.021130467 |
| 1.57 | 0.021480412 |
| 1.574 | 0.021822746 |
| 1.578 | 0.022157779 |
| 1.582 | 0.022485798 |
| 1.586 | 0.022807071 |
| 1.59 | 0.023121850 |
| 1.594 | 0.023430369 |
| 1.598 | 0.023732851 |
| 1.602 | 0.024029504 |
| 1.606 | 0.024320523 |
| 1.61 | 0.024606093 |

**Table 7.** Simulation results for 4-cm sensor over three sensing zones.

| RI @1550 nm | Normalized Power (a.u.) |
| --- | --- |
| 1.3165 | 0.96109828 |
| 1.319 | 0.96011919 |
| 1.3215 | 0.95908476 |
| 1.324 | 0.95799039 |
| 1.3265 | 0.95683095 |
| 1.329 | 0.95560071 |
| 1.3315 | 0.95429323 |
| 1.334 | 0.95290126 |
| 1.3365 | 0.95141655 |
| 1.339 | 0.94982977 |
| 1.3415 | 0.94813017 |
| 1.344 | 0.9463054 |
| 1.3465 | 0.94434108 |
| 1.349 | 0.94222034 |
| 1.3515 | 0.93992316 |
| 1.354 | 0.93742544 |
| 1.3565 | 0.93469761 |
| 1.359 | 0.93170251 |
| 1.3615 | 0.92839168 |
| 1.364 | 0.9246984 |
| 1.3665 | 0.92052202 |
| 1.369 | 0.91568183 |
| 1.37 | 0.91360245 |
| 1.37005 | 0.91348944 |
| 1.3701 | 0.91337622 |
| 1.37015 | 0.91326279 |
| 1.3702 | 0.91314916 |
| 1.37025 | 0.91303532 |
| 1.3703 | 0.91292126 |
| 1.37035 | 0.912807 |
| 1.3704 | 0.91269253 |
| 1.37045 | 0.91257785 |
| 1.3705 | 0.91246296 |
| 1.37055 | 0.91234785 |
| 1.3706 | 0.91223254 |
| 1.37065 | 0.91211701 |
| 1.3707 | 0.91200127 |
| 1.37075 | 0.91188532 |
| 1.3708 | 0.91176915 |
| 1.37085 | 0.91165278 |
| 1.3709 | 0.91153618 |
| 1.37095 | 0.91141937 |
| 1.371 | 0.91130235 |
| 1.37105 | 0.91118511 |
| 1.3711 | 0.91106766 |
| 1.37115 | 0.91094999 |
| 1.3712 | 0.9108321 |
| 1.37125 | 0.91071399 |
| 1.3713 | 0.91059567 |
| 1.37135 | 0.91047713 |
| 1.3714 | 0.91035837 |
| 1.37145 | 0.91023939 |
| 1.3715 | 0.91012019 |
| 1.37155 | 0.91000077 |
| 1.3716 | 0.90988113 |
| 1.37165 | 0.90976127 |
| 1.3717 | 0.90964118 |
| 1.37175 | 0.90952088 |
| 1.3718 | 0.90940035 |
| 1.37185 | 0.9092796 |
| 1.3719 | 0.90915863 |
| 1.37195 | 0.90903743 |
| 1.372 | 0.90891601 |
| 1.37205 | 0.90879436 |
| 1.3721 | 0.90867249 |
| 1.37215 | 0.90855039 |
| 1.3722 | 0.90842806 |
| 1.37225 | 0.90830551 |
| 1.3723 | 0.90818273 |
| 1.37235 | 0.90805972 |
| 1.3724 | 0.90793649 |
| 1.37245 | 0.90781303 |
| 1.3725 | 0.90768933 |
| 1.37255 | 0.90756541 |
| 1.3726 | 0.90744126 |
| 1.37265 | 0.90731687 |
| 1.3727 | 0.90719226 |
| 1.37275 | 0.90706741 |
| 1.3728 | 0.90694233 |
| 1.37285 | 0.90681702 |
| 1.3729 | 0.90669148 |
| 1.37295 | 0.9065657 |
| 1.373 | 0.90643969 |
| 1.37305 | 0.90631344 |
| 1.3731 | 0.90618696 |
| 1.37315 | 0.90606024 |
| 1.3732 | 0.90593328 |
| 1.37325 | 0.90580609 |
| 1.3733 | 0.90567867 |
| 1.37335 | 0.905551 |
| 1.3734 | 0.9054231 |
| 1.37345 | 0.90529495 |
| 1.3735 | 0.90516657 |
| 1.37355 | 0.90503795 |
| 1.3736 | 0.90490909 |
| 1.37365 | 0.90477999 |
| 1.3737 | 0.90465064 |
| 1.37375 | 0.90452106 |
| 1.3738 | 0.90439123 |
| 1.37385 | 0.90426116 |
| 1.3739 | 0.90413085 |
| 1.37395 | 0.90400029 |
| 1.374 | 0.90386949 |
| 1.37405 | 0.90373844 |
| 1.3741 | 0.90360715 |
| 1.37415 | 0.90347561 |
| 1.3742 | 0.90334383 |
| 1.37425 | 0.9032118 |
| 1.3743 | 0.90307952 |
| 1.37435 | 0.90294699 |
| 1.3744 | 0.90281422 |
| 1.37445 | 0.90268119 |
| 1.3745 | 0.90254792 |
| 1.37455 | 0.9024144 |
| 1.3746 | 0.90228062 |
| 1.37465 | 0.9021466 |
| 1.3747 | 0.90201232 |
| 1.37475 | 0.90187779 |
| 1.3748 | 0.90174301 |
| 1.37485 | 0.90160797 |
| 1.3749 | 0.90147268 |
| 1.37495 | 0.90133714 |
| 1.375 | 0.90120134 |
| 1.37505 | 0.90106529 |
| 1.3751 | 0.90092898 |
| 1.37515 | 0.90079241 |
| 1.3752 | 0.90065559 |
| 1.37525 | 0.90051851 |
| 1.3753 | 0.90038117 |
| 1.37535 | 0.90024357 |
| 1.3754 | 0.90010571 |
| 1.37545 | 0.89996759 |
| 1.3755 | 0.89982921 |
| 1.37555 | 0.89969057 |
| 1.3756 | 0.89955167 |
| 1.37565 | 0.89941251 |
| 1.3757 | 0.89927309 |
| 1.37575 | 0.8991334 |
| 1.3758 | 0.89899345 |
| 1.37585 | 0.89885323 |
| 1.3759 | 0.89871275 |
| 1.37595 | 0.898572 |
| 1.376 | 0.89843099 |
| 1.37605 | 0.89828971 |
| 1.3761 | 0.89814816 |
| 1.37615 | 0.89800635 |
| 1.3762 | 0.89786427 |
| 1.37625 | 0.89772191 |
| 1.3763 | 0.89757929 |
| 1.37635 | 0.8974364 |
| 1.3764 | 0.89729324 |
| 1.37645 | 0.89714981 |
| 1.3765 | 0.8970061 |
| 1.37655 | 0.89686213 |
| 1.3766 | 0.89671788 |
| 1.37665 | 0.89657335 |
| 1.3767 | 0.89642856 |
| 1.37675 | 0.89628348 |
| 1.3768 | 0.89613814 |
| 1.37685 | 0.89599251 |
| 1.3769 | 0.89584662 |
| 1.37695 | 0.89570044 |
| 1.377 | 0.89555398 |
| 1.37705 | 0.89540725 |
| 1.3771 | 0.89526024 |
| 1.37715 | 0.89511295 |
| 1.3772 | 0.89496538 |
| 1.37725 | 0.89481753 |
| 1.3773 | 0.8946694 |
| 1.37735 | 0.89452098 |
| 1.3774 | 0.89437229 |
| 1.37745 | 0.89422331 |
| 1.3775 | 0.89407405 |
| 1.37755 | 0.8939245 |
| 1.3776 | 0.89377467 |
| 1.37765 | 0.89362455 |
| 1.3777 | 0.89347415 |
| 1.37775 | 0.89332346 |
| 1.3778 | 0.89317248 |
| 1.37785 | 0.89302122 |
| 1.3779 | 0.89286966 |
| 1.37795 | 0.89271782 |
| 1.378 | 0.89256569 |
| 1.37805 | 0.89241327 |
| 1.3781 | 0.89226055 |
| 1.37815 | 0.89210755 |
| 1.3782 | 0.89195425 |
| 1.37825 | 0.89180066 |
| 1.3783 | 0.89164678 |
| 1.37835 | 0.8914926 |
| 1.3784 | 0.89133813 |
| 1.37845 | 0.89118336 |
| 1.3785 | 0.8910283 |
| 1.37855 | 0.89087294 |
| 1.3786 | 0.89071729 |
| 1.37865 | 0.89056133 |
| 1.3787 | 0.89040508 |
| 1.37875 | 0.89024853 |
| 1.3788 | 0.89009167 |
| 1.37885 | 0.88993452 |
| 1.3789 | 0.88977707 |
| 1.37895 | 0.88961931 |
| 1.379 | 0.88946126 |
| 1.37905 | 0.8893029 |
| 1.3791 | 0.88914423 |
| 1.37915 | 0.88898527 |
| 1.3792 | 0.88882599 |
| 1.37925 | 0.88866641 |
| 1.3793 | 0.88850653 |
| 1.37935 | 0.88834634 |
| 1.3794 | 0.88818584 |
| 1.37945 | 0.88802503 |
| 1.3795 | 0.88786392 |
| 1.37955 | 0.88770249 |
| 1.3796 | 0.88754076 |
| 1.37965 | 0.88737871 |
| 1.3797 | 0.88721635 |
| 1.37975 | 0.88705368 |
| 1.3798 | 0.8868907 |
| 1.37985 | 0.8867274 |
| 1.3799 | 0.88656379 |
| 1.37995 | 0.88639987 |
| 1.38 | 0.88623563 |
| 1.38005 | 0.88607107 |
| 1.3801 | 0.8859062 |
| 1.38015 | 0.88574101 |
| 1.3802 | 0.8855755 |
| 1.38025 | 0.88540967 |
| 1.3803 | 0.88524352 |
| 1.38035 | 0.88507705 |
| 1.3804 | 0.88491027 |
| 1.38045 | 0.88474316 |
| 1.3805 | 0.88457572 |
| 1.38055 | 0.88440797 |
| 1.3806 | 0.88423989 |
| 1.38065 | 0.88407149 |
| 1.3807 | 0.88390276 |
| 1.38075 | 0.8837337 |
| 1.3808 | 0.88356432 |
| 1.38085 | 0.88339461 |
| 1.3809 | 0.88322458 |
| 1.38095 | 0.88305421 |
| 1.381 | 0.88288352 |
| 1.38105 | 0.8827125 |
| 1.3811 | 0.88254114 |
| 1.38115 | 0.88236946 |
| 1.3812 | 0.88219744 |
| 1.38125 | 0.88202509 |
| 1.3813 | 0.88185241 |
| 1.38135 | 0.88167939 |
| 1.3814 | 0.88150604 |
| 1.38145 | 0.88133235 |
| 1.3815 | 0.88115832 |
| 1.38155 | 0.88098396 |
| 1.3816 | 0.88080926 |
| 1.38165 | 0.88063423 |
| 1.3817 | 0.88045885 |
| 1.38175 | 0.88028313 |
| 1.3818 | 0.88010707 |
| 1.38185 | 0.87993067 |
| 1.3819 | 0.87975393 |
| 1.38195 | 0.87957685 |
| 1.382 | 0.87939942 |
| 1.38205 | 0.87922165 |
| 1.3821 | 0.87904353 |
| 1.38215 | 0.87886507 |
| 1.3822 | 0.87868626 |
| 1.38225 | 0.8785071 |
| 1.3823 | 0.8783276 |
| 1.38235 | 0.87814775 |
| 1.3824 | 0.87796754 |
| 1.38245 | 0.87778699 |
| 1.3825 | 0.87760609 |
| 1.38255 | 0.87742483 |
| 1.3826 | 0.87724322 |
| 1.38265 | 0.87706126 |
| 1.3827 | 0.87687895 |
| 1.38275 | 0.87669628 |
| 1.3828 | 0.87651325 |
| 1.38285 | 0.87632987 |
| 1.3829 | 0.87614613 |
| 1.38295 | 0.87596203 |
| 1.383 | 0.87577758 |
| 1.38305 | 0.87559276 |
| 1.3831 | 0.87540759 |
| 1.38315 | 0.87522205 |
| 1.3832 | 0.87503616 |
| 1.38325 | 0.8748499 |
| 1.3833 | 0.87466328 |
| 1.38335 | 0.87447629 |
| 1.3834 | 0.87428894 |
| 1.38345 | 0.87410122 |
| 1.3835 | 0.87391314 |
| 1.38355 | 0.87372469 |
| 1.3836 | 0.87353587 |
| 1.38365 | 0.87334668 |
| 1.3837 | 0.87315713 |
| 1.38375 | 0.8729672 |
| 1.3838 | 0.8727769 |
| 1.38385 | 0.87258623 |
| 1.3839 | 0.87239519 |
| 1.38395 | 0.87220378 |
| 1.384 | 0.87201199 |
| 1.38405 | 0.87181983 |
| 1.3841 | 0.87162729 |
| 1.38415 | 0.87143437 |
| 1.3842 | 0.87124108 |
| 1.38425 | 0.8710474 |
| 1.3843 | 0.87085335 |
| 1.38435 | 0.87065892 |
| 1.3844 | 0.87046411 |
| 1.38445 | 0.87026892 |
| 1.3845 | 0.87007334 |
| 1.38455 | 0.86987738 |
| 1.3846 | 0.86968104 |
| 1.38465 | 0.86948432 |
| 1.3847 | 0.8692872 |
| 1.38475 | 0.86908971 |
| 1.3848 | 0.86889182 |
| 1.38485 | 0.86869355 |
| 1.3849 | 0.86849488 |
| 1.38495 | 0.86829583 |
| 1.385 | 0.86809639 |
| 1.38505 | 0.86789656 |
| 1.3851 | 0.86769633 |
| 1.38515 | 0.86749571 |
| 1.3852 | 0.8672947 |
| 1.38525 | 0.86709329 |
| 1.3853 | 0.86689149 |
| 1.38535 | 0.86668929 |
| 1.3854 | 0.86648669 |
| 1.38545 | 0.8662837 |
| 1.3855 | 0.86608031 |
| 1.38555 | 0.86587651 |
| 1.3856 | 0.86567232 |
| 1.38565 | 0.86546772 |
| 1.3857 | 0.86526273 |
| 1.38575 | 0.86505733 |
| 1.3858 | 0.86485152 |
| 1.38585 | 0.86464531 |
| 1.3859 | 0.8644387 |
| 1.38595 | 0.86423168 |
| 1.386 | 0.86402425 |
| 1.38605 | 0.86381641 |
| 1.3861 | 0.86360816 |
| 1.38615 | 0.86339951 |
| 1.3862 | 0.86319044 |
| 1.38625 | 0.86298096 |
| 1.3863 | 0.86277107 |
| 1.38635 | 0.86256076 |
| 1.3864 | 0.86235004 |
| 1.38645 | 0.86213891 |
| 1.3865 | 0.86192735 |
| 1.38655 | 0.86171539 |
| 1.3866 | 0.861503 |
| 1.38665 | 0.86129019 |
| 1.3867 | 0.86107697 |
| 1.38675 | 0.86086332 |
| 1.3868 | 0.86064925 |
| 1.38685 | 0.86043476 |
| 1.3869 | 0.86021985 |
| 1.38695 | 0.86000451 |
| 1.387 | 0.85978875 |
| 1.38705 | 0.85957256 |
| 1.3871 | 0.85935594 |
| 1.38715 | 0.8591389 |
| 1.3872 | 0.85892143 |
| 1.38725 | 0.85870352 |
| 1.3873 | 0.85848519 |
| 1.38735 | 0.85826643 |
| 1.3874 | 0.85804723 |
| 1.38745 | 0.8578276 |
| 1.3875 | 0.85760753 |
| 1.38755 | 0.85738703 |
| 1.3876 | 0.8571661 |
| 1.38765 | 0.85694472 |
| 1.3877 | 0.85672291 |
| 1.38775 | 0.85650066 |
| 1.3878 | 0.85627797 |
| 1.38785 | 0.85605484 |
| 1.3879 | 0.85583127 |
| 1.38795 | 0.85560725 |
| 1.388 | 0.85538279 |
| 1.38805 | 0.85515789 |
| 1.3881 | 0.85493254 |
| 1.38815 | 0.85470674 |
| 1.3882 | 0.8544805 |
| 1.38825 | 0.85425381 |
| 1.3883 | 0.85402666 |
| 1.38835 | 0.85379907 |
| 1.3884 | 0.85357103 |
| 1.38845 | 0.85334254 |
| 1.3885 | 0.85311359 |
| 1.38855 | 0.85288418 |
| 1.3886 | 0.85265433 |
| 1.38865 | 0.85242401 |
| 1.3887 | 0.85219324 |
| 1.38875 | 0.85196201 |
| 1.3888 | 0.85173033 |
| 1.38885 | 0.85149818 |
| 1.3889 | 0.85126557 |
| 1.38895 | 0.8510325 |
| 1.389 | 0.85079897 |
| 1.38905 | 0.85056497 |
| 1.3891 | 0.85033051 |
| 1.38915 | 0.85009558 |
| 1.3892 | 0.84986018 |
| 1.38925 | 0.84962432 |
| 1.3893 | 0.84938799 |
| 1.38935 | 0.84915119 |
| 1.3894 | 0.84891392 |
| 1.38945 | 0.84867617 |
| 1.3895 | 0.84843796 |
| 1.38955 | 0.84819927 |
| 1.3896 | 0.8479601 |
| 1.38965 | 0.84772046 |
| 1.3897 | 0.84748034 |
| 1.38975 | 0.84723974 |
| 1.3898 | 0.84699867 |
| 1.38985 | 0.84675711 |
| 1.3899 | 0.84651508 |
| 1.38995 | 0.84627256 |
| 1.39 | 0.84602956 |
| 1.39005 | 0.84578607 |
| 1.3901 | 0.8455421 |
| 1.39015 | 0.84529765 |
| 1.3902 | 0.84505271 |
| 1.39025 | 0.84480727 |
| 1.3903 | 0.84456135 |
| 1.39035 | 0.84431494 |
| 1.3904 | 0.84406804 |
| 1.39045 | 0.84382065 |
| 1.3905 | 0.84357276 |
| 1.39055 | 0.84332438 |
| 1.3906 | 0.8430755 |
| 1.39065 | 0.84282613 |
| 1.3907 | 0.84257626 |
| 1.39075 | 0.84232589 |
| 1.3908 | 0.84207502 |
| 1.39085 | 0.84182365 |
| 1.3909 | 0.84157178 |
| 1.39095 | 0.8413194 |
| 1.391 | 0.84106653 |
| 1.39105 | 0.84081314 |
| 1.3911 | 0.84055925 |
| 1.39115 | 0.84030486 |
| 1.3912 | 0.84004995 |
| 1.39125 | 0.83979454 |
| 1.3913 | 0.83953861 |
| 1.39135 | 0.83928218 |
| 1.3914 | 0.83902523 |
| 1.39145 | 0.83876777 |
| 1.3915 | 0.83850979 |
| 1.39155 | 0.8382513 |
| 1.3916 | 0.83799229 |
| 1.39165 | 0.83773277 |
| 1.3917 | 0.83747272 |
| 1.39175 | 0.83721216 |
| 1.3918 | 0.83695107 |
| 1.39185 | 0.83668946 |
| 1.3919 | 0.83642733 |
| 1.39195 | 0.83616467 |
| 1.392 | 0.83590149 |
| 1.39205 | 0.83563778 |
| 1.3921 | 0.83537354 |
| 1.39215 | 0.83510877 |
| 1.3922 | 0.83484348 |
| 1.39225 | 0.83457765 |
| 1.3923 | 0.83431129 |
| 1.39235 | 0.8340444 |
| 1.3924 | 0.83377697 |
| 1.39245 | 0.833509 |
| 1.3925 | 0.8332405 |
| 1.39255 | 0.83297146 |
| 1.3926 | 0.83270189 |
| 1.39265 | 0.83243177 |
| 1.3927 | 0.83216111 |
| 1.39275 | 0.83188991 |
| 1.3928 | 0.83161816 |
| 1.39285 | 0.83134587 |
| 1.3929 | 0.83107303 |
| 1.39295 | 0.83079965 |
| 1.393 | 0.83052572 |
| 1.39305 | 0.83025124 |
| 1.3931 | 0.8299762 |
| 1.39315 | 0.82970062 |
| 1.3932 | 0.82942448 |
| 1.39325 | 0.82914779 |
| 1.3933 | 0.82887055 |
| 1.39335 | 0.82859274 |
| 1.3934 | 0.82831438 |
| 1.39345 | 0.82803547 |
| 1.3935 | 0.82775599 |
| 1.39355 | 0.82747595 |
| 1.3936 | 0.82719534 |
| 1.39365 | 0.82691418 |
| 1.3937 | 0.82663245 |
| 1.39375 | 0.82635015 |
| 1.3938 | 0.82606729 |
| 1.39385 | 0.82578385 |
| 1.3939 | 0.82549985 |
| 1.39395 | 0.82521528 |
| 1.394 | 0.82493014 |
| 1.39405 | 0.82464442 |
| 1.3941 | 0.82435813 |
| 1.39415 | 0.82407126 |
| 1.3942 | 0.82378382 |
| 1.39425 | 0.82349579 |
| 1.3943 | 0.82320719 |
| 1.39435 | 0.82291801 |
| 1.3944 | 0.82262825 |
| 1.39445 | 0.8223379 |
| 1.3945 | 0.82204697 |
| 1.39455 | 0.82175546 |
| 1.3946 | 0.82146336 |
| 1.39465 | 0.82117067 |
| 1.3947 | 0.82087739 |
| 1.39475 | 0.82058352 |
| 1.3948 | 0.82028906 |
| 1.39485 | 0.81999401 |
| 1.3949 | 0.81969836 |
| 1.39495 | 0.81940212 |
| 1.395 | 0.81910529 |
| 1.39505 | 0.81880785 |
| 1.3951 | 0.81850981 |
| 1.39515 | 0.81821118 |
| 1.3952 | 0.81791194 |
| 1.39525 | 0.8176121 |
| 1.3953 | 0.81731166 |
| 1.39535 | 0.81701061 |
| 1.3954 | 0.81670896 |
| 1.39545 | 0.8164067 |
| 1.3955 | 0.81610383 |
| 1.39555 | 0.81580034 |
| 1.3956 | 0.81549625 |
| 1.39565 | 0.81519154 |
| 1.3957 | 0.81488622 |
| 1.39575 | 0.81458029 |
| 1.3958 | 0.81427373 |
| 1.39585 | 0.81396656 |
| 1.3959 | 0.81365877 |
| 1.39595 | 0.81335036 |
| 1.396 | 0.81304133 |
| 1.39605 | 0.81273167 |
| 1.3961 | 0.81242139 |
| 1.39615 | 0.81211048 |
| 1.3962 | 0.81179895 |
| 1.39625 | 0.81148679 |
| 1.3963 | 0.811174 |
| 1.39635 | 0.81086057 |
| 1.3964 | 0.81054652 |
| 1.39645 | 0.81023183 |
| 1.3965 | 0.8099165 |
| 1.39655 | 0.80960054 |
| 1.3966 | 0.80928394 |
| 1.39665 | 0.80896671 |
| 1.3967 | 0.80864883 |
| 1.39675 | 0.80833031 |
| 1.3968 | 0.80801115 |
| 1.39685 | 0.80769134 |
| 1.3969 | 0.80737088 |
| 1.39695 | 0.80704978 |
| 1.397 | 0.80672803 |
| 1.39705 | 0.80640564 |
| 1.3971 | 0.80608259 |
| 1.39715 | 0.80575888 |
| 1.3972 | 0.80543453 |
| 1.39725 | 0.80510952 |
| 1.3973 | 0.80478385 |
| 1.39735 | 0.80445752 |
| 1.3974 | 0.80413053 |
| 1.39745 | 0.80380289 |
| 1.3975 | 0.80347458 |
| 1.39755 | 0.80314561 |
| 1.3976 | 0.80281597 |
| 1.39765 | 0.80248567 |
| 1.3977 | 0.80215469 |
| 1.39775 | 0.80182305 |
| 1.3978 | 0.80149074 |
| 1.39785 | 0.80115776 |
| 1.3979 | 0.8008241 |
| 1.39795 | 0.80048977 |
| 1.398 | 0.80015477 |
| 1.39805 | 0.79981908 |
| 1.3981 | 0.79948272 |
| 1.39815 | 0.79914568 |
| 1.3982 | 0.79880795 |
| 1.39825 | 0.79846954 |
| 1.3983 | 0.79813045 |
| 1.39835 | 0.79779067 |
| 1.3984 | 0.79745021 |
| 1.39845 | 0.79710905 |
| 1.3985 | 0.79676721 |
| 1.39855 | 0.79642467 |
| 1.3986 | 0.79608144 |
| 1.39865 | 0.79573751 |
| 1.3987 | 0.79539289 |
| 1.39875 | 0.79504757 |
| 1.3988 | 0.79470156 |
| 1.39885 | 0.79435484 |
| 1.3989 | 0.79400742 |
| 1.39895 | 0.7936593 |
| 1.399 | 0.79331047 |
| 1.39905 | 0.79296094 |
| 1.3991 | 0.7926107 |
| 1.39915 | 0.79225975 |
| 1.3992 | 0.79190809 |
| 1.39925 | 0.79155571 |
| 1.3993 | 0.79120263 |
| 1.39935 | 0.79084883 |
| 1.3994 | 0.79049431 |
| 1.39945 | 0.79013907 |
| 1.3995 | 0.78978312 |
| 1.39955 | 0.78942644 |
| 1.3996 | 0.78906904 |
| 1.39965 | 0.78871092 |
| 1.3997 | 0.78835207 |
| 1.39975 | 0.7879925 |
| 1.3998 | 0.78763219 |
| 1.39985 | 0.78727116 |
| 1.3999 | 0.78690939 |
| 1.39995 | 0.7865469 |
| 1.4 | 0.78618366 |
| 1.40005 | 0.7858197 |
| 1.4001 | 0.78545499 |
| 1.40015 | 0.78508955 |
| 1.4002 | 0.78472336 |
| 1.40025 | 0.78435643 |
| 1.4003 | 0.78398876 |
| 1.40035 | 0.78362035 |
| 1.4004 | 0.78325118 |
| 1.40045 | 0.78288127 |
| 1.4005 | 0.78251061 |
| 1.40055 | 0.7821392 |
| 1.4006 | 0.78176703 |
| 1.40065 | 0.78139412 |
| 1.4007 | 0.78102044 |
| 1.40075 | 0.78064601 |
| 1.4008 | 0.78027081 |
| 1.40085 | 0.77989486 |
| 1.4009 | 0.77951815 |
| 1.40095 | 0.77914067 |
| 1.401 | 0.77876243 |
| 1.40105 | 0.77838341 |
| 1.4011 | 0.77800363 |
| 1.40115 | 0.77762309 |
| 1.4012 | 0.77724176 |
| 1.40125 | 0.77685967 |
| 1.4013 | 0.7764768 |
| 1.40135 | 0.77609316 |
| 1.4014 | 0.77570873 |
| 1.40145 | 0.77532353 |
| 1.4015 | 0.77493754 |
| 1.40155 | 0.77455078 |
| 1.4016 | 0.77416323 |
| 1.40165 | 0.77377489 |
| 1.4017 | 0.77338576 |
| 1.40175 | 0.77299585 |
| 1.4018 | 0.77260514 |
| 1.40185 | 0.77221365 |
| 1.4019 | 0.77182135 |
| 1.40195 | 0.77142827 |
| 1.402 | 0.77103438 |
| 1.40205 | 0.7706397 |
| 1.4021 | 0.77024421 |
| 1.40215 | 0.76984793 |
| 1.4022 | 0.76945084 |
| 1.40225 | 0.76905294 |
| 1.4023 | 0.76865424 |
| 1.40235 | 0.76825472 |
| 1.4024 | 0.7678544 |
| 1.40245 | 0.76745327 |
| 1.4025 | 0.76705132 |
| 1.40255 | 0.76664855 |
| 1.4026 | 0.76624497 |
| 1.40265 | 0.76584057 |
| 1.4027 | 0.76543535 |
| 1.40275 | 0.76502931 |
| 1.4028 | 0.76462244 |
| 1.40285 | 0.76421475 |
| 1.4029 | 0.76380623 |
| 1.40295 | 0.76339688 |
| 1.403 | 0.7629867 |
| 1.40305 | 0.76257569 |
| 1.4031 | 0.76216385 |
| 1.40315 | 0.76175117 |
| 1.4032 | 0.76133765 |
| 1.40325 | 0.76092329 |
| 1.4033 | 0.7605081 |
| 1.40335 | 0.76009206 |
| 1.4034 | 0.75967517 |
| 1.40345 | 0.75925744 |
| 1.4035 | 0.75883886 |
| 1.40355 | 0.75841944 |
| 1.4036 | 0.75799916 |
| 1.40365 | 0.75757803 |
| 1.4037 | 0.75715604 |
| 1.40375 | 0.7567332 |
| 1.4038 | 0.7563095 |
| 1.40385 | 0.75588494 |
| 1.4039 | 0.75545952 |
| 1.40395 | 0.75503324 |
| 1.404 | 0.75460609 |
| 1.40405 | 0.75417808 |
| 1.4041 | 0.75374919 |
| 1.40415 | 0.75331944 |
| 1.4042 | 0.75288881 |
| 1.40425 | 0.75245732 |
| 1.4043 | 0.75202494 |
| 1.40435 | 0.75159169 |
| 1.4044 | 0.75115756 |
| 1.40445 | 0.75072255 |
| 1.4045 | 0.75028665 |
| 1.40455 | 0.74984987 |
| 1.4046 | 0.74941221 |
| 1.40465 | 0.74897366 |
| 1.4047 | 0.74853421 |
| 1.40475 | 0.74809388 |
| 1.4048 | 0.74765265 |
| 1.40485 | 0.74721053 |
| 1.4049 | 0.74676751 |
| 1.40495 | 0.74632359 |
| 1.405 | 0.74587878 |
| 1.40505 | 0.74543306 |
| 1.4051 | 0.74498643 |
| 1.40515 | 0.7445389 |
| 1.4052 | 0.74409046 |
| 1.40525 | 0.74364111 |
| 1.4053 | 0.74319086 |
| 1.40535 | 0.74273968 |
| 1.4054 | 0.7422876 |
| 1.40545 | 0.74183459 |
| 1.4055 | 0.74138067 |
| 1.40555 | 0.74092582 |
| 1.4056 | 0.74047006 |
| 1.40565 | 0.74001337 |
| 1.4057 | 0.73955575 |
| 1.40575 | 0.73909721 |
| 1.4058 | 0.73863773 |
| 1.40585 | 0.73817733 |
| 1.4059 | 0.73771599 |
| 1.40595 | 0.73725371 |
| 1.406 | 0.7367905 |
| 1.40605 | 0.73632635 |
| 1.4061 | 0.73586126 |
| 1.40615 | 0.73539522 |
| 1.4062 | 0.73492824 |
| 1.40625 | 0.73446031 |
| 1.4063 | 0.73399144 |
| 1.40635 | 0.73352161 |
| 1.4064 | 0.73305083 |
| 1.40645 | 0.7325791 |
| 1.4065 | 0.73210641 |
| 1.40655 | 0.73163277 |
| 1.4066 | 0.73115816 |
| 1.40665 | 0.73068259 |
| 1.4067 | 0.73020606 |
| 1.40675 | 0.72972856 |
| 1.4068 | 0.7292501 |
| 1.40685 | 0.72877066 |
| 1.4069 | 0.72829026 |
| 1.40695 | 0.72780888 |
| 1.407 | 0.72732652 |
| 1.40705 | 0.72684319 |
| 1.4071 | 0.72635888 |
| 1.40715 | 0.72587359 |
| 1.4072 | 0.72538731 |
| 1.40725 | 0.72490005 |
| 1.4073 | 0.7244118 |
| 1.40735 | 0.72392257 |
| 1.4074 | 0.72343234 |
| 1.40745 | 0.72294112 |
| 1.4075 | 0.7224489 |
| 1.40755 | 0.72195569 |
| 1.4076 | 0.72146148 |
| 1.40765 | 0.72096627 |
| 1.4077 | 0.72047006 |
| 1.40775 | 0.71997284 |
| 1.4078 | 0.71947462 |
| 1.40785 | 0.71897538 |
| 1.4079 | 0.71847514 |
| 1.40795 | 0.71797388 |
| 1.408 | 0.71747161 |
| 1.40805 | 0.71696832 |
| 1.4081 | 0.71646402 |
| 1.40815 | 0.71595869 |
| 1.4082 | 0.71545234 |
| 1.40825 | 0.71494497 |
| 1.4083 | 0.71443657 |
| 1.40835 | 0.71392714 |
| 1.4084 | 0.71341668 |
| 1.40845 | 0.71290519 |
| 1.4085 | 0.71239266 |
| 1.40855 | 0.7118791 |
| 1.4086 | 0.7113645 |
| 1.40865 | 0.71084886 |
| 1.4087 | 0.71033217 |
| 1.40875 | 0.70981444 |
| 1.4088 | 0.70929567 |
| 1.40885 | 0.70877584 |
| 1.4089 | 0.70825496 |
| 1.40895 | 0.70773304 |
| 1.409 | 0.70721005 |
| 1.40905 | 0.70668601 |
| 1.4091 | 0.70616091 |
| 1.40915 | 0.70563475 |
| 1.4092 | 0.70510752 |
| 1.40925 | 0.70457923 |
| 1.4093 | 0.70404988 |
| 1.40935 | 0.70351945 |
| 1.4094 | 0.70298795 |
| 1.40945 | 0.70245538 |
| 1.4095 | 0.70192173 |
| 1.40955 | 0.70138701 |
| 1.4096 | 0.7008512 |
| 1.40965 | 0.70031431 |
| 1.4097 | 0.69977634 |
| 1.40975 | 0.69923729 |
| 1.4098 | 0.69869714 |
| 1.40985 | 0.6981559 |
| 1.4099 | 0.69761358 |
| 1.40995 | 0.69707015 |
| 1.41 | 0.69652563 |
| 1.41005 | 0.69598002 |
| 1.4101 | 0.6954333 |
| 1.41015 | 0.69488547 |
| 1.4102 | 0.69433655 |
| 1.41025 | 0.69378651 |
| 1.4103 | 0.69323537 |
| 1.41035 | 0.69268311 |
| 1.4104 | 0.69212974 |
| 1.41045 | 0.69157526 |
| 1.4105 | 0.69101966 |
| 1.41055 | 0.69046293 |
| 1.4106 | 0.68990509 |
| 1.41065 | 0.68934612 |
| 1.4107 | 0.68878602 |
| 1.41075 | 0.6882248 |
| 1.4108 | 0.68766244 |
| 1.41085 | 0.68709896 |
| 1.4109 | 0.68653433 |
| 1.41095 | 0.68596857 |
| 1.411 | 0.68540167 |
| 1.41105 | 0.68483363 |
| 1.4111 | 0.68426444 |
| 1.41115 | 0.68369411 |
| 1.4112 | 0.68312263 |
| 1.41125 | 0.68255 |
| 1.4113 | 0.68197622 |
| 1.41135 | 0.68140128 |
| 1.4114 | 0.68082519 |
| 1.41145 | 0.68024793 |
| 1.4115 | 0.67966952 |
| 1.41155 | 0.67908994 |
| 1.4116 | 0.67850919 |
| 1.41165 | 0.67792728 |
| 1.4117 | 0.6773442 |
| 1.41175 | 0.67675994 |
| 1.4118 | 0.67617451 |
| 1.41185 | 0.67558791 |
| 1.4119 | 0.67500012 |
| 1.41195 | 0.67441116 |
| 1.412 | 0.67382101 |
| 1.41205 | 0.67322967 |
| 1.4121 | 0.67263715 |
| 1.41215 | 0.67204343 |
| 1.4122 | 0.67144853 |
| 1.41225 | 0.67085242 |
| 1.4123 | 0.67025513 |
| 1.41235 | 0.66965663 |
| 1.4124 | 0.66905693 |
| 1.41245 | 0.66845603 |
| 1.4125 | 0.66785392 |
| 1.41255 | 0.6672506 |
| 1.4126 | 0.66664608 |
| 1.41265 | 0.66604034 |
| 1.4127 | 0.66543338 |
| 1.41275 | 0.66482521 |
| 1.4128 | 0.66421582 |
| 1.41285 | 0.66360521 |
| 1.4129 | 0.66299337 |
| 1.41295 | 0.66238031 |
| 1.413 | 0.66176602 |
| 1.41305 | 0.6611505 |
| 1.4131 | 0.66053374 |
| 1.41315 | 0.65991575 |
| 1.4132 | 0.65929652 |
| 1.41325 | 0.65867605 |
| 1.4133 | 0.65805434 |
| 1.41335 | 0.65743139 |
| 1.4134 | 0.65680719 |
| 1.41345 | 0.65618173 |
| 1.4135 | 0.65555503 |
| 1.41355 | 0.65492707 |
| 1.4136 | 0.65429786 |
| 1.41365 | 0.65366739 |
| 1.4137 | 0.65303566 |
| 1.41375 | 0.65240266 |
| 1.4138 | 0.6517684 |
| 1.41385 | 0.65113287 |
| 1.4139 | 0.65049608 |
| 1.41395 | 0.64985801 |
| 1.414 | 0.64921866 |
| 1.41405 | 0.64857804 |
| 1.4141 | 0.64793614 |
| 1.41415 | 0.64729295 |
| 1.4142 | 0.64664849 |
| 1.41425 | 0.64600273 |
| 1.4143 | 0.64535569 |
| 1.41435 | 0.64470736 |
| 1.4144 | 0.64405773 |
| 1.41445 | 0.64340681 |
| 1.4145 | 0.64275459 |
| 1.41455 | 0.64210107 |
| 1.4146 | 0.64144624 |
| 1.41465 | 0.64079011 |
| 1.4147 | 0.64013268 |
| 1.41475 | 0.63947393 |
| 1.4148 | 0.63881387 |
| 1.41485 | 0.6381525 |
| 1.4149 | 0.63748981 |
| 1.41495 | 0.6368258 |
| 1.415 | 0.63616047 |
| 1.41505 | 0.63549381 |
| 1.4151 | 0.63482583 |
| 1.41515 | 0.63415652 |
| 1.4152 | 0.63348588 |
| 1.41525 | 0.6328139 |
| 1.4153 | 0.63214059 |
| 1.41535 | 0.63146594 |
| 1.4154 | 0.63078995 |
| 1.41545 | 0.63011261 |
| 1.4155 | 0.62943393 |
| 1.41555 | 0.62875391 |
| 1.4156 | 0.62807253 |
| 1.41565 | 0.6273898 |
| 1.4157 | 0.62670571 |
| 1.41575 | 0.62602027 |
| 1.4158 | 0.62533346 |
| 1.41585 | 0.6246453 |
| 1.4159 | 0.62395577 |
| 1.41595 | 0.62326487 |
| 1.416 | 0.6225726 |
| 1.41605 | 0.62187896 |
| 1.4161 | 0.62118395 |
| 1.41615 | 0.62048756 |
| 1.4162 | 0.61978979 |
| 1.41625 | 0.61909063 |
| 1.4163 | 0.6183901 |
| 1.41635 | 0.61768818 |
| 1.4164 | 0.61698486 |
| 1.41645 | 0.61628016 |
| 1.4165 | 0.61557406 |
| 1.41655 | 0.61486657 |
| 1.4166 | 0.61415768 |
| 1.41665 | 0.61344739 |
| 1.4167 | 0.61273569 |
| 1.41675 | 0.61202259 |
| 1.4168 | 0.61130808 |
| 1.41685 | 0.61059215 |
| 1.4169 | 0.60987482 |
| 1.41695 | 0.60915607 |
| 1.417 | 0.6084359 |
| 1.41705 | 0.60771431 |
| 1.4171 | 0.6069913 |
| 1.41715 | 0.60626686 |
| 1.4172 | 0.60554099 |
| 1.41725 | 0.60481369 |
| 1.4173 | 0.60408496 |
| 1.41735 | 0.6033548 |
| 1.4174 | 0.6026232 |
| 1.41745 | 0.60189015 |
| 1.4175 | 0.60115567 |
| 1.41755 | 0.60041974 |
| 1.4176 | 0.59968236 |
| 1.41765 | 0.59894353 |
| 1.4177 | 0.59820325 |
| 1.41775 | 0.59746151 |
| 1.4178 | 0.59671832 |
| 1.41785 | 0.59597367 |
| 1.4179 | 0.59522755 |
| 1.41795 | 0.59447997 |
| 1.418 | 0.59373092 |
| 1.41805 | 0.5929804 |
| 1.4181 | 0.59222841 |
| 1.41815 | 0.59147495 |
| 1.4182 | 0.59072001 |
| 1.41825 | 0.58996358 |
| 1.4183 | 0.58920568 |
| 1.41835 | 0.58844629 |
| 1.4184 | 0.58768542 |
| 1.41845 | 0.58692305 |
| 1.4185 | 0.5861592 |
| 1.41855 | 0.58539384 |
| 1.4186 | 0.584627 |
| 1.41865 | 0.58385865 |
| 1.4187 | 0.5830888 |
| 1.41875 | 0.58231745 |
| 1.4188 | 0.58154459 |
| 1.41885 | 0.58077022 |
| 1.4189 | 0.57999433 |
| 1.41895 | 0.57921694 |
| 1.419 | 0.57843803 |
| 1.41905 | 0.57765759 |
| 1.4191 | 0.57687564 |
| 1.41915 | 0.57609216 |
| 1.4192 | 0.57530716 |
| 1.41925 | 0.57452063 |
| 1.4193 | 0.57373256 |
| 1.41935 | 0.57294296 |
| 1.4194 | 0.57215183 |
| 1.41945 | 0.57135915 |
| 1.4195 | 0.57056494 |
| 1.41955 | 0.56976918 |
| 1.4196 | 0.56897187 |
| 1.41965 | 0.56817302 |
| 1.4197 | 0.56737261 |
| 1.41975 | 0.56657065 |
| 1.4198 | 0.56576714 |
| 1.41985 | 0.56496206 |
| 1.4199 | 0.56415543 |
| 1.41995 | 0.56334723 |
| 1.42 | 0.56253746 |
| 1.42005 | 0.56172613 |
| 1.4201 | 0.56091322 |
| 1.42015 | 0.56009874 |
| 1.4202 | 0.55928269 |
| 1.42025 | 0.55846505 |
| 1.4203 | 0.55764584 |
| 1.42035 | 0.55682504 |
| 1.4204 | 0.55600266 |
| 1.42045 | 0.55517869 |
| 1.4205 | 0.55435312 |
| 1.42055 | 0.55352597 |
| 1.4206 | 0.55269722 |
| 1.42065 | 0.55186687 |
| 1.4207 | 0.55103492 |
| 1.42075 | 0.55020136 |
| 1.4208 | 0.5493662 |
| 1.42085 | 0.54852944 |
| 1.4209 | 0.54769106 |
| 1.42095 | 0.54685107 |
| 1.421 | 0.54600946 |
| 1.42105 | 0.54516624 |
| 1.4211 | 0.5443214 |
| 1.42115 | 0.54347493 |
| 1.4212 | 0.54262684 |
| 1.42125 | 0.54177712 |
| 1.4213 | 0.54092578 |
| 1.42135 | 0.5400728 |
| 1.4214 | 0.53921818 |
| 1.42145 | 0.53836193 |
| 1.4215 | 0.53750404 |
| 1.42155 | 0.5366445 |
| 1.4216 | 0.53578332 |
| 1.42165 | 0.5349205 |
| 1.4217 | 0.53405602 |
| 1.42175 | 0.5331899 |
| 1.4218 | 0.53232212 |
| 1.42185 | 0.53145268 |
| 1.4219 | 0.53058159 |
| 1.42195 | 0.52970883 |
| 1.422 | 0.52883441 |
| 1.42205 | 0.52795832 |
| 1.4221 | 0.52708057 |
| 1.42215 | 0.52620114 |
| 1.4222 | 0.52532004 |
| 1.42225 | 0.52443727 |
| 1.4223 | 0.52355281 |
| 1.42235 | 0.52266668 |
| 1.4224 | 0.52177886 |
| 1.42245 | 0.52088936 |
| 1.4225 | 0.51999817 |
| 1.42255 | 0.51910529 |
| 1.4226 | 0.51821072 |
| 1.42265 | 0.51731446 |
| 1.4227 | 0.51641649 |
| 1.42275 | 0.51551683 |
| 1.4228 | 0.51461547 |
| 1.42285 | 0.5137124 |
| 1.4229 | 0.51280763 |
| 1.42295 | 0.51190115 |
| 1.423 | 0.51099295 |
| 1.42305 | 0.51008305 |
| 1.4231 | 0.50917142 |
| 1.42315 | 0.50825808 |
| 1.4232 | 0.50734302 |
| 1.42325 | 0.50642624 |
| 1.4233 | 0.50550773 |
| 1.42335 | 0.5045875 |
| 1.4234 | 0.50366554 |
| 1.42345 | 0.50274184 |
| 1.4235 | 0.50181641 |
| 1.42355 | 0.50088925 |
| 1.4236 | 0.49996034 |
| 1.42365 | 0.4990297 |
| 1.4237 | 0.49809731 |
| 1.42375 | 0.49716318 |
| 1.4238 | 0.4962273 |
| 1.42385 | 0.49528967 |
| 1.4239 | 0.49435029 |
| 1.42395 | 0.49340915 |
| 1.424 | 0.49246626 |
| 1.42405 | 0.49152161 |
| 1.4241 | 0.4905752 |
| 1.42415 | 0.48962702 |
| 1.4242 | 0.48867708 |
| 1.42425 | 0.48772537 |
| 1.4243 | 0.4867719 |
| 1.42435 | 0.48581665 |
| 1.4244 | 0.48485963 |
| 1.42445 | 0.48390083 |
| 1.4245 | 0.48294025 |
| 1.42455 | 0.4819779 |
| 1.4246 | 0.48101376 |
| 1.42465 | 0.48004784 |
| 1.4247 | 0.47908013 |
| 1.42475 | 0.47811063 |
| 1.4248 | 0.47713934 |
| 1.42485 | 0.47616626 |
| 1.4249 | 0.47519138 |
| 1.42495 | 0.4742147 |
| 1.425 | 0.47323623 |
| 1.42505 | 0.47225596 |
| 1.4251 | 0.47127388 |
| 1.42515 | 0.47028999 |
| 1.4252 | 0.4693043 |
| 1.42525 | 0.4683168 |
| 1.4253 | 0.46732749 |
| 1.42535 | 0.46633637 |
| 1.4254 | 0.46534343 |
| 1.42545 | 0.46434867 |
| 1.4255 | 0.46335209 |
| 1.42555 | 0.46235369 |
| 1.4256 | 0.46135347 |
| 1.42565 | 0.46035143 |
| 1.4257 | 0.45934755 |
| 1.42575 | 0.45834185 |
| 1.4258 | 0.45733432 |
| 1.42585 | 0.45632495 |
| 1.4259 | 0.45531375 |
| 1.42595 | 0.45430071 |
| 1.426 | 0.45328584 |
| 1.42605 | 0.45226912 |
| 1.4261 | 0.45125056 |
| 1.42615 | 0.45023016 |
| 1.4262 | 0.44920791 |
| 1.42625 | 0.44818382 |
| 1.4263 | 0.44715787 |
| 1.42635 | 0.44613007 |
| 1.4264 | 0.44510043 |
| 1.42645 | 0.44406892 |
| 1.4265 | 0.44303556 |
| 1.42655 | 0.44200034 |
| 1.4266 | 0.44096327 |
| 1.42665 | 0.43992433 |
| 1.4267 | 0.43888352 |
| 1.42675 | 0.43784086 |
| 1.4268 | 0.43679632 |
| 1.42685 | 0.43574992 |
| 1.4269 | 0.43470165 |
| 1.42695 | 0.4336515 |
| 1.427 | 0.43259949 |
| 1.42705 | 0.4315456 |
| 1.4271 | 0.43048983 |
| 1.42715 | 0.42943218 |
| 1.4272 | 0.42837266 |
| 1.42725 | 0.42731126 |
| 1.4273 | 0.42624797 |
| 1.42735 | 0.4251828 |
| 1.4274 | 0.42411574 |
| 1.42745 | 0.4230468 |
| 1.4275 | 0.42197597 |
| 1.42755 | 0.42090325 |
| 1.4276 | 0.41982864 |
| 1.42765 | 0.41875214 |
| 1.4277 | 0.41767374 |
| 1.42775 | 0.41659345 |
| 1.4278 | 0.41551126 |
| 1.42785 | 0.41442718 |
| 1.4279 | 0.41334119 |
| 1.42795 | 0.41225331 |
| 1.428 | 0.41116353 |
| 1.42805 | 0.41007184 |
| 1.4281 | 0.40897825 |
| 1.42815 | 0.40788275 |
| 1.4282 | 0.40678535 |
| 1.42825 | 0.40568604 |
| 1.4283 | 0.40458482 |
| 1.42835 | 0.4034817 |
| 1.4284 | 0.40237666 |
| 1.42845 | 0.40126971 |
| 1.4285 | 0.40016085 |
| 1.42855 | 0.39905007 |
| 1.4286 | 0.39793738 |
| 1.42865 | 0.39682278 |
| 1.4287 | 0.39570626 |
| 1.42875 | 0.39458782 |
| 1.4288 | 0.39346746 |
| 1.42885 | 0.39234519 |
| 1.4289 | 0.39122099 |
| 1.42895 | 0.39009488 |
| 1.429 | 0.38896684 |
| 1.42905 | 0.38783688 |
| 1.4291 | 0.386705 |
| 1.42915 | 0.38557119 |
| 1.4292 | 0.38443546 |
| 1.42925 | 0.3832978 |
| 1.4293 | 0.38215822 |
| 1.42935 | 0.38101672 |
| 1.4294 | 0.37987328 |
| 1.42945 | 0.37872792 |
| 1.4295 | 0.37758063 |
| 1.42955 | 0.37643142 |
| 1.4296 | 0.37528027 |
| 1.42965 | 0.3741272 |
| 1.4297 | 0.37297219 |
| 1.42975 | 0.37181526 |
| 1.4298 | 0.37065639 |
| 1.42985 | 0.3694956 |
| 1.4299 | 0.36833287 |
| 1.42995 | 0.36716822 |
| 1.43 | 0.36600163 |
| 1.43005 | 0.36483312 |
| 1.4301 | 0.36366267 |
| 1.43015 | 0.36249029 |
| 1.4302 | 0.36131598 |
| 1.43025 | 0.36013973 |
| 1.4303 | 0.35896156 |
| 1.43035 | 0.35778145 |
| 1.4304 | 0.35659942 |
| 1.43045 | 0.35541545 |
| 1.4305 | 0.35422955 |
| 1.43055 | 0.35304172 |
| 1.4306 | 0.35185196 |
| 1.43065 | 0.35066027 |
| 1.4307 | 0.34946666 |
| 1.43075 | 0.34827111 |
| 1.4308 | 0.34707363 |
| 1.43085 | 0.34587423 |
| 1.4309 | 0.34467289 |
| 1.43095 | 0.34346963 |
| 1.431 | 0.34226445 |
| 1.43105 | 0.34105733 |
| 1.4311 | 0.33984829 |
| 1.43115 | 0.33863733 |
| 1.4312 | 0.33742444 |
| 1.43125 | 0.33620963 |
| 1.4313 | 0.3349929 |
| 1.43135 | 0.33377425 |
| 1.4314 | 0.33255368 |
| 1.43145 | 0.33133118 |
| 1.4315 | 0.33010677 |
| 1.43155 | 0.32888045 |
| 1.4316 | 0.3276522 |
| 1.43165 | 0.32642204 |
| 1.4317 | 0.32518997 |
| 1.43175 | 0.32395599 |
| 1.4318 | 0.3227201 |
| 1.43185 | 0.32148229 |
| 1.4319 | 0.32024258 |
| 1.43195 | 0.31900097 |
| 1.432 | 0.31775745 |
| 1.43205 | 0.31651203 |
| 1.4321 | 0.3152647 |
| 1.43215 | 0.31401548 |
| 1.4322 | 0.31276436 |
| 1.43225 | 0.31151134 |
| 1.4323 | 0.31025644 |
| 1.43235 | 0.30899964 |
| 1.4324 | 0.30774095 |
| 1.43245 | 0.30648037 |
| 1.4325 | 0.30521791 |
| 1.43255 | 0.30395357 |
| 1.4326 | 0.30268735 |
| 1.43265 | 0.30141925 |
| 1.4327 | 0.30014927 |
| 1.43275 | 0.29887742 |
| 1.4328 | 0.29760371 |
| 1.43285 | 0.29632812 |
| 1.4329 | 0.29505067 |
| 1.43295 | 0.29377136 |
| 1.433 | 0.2924902 |
| 1.43305 | 0.29120718 |
| 1.4331 | 0.2899223 |
| 1.43315 | 0.28863558 |
| 1.4332 | 0.28734701 |
| 1.43325 | 0.2860566 |
| 1.4333 | 0.28476435 |
| 1.43335 | 0.28347027 |
| 1.4334 | 0.28217435 |
| 1.43345 | 0.28087661 |
| 1.4335 | 0.27957704 |
| 1.43355 | 0.27827566 |
| 1.4336 | 0.27697245 |
| 1.43365 | 0.27566744 |
| 1.4337 | 0.27436062 |
| 1.43375 | 0.27305199 |
| 1.4338 | 0.27174157 |
| 1.43385 | 0.27042935 |
| 1.4339 | 0.26911534 |
| 1.43395 | 0.26779955 |
| 1.434 | 0.26648198 |
| 1.43405 | 0.26516263 |
| 1.4341 | 0.26384151 |
| 1.43415 | 0.26251863 |
| 1.4342 | 0.26119398 |
| 1.43425 | 0.25986759 |
| 1.4343 | 0.25853944 |
| 1.43435 | 0.25720955 |
| 1.4344 | 0.25587792 |
| 1.43445 | 0.25454456 |
| 1.4345 | 0.25320947 |
| 1.43455 | 0.25187266 |
| 1.4346 | 0.25053414 |
| 1.43465 | 0.24919392 |
| 1.4347 | 0.24785199 |
| 1.43475 | 0.24650836 |
| 1.4348 | 0.24516305 |
| 1.43485 | 0.24381606 |
| 1.4349 | 0.2424674 |
| 1.43495 | 0.24111706 |
| 1.435 | 0.23976507 |
| 1.43505 | 0.23841143 |
| 1.4351 | 0.23705614 |
| 1.43515 | 0.23569922 |
| 1.4352 | 0.23434067 |
| 1.43525 | 0.23298049 |
| 1.4353 | 0.23161871 |
| 1.43535 | 0.23025532 |
| 1.4354 | 0.22889033 |
| 1.43545 | 0.22752376 |
| 1.4355 | 0.22615561 |
| 1.43555 | 0.22478589 |
| 1.4356 | 0.22341462 |
| 1.43565 | 0.22204179 |
| 1.4357 | 0.22066742 |
| 1.43575 | 0.21929152 |
| 1.4358 | 0.2179141 |
| 1.43585 | 0.21653517 |
| 1.4359 | 0.21515474 |
| 1.43595 | 0.21377282 |
| 1.436 | 0.21238942 |
| 1.43605 | 0.21100455 |
| 1.4361 | 0.20961823 |
| 1.43615 | 0.20823046 |
| 1.4362 | 0.20684125 |
| 1.43625 | 0.20545063 |
| 1.4363 | 0.20405859 |
| 1.43635 | 0.20266516 |
| 1.4364 | 0.20127034 |
| 1.43645 | 0.19987416 |
| 1.4365 | 0.19847661 |
| 1.43655 | 0.19707772 |
| 1.4366 | 0.19567749 |
| 1.43665 | 0.19427595 |
| 1.4367 | 0.1928731 |
| 1.43675 | 0.19146896 |
| 1.4368 | 0.19006355 |
| 1.43685 | 0.18865688 |
| 1.4369 | 0.18724896 |
| 1.43695 | 0.18583981 |
| 1.437 | 0.18442945 |
| 1.43705 | 0.1830179 |
| 1.4371 | 0.18160516 |
| 1.43715 | 0.18019125 |
| 1.4372 | 0.1787762 |
| 1.43725 | 0.17736002 |
| 1.4373 | 0.17594273 |
| 1.43735 | 0.17452435 |
| 1.4374 | 0.17310489 |
| 1.43745 | 0.17168437 |
| 1.4375 | 0.17026282 |
| 1.43755 | 0.16884024 |
| 1.4376 | 0.16741667 |
| 1.43765 | 0.16599213 |
| 1.4377 | 0.16456663 |
| 1.43775 | 0.16314019 |
| 1.4378 | 0.16171284 |
| 1.43785 | 0.16028459 |
| 1.4379 | 0.15885548 |
| 1.43795 | 0.15742552 |
| 1.438 | 0.15599474 |
| 1.43805 | 0.15456316 |
| 1.4381 | 0.15313081 |
| 1.43815 | 0.15169771 |
| 1.4382 | 0.15026388 |
| 1.43825 | 0.14882936 |
| 1.4383 | 0.14739417 |
| 1.43835 | 0.14595833 |
| 1.4384 | 0.14452188 |
| 1.43845 | 0.14308483 |
| 1.4385 | 0.14164723 |
| 1.43855 | 0.1402091 |
| 1.4386 | 0.13877047 |
| 1.43865 | 0.13733137 |
| 1.4387 | 0.13589183 |
| 1.43875 | 0.13445189 |
| 1.4388 | 0.13301157 |
| 1.43885 | 0.13157092 |
| 1.4389 | 0.13012996 |
| 1.43895 | 0.12868873 |
| 1.439 | 0.12724726 |
| 1.43905 | 0.1258056 |
| 1.4391 | 0.12436378 |
| 1.43915 | 0.12292183 |
| 1.4392 | 0.1214798 |
| 1.43925 | 0.12003773 |
| 1.4393 | 0.11859566 |
| 1.43935 | 0.11715363 |
| 1.4394 | 0.11571168 |
| 1.43945 | 0.11426986 |
| 1.4395 | 0.11282821 |
| 1.43955 | 0.11138678 |
| 1.4396 | 0.10994561 |
| 1.43965 | 0.10850475 |
| 1.4397 | 0.10706426 |
| 1.43975 | 0.10562418 |
| 1.4398 | 0.10418457 |
| 1.43985 | 0.10274548 |
| 1.4399 | 0.10130696 |
| 1.43995 | 0.09986907 |
| 1.44 | 0.09843186 |
| 1.44005 | 0.0969954 |
| 1.4401 | 0.09555975 |
| 1.44015 | 0.09412497 |
| 1.4402 | 0.09269111 |
| 1.44025 | 0.09125826 |
| 1.4403 | 0.08982647 |
| 1.44035 | 0.08839581 |
| 1.4404 | 0.08696636 |
| 1.44045 | 0.08553819 |
| 1.4405 | 0.08411137 |
| 1.44055 | 0.08268598 |
| 1.4406 | 0.0812621 |
| 1.44065 | 0.07983981 |
| 1.4407 | 0.0784192 |
| 1.44075 | 0.07700035 |
| 1.4408 | 0.07558336 |
| 1.44085 | 0.07416831 |
| 1.4409 | 0.07275529 |
| 1.44095 | 0.07134442 |
| 1.441 | 0.06993578 |
| 1.44105 | 0.06852949 |
| 1.4411 | 0.06712565 |
| 1.44115 | 0.06572437 |
| 1.4412 | 0.06432577 |
| 1.44125 | 0.06292997 |
| 1.4413 | 0.06153709 |
| 1.44135 | 0.06014726 |
| 1.4414 | 0.05876061 |
| 1.44145 | 0.05737727 |
| 1.4415 | 0.0559974 |
| 1.44155 | 0.05462114 |
| 1.4416 | 0.05324864 |
| 1.44165 | 0.05188006 |
| 1.4417 | 0.05051557 |
| 1.44175 | 0.04915534 |
| 1.4418 | 0.04779955 |
| 1.44185 | 0.04644839 |
| 1.4419 | 0.04510205 |
| 1.44195 | 0.04376073 |
| 1.442 | 0.04242466 |
| 1.44205 | 0.04109405 |
| 1.4421 | 0.03976913 |
| 1.44215 | 0.03845016 |
| 1.4422 | 0.03713738 |
| 1.44225 | 0.03583108 |
| 1.4423 | 0.03453152 |
| 1.44235 | 0.03323901 |
| 1.4424 | 0.03195387 |
| 1.44245 | 0.03067643 |
| 1.4425 | 0.02940704 |
| 1.44255 | 0.02814607 |
| 1.4426 | 0.02689393 |
| 1.44265 | 0.02565102 |
| 1.4427 | 0.02441781 |
| 1.44275 | 0.02319477 |
| 1.4428 | 0.02198241 |
| 1.44285 | 0.0207813 |
| 1.4429 | 0.01959202 |
| 1.44295 | 0.01841522 |
| 1.443 | 0.01725159 |
| 1.44305 | 0.01610189 |
| 1.4431 | 0.01496696 |
| 1.44315 | 0.0138477 |
| 1.4432 | 0.01274513 |
| 1.44325 | 0.01166036 |
| 1.4433 | 0.01059464 |
| 1.44335 | 0.0095494 |
| 1.4434 | 0.00852626 |
| 1.44345 | 0.00752709 |
| 1.4435 | 0.00655409 |
| 1.44355 | 0.0056099 |
| 1.4436 | 0.00469774 |
| 1.44365 | 0.00382174 |
| 1.4437 | 0.00298734 |
| 1.44375 | 0.00220224 |
| 1.4438 | 0.00147827 |
| 1.44385 | 0.00083586 |
| 1.4439 | 0.00031585 |
| 1.444 | 0 |
| 1.446 | 0.00036307 |
| 1.45 | 0.00145506 |
| 1.454 | 0.00233888 |
| 1.458 | 0.00306393 |
| 1.462 | 0.0036866 |
| 1.466 | 0.00423978 |
| 1.47 | 0.00474291 |
| 1.474 | 0.00520805 |
| 1.478 | 0.00564312 |
| 1.482 | 0.00605355 |
| 1.486 | 0.00644323 |
| 1.49 | 0.00681504 |
| 1.494 | 0.00717118 |
| 1.498 | 0.00751339 |
| 1.502 | 0.00784305 |
| 1.506 | 0.00816131 |
| 1.51 | 0.00846913 |
| 1.514 | 0.00876732 |
| 1.518 | 0.00905657 |
| 1.522 | 0.00933749 |
| 1.526 | 0.00961061 |
| 1.53 | 0.0098764 |
| 1.534 | 0.01013528 |
| 1.538 | 0.01038763 |
| 1.542 | 0.01063379 |
| 1.546 | 0.01087407 |
| 1.55 | 0.01110874 |
| 1.554 | 0.01133807 |
| 1.558 | 0.01156229 |
| 1.562 | 0.01178163 |
| 1.566 | 0.01199628 |
| 1.57 | 0.01220643 |
| 1.574 | 0.01241226 |
| 1.578 | 0.01261393 |
| 1.582 | 0.0128116 |
| 1.586 | 0.0130054 |
| 1.59 | 0.01319548 |
| 1.594 | 0.01338195 |
| 1.598 | 0.01356494 |
| 1.602 | 0.01374455 |
| 1.606 | 0.0139209 |
| 1.61 | 0.01409409 |
